# Neighbourhoods & recovery from psychosis in Trinidad: A qualitative study

**DOI:** 10.1016/j.ssmqr.2023.100373

**Published:** 2024-06

**Authors:** Tessa Roberts, Joni Lee Pow, Casswina Donald, Gerard Hutchinson, Craig Morgan

**Affiliations:** a Unit for Social and Community Psychiatry, Centre for Psychiatry & Mental Health, Wolfson Institute of Population Health, Faculty of Medicine and Dentistry, Queen Mary University of London, London, United Kingdom; b ESRC Centre for Society & Mental Health, Institute of Psychiatry, Psychology & Neuroscience, King's College London, London, United Kingdom; c Department of Psychiatry, The University of the West Indies, St. Augustine, Trinidad and Tobago

## Abstract

• Various aspects of neighbourhood environments influence recovery from psychosis.
• High levels of violence at the neighbourhood level may negatively affect recovery.
• Social cohesion may be a protective factor that promotes recovery.
• Normalisation of cannabis use and easy access to cannabis may also hinder recovery.
• Community involvement is needed to design interventions targeting these factors.

## 1 Introduction

The course of psychotic disorders varies across multiple dimensions, with marked inequalities by place and ethnic and social group, and both clinical and social outcomes remain difficult to predict (Ajnakina et al., 2021; Cohen et al., 2008; Lally et al., 2017; Santesteban-Echarri et al., 2017). Understanding these variations could lead to new strategies to support people with psychosis.

Most research on outcomes for psychosis focusses on clinical and demographic factors, but neighbourhood environments may also be important. In relation to onset, there is evidence that place matters. The incidence of psychosis, for example, varies widely by place (Jongsma et al., 2018; March et al., 2008) (e.g., higher in urban areas within Northern Europe (Heinz et al., 2013); in areas with high levels of deprivation (O'Donoghue et al., 2016) and crime (Bhavsar et al., 2014); and among minority populations living in predominantly majority areas (Baker et al., 2021). However, few studies have explored neighbourhood effects on outcomes.

Some qualitative studies indicate the importance of local context for personal recovery. The personal recovery literature highlights that some people – although by no means all – live full and meaningful lives even with ongoing psychotic experiences (Rossi et al., 2022). A meta-synthesis of qualitative research on neighbourhoods and personal recovery suggests that the places influence recovery by enabling various forms of “doing” (e.g. activities that support recovery), “being” (e.g. feeling safe and at peace), “belonging” (e.g. connecting with others) and “becoming” (fostering hope and purpose) (Doroud et al., 2018). Thus there are multiple pathways through which neighbourhoods may influence recovery, but the specific characteristics that enable these pathways are likely to be context-specific. What constitutes recovery may also vary between settings, as concepts of personal recovery – as a process of growth towards personal goals – evolved in Anglophone countries of the global north (Bayetti et al., 2016). Existing research on neighbourhoods and psychosis originates almost exclusively from Northern Europe. Qualitative research in Switzerland has explored how people with psychosis experience urban environments, implicating crowd density, sensory overload, and obstacles to pedestrian mobility as sources of stress (Söderström, 2019); but it is unclear whether these findings would generalise across diverse settings.

The current study focusses on the Caribbean island of Trinidad, where rates of psychosis are extremely high (Morgan et al., 2023); with major variation by local area (Roberts et al., 2023). Previous qualitative research on psychosis in Trinidad has described causal beliefs around black magic and spirit possession, the contribution of stress or “pressure” to precipitating episodes, its links with vices such as substance use, and also highlights the perceived potential for violence during psychotic episodes which justifies the role of police and the psychiatric hospital in protecting the community (rather than necessarily promoting recovery) (Littlewood, 1988, 2007). Recent studies have emphasised the co-existence of pluralistic understandings of psychosis, both in terms of causes and appropriate strategies to manage the condition (Cohen et al., 2016); which mirrors studies from elsewhere in the Caribbean (Arthur & Whitley, 2015). However, there has been little work exploring the lived experience of psychosis in the Caribbean in relation to their local environments.

This study contributes to the existing evidence by diversifying perspectives on neighbourhoods and mental health beyond Europe and North America, and by identifying targets for intervention to improve outcomes for people with psychosis in Trinidad, and potentially the wider Caribbean.

### 1.1 Objectives

This study aimed to explore:

(1) How people with lived experience of psychosis in Trinidad conceptualise and experience their neighbourhood;
(2) How people with lived experience of psychosis in Trinidad consider their neighbourhood to influence their recovery.

## 2 Methods

### 2.1 Participants and recruitment

Thirty-two participants were purposively sampled from the INTREPID II cohort (Roberts et al., 2020); stratified by municipality. INTREPID II is a multi-site research programme in India, Nigeria and Trinidad to investigate the epidemiology of psychotic disorders across diverse settings. As part of this programme, 212 individuals with a psychotic disorder in Northern Trinidad (Diego Martin, Port of Spain, San Juan/Laventille, Tunapuna/Piarco, Arima, Chaguanas, and Sangre Grande) were recruited and interviewed between 2018 and 2020, then followed up for 2 years.

All participants had a diagnosis of psychotic disorder (with no identifiable organic cause) confirmed by a local psychiatrist following a diagnostic interview, were aged 18–64 at the time of recruitment, resided within the catchment area, and had given permission to be re-contacted for further research. Potential participants were excluded if they lacked capacity to give informed consent due to their current symptom severity, intoxication, or any other reason. We monitored the age and gender profile of the sample during recruitment and attempted to ensure a range of ages and a balance of genders within each municipality.

Potential participants were first approached by a member of the INTREPID II research team who was known to them. Those who expressed interest were then contacted by the lead researcher if they agreed for their contact details to be shared, to invite them to participate in the current study.

### 2.2 Setting

Trinidad and Tobago is a dual-island nation in the southern Caribbean, with a population of approximately 1.37 million. The country is characterised by major social and economic inequalities, which intersect with historical ethnic divisions between the two major groups (Afro-Trinidadians and Indo-Trinidadians). These inequalities are rooted in the colonial legacy of slavery and indentured labour, which has led to ongoing racial segregation and economic hierarchies – with Afro-Trinidadians typically holding least resources – and political divisions along ethnic lines. It is religiously mixed, with large Catholic and Hindu populations in addition to various other Christian denominations and followers of Islam, Orisha and Rastafarianism. The Trinidadian economy is based around the oil industry, with little tourism, unlike much of the rest of the English-speaking Caribbean. Its relatively strong economy has driven immigration from other countries in the region, including increasing numbers from nearby Venezuela in recent years.

**Fig. 1:**
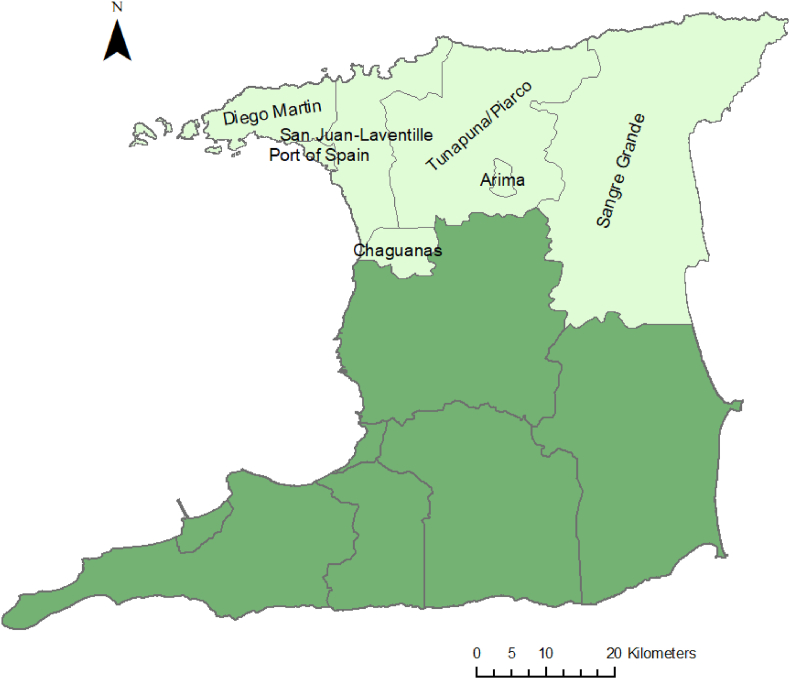
Map of Trinidad, showing INTREPID II catchment area in pale green (from which participants for the current study were recruited). (For interpretation of the references to colour in this figure legend, the reader is referred to the Web version of this article.)

The catchment area for INTREPID II (see Fig. 1), from which participants were sampled, comprises seven municipalities with a diverse population. It includes both densely populated urban areas (e.g. Port of Spain; 3090 persons/sq.km) and rural areas (e.g. Sangre Grande; 82 persons/sq.km). Table 1 describes some characteristics of the municipalities included in the study. The municipalities in the catchment area are described in more detail in the supplementary material.

**Table 1:** Characteristics of municipalities (selected indicators from the 2011 Census of Trinidad & Tobago and Central Statistical Office of Trinidad & Tobago).

	Population	Population density (per sq km)	% Afro-Trinidadian	% secondary education	% tertiary education	Average monthly household consumption expenditure (TT $)	% seeking work	% households with 1 or more people in work	% households renting/leasing (inc rent-free)	% rented govt housing	% running water within house	% only basic sanitation facilities (pit/latrine)	% living at same residence 10 years ago	Median duration of residence (years)	Serious crimes reported per 1000 population^a^	Violent crimes reported per 1000 population^b^
Port of Spain	34,097	2910	54%	70%	18%	6878	4%	61%	42%	15%	92%	7%	78%	20	52	31
Arima	31,259	2784	34%	66%	22%	7458	4%	74%	19%	0%	97%	2%	73%	24	27	19
Chaguanas	80,125	1415	26%	66%	19%	6496	3%	75%	20%	0%	93%	4%	73%	11	13	8
Diego Martin	97,003	812	45%	70%	21%	8733	4%	67%	22%	1%	79%	9%	77%	22	8	5
San Juan/Laventille	149,468	657	55%	64%	14%	7160	4%	72%	32%	3%	80%	9%	75%	30	7	5
Tunapuna/Piarco	182,388	417	37%	66%	20%	7718	3%	66%	23%	23%	93%	7%	76%	16	10	7
Sangre Grande	73,647	82	31%	56%	11%	6809	4%	70%	12%	0%	77%	22%	76%	26	10	8

a Serious crime includes murder, attempted murder, assault, rape, sexual assault and other sexual offences (including incest), serious indecency, kidnapping, burglary, robbery, larceny, fraud, narcotic offences, possession of firearms/ammunition, manslaughter, attempted suicide, malicious damage, arson, perverting the course of justice, misbehaviour in public office, etc. (Note that original CSO definition includes “etc”; no definitive list available).

b Violent crime includes murder, assault, rape, sexual assault and other sexual offences, kidnapping, burglary, robbery, possession of firearms and ammunition. (Classification created by the researchers for the purposes of the current study, and constrained by the original categories used for reporting).

### 2.3 Data collection

Data were collected between January and March 2022. Participants were invited to take part in walking interviews (Evans & Jones, 2011) with the lead author (TR), using a walk-along approach in which participants define the route and answered questions while moving through their neighbourhood. This approach shifts power from interviewer to interviewee by placing the interviewee in the role of “tour guide” and the interviewer as the visitor who follows and learns from the guide, prompts observations that may not be elicited within an interview room, and provides spatial context for the data (Jones et al., 2008). Interviews were semi-structured, using a topic guide designed to address the research questions of the study. In the topic guide, “recovery” was left for participants to interpret as relevant to them. Interviews were audio recorded and subsequently transcribed. The walking route was also captured using a GPS tracker app (Bogdanovich, 2013); which provides timestamped location data that can be linked with the audio transcripts to locate the data geographically.

### 2.4 Ethics

This study was approved by the Health Faculties Research Ethics Sub-Committee at King's College London (HR/DP-21/22–22378) and the Campus Research Ethics Committee at St. Augustine campus, University of the West Indies (CREC-SA.1237/11/2021). Participants were sent an information sheet in advance, and given the opportunity to ask questions before deciding whether to participate. Participants provided written informed consent and were given hard copies of the information sheet to keep. It was emphasised that they did not have to answer any question that they preferred not to answer, and could end the interview at any time. Data were held confidentially on a password-protected computer and backed up on a secure server. No identifying details have been included in this report or any other research outputs.

### 2.5 Analysis

Data were analysed using an inductive approach to thematic analysis (Braun & Clarke, 2006); facilitated by NVivo software (Ltd QIP, 2020). The analysis process proceeded in three stages: describing, organising, and connecting. In the first stage, immersion and familiarisation, the first author summarised the narrative of each interview and took preliminary notes on the main points raised. In the second stage, the lead author organised the data into meaningful themes, in consultation with the co-authors, focussing on neighbourhood-level factors linked to recovery, concepts of neighbourhood and concepts of recovery. These codes were iteratively refined through comparison across interviews, and progressively reorganised to group similar ideas and avoid duplication, until a codebook was developed that incorporated all emergent themes of relevance to the research questions. The full transcripts were then coded using this codebook. Stage three involved exploring and identifying inter-relationships between codes. The themes identified were presented back to participants remotely for feedback and to guide the interpretation of results. For those transcripts with accompanying GPS data, codes and associated quotes were then linked to the GPS route and displayed geographically using ArcGIS (CESRI, 2011). These maps were consulted when synthesising and writing up the findings, and are commented on below. Double-coding was not possible due to resource constraints; this is discussed in the limitations section below.

### 2.6 Positionality

Interviews were conducted by a white British female researcher (TR). This identity provided an “outsider” status, with both benefits and drawbacks. Being an outsider enabled questions to be posed that might seem odd for a Trinidadian researcher to ask. However, it limited participants' willingness to walk in some areas, due to concerns about safety and potential gossip. Being interviewed by a white researcher in areas where white people rarely go may have also influenced the content of the conversations in unanticipated ways; for instance, issues of race and racism were frequently raised despite not being part of the topic guide. The interviewer's gender may have also influenced how participants discussed their experiences, particularly as issues of violence and safety are highly gendered in this context. Female participants may have felt more able to speak freely about sexual assault, for example, and it is possible that male respondents could show more vulnerability than with a male interviewer. For younger participants, the interviewer's age (mid-thirties) may have generated greater formality or deference.

The analysis was led by the same researcher, which brings a specific perspective to the data. While this researcher has lived in various settings internationally and was based in Trinidad for the duration of this project, she was educated in the UK and her worldview is influenced by her public health training and her life experiences as a white British woman. The findings were discussed at various stages with the Trinidadian research team (GH, JLP, CD) and presented back to participants to protect against misunderstandings and contextualise the results, but the interpretation of findings and relative salience of themes may nonetheless have been influenced by the lead researcher's relationship to the data. A female perspective will also have brought a particular lens to the analysis, which may have differed from that of a male researcher exploring the same issues, given the gendered nature of some of the themes.

## 3 Results

### 3.1 Participant characteristics

Table 2 summarises study participants’ characteristics. 22 of the 32 participants agreed to walk during the interview. Those who refused agreed to be interviewed in their home. Reasons for refusing were (a) concerns about gossip, (b) considering neighbourhood irrelevant to their recovery because they did not spend time outside of the home locally, and (c) adverse weather (typically extreme heat/intense sun). Those who agreed to walk generally chose short routes lasting for a minority of the total interview time, due to heat but also in some cases because the places that participants regularly visited were all within a short distance of their home.

**Table 2:** Participant characteristics.

	n (%)
Municipality *Port of Spain* *Diego Martin* *San Juan/Laventille* *Tunapuna/Piarco* *Arima* *Chaguanas* *Sangre Grande*	5 (15.6) 3 (9.4) 6 (18.8) 5 (15.6) 4 (12.5) 4 (12.5) 5 (15.6)
Age group *18–29* *30–39* *≥40*	16 (50.0) 10 (31.3) 6 (18.8)
Gender *Male* *Female*	20 (62.5) 12 (37.5)
Ethnicity *Afro-Trinidadian* *Indo-Trinidadian* *Mixed* *Other*	19 (59.4) 3 (9.4) 9 (28.1) 1 (3.1)
Diagnosis *Affective* *Non-affective* *Substance-induced*	9 (28.1) 14 (43.8) 9 (28.1)
Length of residence in area *Entire life* *>10 years* *5-10 years* *<5 years*	15 (46.9) 8 (25.0) 4 (12.5) 5 (15.6)
Living situation *Alone* *With parents/siblings/other relatives* *With partner and/or children* *Staying at workplace*	1/32 (3.1) 18 (56.3) 12 (37.5) 1/32 (3.1)
Marital status *Married* *In a steady relationship* *Divorced/separated* *Single*	6 (18.8) 4 (12.5) 2 (6.3) 20 (62.5)
Highest education level completed *Primary* *Secondary* *Tertiary or higher*	6 (18.8) 23 (71.9) 3 (9.4)
Employment status *Working (including self-employed)* *Informal or ad hoc employment* *Unemployed* *Full-time studies* *Recently had a baby (unclear if on maternity leave or left work)*	21 (65.6) 6 (18.8) 3 (9.4) 1 (3.1) 1 (3.1)
Treatment status *Not currently using treatment* *Taking some form of medication* *Not taking medication but still engaged in services/therapy* *Unclear*	14 (43.8) 13 (40.6) 2 (6.3) 3 (9.4)

### 3.2 Concepts of recovery

Many participants discussed recovery in terms of symptomatic remission. The symptoms that most concerned interviewees were not always psychotic symptoms. Several participants saw their primary problem as depression or anxiety (although the term “depression” may be used to refer to mental health problems more generally, as it is less stigmatised than psychosis). These participants often described recovery as being free from or able to manage these problems. Other participants had a single psychotic episode which they attributed to substance use, and considered themselves to have recovered after this episode.

There was a common perception that recovery is incompatible with continued medication use. Those who had been told by medical professionals they would need to take medication for life understood this to mean that recovery was impossible.

“What makes you say you can’t recover?”

“No I, I can’t … Once you start taking the injection you have to take the injection right through to maintain that.” (42-year-old male, rural area)

This perception was detrimental to the outlook and self-esteem of such participants, particularly those with a schizophrenia diagnosis, which is highly stigmatised.

Others rejected their diagnosis entirely and therefore either saw recovery as irrelevant or as a process of proving to themselves and others that the professionals were wrong and they were “not mad”.

Many participants discussed issues affecting quality of life that were particularly important to them, e.g. getting a (better) job, moving into better accommodation, and claiming their rights. However, it was not clear whether they considered these broader dimensions of their life to constitute part of their “recovery” per se, as the term is typically used to refer to clinical recovery. Due to the clinical associations of the word “recovery”, during interviews we often asked questions in broader terms such as “overcoming the problems that you have experienced” and “achieving your goals” in addition to asking what helps or hinders their recovery.

### 3.3 Concepts of neighbourhoods

When asked to define their neighbourhood, many interviewees described small areas, often limited to their own street or a couple of neighbouring streets. Places that were over 10 minutes’ walk were described as “far”, and several participants drew sharp distinctions between their neighbourhood and the next, despite being adjacent and within the same ward.

“The racialists is on my side, not out the back on that side. On the back on that side is more African, but here it’s like 50:50 African-Indian … They’re totally, totally different.” (26-year-old male, peri-urban area)

On the other hand, some participants drew much wider boundaries; in one case including the whole island:

“Although I grew up here … I live plenty of places … where people will consider bad areas … So when I think of neighbourhood I don’t think of anywhere specific, I just think of the whole of Trinidad, because I always all over the place in Trinidad, I don’t really have no place I don’t feel safe.” (20-year-old male, rural area)

Defining neighbourhoods was further complicated by several participants reporting multiple addresses (e.g. splitting time between separated parents' houses), or living elsewhere to their official registered address. Other settings were also relevant to interviewees’ recovery, e.g. prisons and the state psychiatric hospital (both typically described in extremely negative terms) and workplaces outside their local area. Non-location0based forms of community were also frequently mentioned, such as virtual/remote relationships or communities based around common interests (e.g. karaoke, Dungeons and Dragons – a fantasy role-playing game).

A substantial proportion of participants described staying at home most of the time. For some this was due to fear of crime (described below); some, who described themselves as introverts, preferred isolation. Experiences of neighbourhoods therefore varied widely among interviewees.

### 3.4 Neighbourhood influences on recovery

**Fig. 2:**
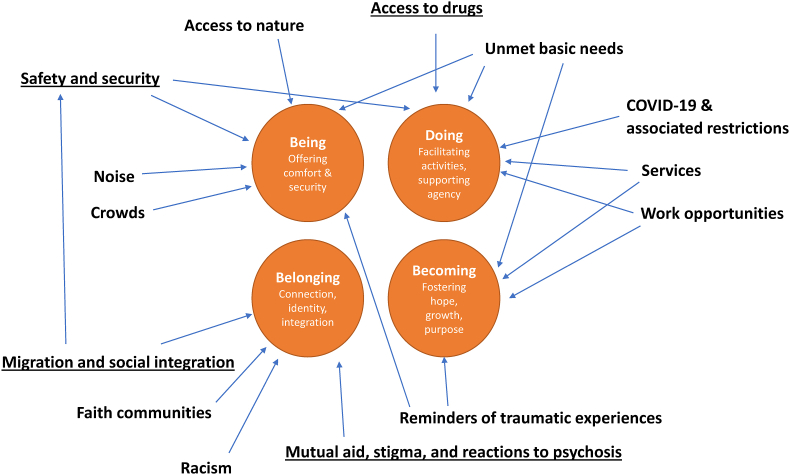
Neighbourhood characteristics and their relationship to Doroud's four dimensions of place and recovery.

Neighbourhood factors that participants linked to recovery are summarised in Fig. 2, organised around the themes identified by Doroud and colleagues (Doroud et al., 2018).

#### 3.4.1 Economic factors

Although Trinidad is a high-income country and most people have access to basic amenities (e.g. electricity access is effectively universal), income inequality was stark in many neighbourhoods, with large, well-built houses interspersed with much smaller and unfinished buildings. However, few participants explicitly linked their neighbourhood's economic situation with their recovery. Exceptions included hostility from neighbours when living in government housing (since demand outstrips supply), and high property prices in some neighbourhoods preventing young people from staying locally if they leave their parents' home, which influenced their experiences of social support (an important influence on mental health, described below). One participant from an affluent neighbourhood argued that his community would tolerate deviation from social norms if he were financially successful (“In Trinidad, if you is a oil man … like if I was working offshore, I could be crazy as a bat!” (26-year-old male, rural area)).

Accessing employment was reportedly dependent on personal connections, and stigma against high-crime neighbourhoods presented a barrier to recruitment for people from these areas. Participants described greater work opportunities in urban areas, leading to an exodus of young people from villages (which affects the social fabric, see “Social integration” below). Some participants said greater availability of good-quality work opportunities locally would enhance their mental health.

Few participants described having their basic needs unmet, but those who did perceived these deprivations to have a major detrimental effect on their mental health. One participant (42-year-old, male, rural area) had no electricity or running water, since his house was built without permission on government land. He carried water uphill daily from the river, and depended on neighbours to fill containers for him during the dry season. He described it as “hard to survive”.

Three participants described the devastating impacts of homelessness on their mental health

“If you stop thinking about trying to survive, trying to stay warm, you wouldn’t believe how much you just sit down and want to die … I was contemplating either committing a crime to go to jail, or killing myself.” (25-year-old male, peri-urban area)

#### 3.4.2 Safety and security

Safety and security were recurrent themes in many interviews. When asked to describe their neighbourhood, almost all participants started by stating how safe or dangerous it is. Violent crime was a major concern for many, who described an increase in violence in recent years:

“Recently we had two deaths that were not - they weren't involved in crime … they were innocent people and they were killed brutally … So I don't really feel it's a nice area to live in any more. It used to be long time, the children used to come out and play in the street, they used to play cricket and other games and now there's no more of that … I don't feel safe any more.” (23-year-old female, urban area)

“Last weekend … a man got shot on the main road … It was not like that, you wouldn’t hear guns shooting in Sangre Grande. But as Sangre Grande started getting … more and more developed, the crime starting going.” (35-year-old female, peri-urban area)

Participants living in high-crime areas described the stress that this caused and its impact on their wellbeing, fuelling a sense of being under threat:

“In this area what has happened over the years, it had a lot of unwanted gang-related activities. And a lot of us have suffered from that … A lot of the neighbours suffered mentally, physically, how they deal with it, and emotionally sometimes. Because it doesn’t be nice to hear. Sometimes somebody who you used to talk to just the other day … you come and hear something happened to them …” (25-year-old male, urban area)

“I get a little scared because the street is very lonely and I always be looking behind me because I don’t know what could happen. And it have fellas up on that side who sell drugs, also, so I just always be … looking back to make sure nobody behind me … I get frustrated and angry to be always – like to be walking on eggshells because you’re not sure what is going to happen.” (43-year-old female, peri-urban area)

“If I know an area have a reputation for having a lot of crime … I don’t sleep good in the night … I always get up at 1–2 in the morning to look out the window to see, to make sure.” (35-year-old female, peri-urban area)

One participant described being kidnapped, while another had been violently assaulted as part of an attempted robbery. Others mentioned having family shot and killed, either by gangs or the police. Several described experiences of rape or sexual assault. Some reported avoiding certain places that reminded them of traumatic experiences. The risk of violence meant that many participants deliberately chose to stay at home for safety:

“Like right out on the main road here you all hear guns, so you know people choose to stay inside.” (35-year-old female, peri-urban area)

“By the time school over, we in … Everybody in, so we can survive … So when they shooting up one another, we in. We don't be out late.” (43-year-old female, urban area)

“I don’t really go anywhere, because I always feel somebody is watching me, or I might get hurt or I might get kidnapped.” (43-year-old female, peri-urban area)

The perceived risk of violence was not uniform across all areas. Living in a relatively safe area was said to facilitate recovery. Participants explained how living somewhere where they knew everyone from childhood provided protection:

“I grew up here so I comfortable here. Like I could walk the road, phone in my hand, money, and not worry about anything.” (20-year-old male, rural area)

“What do people say about Laventille?” “Well, people consider it as a bad area. Because it have plenty of murders and things go on.” “How safe do you feel in this area?” “Well 100%, because I’m born and grow up here.” (21-year-old male, urban area)

“Everybody know everybody. Everybody is some kind of relation, family, so you really don’t have much crime, you can’t have a gang because everybody kind of related.” (41-year-old male, rural area)

“Everybody know me … If I travelling with someone who know me from young, they going to treat me good. Or they wouldn’t think that something wrong with me and take advantage of me … But if I go in the next place, that I don’t know … they might take advantage.” (20-year-old male, peri-urban area)

#### 3.4.3 Access to drugs

Another common theme was the availability of drugs, predominantly marijuana. Several interviewees attributed their psychosis to substance use, and for regular smokers the ubiquity of cannabis made it difficult to quit, increasing the likelihood of future episodes.

“Everyone’s doing it, everyone has it, so it's very easy to buy yourself an episode.” (23-year-old female, urban area)

“The area was a bad influence … They more shoving drugs to you, them kind of things … But here’s the same thing too. Because you can go right here and get weed.” (28-year-old male, urban area)

The extent to which access to drugs varied by area was unclear, however, particularly following the 2019 decriminalisation of cannabis. Even participants who lived in remote rural areas described having easy access to marijuana:

“Everybody plant now, everybody gonna have … Sometimes you going to be getting high off your own supplies, sometimes you’re leaving it to spoil and waste …” (33-year-old male, rural area)

#### 3.4.4 Social integration

As noted, participants' relationships with their neighbours were strongly influenced by migration (mostly internal migration). Newcomers to an area frequently reported more negative experiences than long-term residents, particularly in high-crime areas, reflecting their “outsider” status. Knowing neighbours had some positive impacts on interviewees’ recovery, in terms of safety (described above), and providing a sense of identity and belonging.

“After I come out of St Ann's [psychiatric hospital] it was actually a good thing that I moved back and reconnect with my family. I find back my convictions, my upbringing … These people is like your horcrux [objects that contain fragments of your soul], you go back and you get a piece, and a piece of you.” (20-year-old male, rural area)

Lacking social ties locally was generally described as detrimental to wellbeing, fostering suspicion, mistrust, and isolation:

“When he [partner] not home I doesn’t feel safe. Because it’s different people, every day it’s somebody new moves in. Like if to trust to talk this person or if to pass this person straight.” (31-year-old female, peri-urban area)

“When I was renting it was hard because I don’t know them and I was always the foreigner, and on top of that I’m insane, so I was the loud foreigner, so the neighbours had issues because who’s this person coming to rent and he’s always making noise now?” (26-year-old male, rural area)

“I'm not from here, so the neighbours, as soon as we gone to work, they thieve until we're all out … So it's like you’re not from there so they just do all the wicked things they find they could do to you.” (43-year-old female, urban area)

However, there were downsides to living in a neighbourhood where everybody knows everybody else. One interviewee described major disagreements among the original local community. Others described the negative impact on privacy, particularly in low-income neighbourhoods where houses are close together:

“I will tell you something like my personal business, and then two, three people coming back to tell me exactly what I told you. About my personal business … And all that, it just put a weight on me.” (26-year-old male, peri-urban area)

“So this is why I don’t go out. Instead of making friends here I will make friends outside, because there’s too much of gossip around here.” (29-year-old female, urban area)

“He told them I went to the madhouse …. It a small neighbourhood, I guess they must know because they pass it on.” (37-year-old female, rural area)

Although several participants mentioned “liming” (hanging out, relaxing, or partying) with people from their local area, "liming” together did not necessarily imply a relationship of trust.

Race-related issues were mentioned by several participants, mostly Afro-Trinidadians. There was a dimension of place to these experiences as some interviewees (although not all) described experiencing greater racism in predominantly Indo-Trinidadian areas compared with areas with mostly Afro-Trinidadian residents.

Neighbours’ responses to psychosis were mixed. Some experienced stigmatising responses, which worsened their symptoms and damaged their self-esteem:

“They were not supportive at all, they were afraid. They thought it was something called Obeah … [Caribbean belief system that includes spell-casting and healing, derived from traditional West African practices] They thought I was possessed by something evil … Like my neighbour, she triggers me … Because I was having an episode and she didn’t realise. And she didn’t know what to do other than pray. And when she was praying she was scaring me.” (23-year-old female, urban area)

“They just watch me as a different person.” “Different how?” “A mad person.” (28-year-old male, urban area)

“Sometimes statements, statements that they make. So like if somebody will say boy you’re mad, things, slow …” “And how does that affect you?” “Low confidence.” (25-year-old male, urban area)

Other participants described receiving practical and emotional support from their neighbours, which bolstered their recovery:

“Everybody was really concerned … Everybody was showing support, happy that I was back.” (41-year-old male, rural area)

“People encourage you … like I was thinking I had to stay away from people because they might … You might think they wouldn’t care, they would try to bad talk you … But people might actually want to help you.” (20-year-old male, peri-urban area)

“If somebody like falls sick on the road, like which that time I did, they help. They helped … they carry me by the doctor and everything.” (35-year-old female, peri-urban area)

Although several participants mentioned personal faith as important to their recovery, few mentioned religious communities as a source of support, the exception being Jehovah's Witnesses, who described feeling acceptance and belonging, and receiving guidance and practical help from members of their local Kingdom Hall.

One interviewee believed that he would not be considered mentally ill if he lived somewhere where artistic eccentricity was more accepted, whereas in his rural community his alternative lifestyle fell outside of social norms:

“I personally want to be among people that … my normal is normal, so then I won’t be insane … You can’t really be in neighbourhoods with people who not on that level … They want to call the police away.” (26-year-old male, rural area)

The COVID-19 pandemic and associated restrictions were detrimental to social cohesion, due to the cancellation of community activities, reduced interaction between neighbours, and greater suspicion.

“There used to be more interaction, more physical interaction, and now everyone is just like socially distancing …. Everybody watching everybody like a threat, you could be sick, you don’t know who has it, so we keep our masks on, we only come out to go to the shop.” (23-year-old female, urban area)

Polarisation of opinions around government recommendations reportedly created rifts between community members who took the guidance seriously and those who were sceptical of official narratives. The pandemic was also detrimental to recovery in other ways, as hearing about the deaths of friends, relatives or acquaintances generated grief and increased participants’ sense of insecurity.

However, some benefits of restrictions were noted. One interviewee said that her house was no longer regularly burgled since her children were home due to school closures. Another claimed that deaths due to violent crime were reduced by restrictions on movement.

#### 3.4.5 Environmental factors

Noise (e.g. loud music, rituals from religious institutions) was mentioned by several participants, who described its impact on sleep and low-level irritation. Many participants wanted to live somewhere quiet and peaceful, and considered tranquillity to be helpful to their recovery.

Several interviewees mentioned finding relaxation through being in nature. The catchment area includes many areas of natural beauty, and several neighbourhoods had a backdrop of lushly forested hills (referred to as “the bush”) or a nearby coastline. The beach was often mentioned as therapeutic. In Trinidad many lower-income neighbourhoods are located at high altitudes, and some participants from these areas said their sea views increased their quality of life.

#### 3.4.6 Services

Mental health services were sometimes mentioned as a facilitator of recovery, but equally often as a barrier. No geographic barriers to accessing mental health care were raised, even by those living in remote rural locations. Difficulties in accessing talking therapy were mentioned (treatment mostly consisted of medication) but were not area-specific.

The psychiatric hospital, St Ann's, was described as a place of restricted freedoms, lack of dignity, and coercion. It was also said to enable rest, however, which some participants saw as key to recovery. Several participants likened the hospital environment to prison, and avoiding readmission was a key priority for their recovery.

Some participants were frustrated by how medical professionals did not ask about their lives to put their mental health into context. They reported that the psychiatric system takes an acontextual approach to understanding distress, offering only medication as a solution and failing to recognise the social origins of their problems.

The only other service mentioned was the police. Participants often described the police critically, as corrupt or incompetent. Nonetheless, participants said living close to police stations made them feel safer.

### 3.5 Findings in spatial context

In this study, the spatial aspect of transcripts added little to the analysis beyond the traditional approach using NVivo. No patterns were noted regarding the places in which people were located when mentioning particular themes.

## 4 Discussion

This is the first study to explore the role of neighbourhoods in recovery from psychosis in the Caribbean. It adds to existing evidence on place and mental health by documenting the perceptions of people with experience of psychosis, in a context with high rates of psychosis, particularly in urban areas (Morgan et al., 2023; Roberts et al., 2023). For the purposes of analysing and interpreting our data, we defined recovery broadly as having the ability to do or be that which the person considers valuable (Hopper, 2007). According to this approach, whether people can lead the sort of life that they want to lead depends on the interaction between people's individual characteristics or psychological state and their wider social environment, which includes structural barriers such as discrimination and inequalities that determine whether people living with psychosis can lead fulfilling lives within the social environments they occupy. We found several neighbourhood characteristics may be relevant to recovery, including exposure to high levels of violence, the strength of local social ties (which was linked with migration), and access to cannabis and other drugs. This is consistent with previous research (Doroud et al., 2018) but furthers our understanding of the factors most salient for people with psychosis in Trinidad. We also found varying conceptualisations of neighbourhoods, with several participants defining their neighbourhoods far more narrowly than arbitrary administrative boundaries.

## 5 Limitations

The spatial data were of limited utility. The reasons for this include: Firstly, ethically we could not ask participants to visit places where they felt uncomfortable. As a result, walking routes were within a small radius of interviewees’ homes, within areas they felt safe. Although participants described places where they felt threatened or anxious, we did not visit those locations and therefore the GPS data could not distinguish these. Secondly, the tone of interviews shifted when people were in public places, compared with when interviewed at home. During walks, conversations became less introspective and more factual, with information rarely linked explicitly to their psychological state. Finally, most interviewees said their mental health was influenced by general neighbourhood characteristics such as crime levels, rather than by specific locations within the neighbourhood, with few exceptions (e.g. places that reminded them of traumatic experiences).

Furthermore, the study was restricted to participants with diagnosed psychotic disorder, with no comparison group. Participants’ perceptions of their neighbourhood may have been influenced by symptoms such as paranoia, and their perceptions may not be widely shared. Paradoxically, previous research in Trinidad has found that fear of crime among the general population is higher in low-crime than high-crime neighbourhoods (Chadee, 2003).

Finally, analyses were primarily conducted by the lead author (TR). To protect against misinterpretation, the lead researcher consulted with the Trinidadian members of the team throughout data collection and analysis and held two online feedback sessions with participants to invite their reflections on preliminary findings before the analysis was completed.

## 6 Comparison with existing literature

### 6.1 Crime and violence

Our findings suggest that living in a neighbourhood with higher rates of violent crime may hinder recovery. For context, Trinidad has experienced a dramatic rise in violent crime over the past two decades, and now has one of the highest homicide rates in the world (Baird, 2020; Caribbean, 2012). There appears to have been a parallel rise in the incidence of psychosis over this period (Bhugra et al., 1996; Morgan et al., 2023). The urban municipalities that have been most affected by gang violence also have the highest rates of psychosis (Roberts et al., 2023).

Our findings are consistent with previous evidence linking violence with the onset of psychosis. Exposure to violence during childhood is an established risk factor for psychosis (Morgan & Gayer‐Anderson, 2016). Preliminary evidence suggests that violence and perceived threat at both the individual and neighbourhood levels may influence mental health outcomes throughout the life course (Baranyi et al., 2020; Conley et al., 2022); and victims of violent crime report more psychotic experiences than controls across diverse settings (DeVylder et al., 2018). The evidence on neighbourhood crime and psychosis is limited, but a positive association has been reported (Baranyi et al., 2021). A UK longitudinal study found that the cumulative effects of personal crime victimization and social cohesion and disorder may explain higher rates of psychotic experiences in more urban neighbourhoods (Newbury et al., 2018).

Moving further upstream, there is evidence of a relationship between economic inequality and rates of violence (Wilkinson, 2004); which may at least partially explain the associations observed elsewhere between inequality and rates of psychosis (Burns & Esterhuizen, 2008). Our findings lend some support to the hypothesis that rising inequality may contribute to increasing rates of psychosis by corroding social relationships within communities and increasing the likelihood of violence.

Prior literature on personal recovery emphasises the importance of both objective and perceived safety (Dell et al., 2021; Dixon et al., 2021). Safety is also emphasised under “place for being” in Doroud et al.‘s framework (Doroud et al., 2018) (see Fig. 2). The findings from this and other studies suggest that ensuring the safety of people with psychosis – in services and the community – is essential to enable recovery.

### 6.2 Social cohesion/social capital

Our finding that greater social cohesion may buffer the effect of dangerous neighbourhoods is in line with research on subclinical psychotic experiences (Marsh et al., 2022) and mental health in general (Latham et al., 2022). Some studies report a negative association between the incidence of psychosis and social cohesion (Boydell et al., 2002); while others suggest that high social cohesion may increase the risk of psychosis (Kirkbride et al., 2008); potentially because more cohesive majority groups exclude minorities more effectively. The double-edged nature of social cohesion was also highlighted in our findings. Our findings suggest that, in Trinidad, engagement in social activities is less relevant than the security and sense of identity provided by knowing and trusting one's neighbours.

Existing evidence on social capital and the course of psychosis is limited (Rotenberg et al., 2020); but social cohesion and connection fit within “place for belonging” (Doroud et al., 2018); and feature prominently in many models of personal recovery (Leamy et al., 2011; Vera San Juan et al., 2021). The current study found that while neighbourhood social cohesion might promote recovery, individual experiences vary substantially within neighbourhoods.

Previous studies from Europe and North America have documented deliberate withdrawal from urban life as a strategy for coping with psychosis (Corin, 1990) in response to the stress induced by crowding and noise (Söderström, 2019). Our findings suggest that social withdrawal and the creation of safe spaces are also relevant coping strategies for people living with psychosis in Trinidad, but suggest that in this context staying within one's local area is not solely driven by managing over-stimulation, but also the threat of violence.

### 6.3 Substance use

Substance use was also identified as important. Cannabis use is associated with worse outcomes for psychosis (Hasan et al., 2020) and may contribute to the high incidence of psychosis in Trinidad (Pow et al., 2023). Greater availability of cannabis – particularly high-potency strains and synthetic cannabinoids (Murray et al., 2016) – is likely to worsen outcomes for psychosis. The same may apply to other illicit substances that contribute to psychosis risk. If access to such substances varies by neighbourhood, this may lead to local inequalities in rates of clinical and personal recovery.

### 6.4 Discrepancies with existing evidence

Some themes were notably absent from participant interviews. Firstly, access to services was not raised; this may be because Trinidad has a relatively accessible public mental health system in all municipalities. While formal public transport is limited, “maxi taxis” (private minibuses) operate in all areas, enabling those without cars to travel relatively easy. Secondly, neighbourhood deprivation and inequality were not directly mentioned. Deprivation may however be associated with reduced social cohesion and higher levels of violent crime (Maguire-Jack et al., 2022); with these factors mediating an association between psychosis and neighbourhood deprivation. Thirdly, no interviewee raised the visual appearance of the neighbourhood as relevant to their recovery (O'Brien et al., 2019). However, we did find evidence that fear of crime can lead to social retreat, and that living in stressful contexts impacts negatively on mental health, which are two mechanisms by which neighbourhood disorder is hypothesised to generate mental health inequalities (Massey, 2004).

### 6.5 Operationalising neighbourhoods

Finally, we found that research that uses administrative boundaries to define neighbourhoods fails to reflect residents’ own conceptualisations of their neighbourhoods. Frequent references to small-scale geographic areas (similar to “urban bubbles” in London (Whitley et al., 2005) suggest that more fine-grained and flexible analyses are needed to fully capture neighbourhood processes in Trinidad, respecting the varied ways in which participants think about their neighbourhoods, and acknowledging that neighbourhoods are to a large extent experienced as social rather than spatial. This has potentially important implications for the delivery of mental health services, which are organised by geographic catchment area, and might benefit from shifting away from a place-based approach to a more community-based approach that builds from existing social structures. Asset mapping techniques (Lightfoot et al., 2014) could be a promising avenue in mapping community networks and building support systems around these.

An important neighbourhood variable implicated by this analysis is local rates of violent crime, and future neighbourhood studies should analyse this at a small scale (e.g. police station catchment areas, rather than municipality), while being mindful of the limitations of such boundaries in capturing the variable experiences of residents.

## 7 Implications for policy and practice

Our results suggest that public health strategies to promote recovery from psychosis in Trinidad (and similar contexts) should prioritise ensuring the safety of people affected by mental illness, and of their wider communities. This would involve both taking a trauma-informed approach to the design and delivery of clinical services, to avoid retraumatising people who have been affected by violence, and implementing public health measures to reduce the violence to which people are exposed in the community. These results further point to the need for interventions to facilitate greater social cohesion and the integration of new migrants from other areas – particularly within high-risk neighbourhoods – as well as support for mental health service users who use cannabis regularly, who currently receive little support to reduce their dependence. Working with communities to identify contextually-appropriate strategies to build more mental-health-promoting neighbourhoods will be critical to ensure that such policies are effective and informed by the local cultural context; for example, beliefs about the healing power of cannabis and spiritual contributions to mental illness.

Our findings also point to the need to target additional resources towards neighbourhoods with high rates of violent crime and psychosis. Many of the factors identified in this study have previously been linked with the onset of psychosis, engagement with services, and other mental health conditions, which suggests that such interventions could have multiple complementary benefits. To our knowledge, no studies have attempted to evaluate the impact of neighbourhood-level interventions on psychosis outcomes, although emerging evidence suggests that neighbourhood regeneration projects can reduce mental health inequalities (not psychosis specifically), potentially by reducing neighbourhood disorder (Greene et al., 2020). Evaluating community-led strategies to tackle the major social determinants of mental health in Trinidad and the wider Caribbean is an important avenue for future research.

## 8 Conclusion

This study found that people with psychosis in Trinidad perceive various aspects of their neighbourhoods to be relevant to their recovery. The most prominent neighbourhood characteristics raised were crime and safety, social connectedness or cohesion (which was linked with migration), and access to drugs. These findings are broadly consistent with evidence from other settings, but the relative salience of these specific factors reflects the contemporary Caribbean context.

## Funding

This work was supported by a 10.13039/501100000286British Academy fellowship (grant number: PF21\210001). It was nested within the INTREPID II programme, which was funded by the UK 10.13039/501100000265Medical Research Council (MRC) (MRC Reference: MR/PO25927/1).

## Data statement

The data used in this analysis are available upon reasonable request from the first author (with identifying details removed).

## CRediT authorship contribution statement

**Tessa Roberts:** Conceptualization, Data curation, Formal analysis, Funding acquisition, Investigation, Methodology, Project administration, Writing – original draft, Writing – review & editing. **Joni Lee Pow:** Investigation, Project administration, Writing – review & editing. **Casswina Donald:** Investigation, Project administration, Writing – review & editing. **Gerard Hutchinson:** Supervision, Writing – review & editing. **Craig Morgan:** Conceptualization, Funding acquisition, Supervision, Writing – review & editing.

## Declaration of competing interest

The authors declare that they have no known competing financial interests or personal relationships that could have appeared to influence the work reported in this paper.
