# Supplementary material for: Neighbourhoods & recovery from psychosis in Trinidad: A qualitative study
Source: SSM Qual Res Health. 2024 Jun;5:100373. doi: 10.1016/j.ssmqr.2023.100373 (PMC11190840; doi:10.1016/j.ssmqr.2023.100373)
Supplement: Multimedia component 1 [file mmc1.docx]

# Initial topic guide

## Defining neighbourhoods (before the walk)

1. Have you always lived in this area? Would you describe it as a good place to live? Why/why not? What do people say about this area?
2. What do you think of as your neighbourhood? Where does it start and end? Do you consider everyone who lives in [local administrative area] to be part of your neighbourhood?
3. Do you feel like you belong in [local administrative area]? Why/why not? Would you describe the people who live in this area as a community? What is that community like?

## Interaction with neighbourhood (during the walk)

1. Can you show me the places that you go to during a typical week? Please talk me through why you go to each place, how often you go there, and what you do there.
2. Are there any places in your neighbourhood that you deliberately avoid? Why?
3. What proportion of your time do you spend within your local neighbourhood? Which places do you go to outside of the neighbourhood, and how much time do you spend there in a typical week?

## Experiences of neighbourhood (during the walk)

For each place:

- Can you tell me whether you feel safe there and why/why not?
- Can you tell me whether you feel included there (i.e. do you feel like you are welcome/an insider or an outsider?) and why/why not?
- Is this a place that you enjoy visiting/look forward to going to, or that you dislike/dread going to? Why is that?
- Can you tell me about the people that you encounter here – who do you usually see in each place? Do you usually speak to them? How do they react to you? How do you feel when you see them?
- How does the look of the place make you feel? E.g. the buildings (how tall/low they are, how well-built/run-down they are, etc.), the streets (how wide they are, how clean/dirty they are, etc.), any green space or other features of the area.
- Do you experience more or less of [hearing voices/seeing things that others don’t see etc – insert unusual experiences reported previously by participant] in this place? Why do you think these places increase or decrease these experiences?

## Reflections on neighbourhood and recovery (after the walk)

1. Do you believe that the place where you live helps you to overcome the problems that you’ve been experiencing? Why/why not?
2. Are there any changes that you would like to see to your area that would help to support your recovery? How would these help?
3. Do you consider your local community to be helpful or harmful to your recovery? Is there anything that people in your neighbourhood could do differently to help you to recover?
