# Supplementary material for: Neighbourhoods & recovery from psychosis in Trinidad: A qualitative study
Source: SSM Qual Res Health. 2024 Jun;5:100373. doi: 10.1016/j.ssmqr.2023.100373 (PMC11190840; doi:10.1016/j.ssmqr.2023.100373)
Supplement: Multimedia component 2 [file mmc2.pdf]

Below is a collection of photographs of the catchment area for the study, taken by a member of the INTREPID II research team in March 2020. These were not categorised by municipality, but provide some context for those who are not familiar with the Trinidadian setting.

Photo credit: Dr Georgina Miguel Esponda

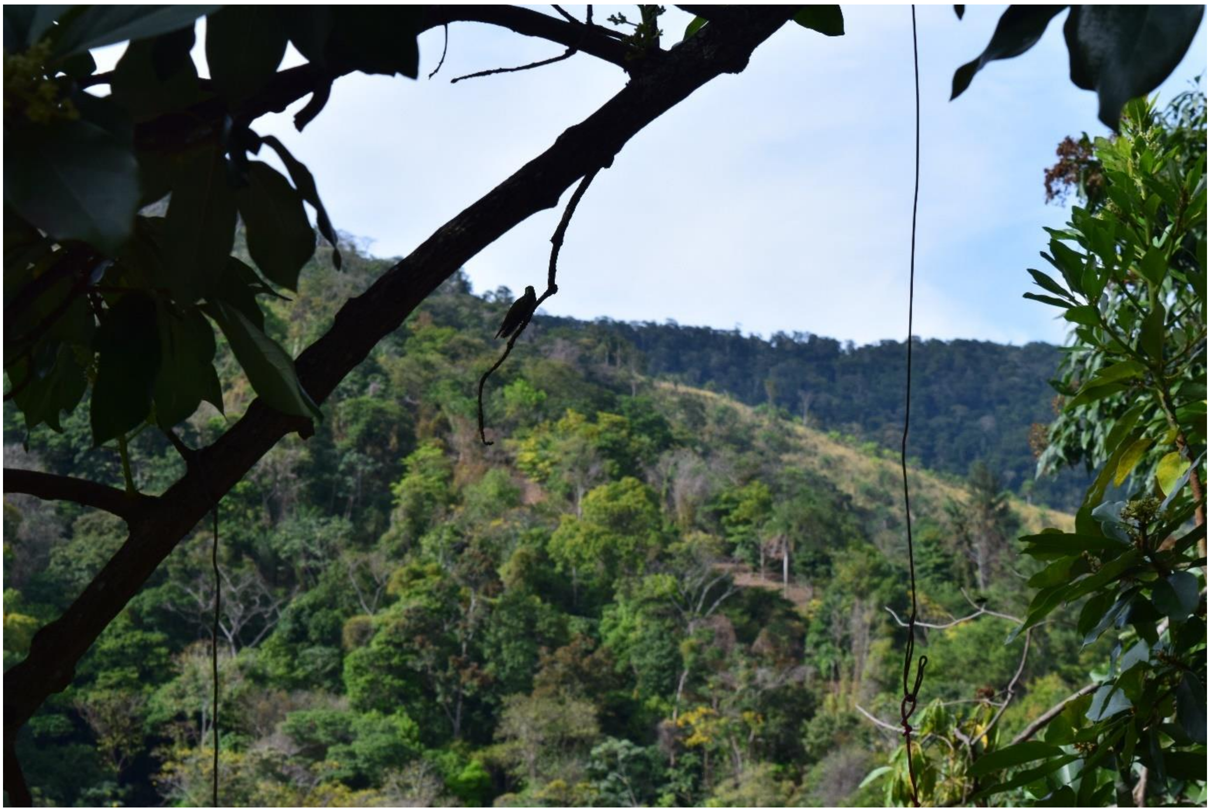

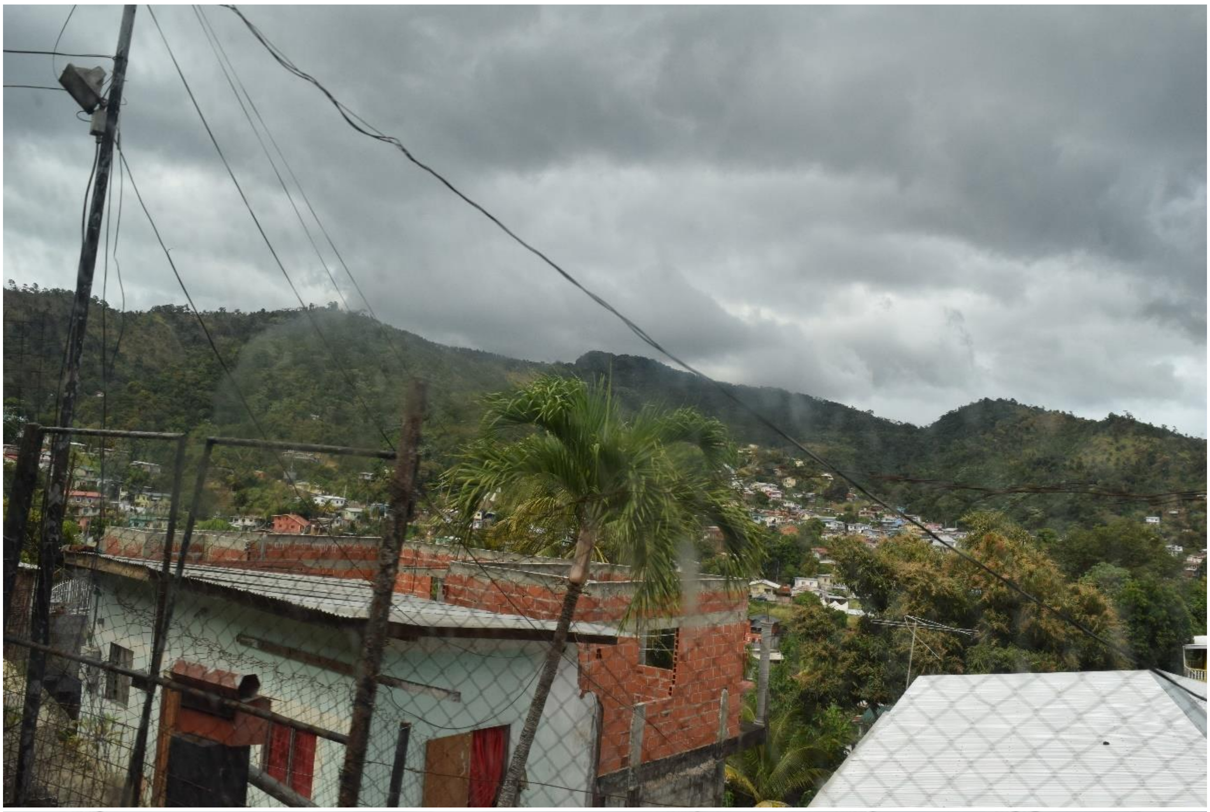

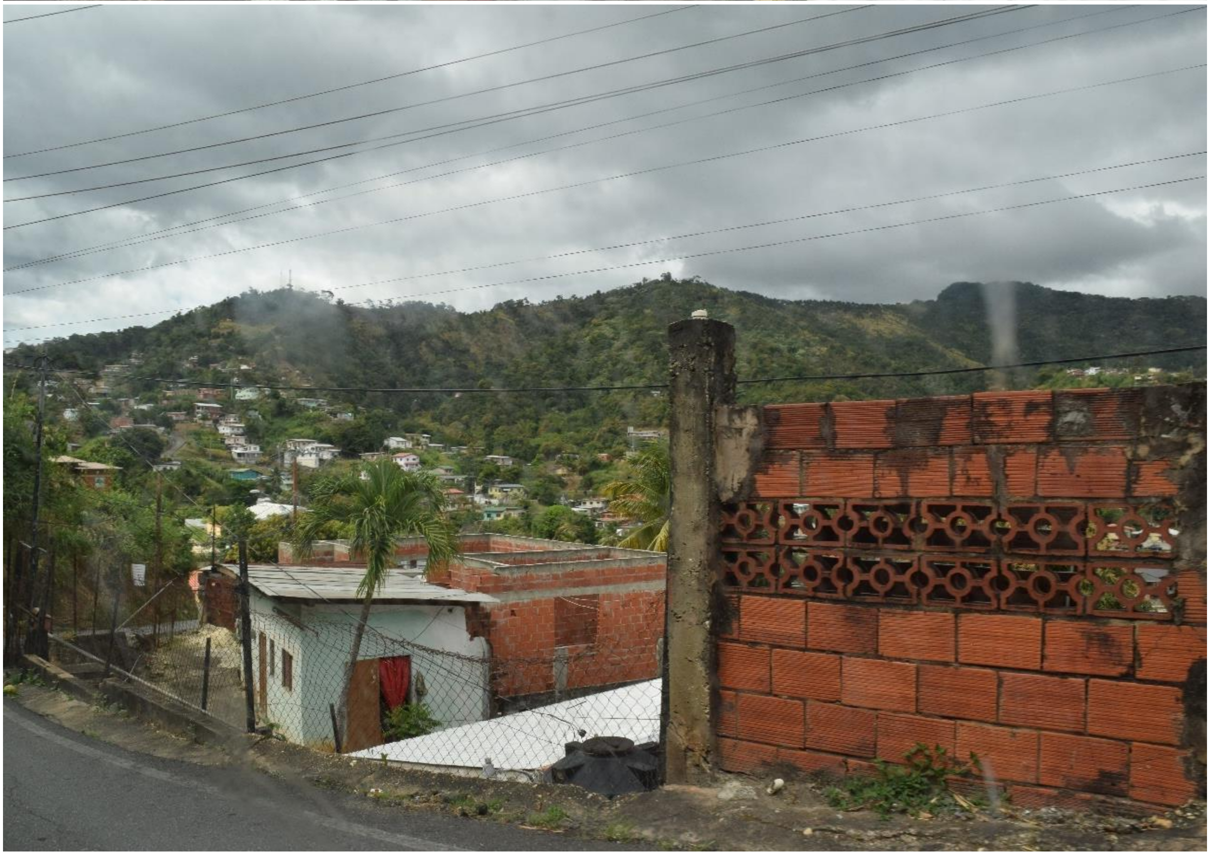

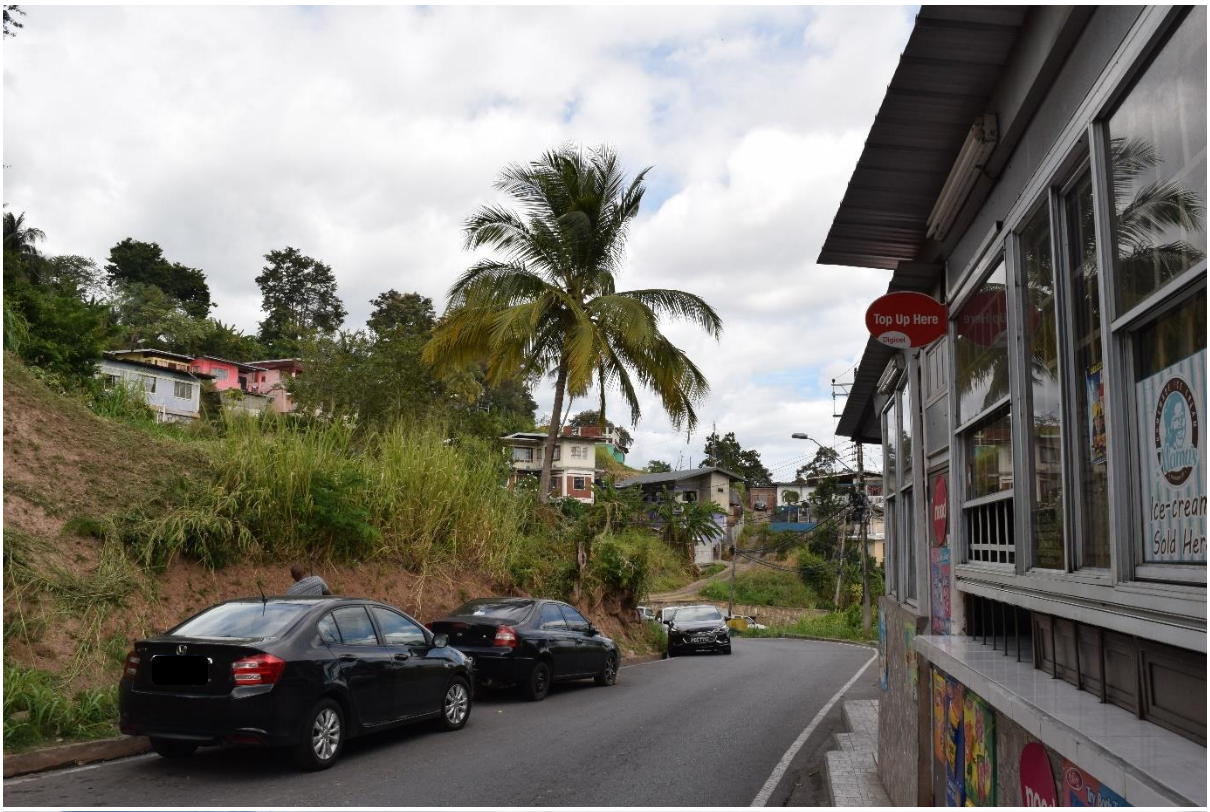
Top Up Here
Digicel

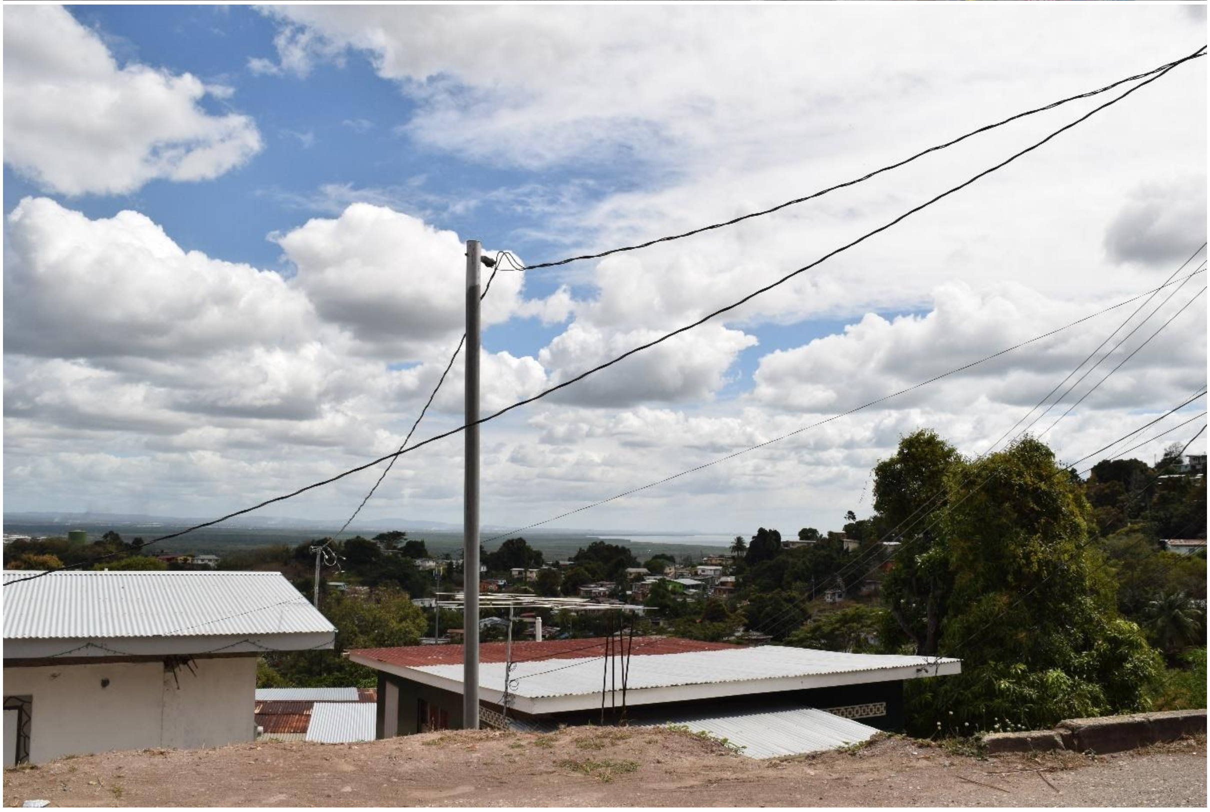

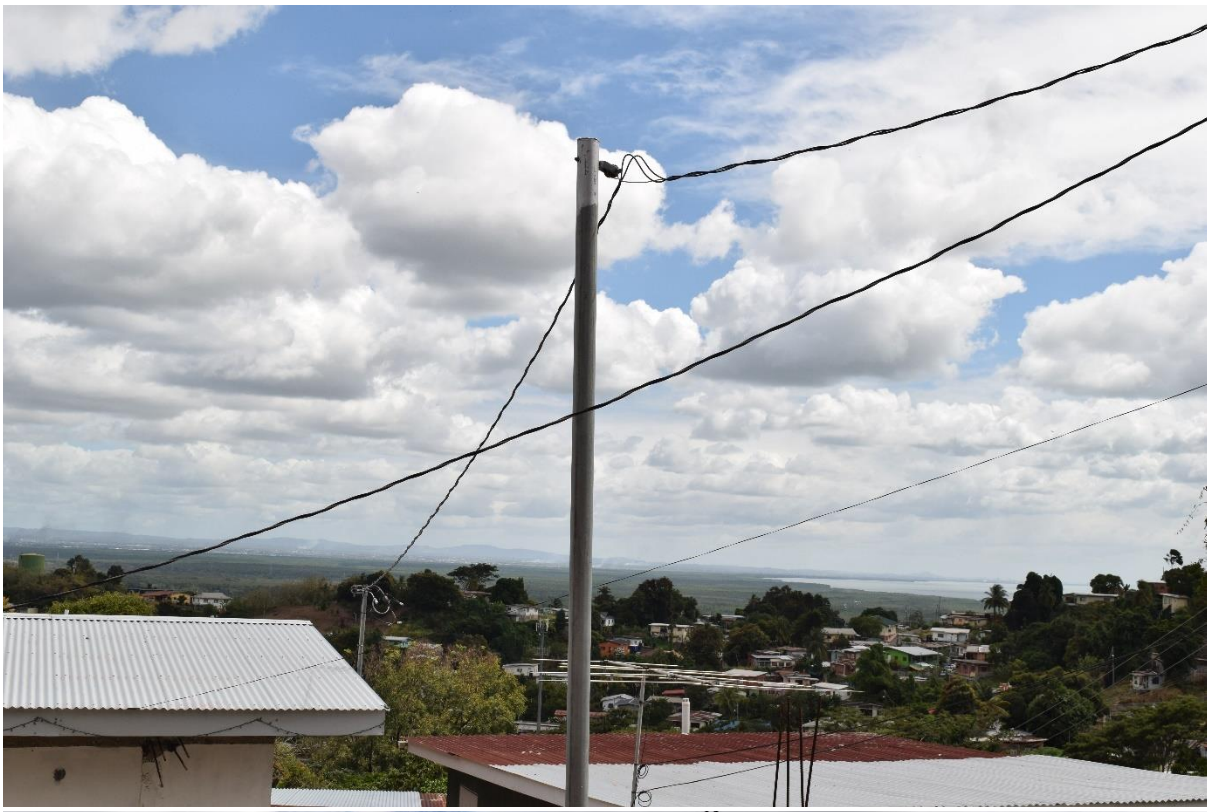

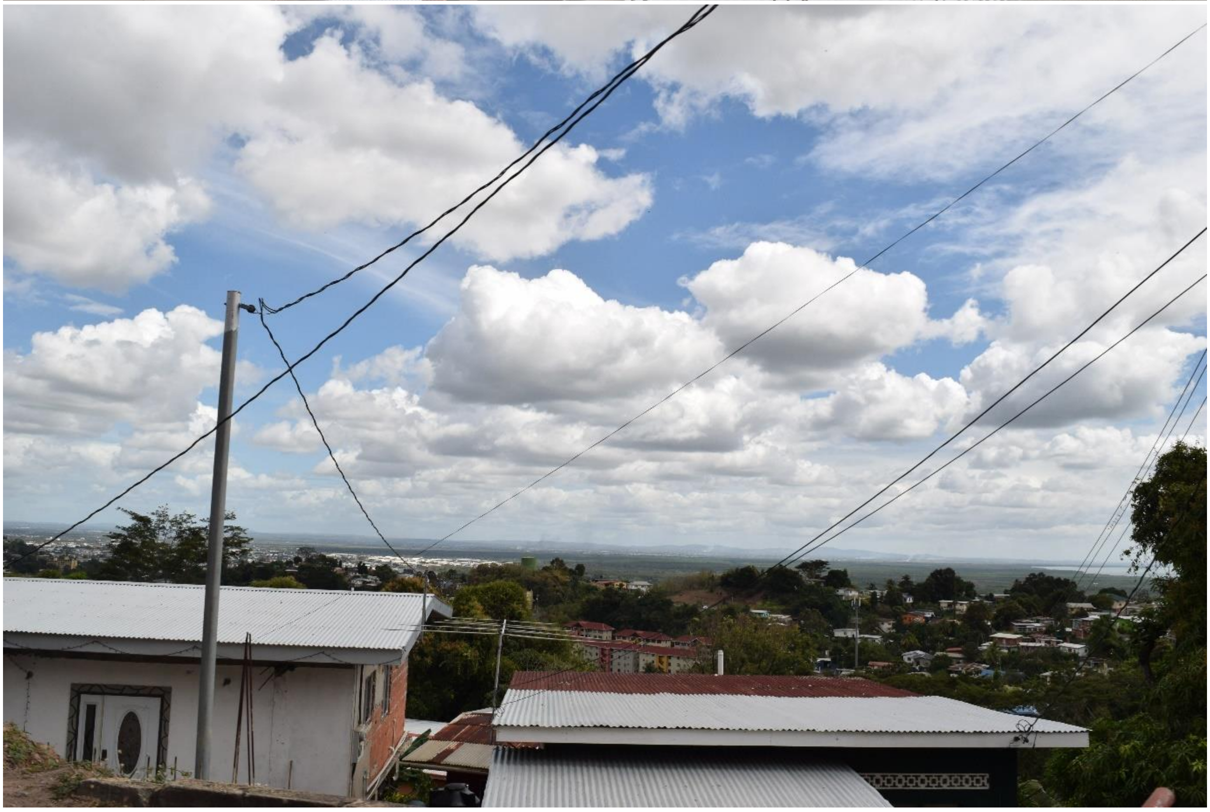

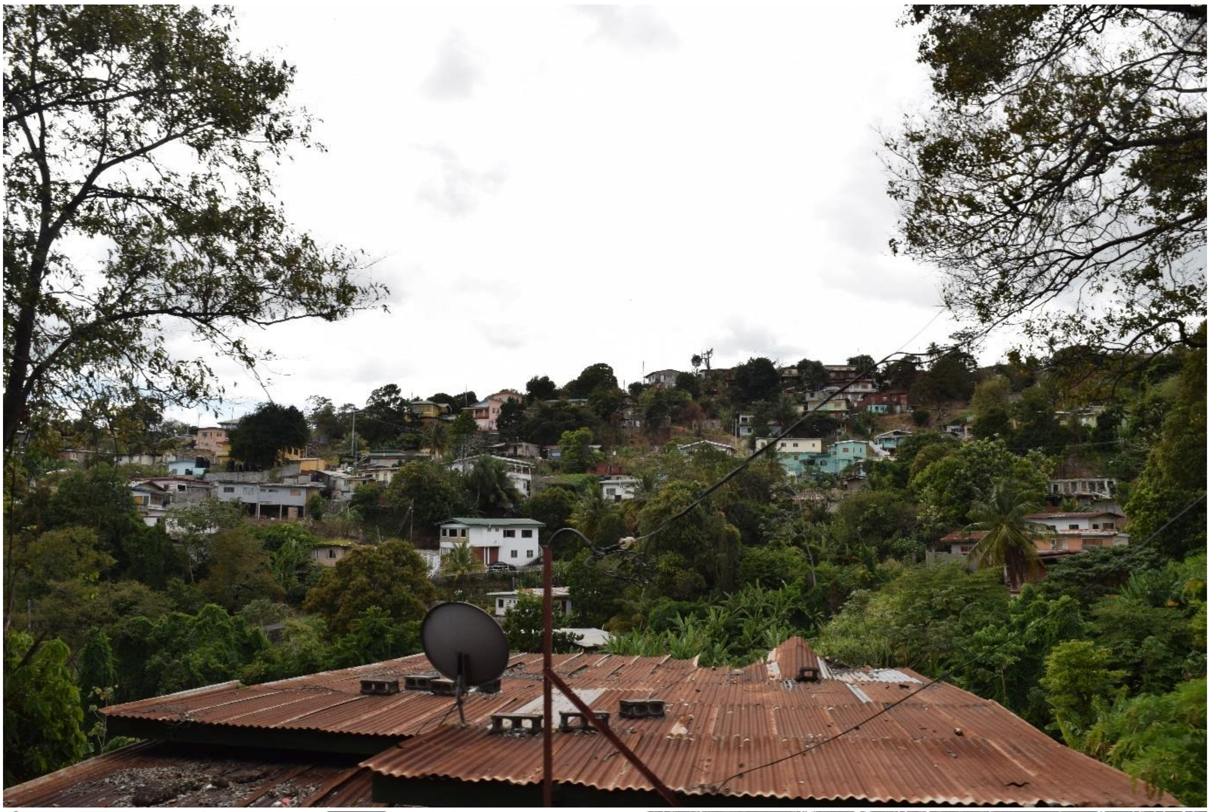

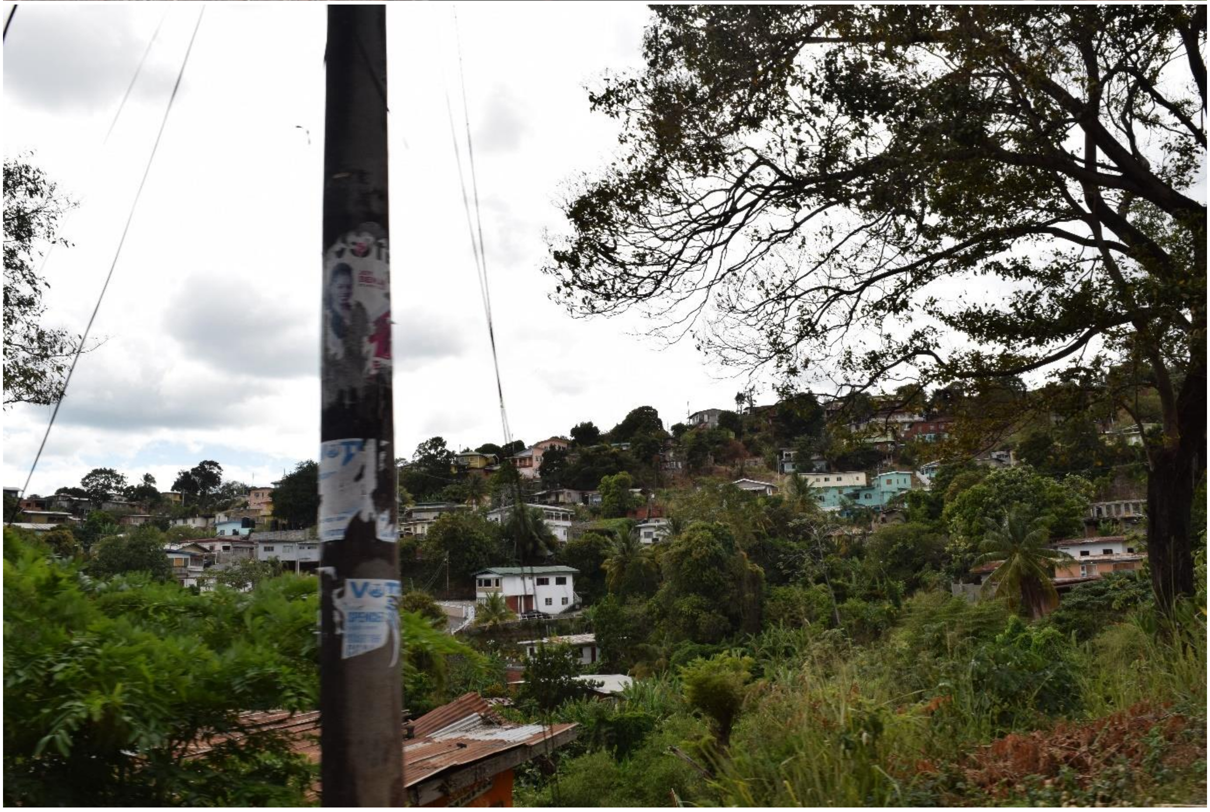

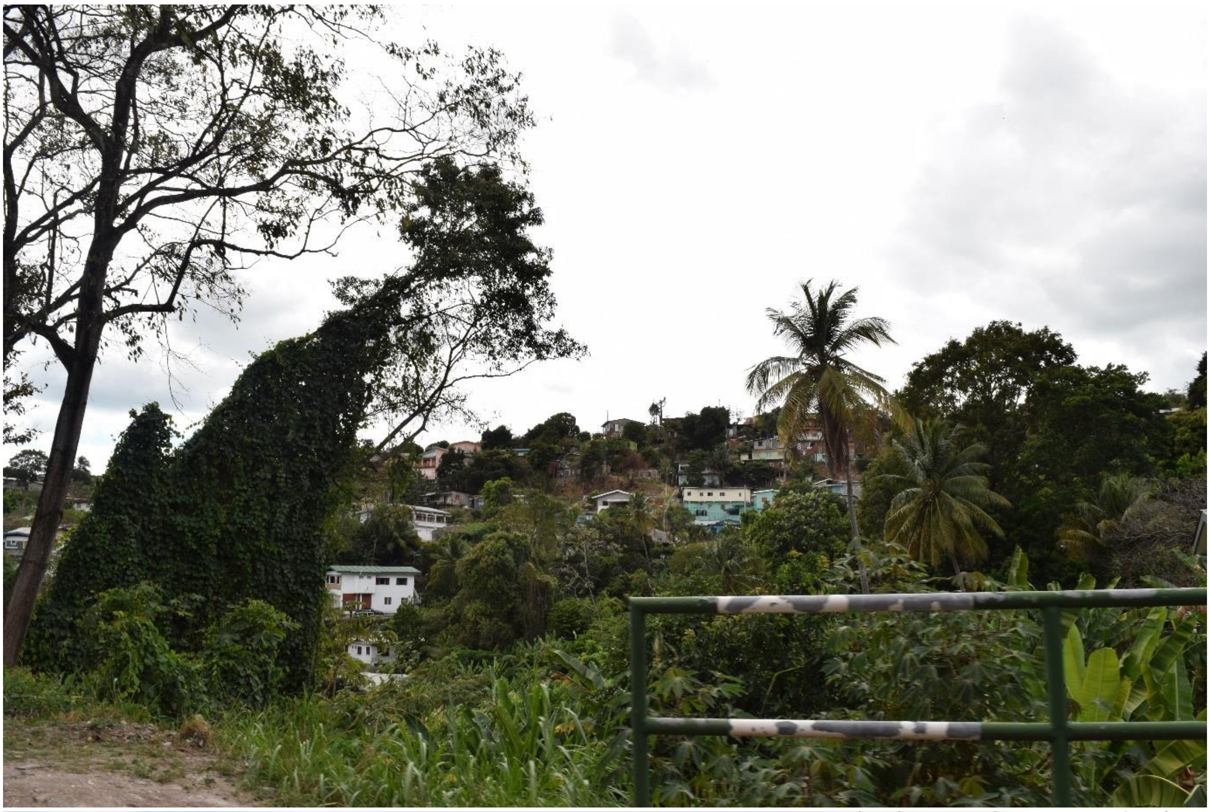

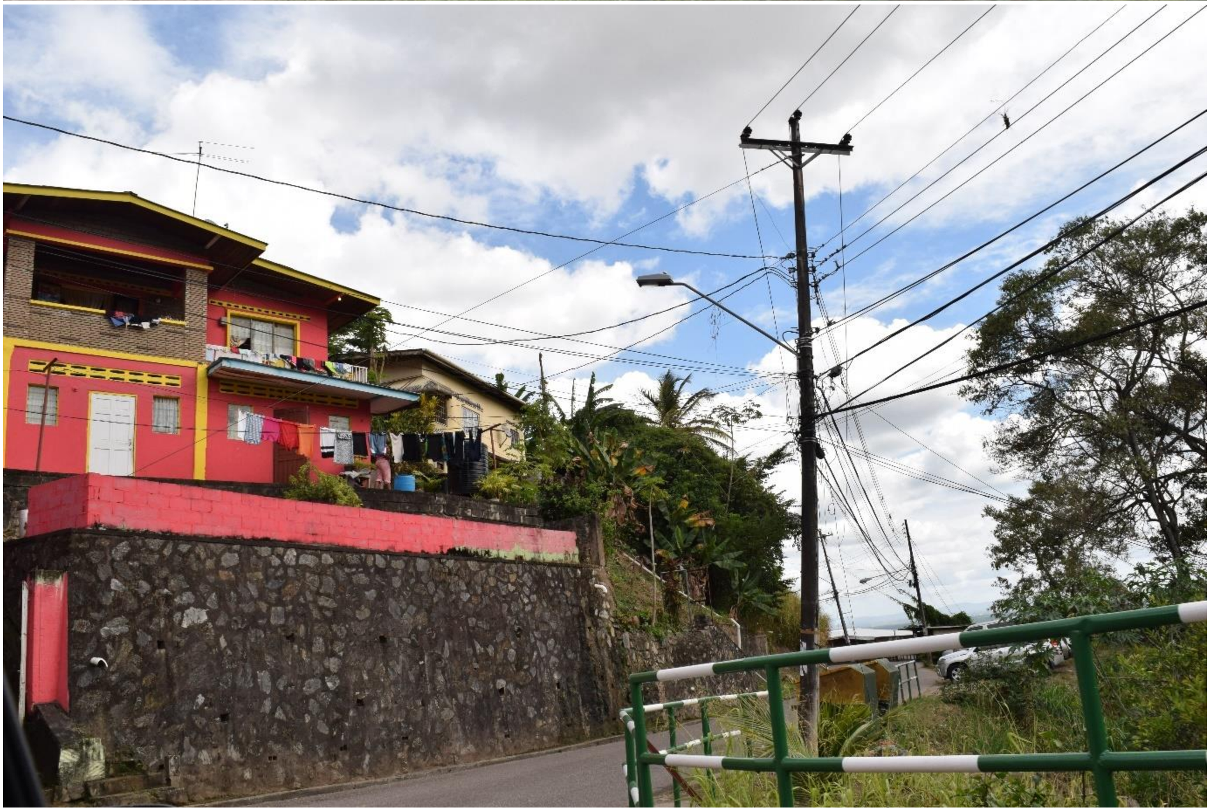

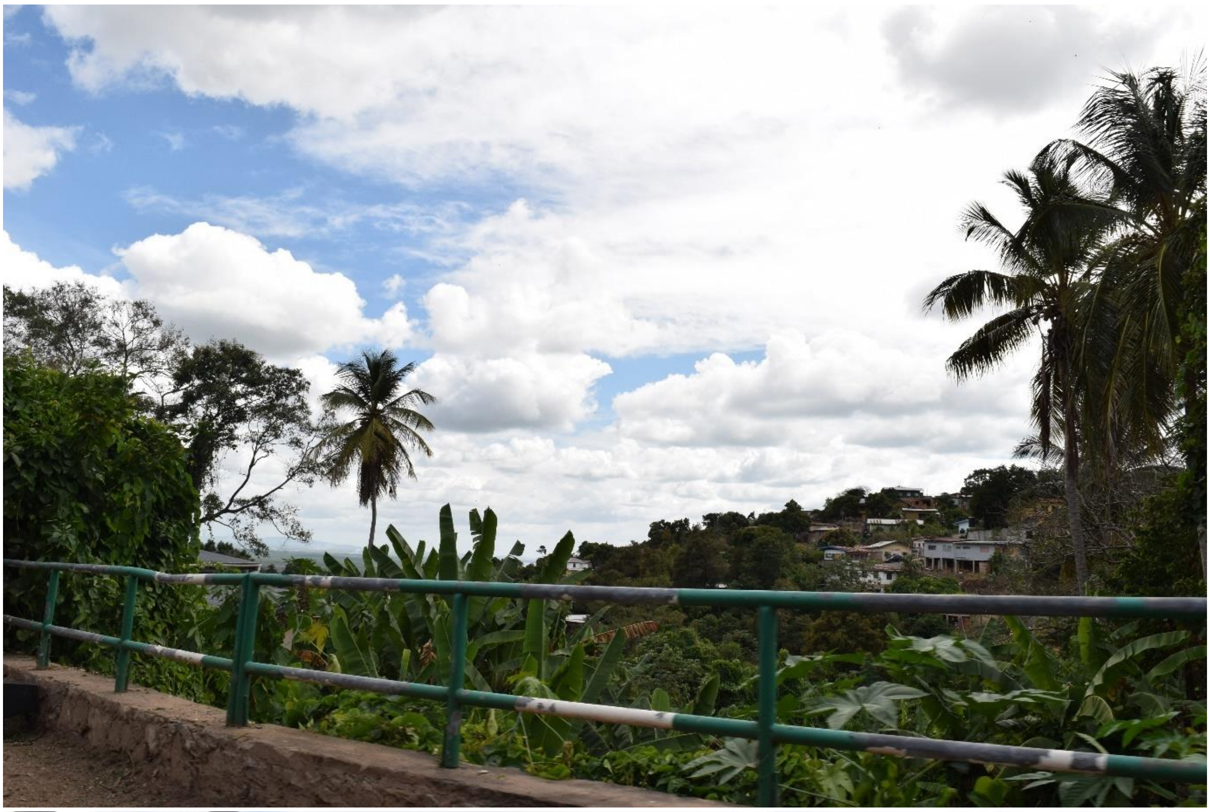

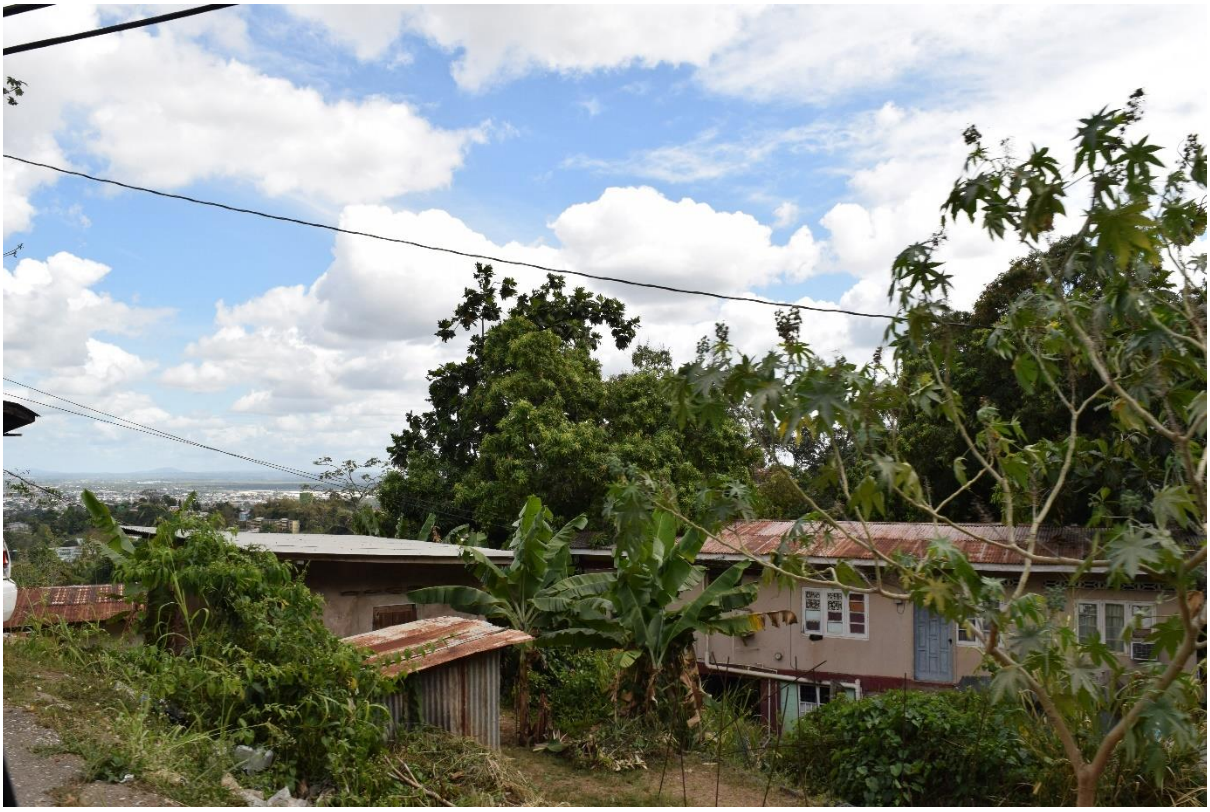

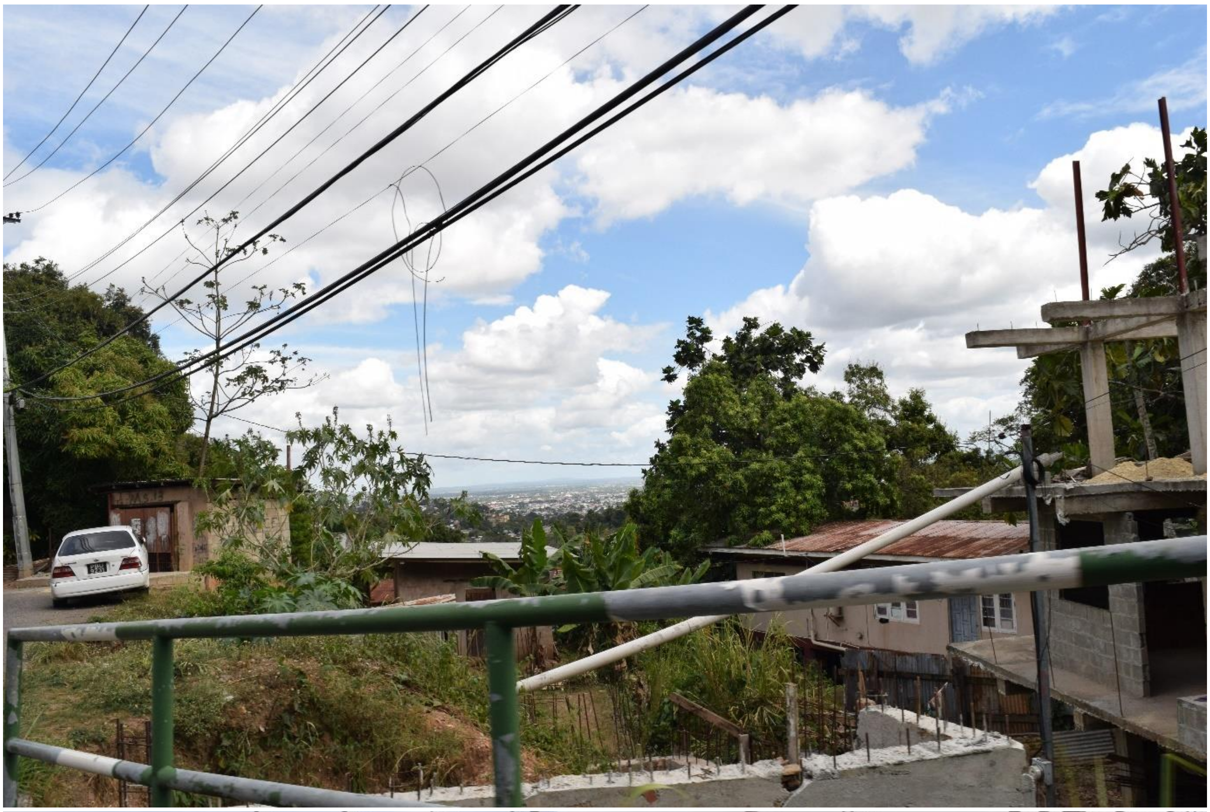

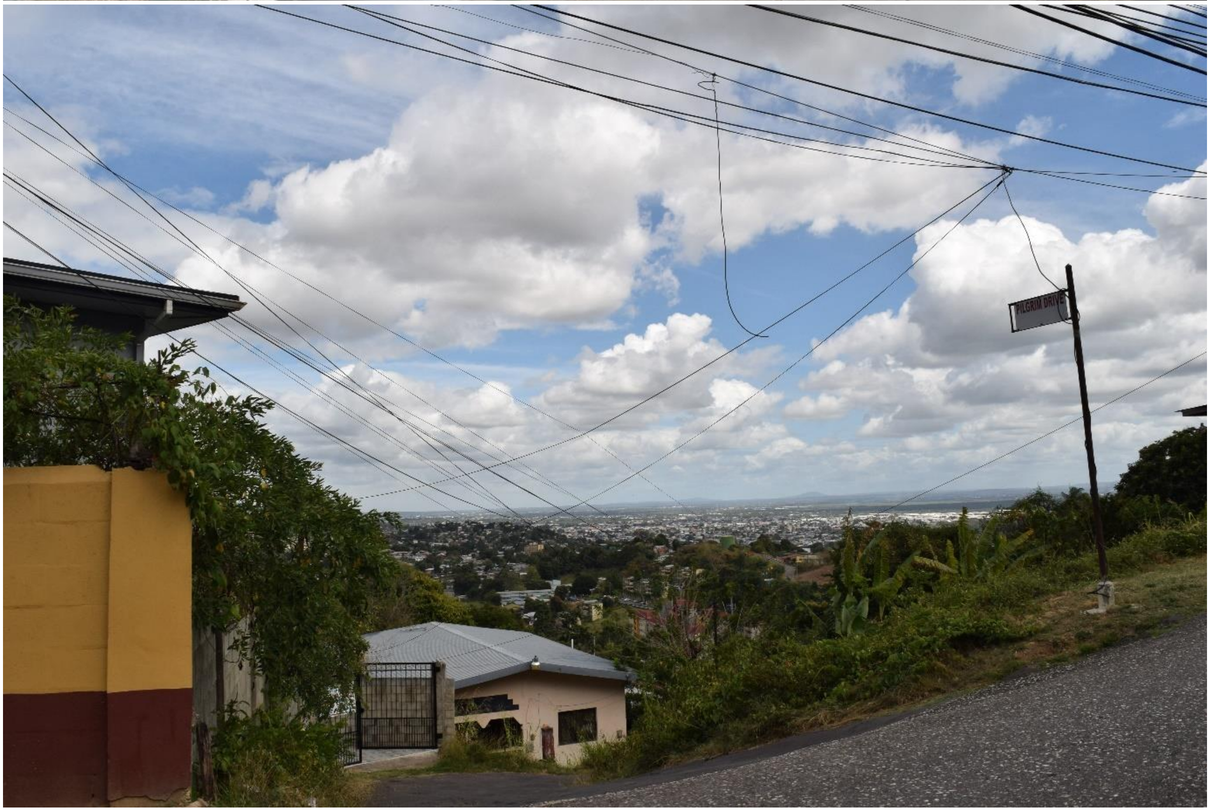
PILGRIM DRIVE

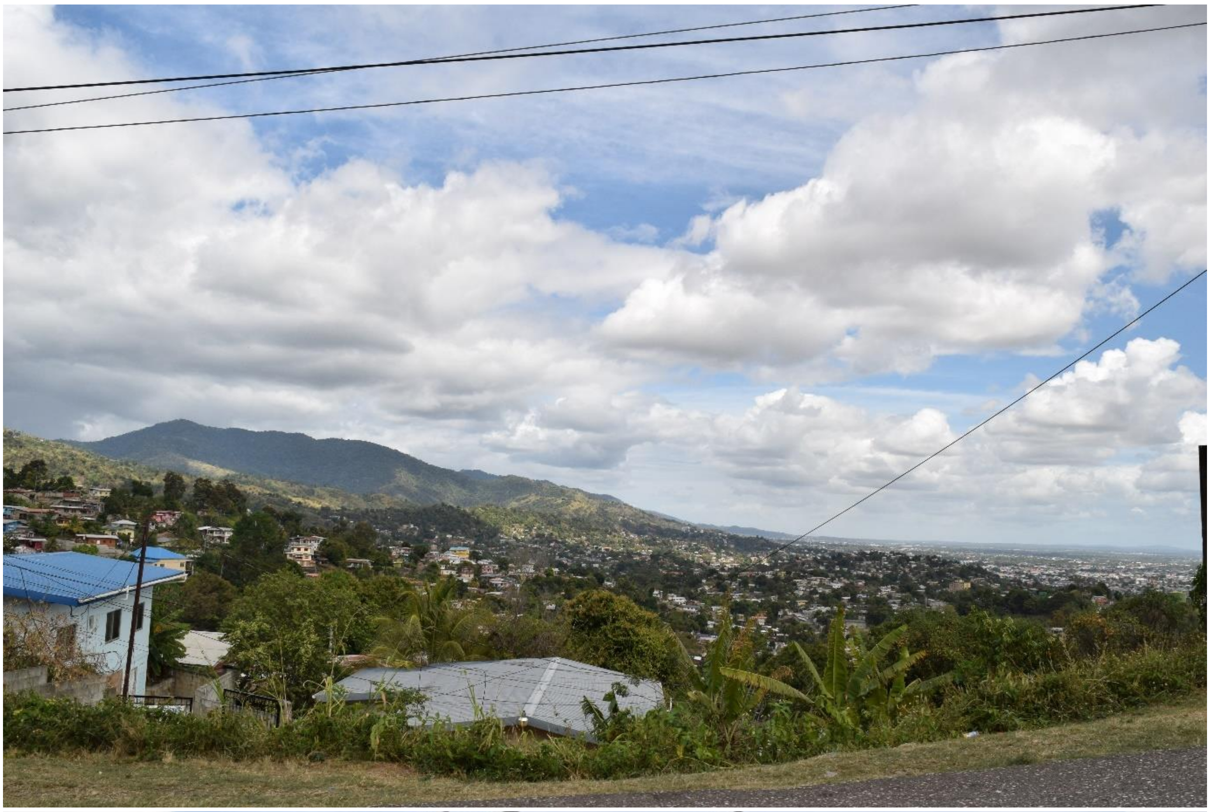

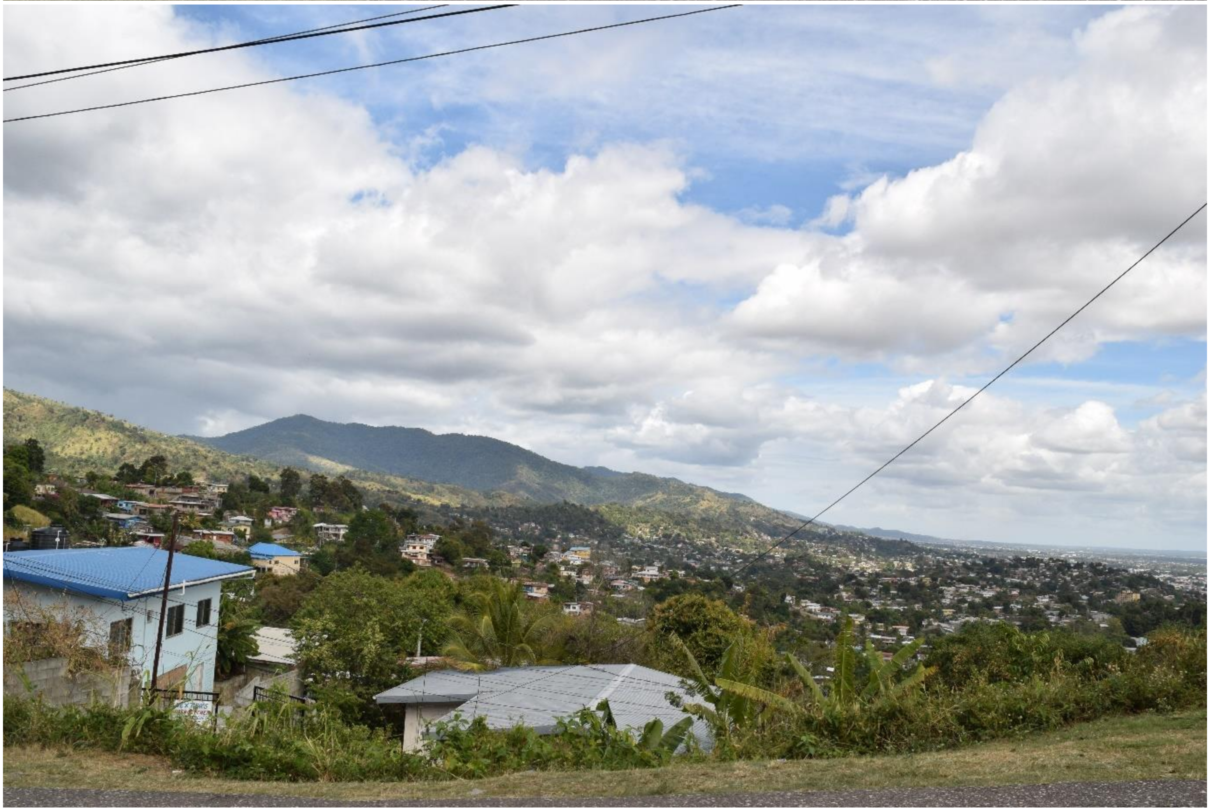

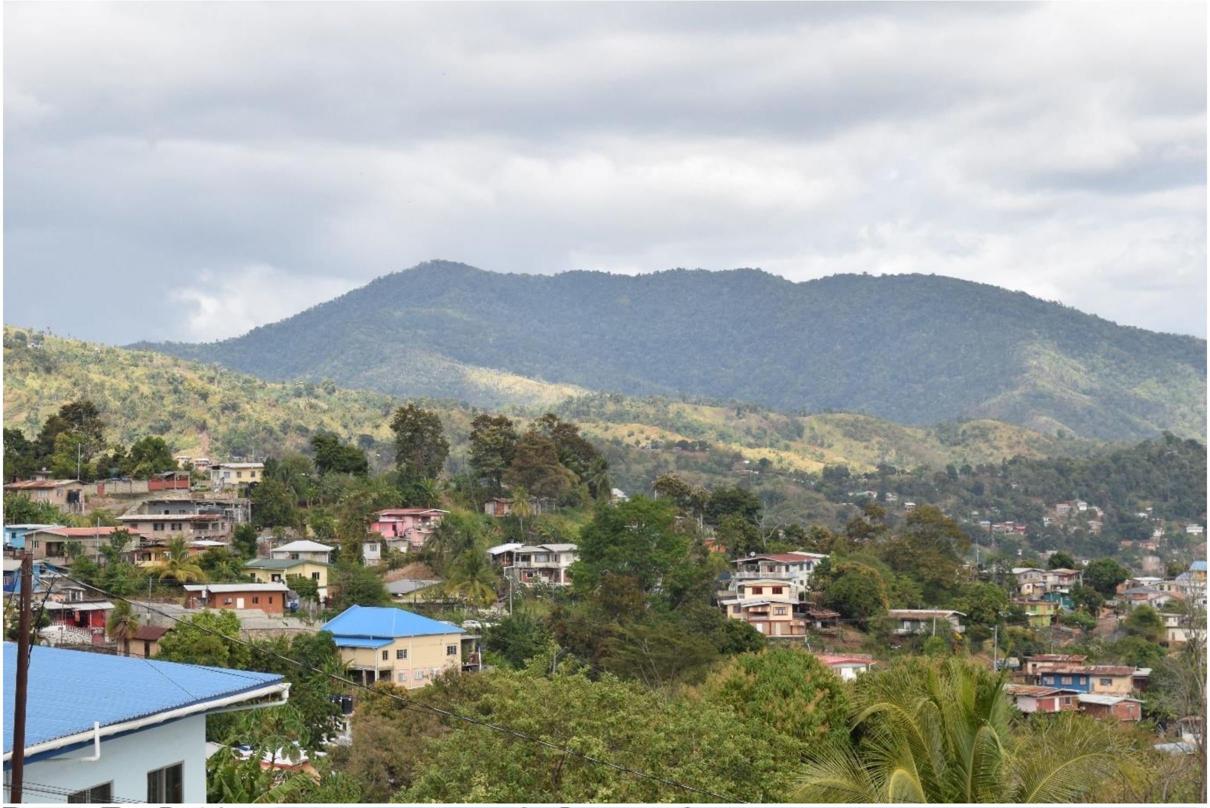

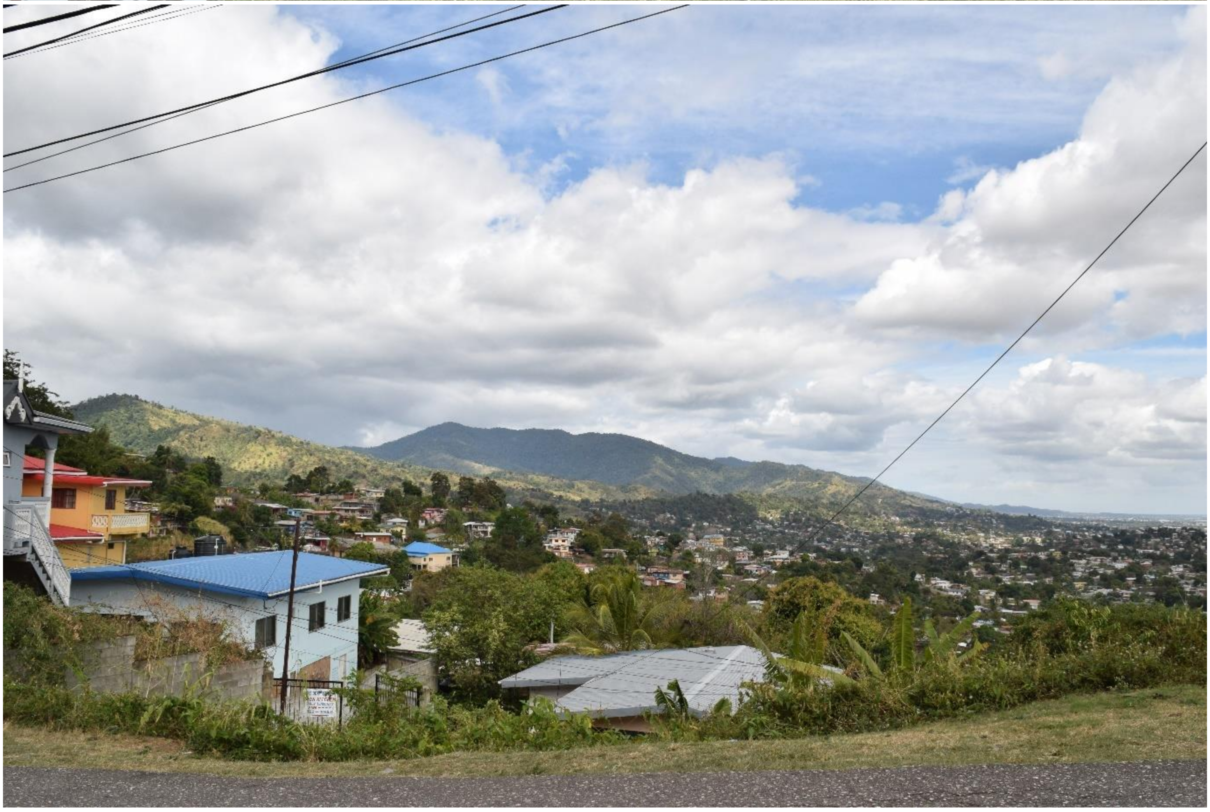

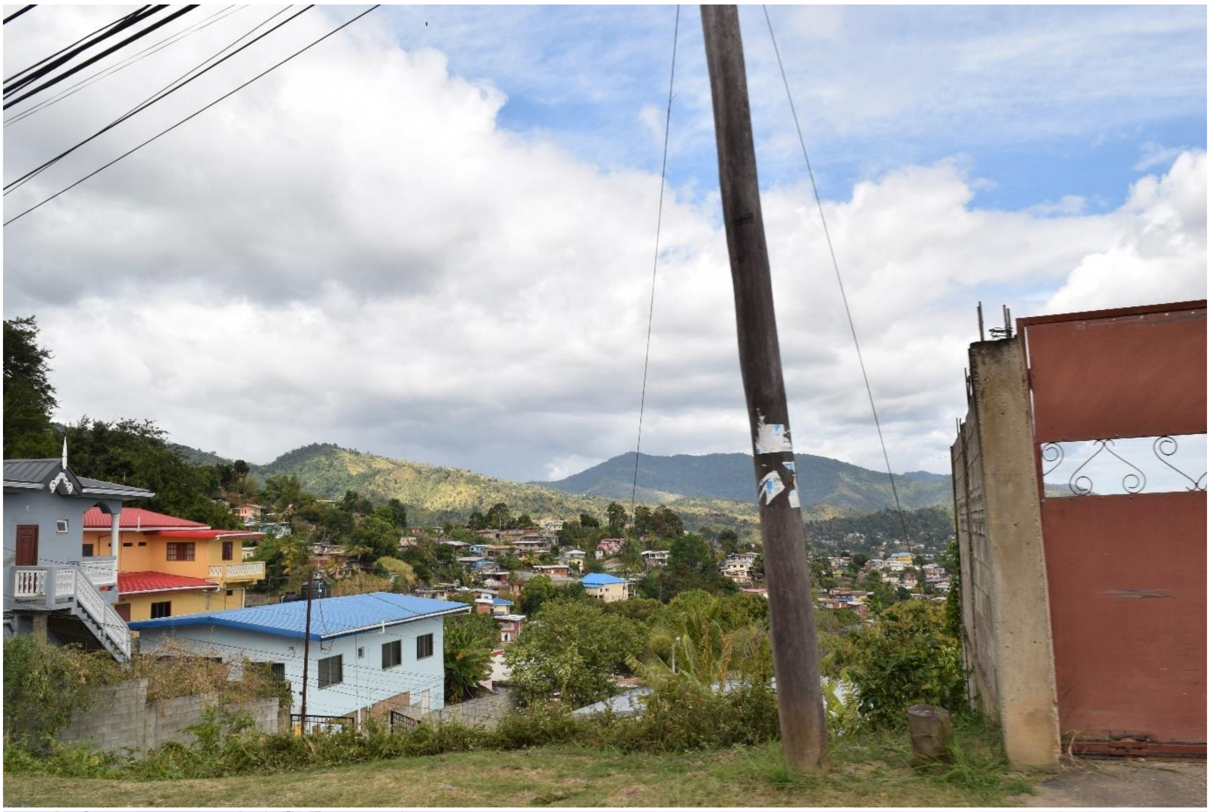

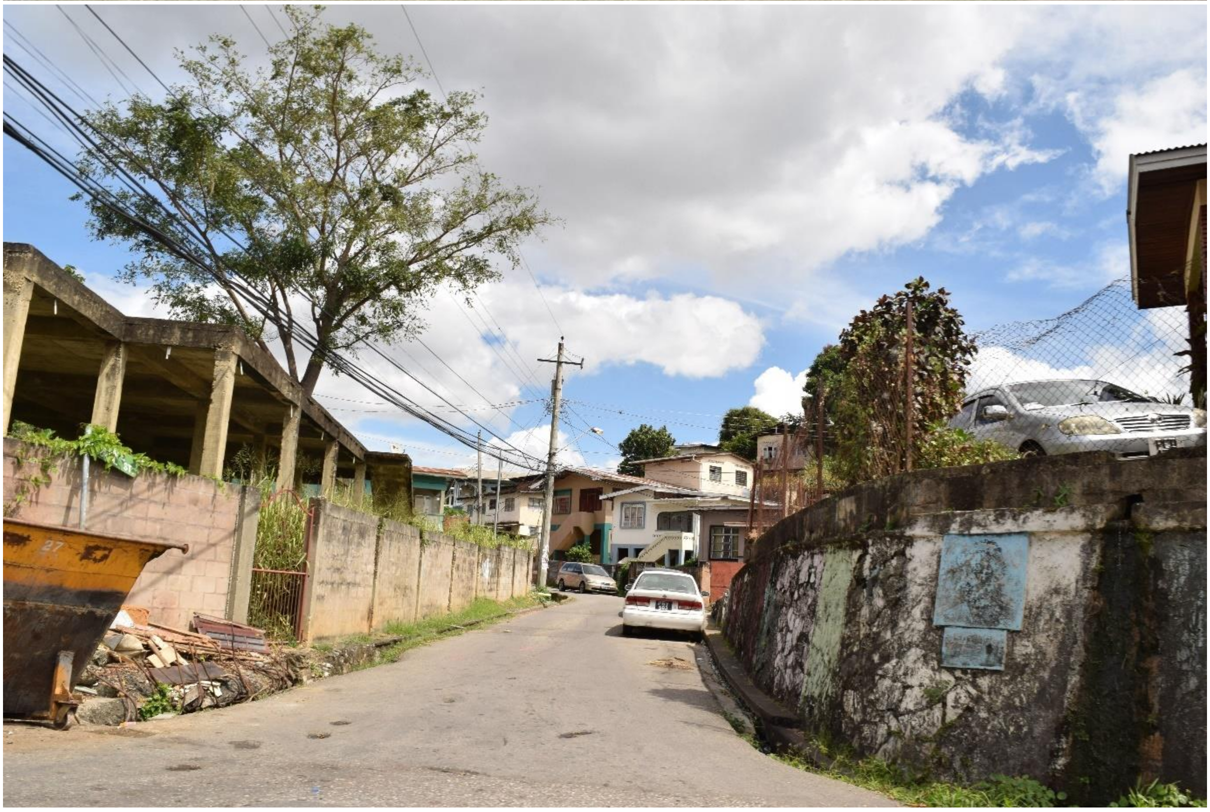

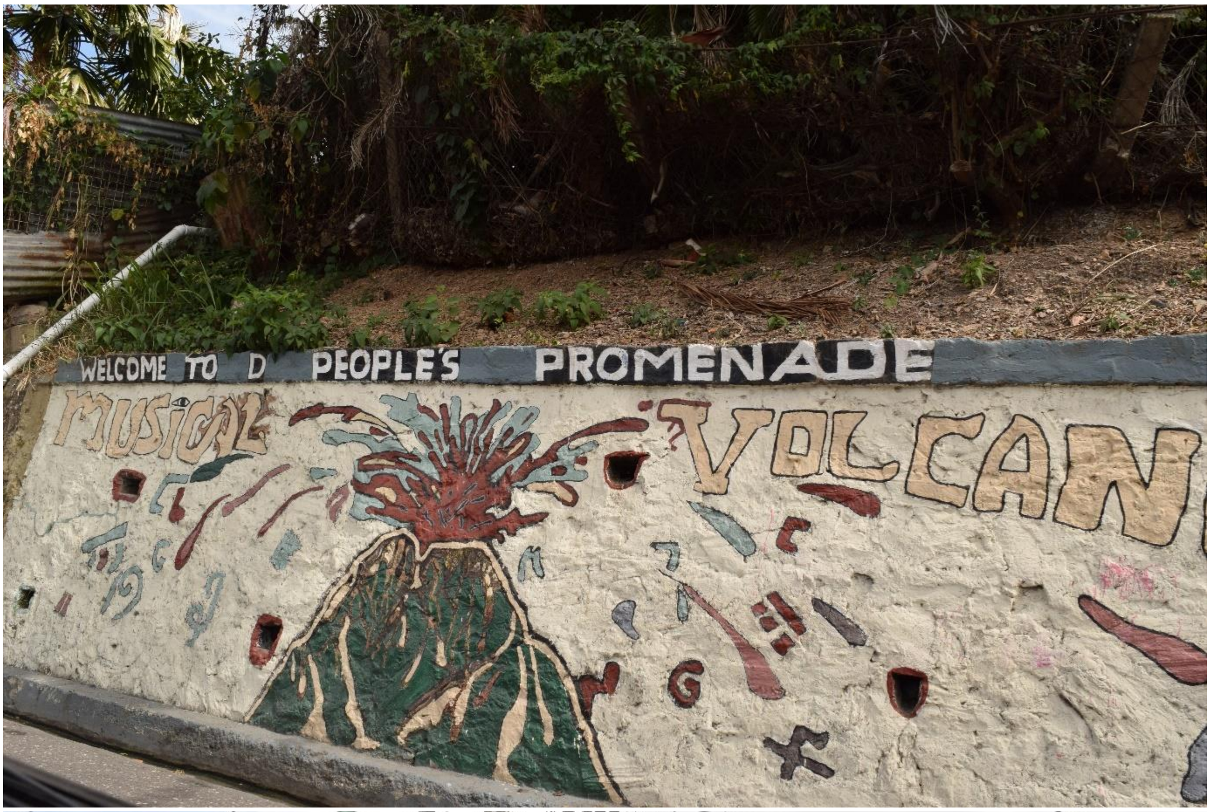
WELCOME TO D PEOPLE'S PROMENADE

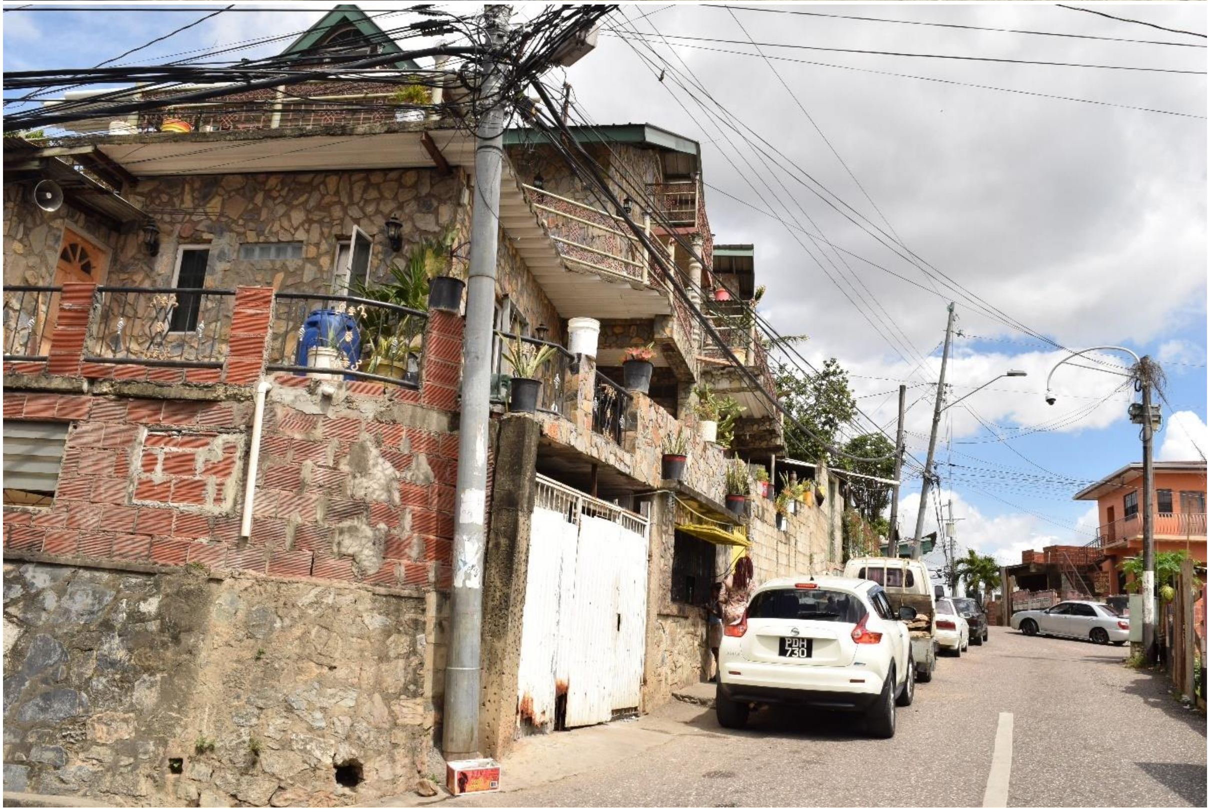

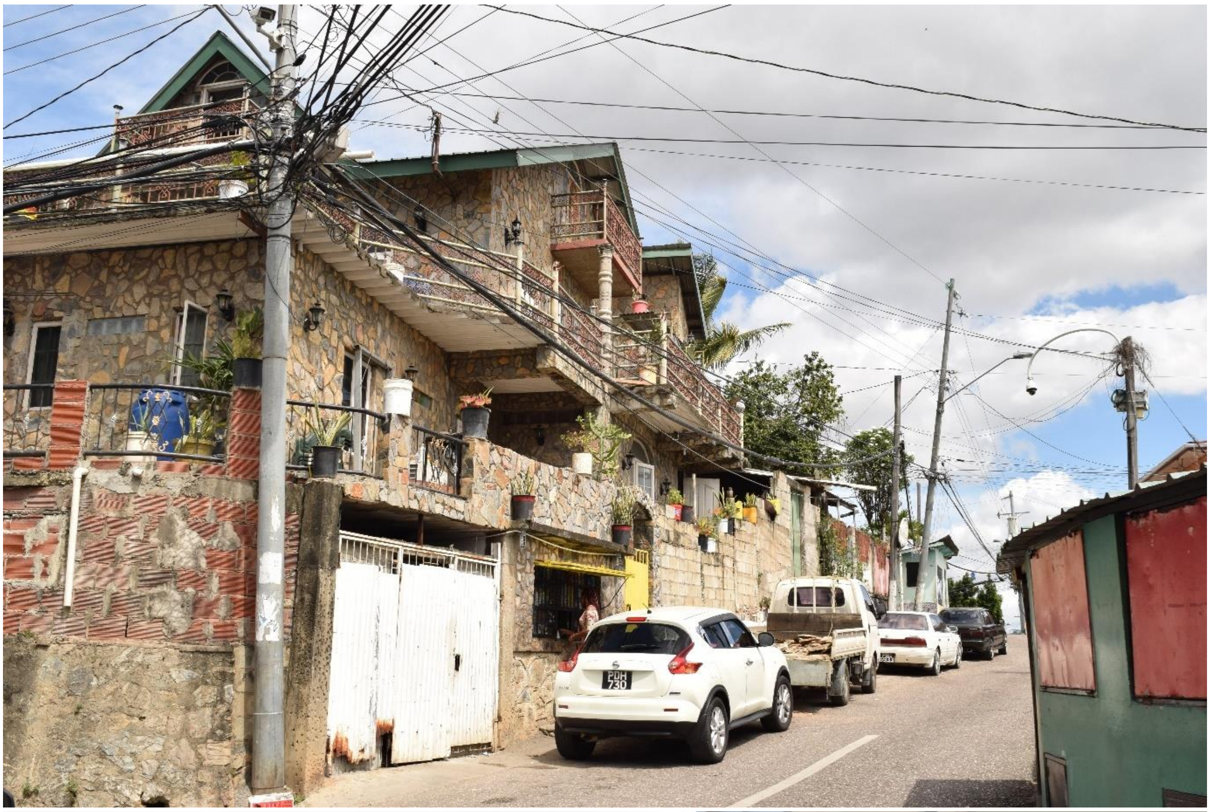

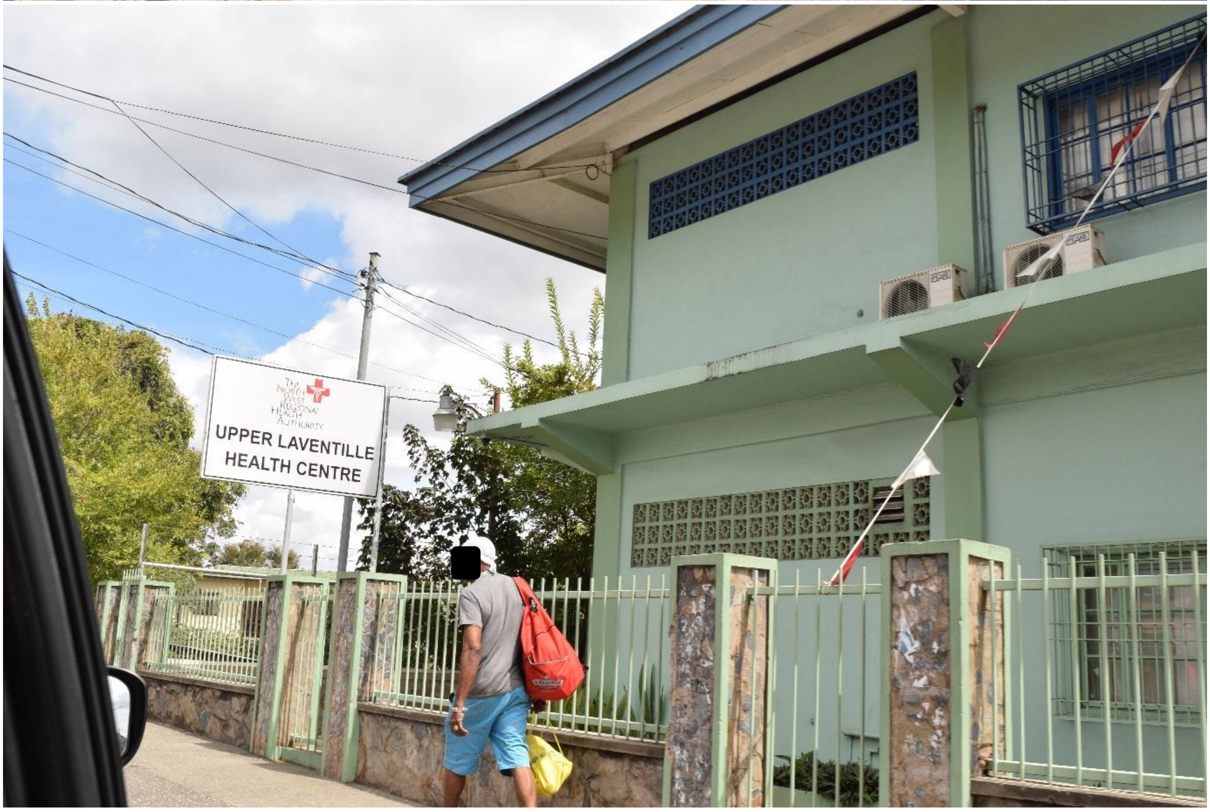
UPPER LAVENTILLE
HEALTH CENTRE

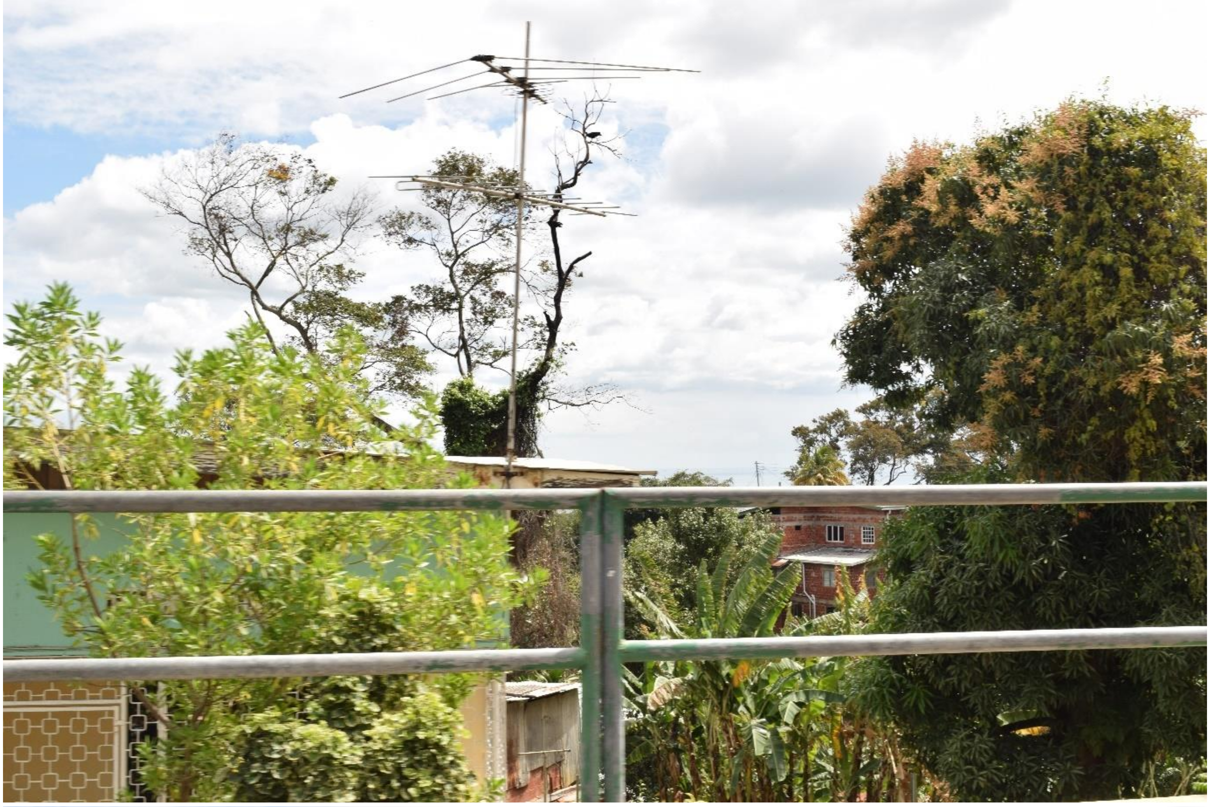

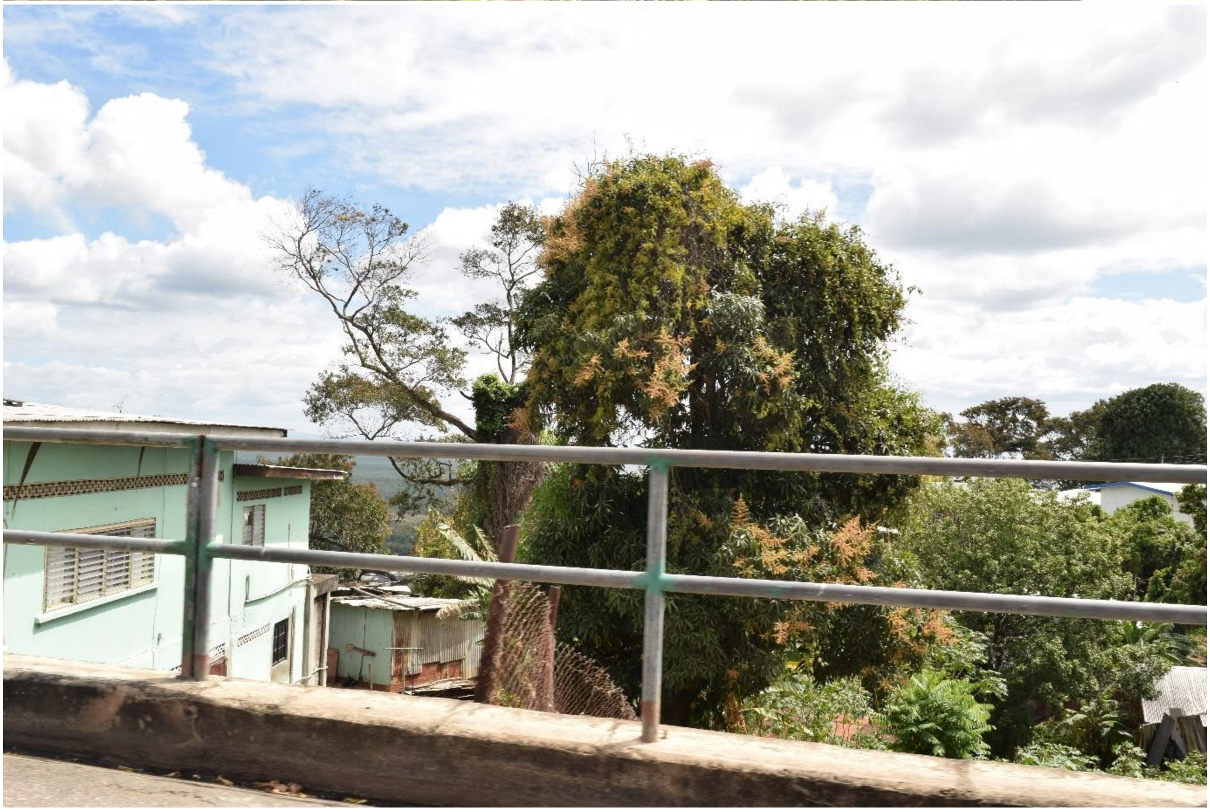

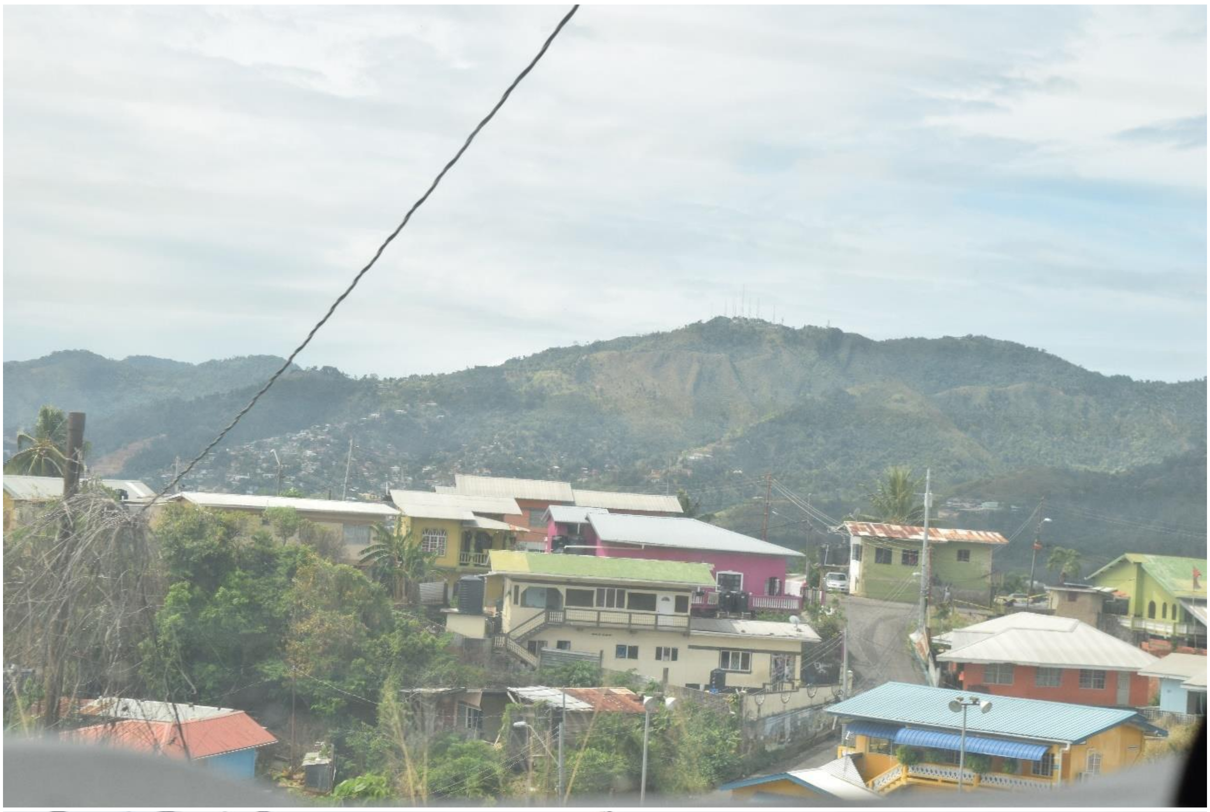

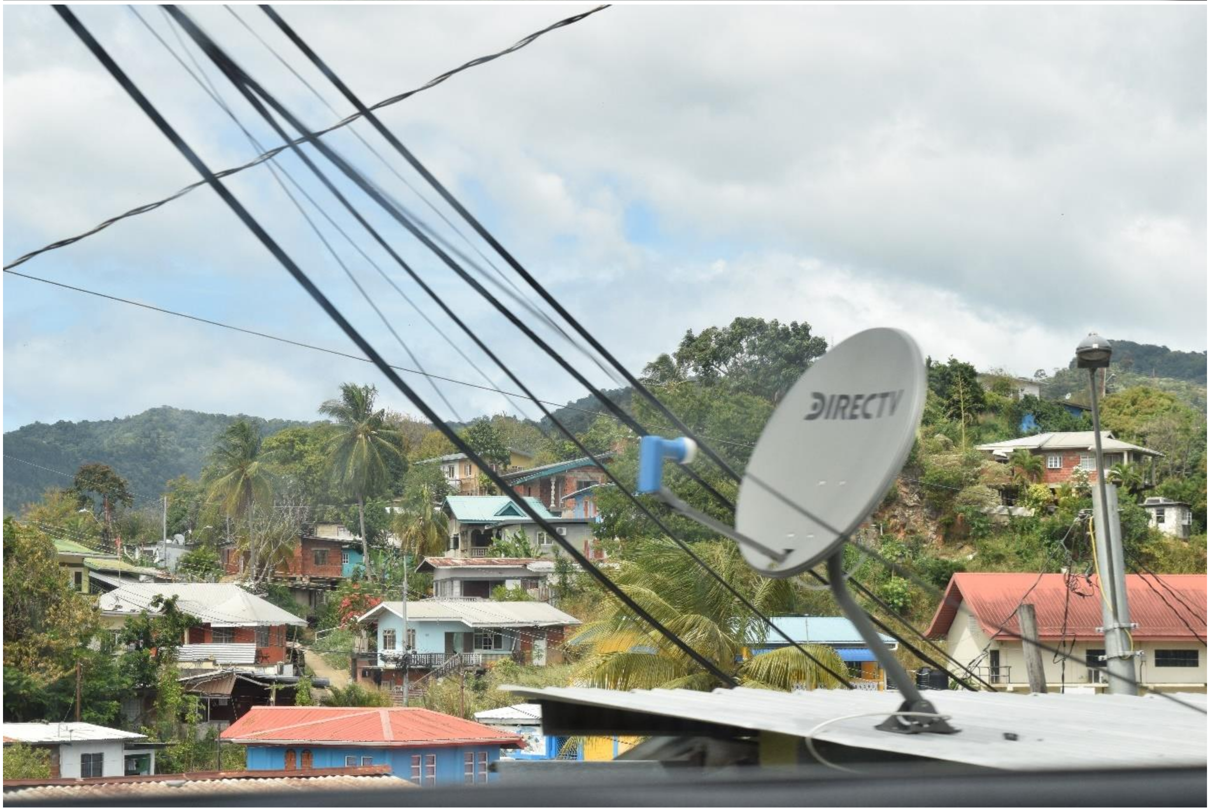
DIRECTV

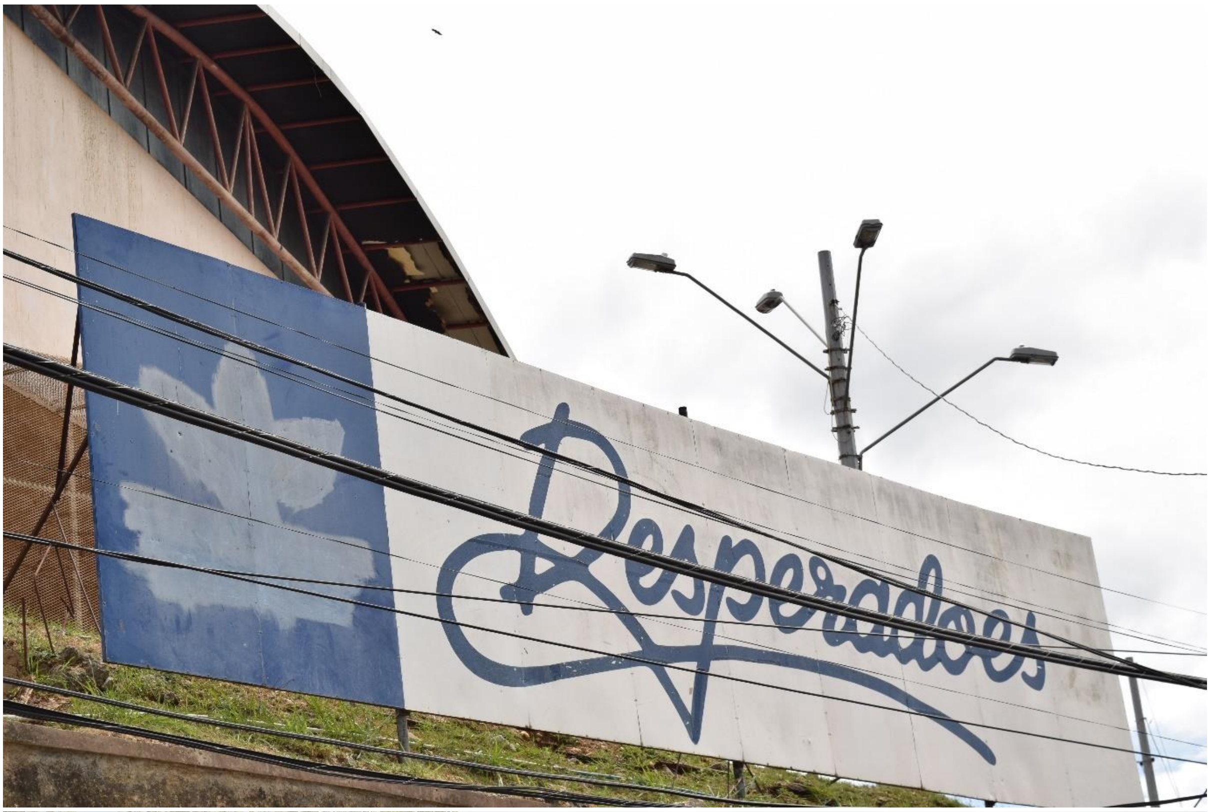
Desperadoes

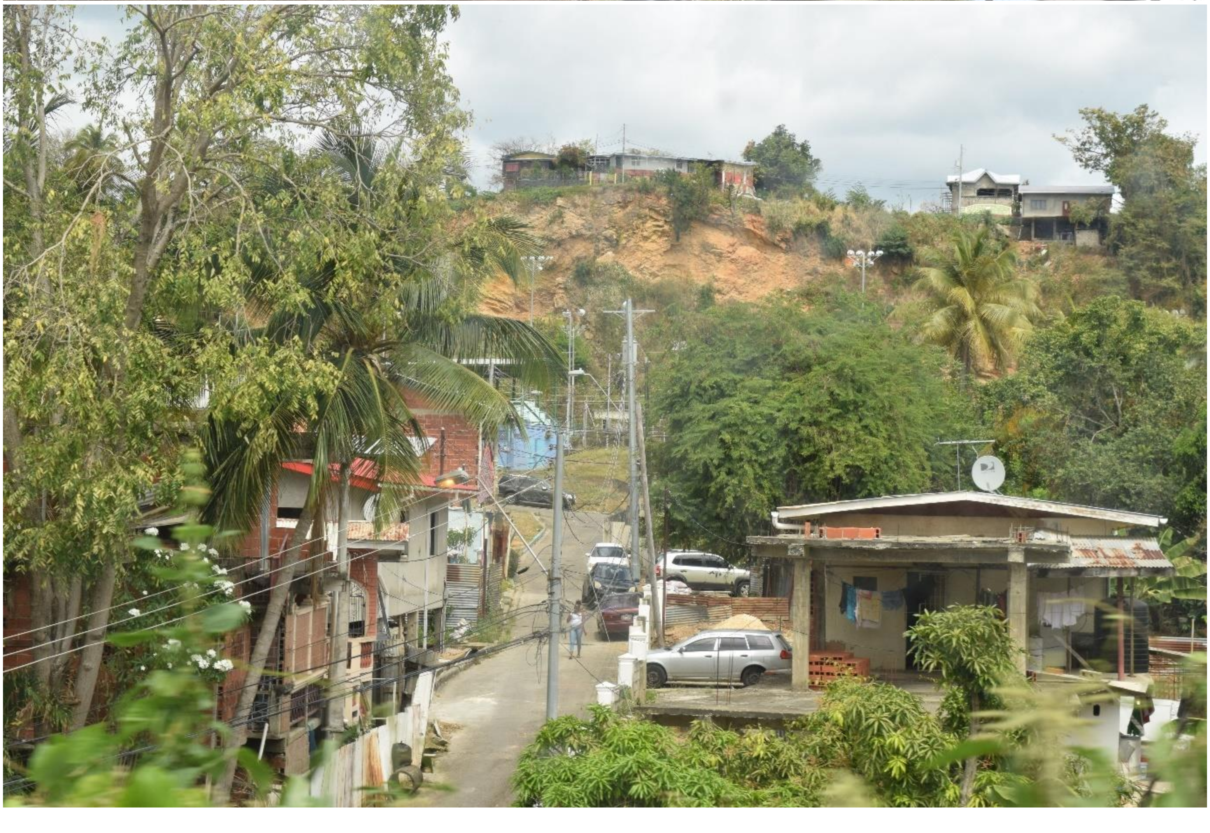

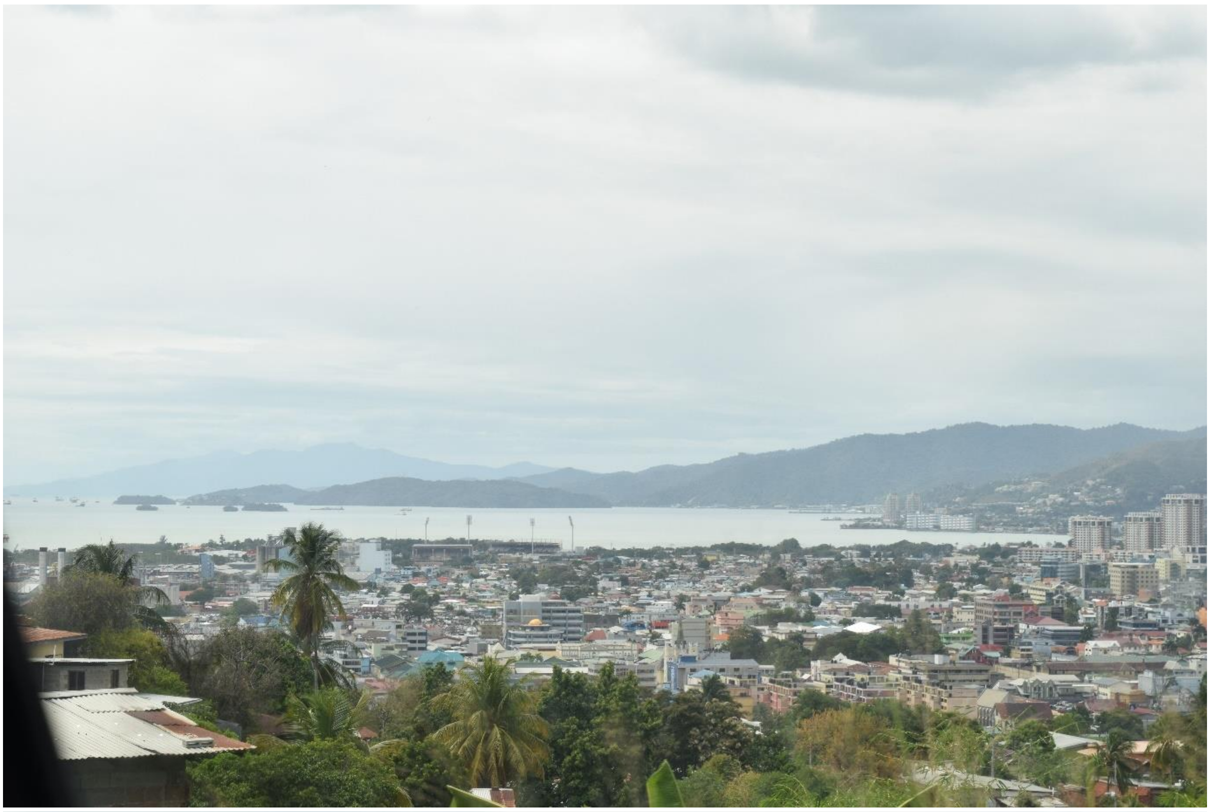
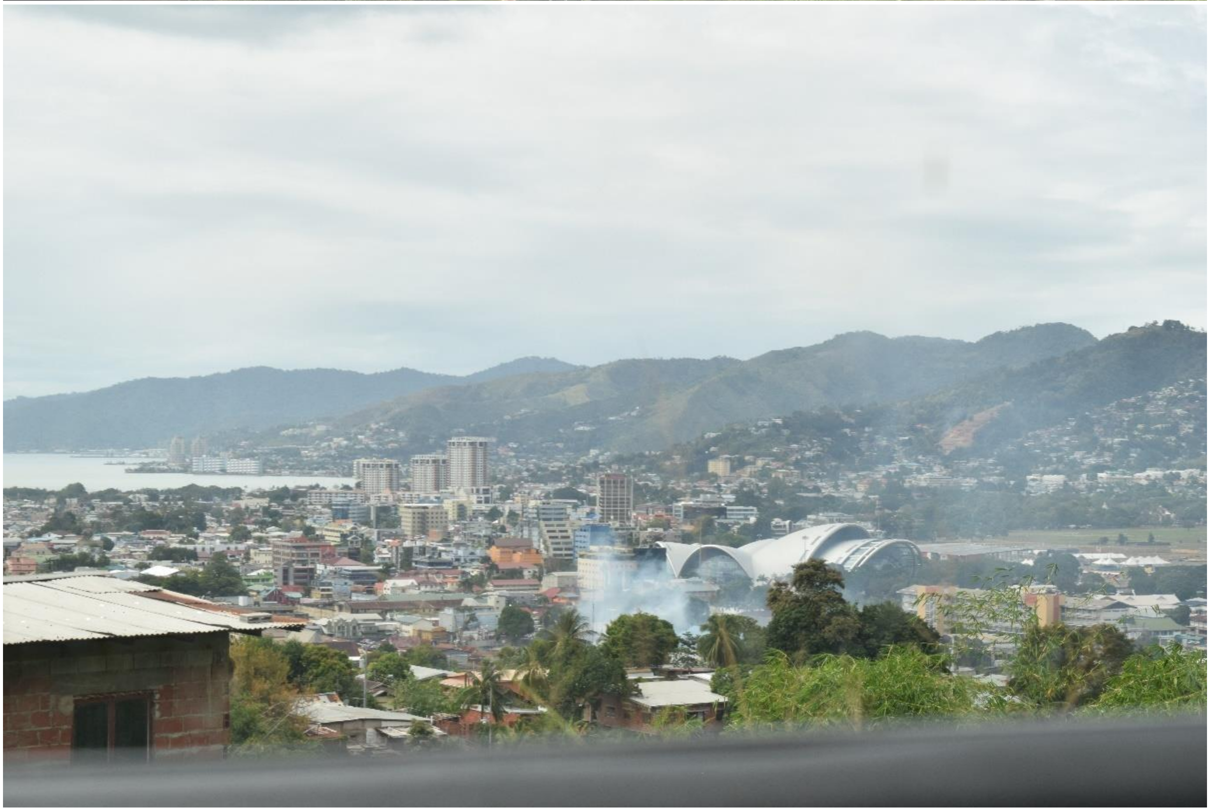

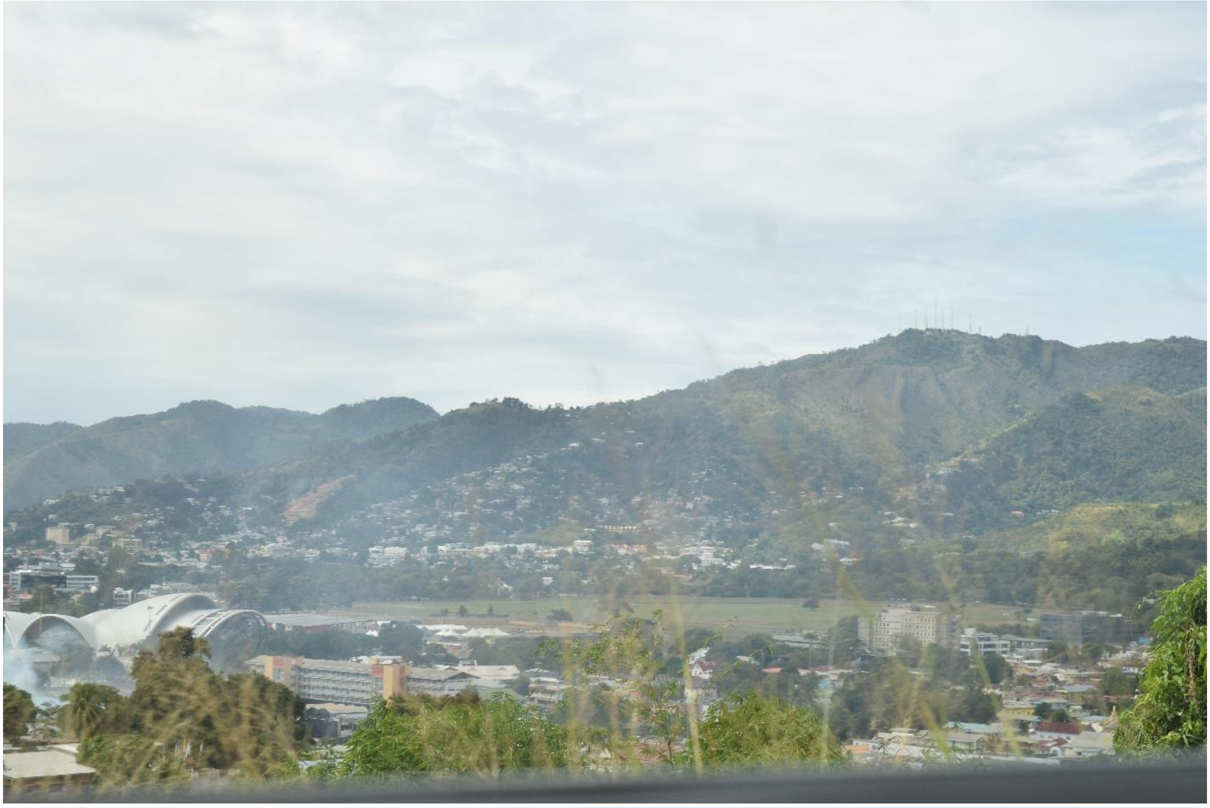

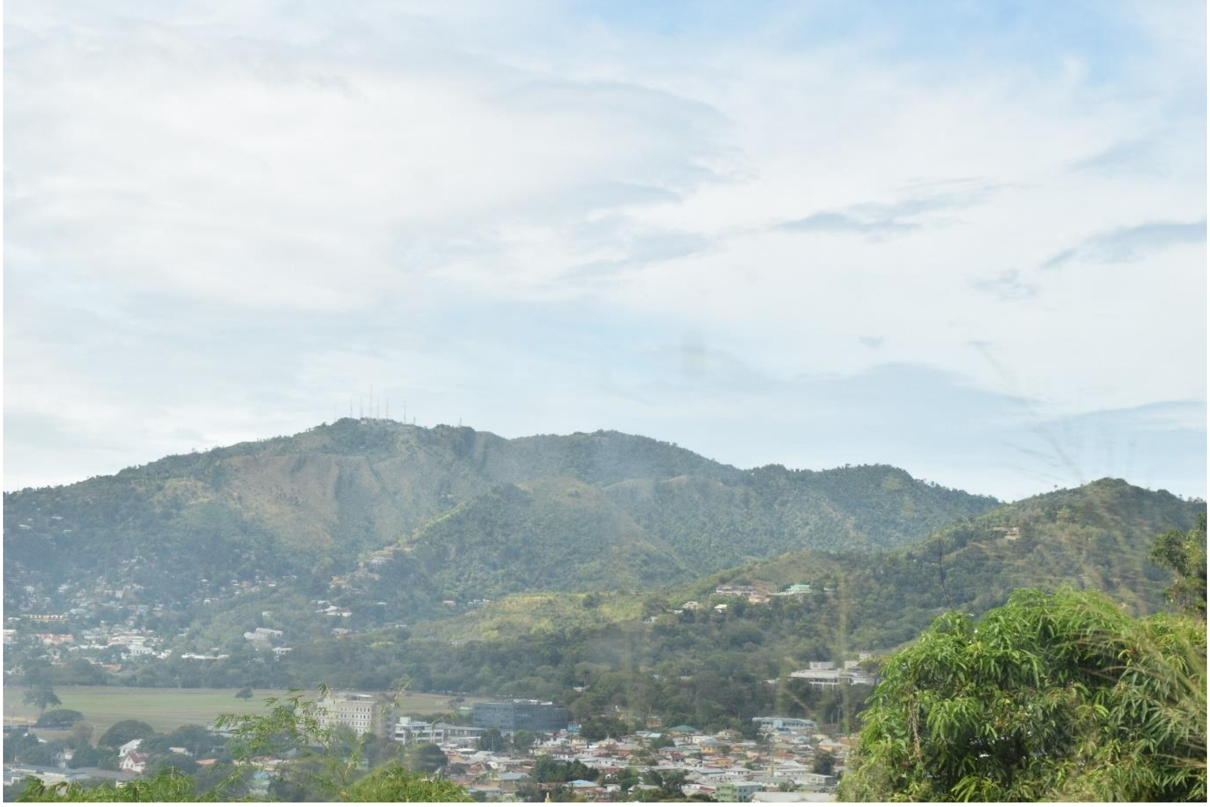

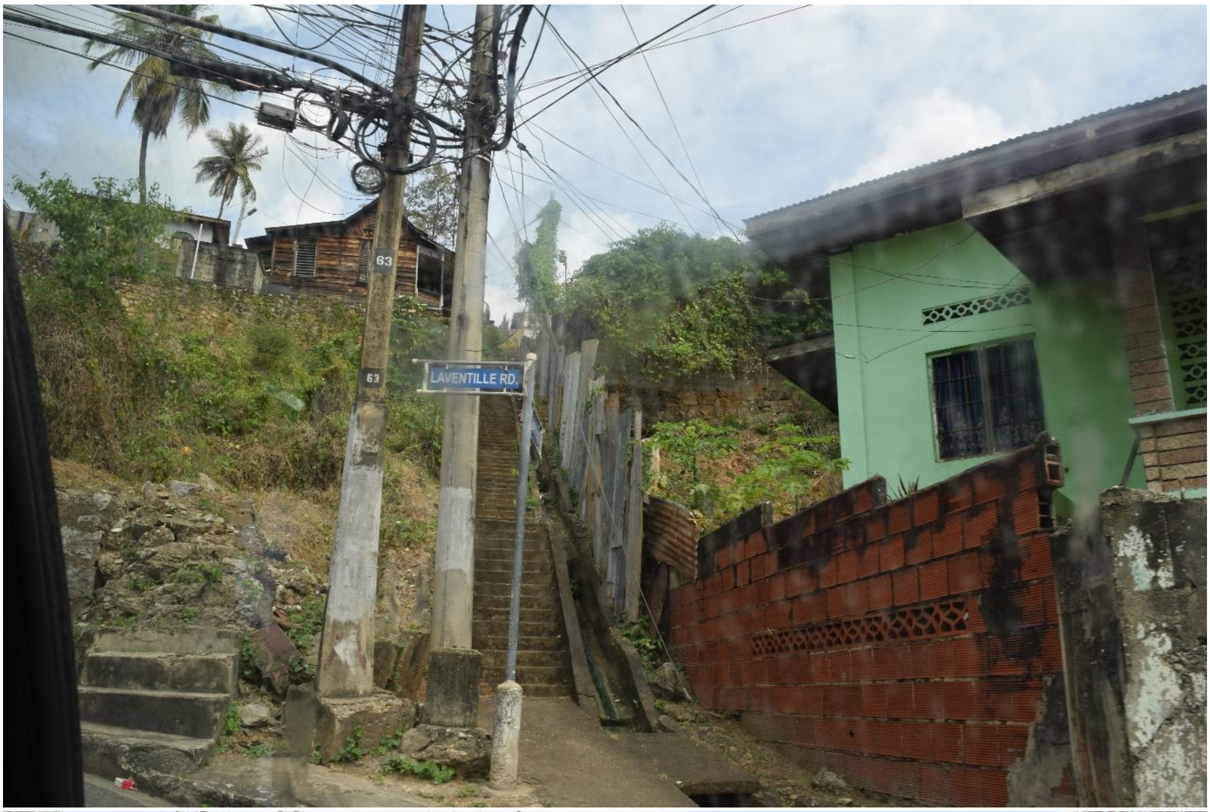
LAVENTILLE RD.
63

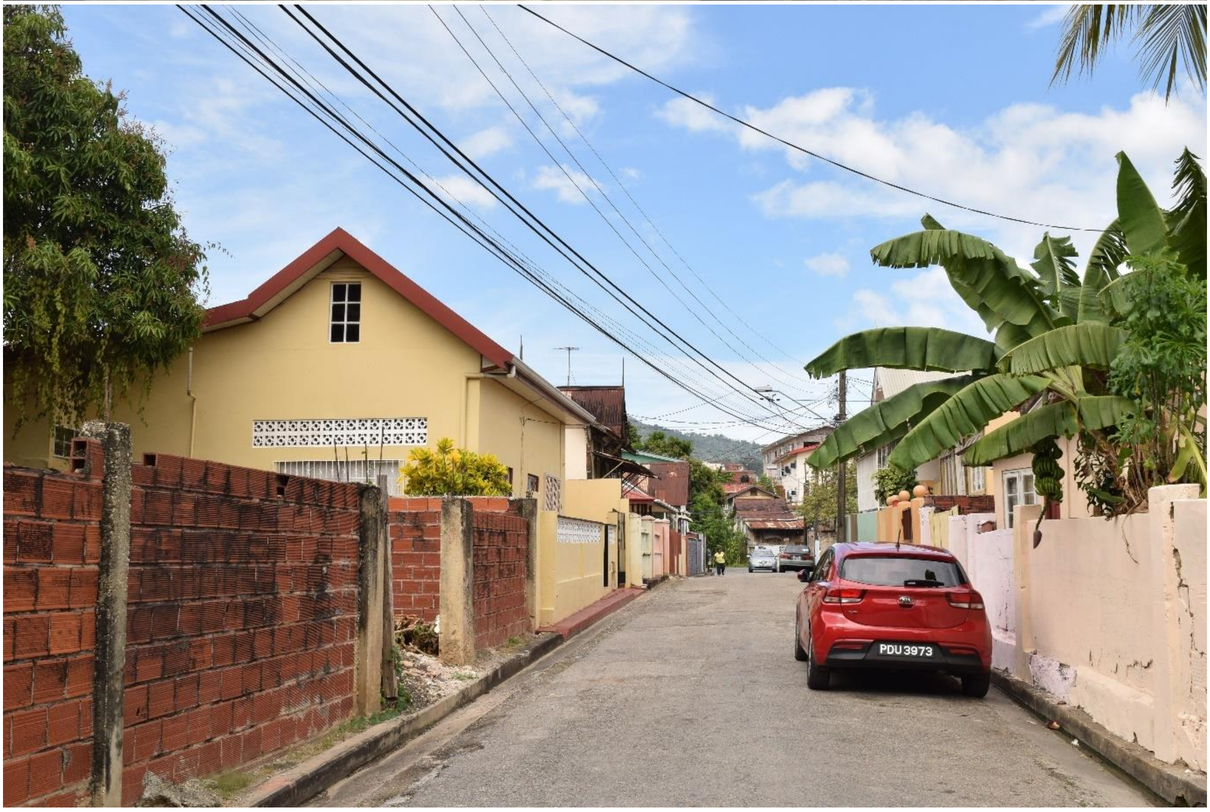
PDU 3973

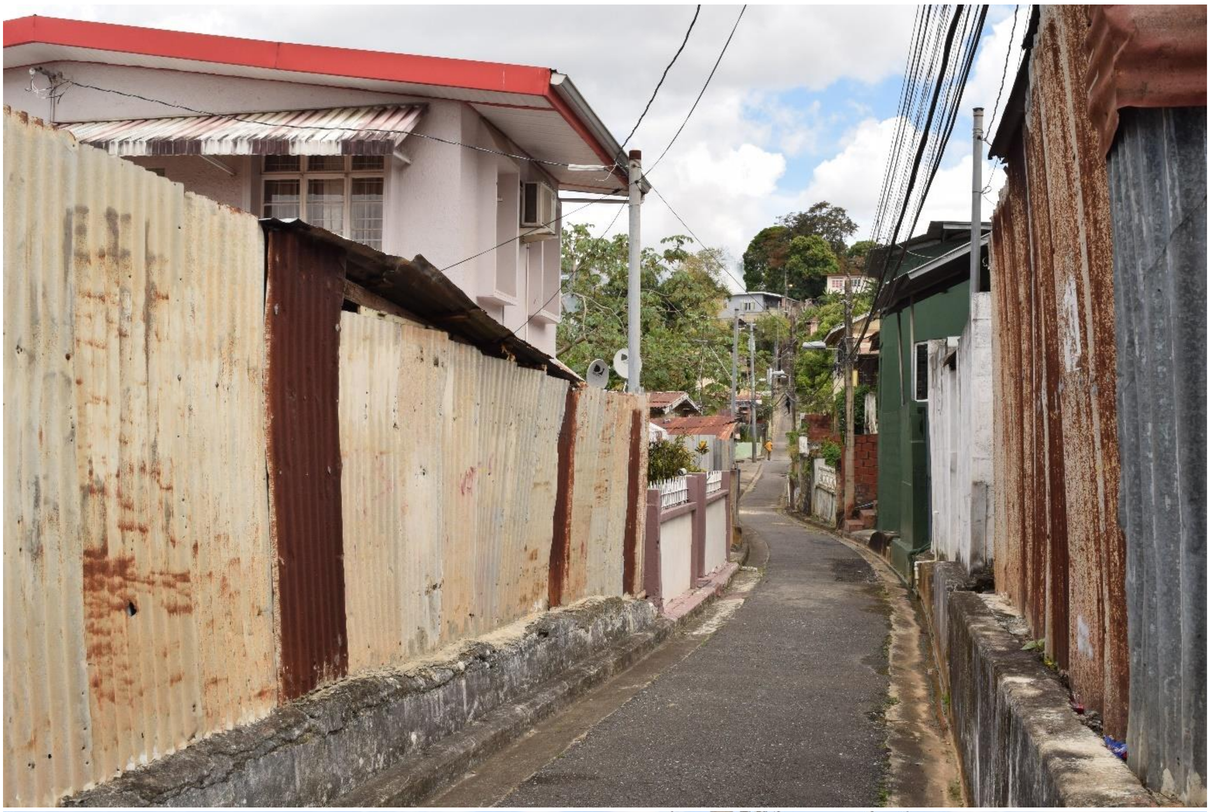

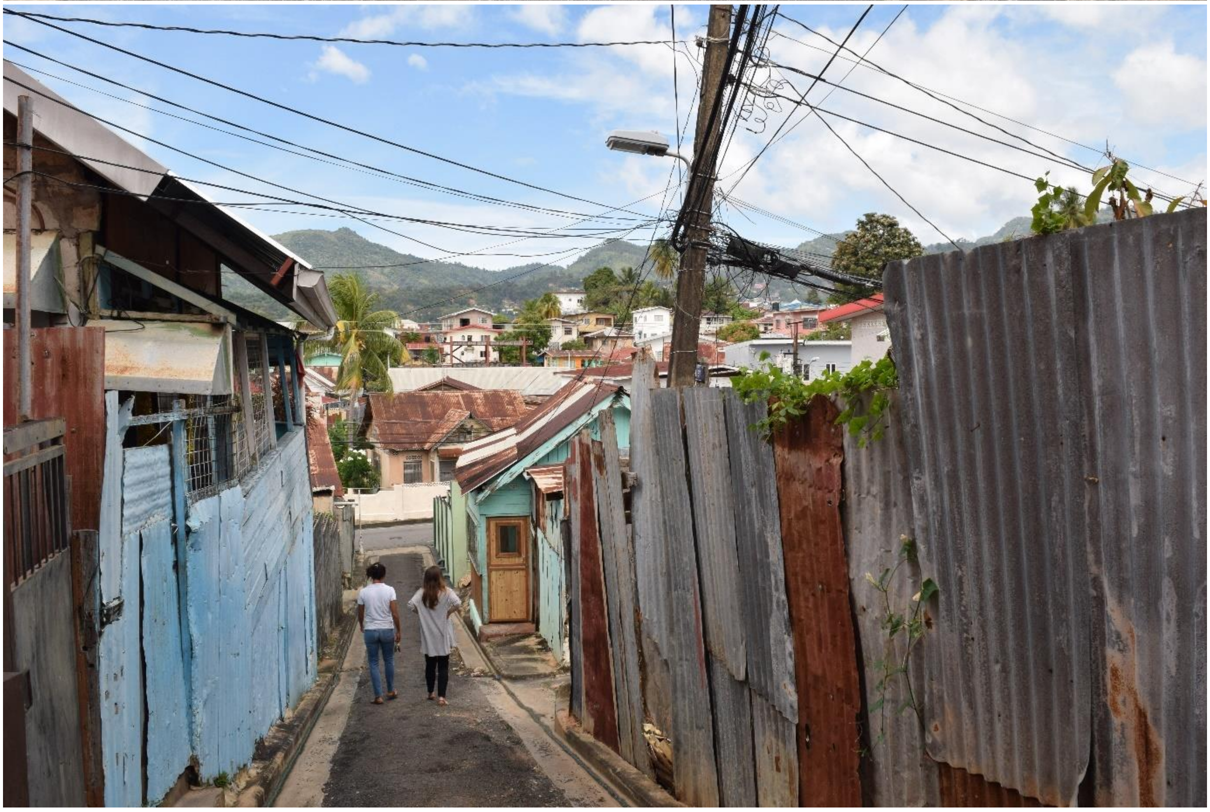

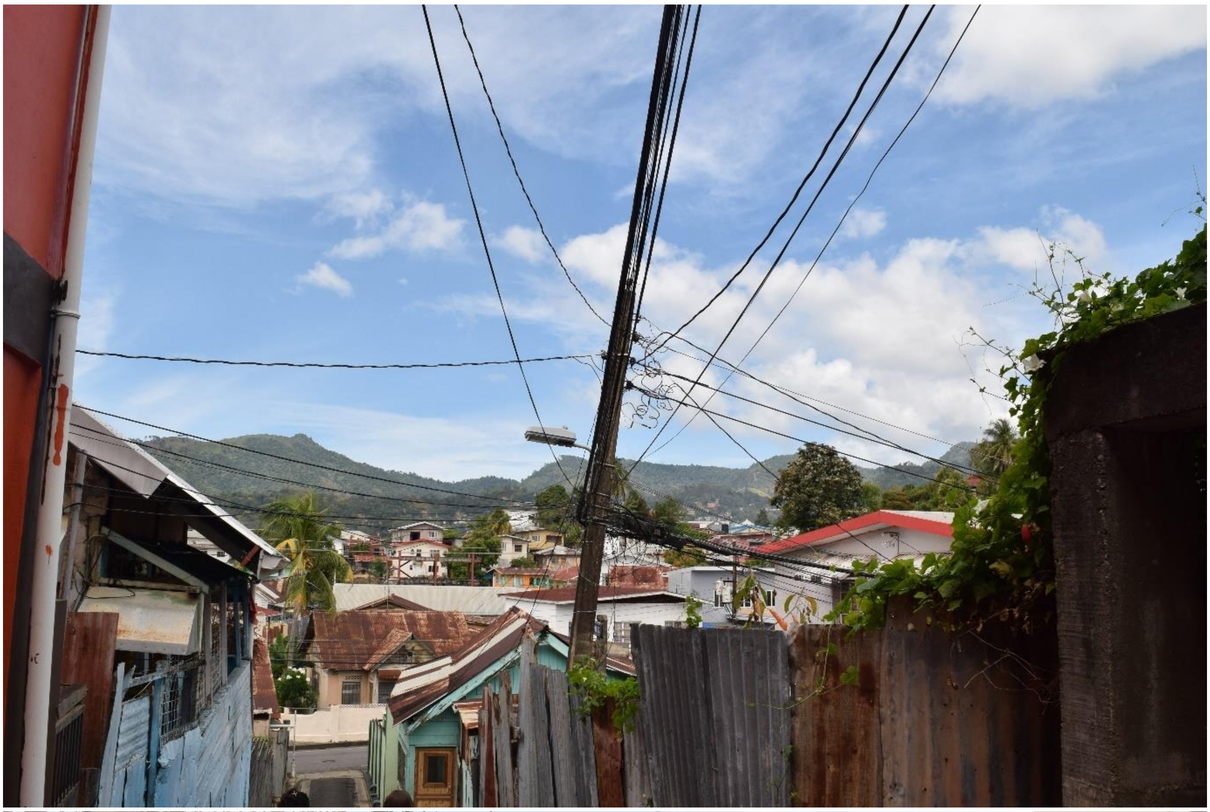

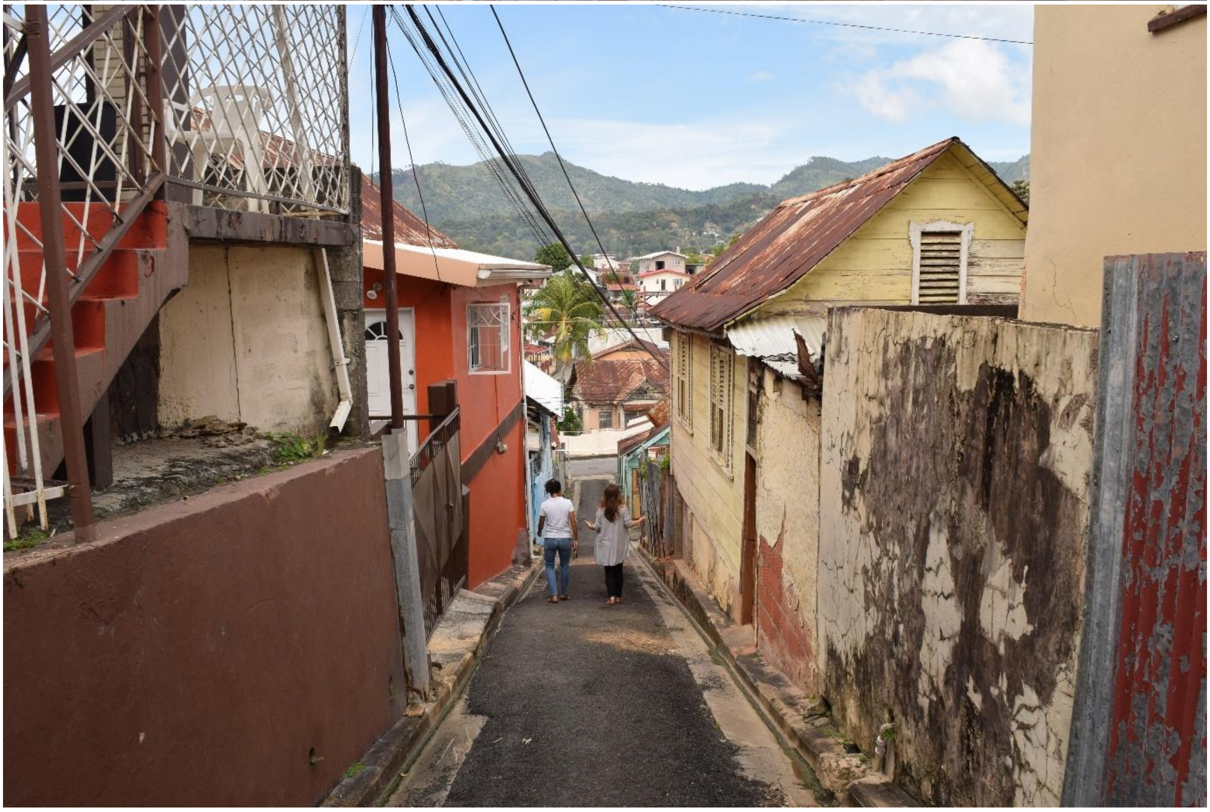

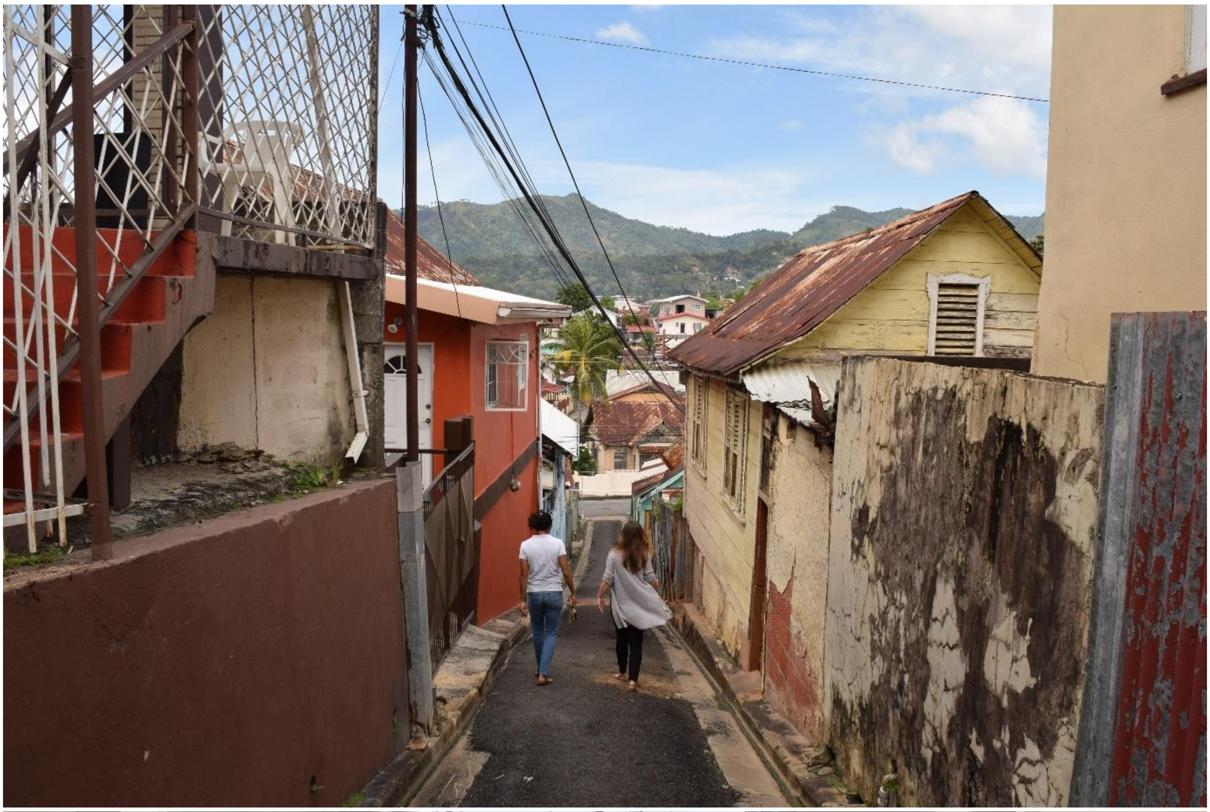

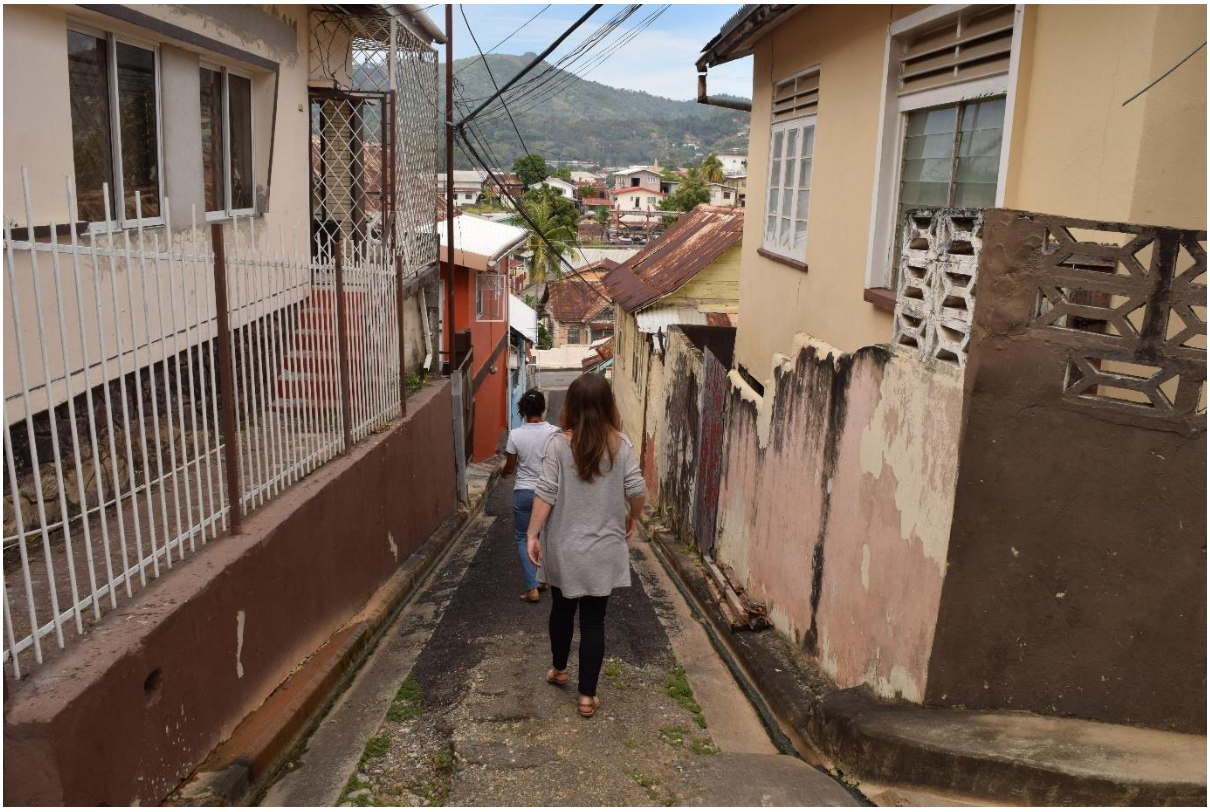

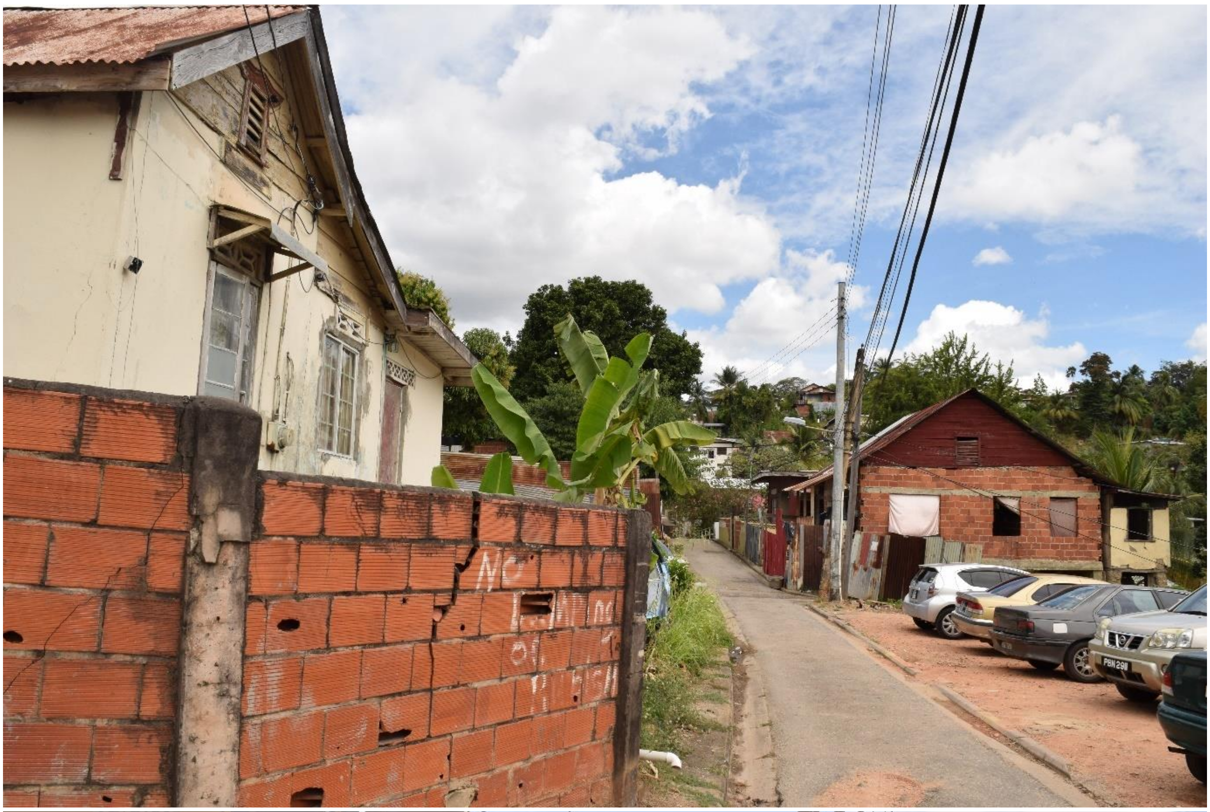
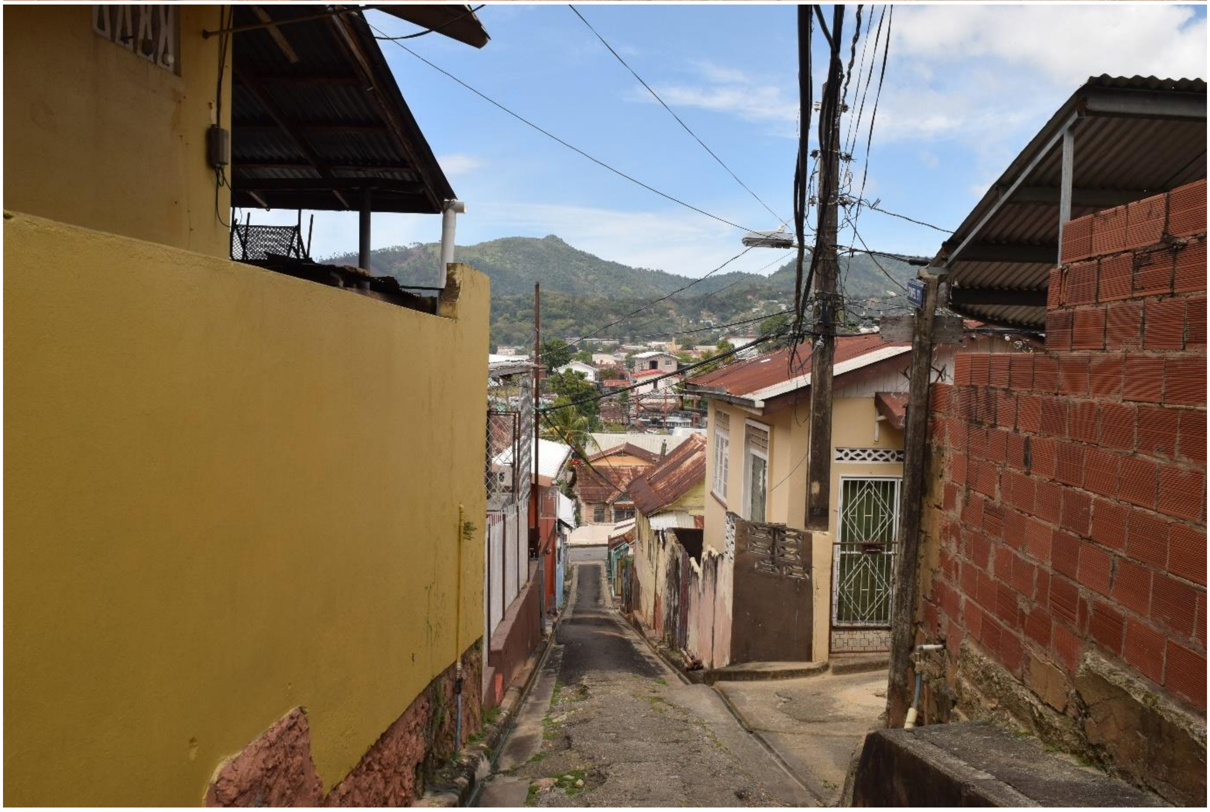

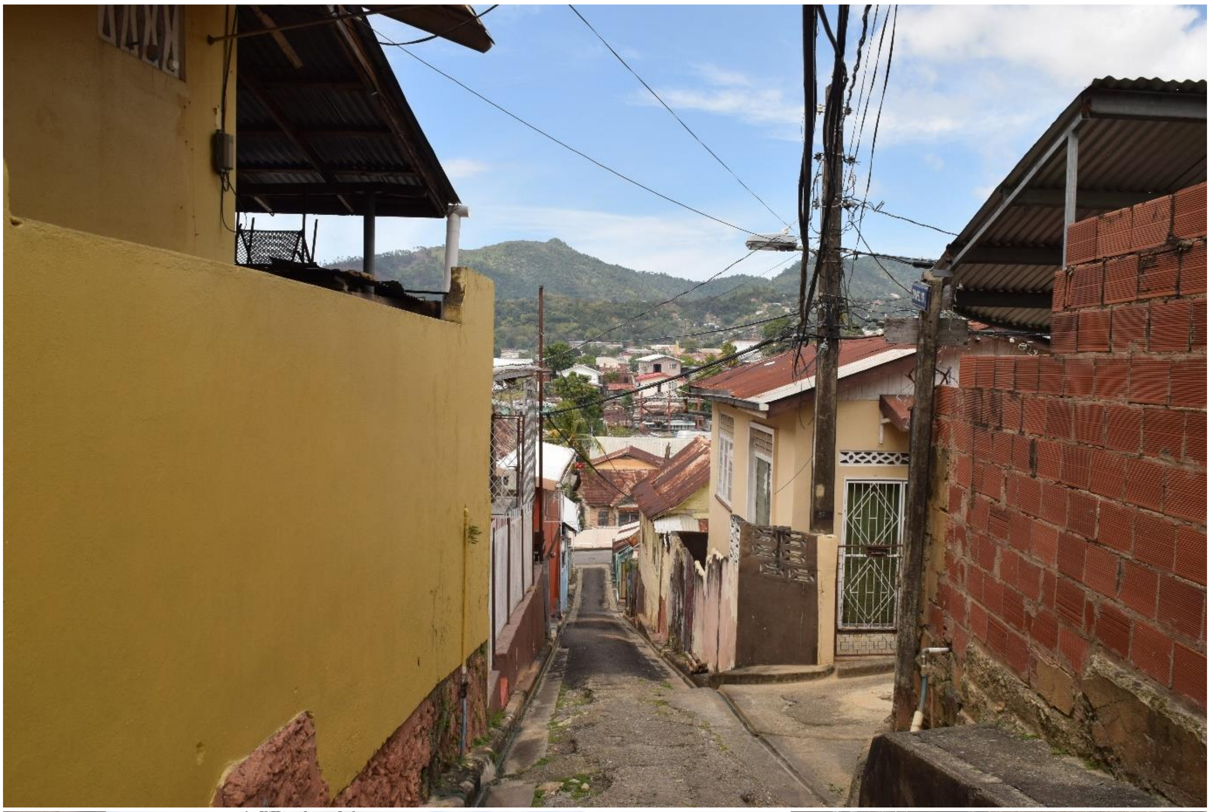
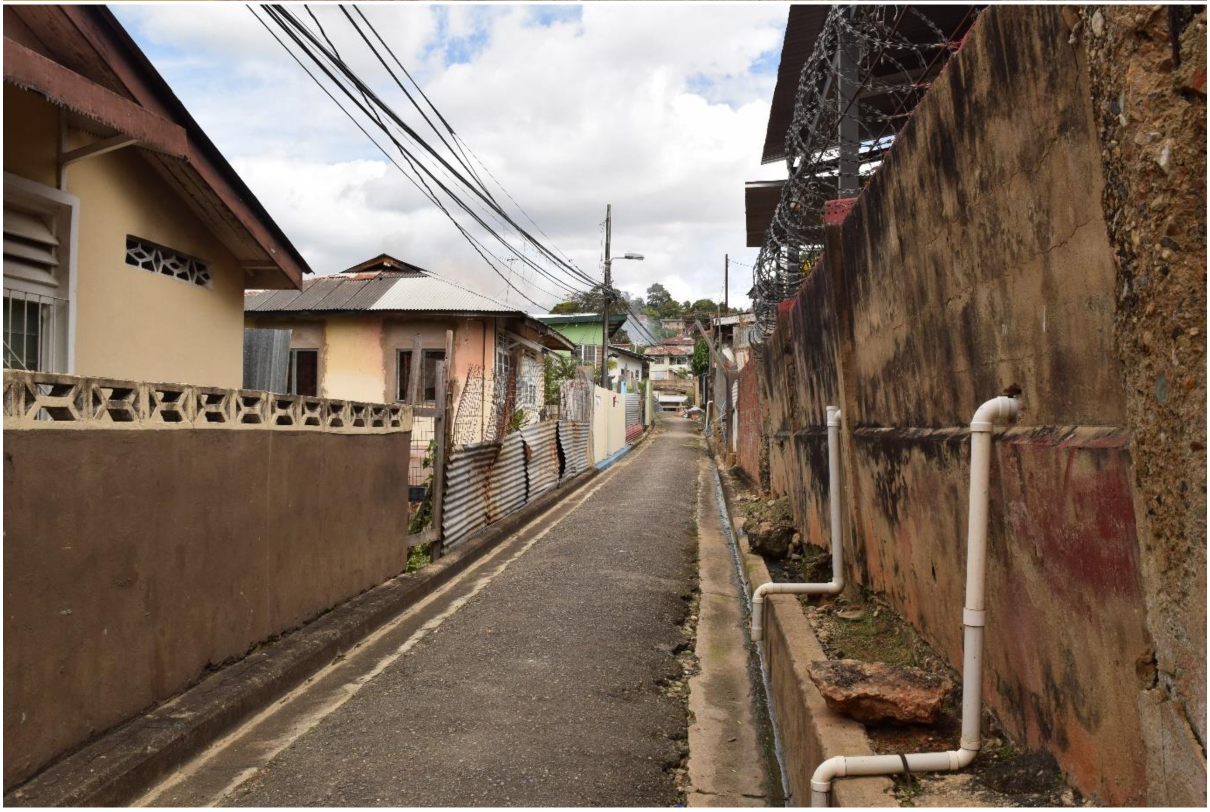

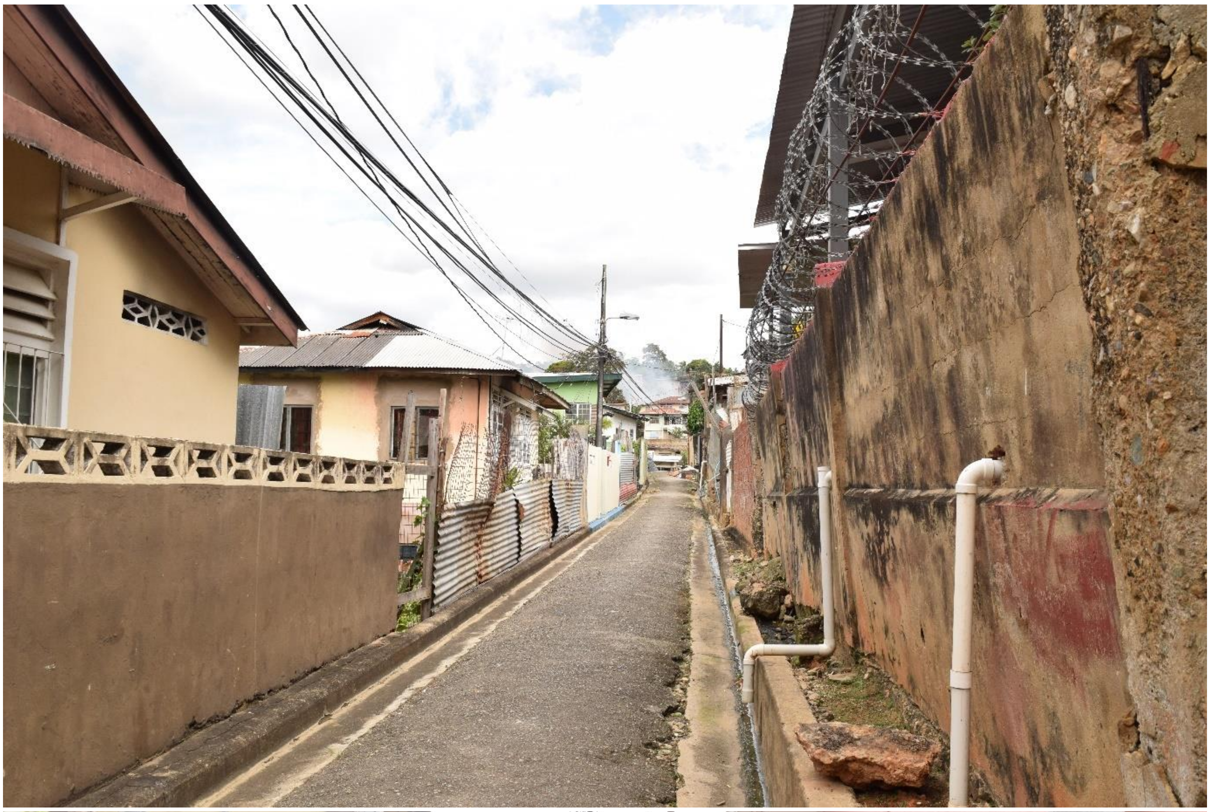

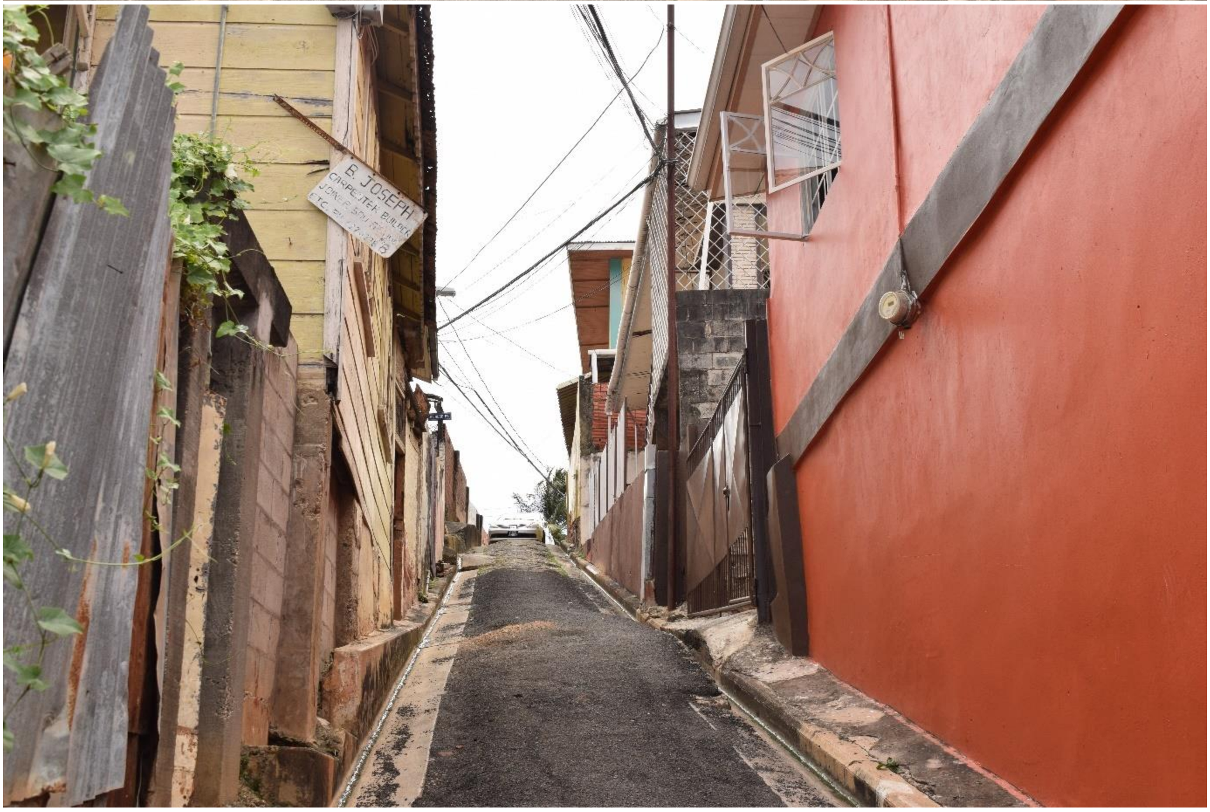
B. JOSEPH
CARPENTER BUILDER

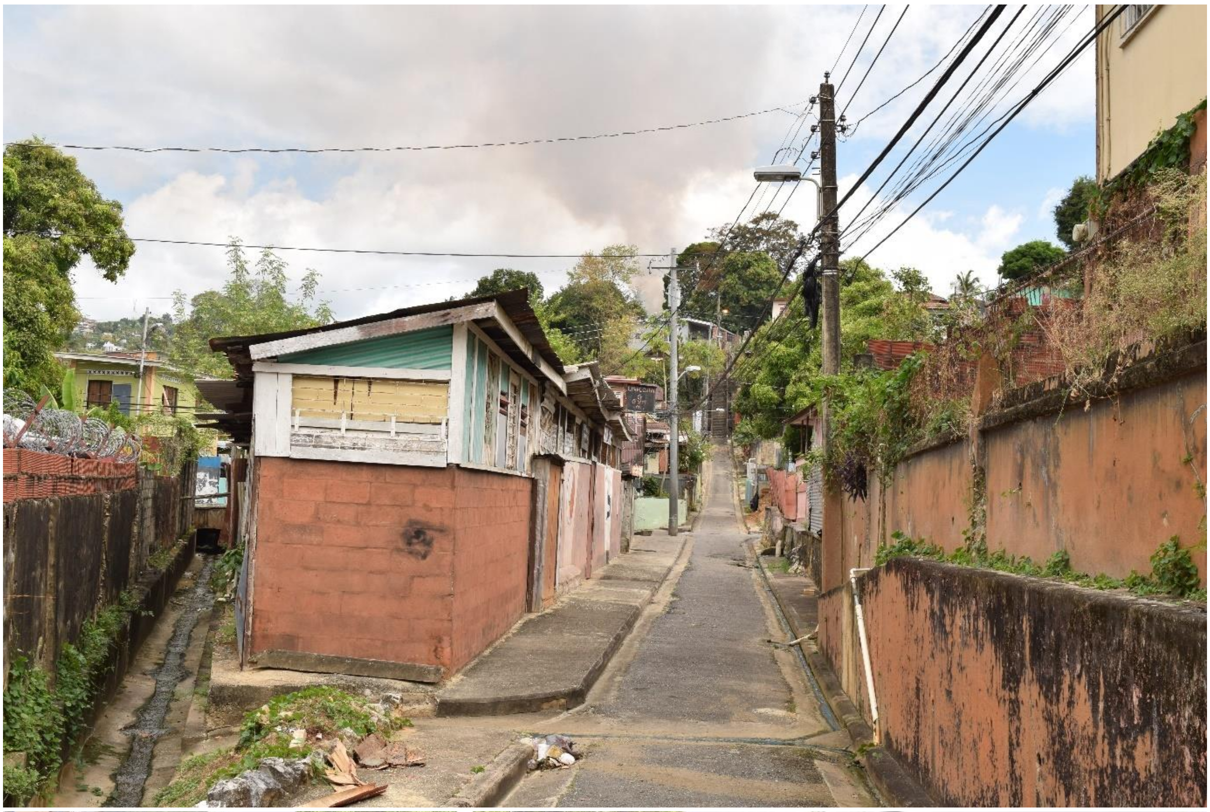

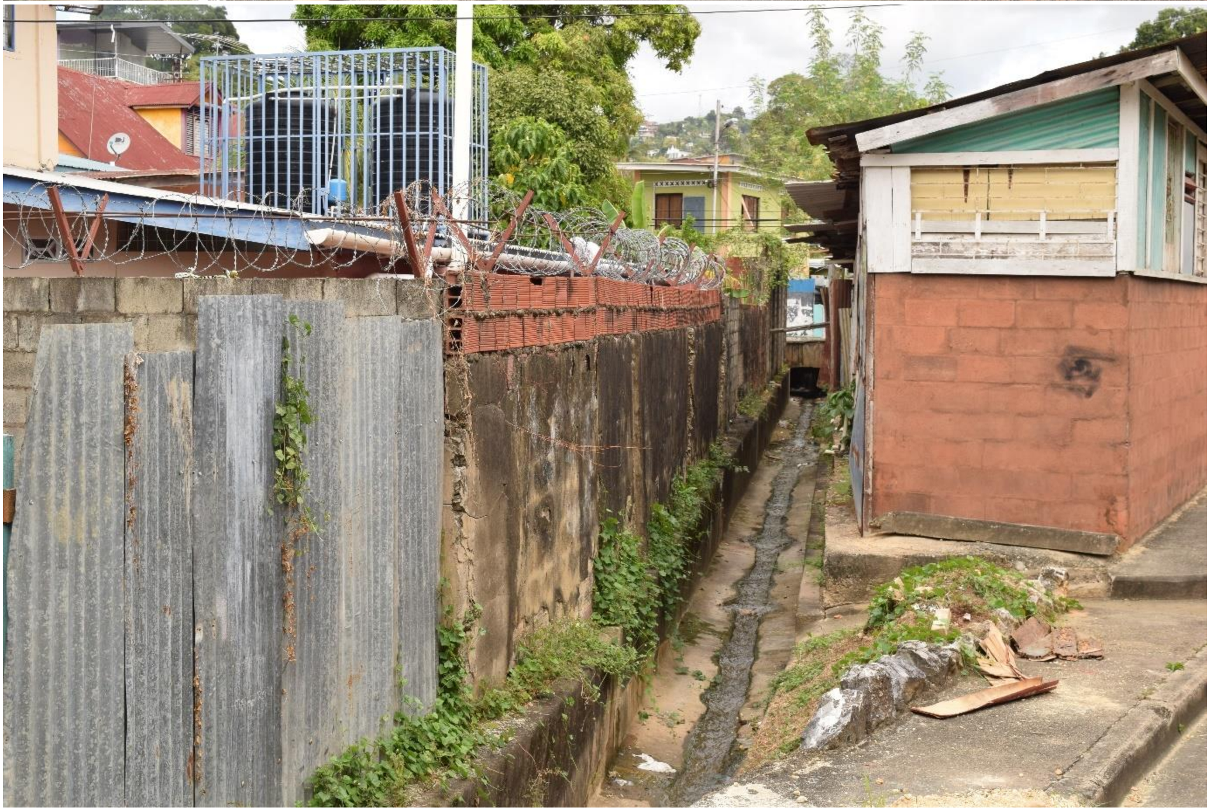

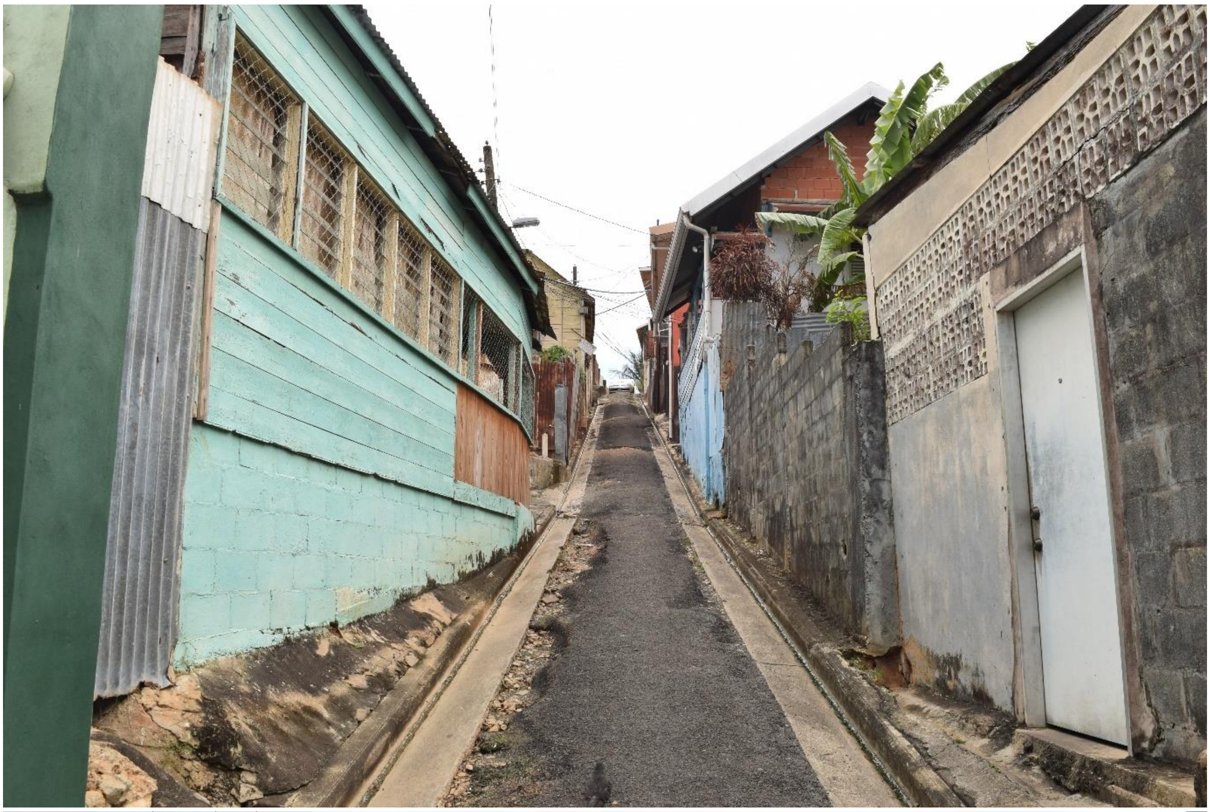

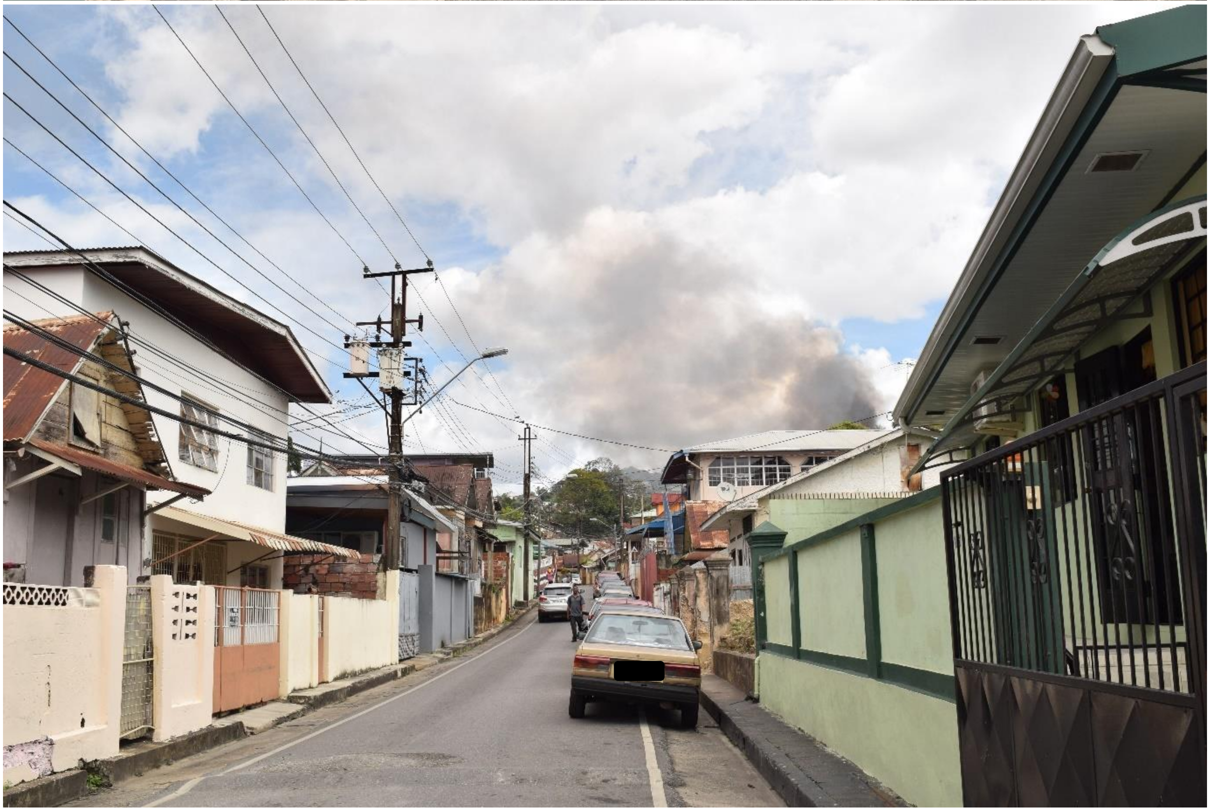

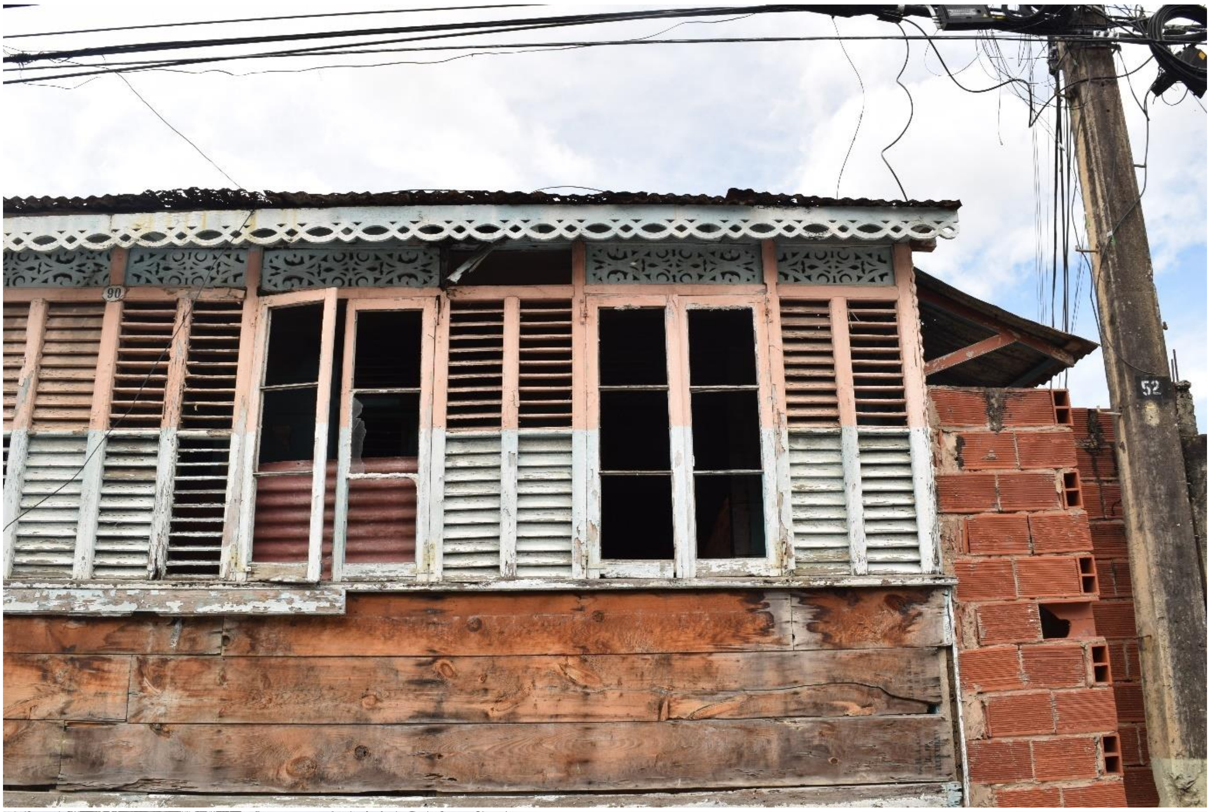
90
52

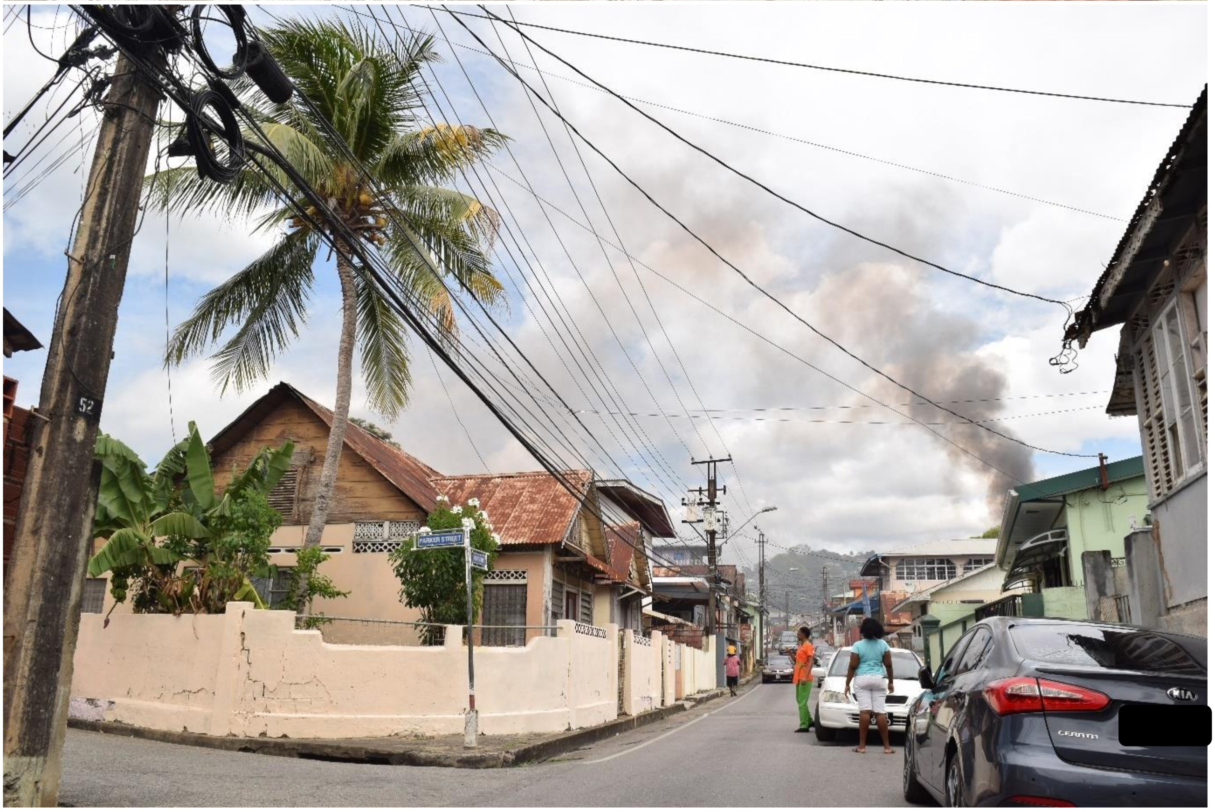
52
PARKER STREET
KIA
CERATO

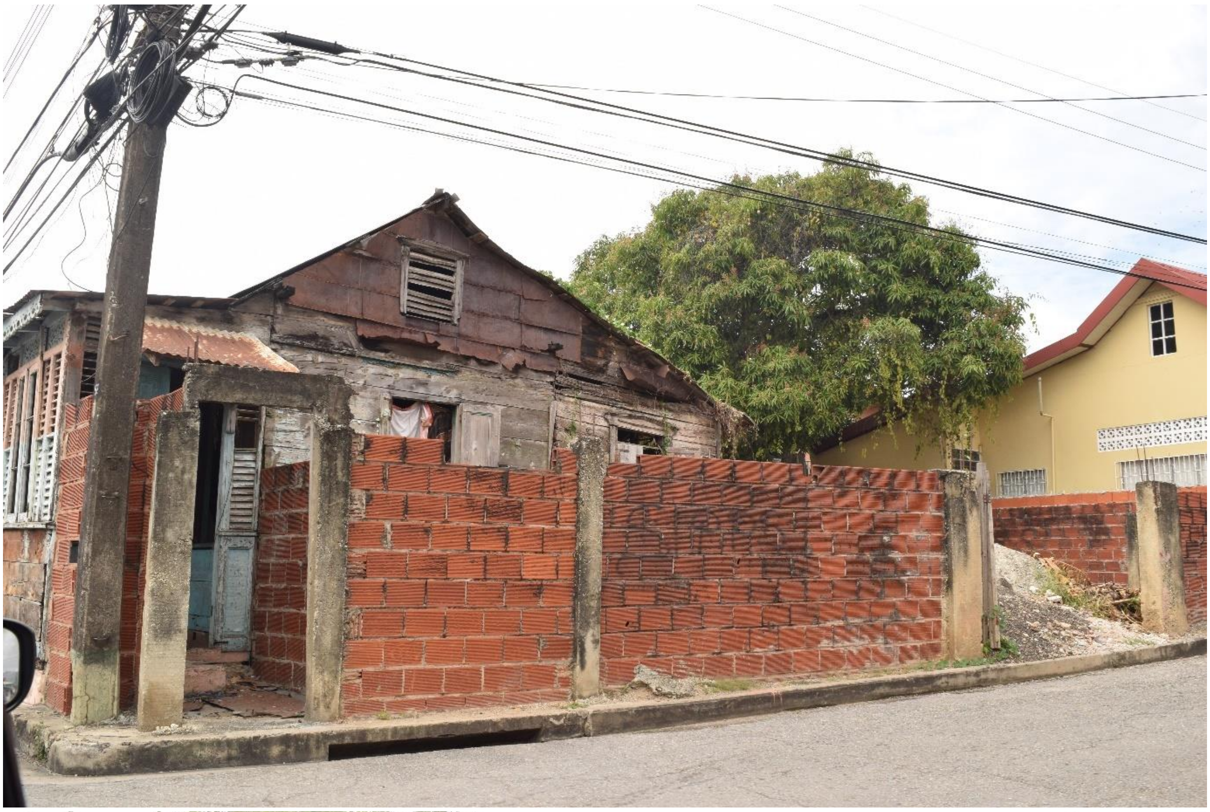

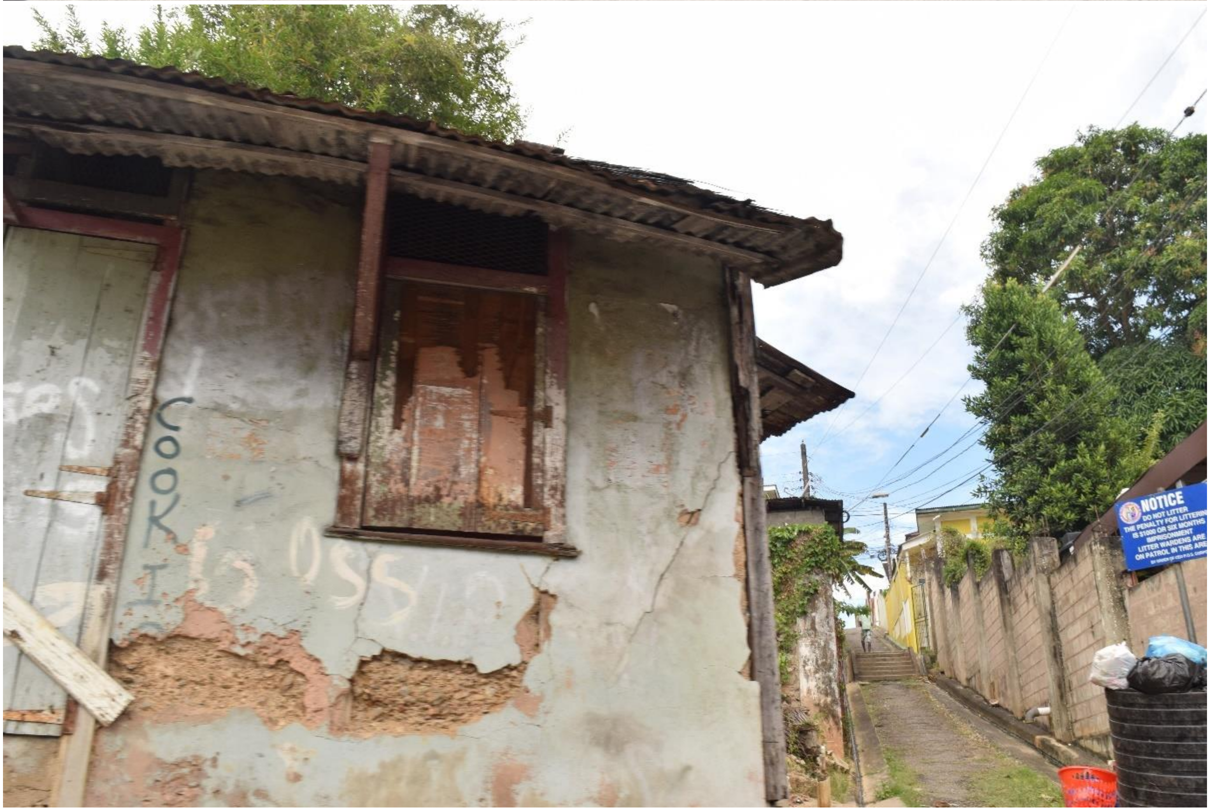
COOK
NOTICE
DO NOT LITTER
IS $1000 OR SIX MONTHS
IMPRISONMENT
LITTER WARDENS ARE

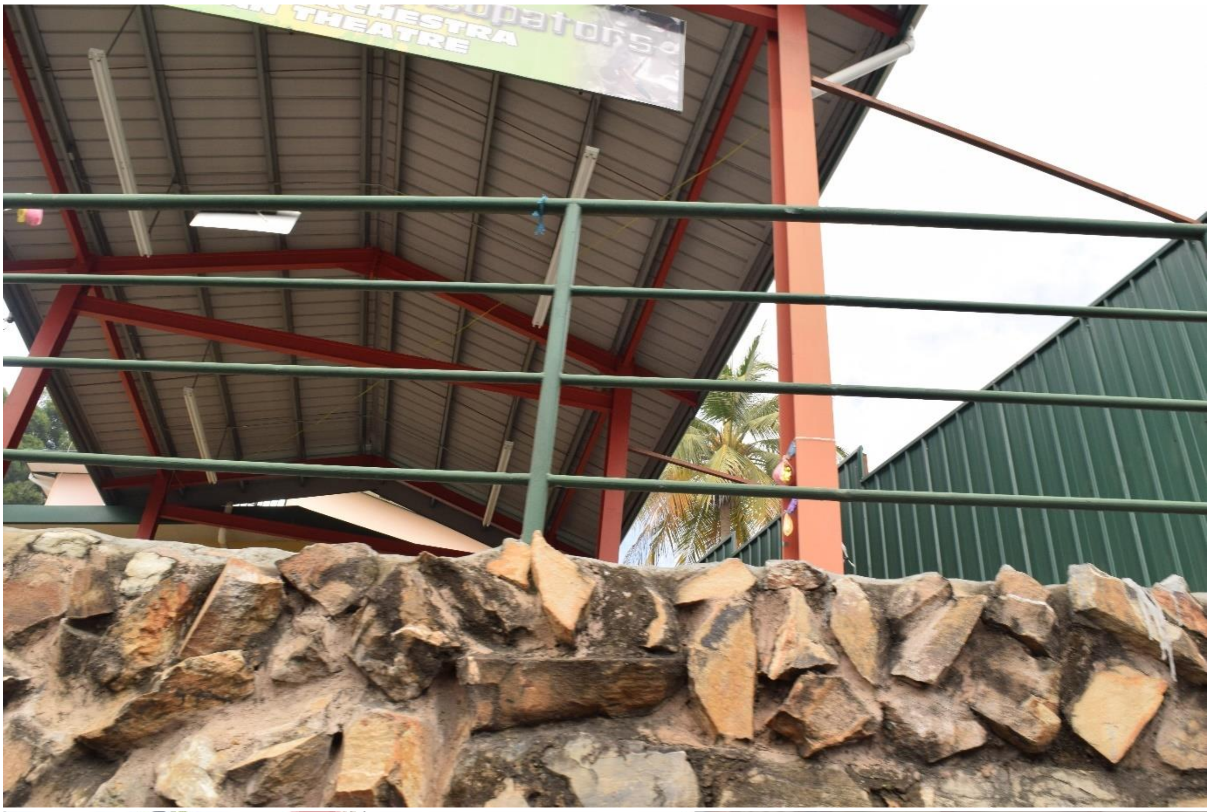
THEATRE

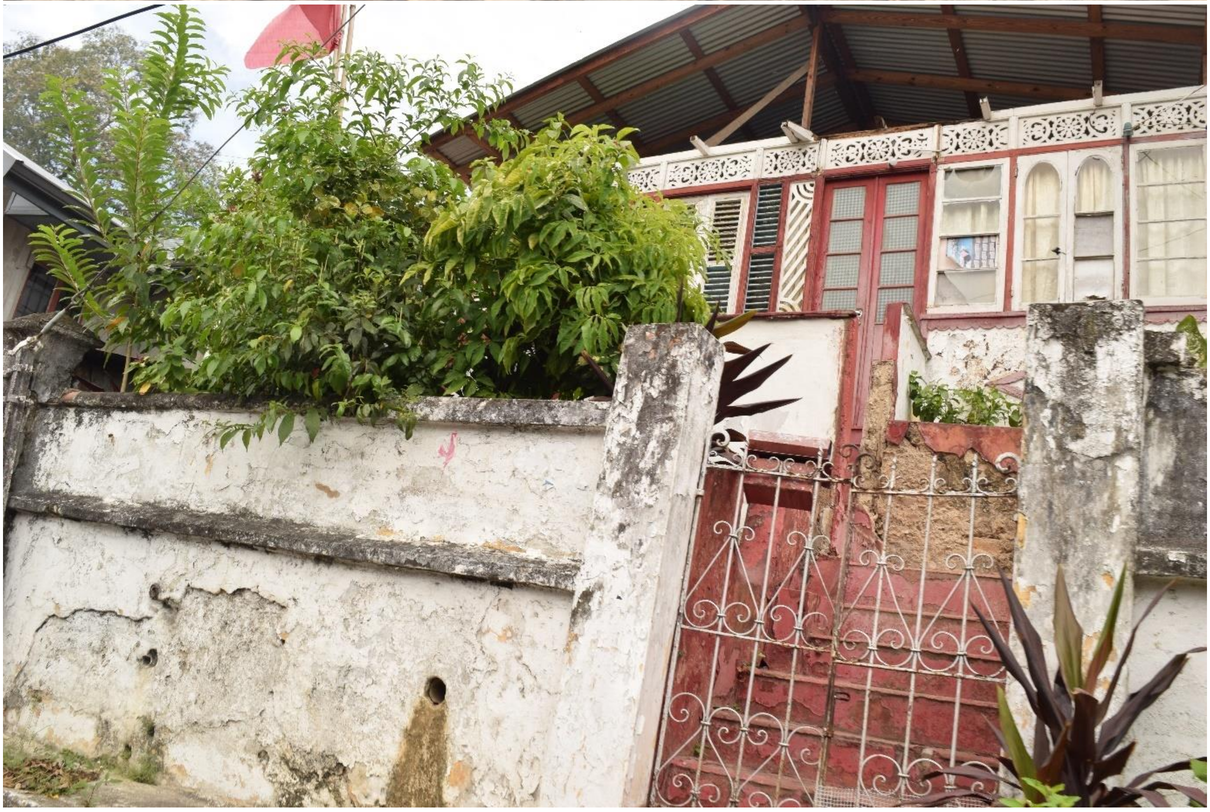

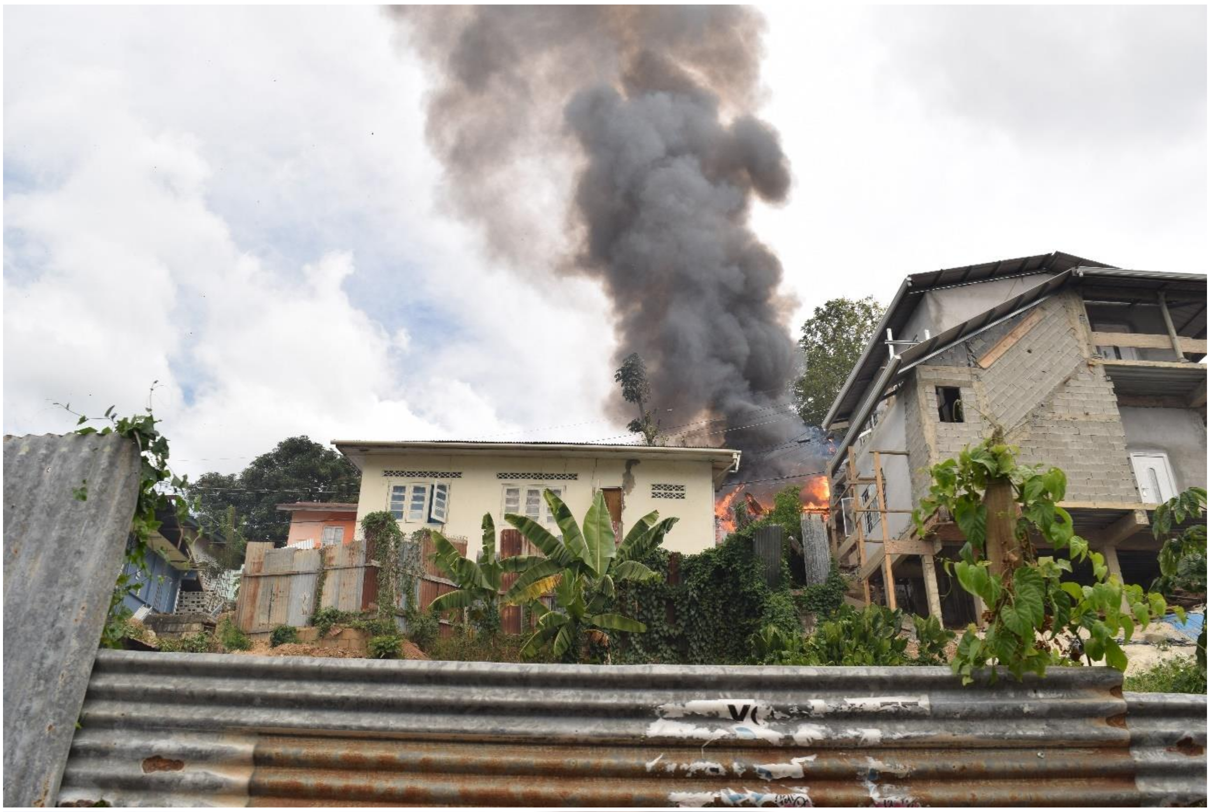

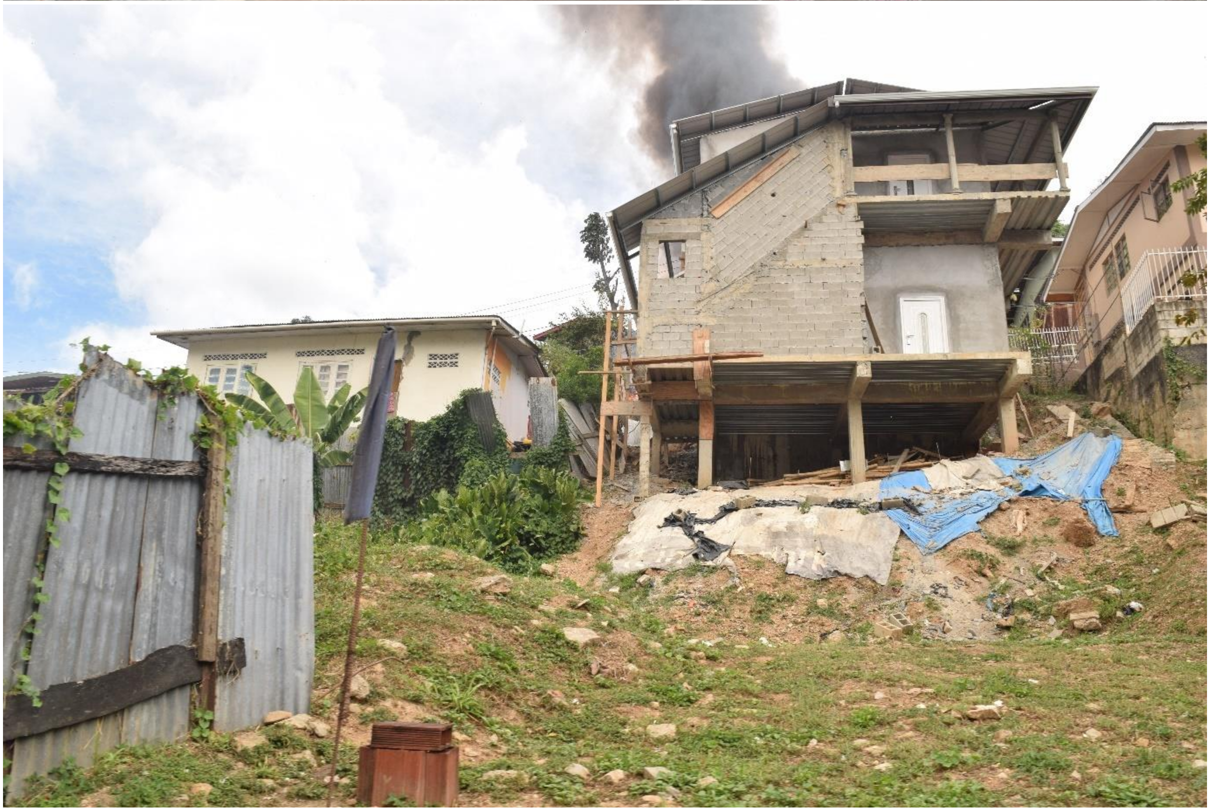

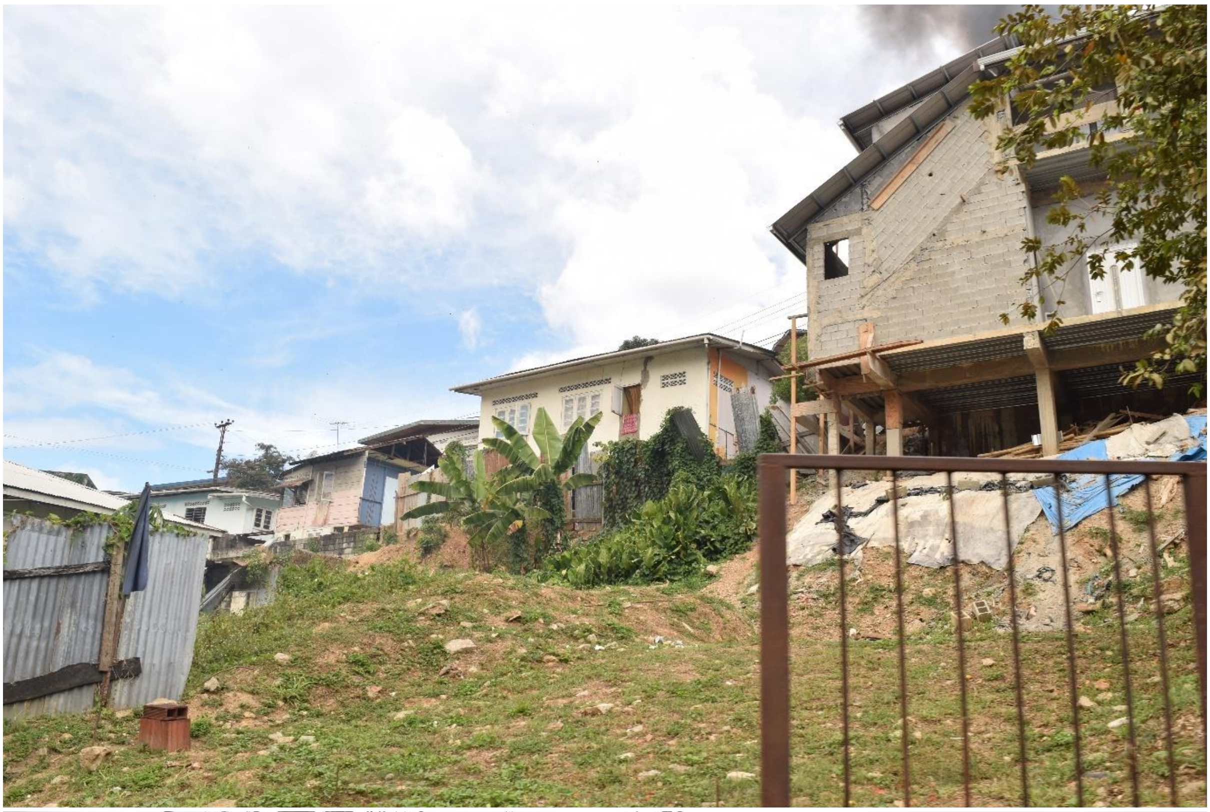

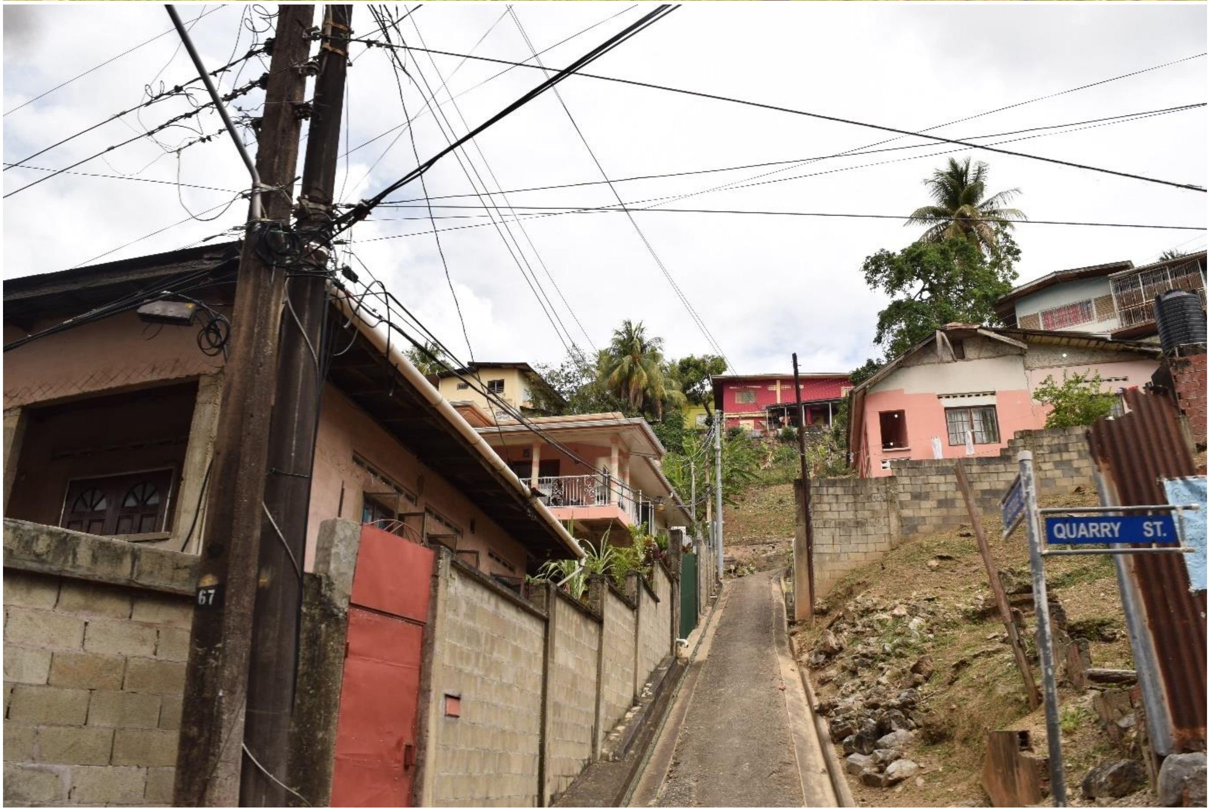
67
QUARRY ST.

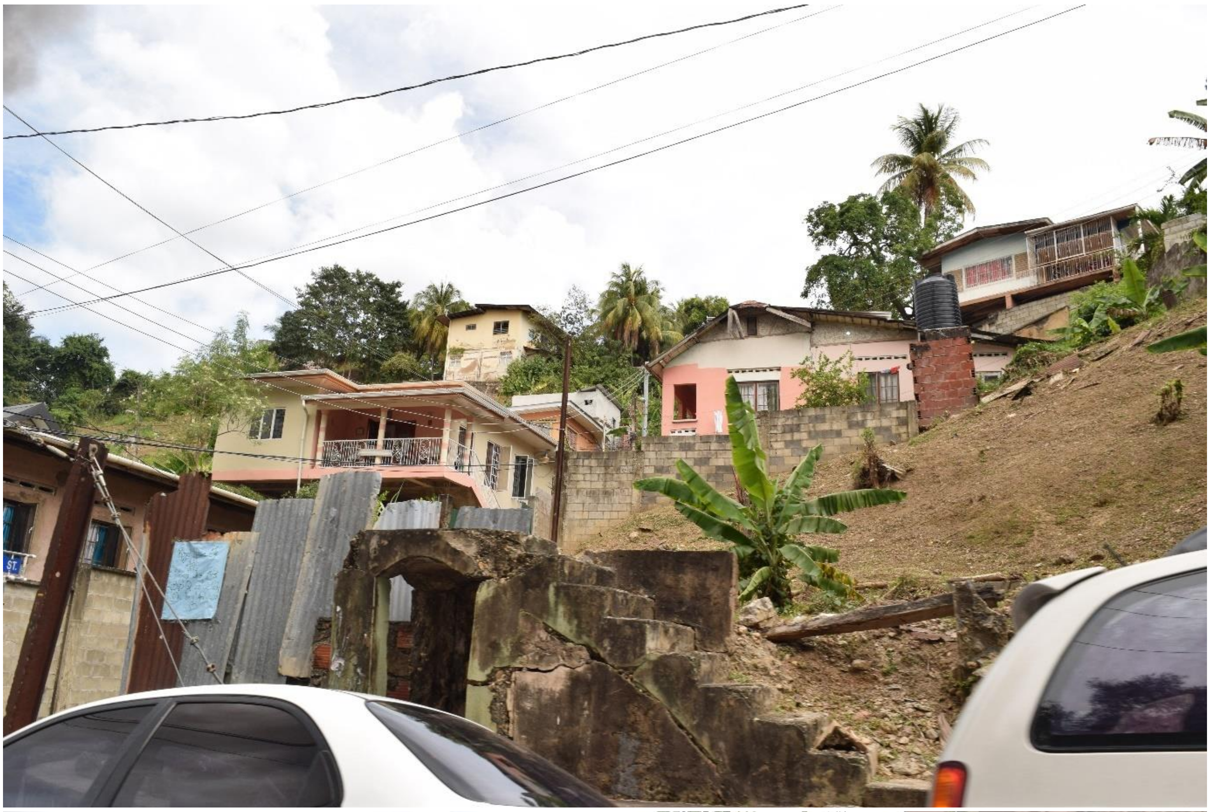

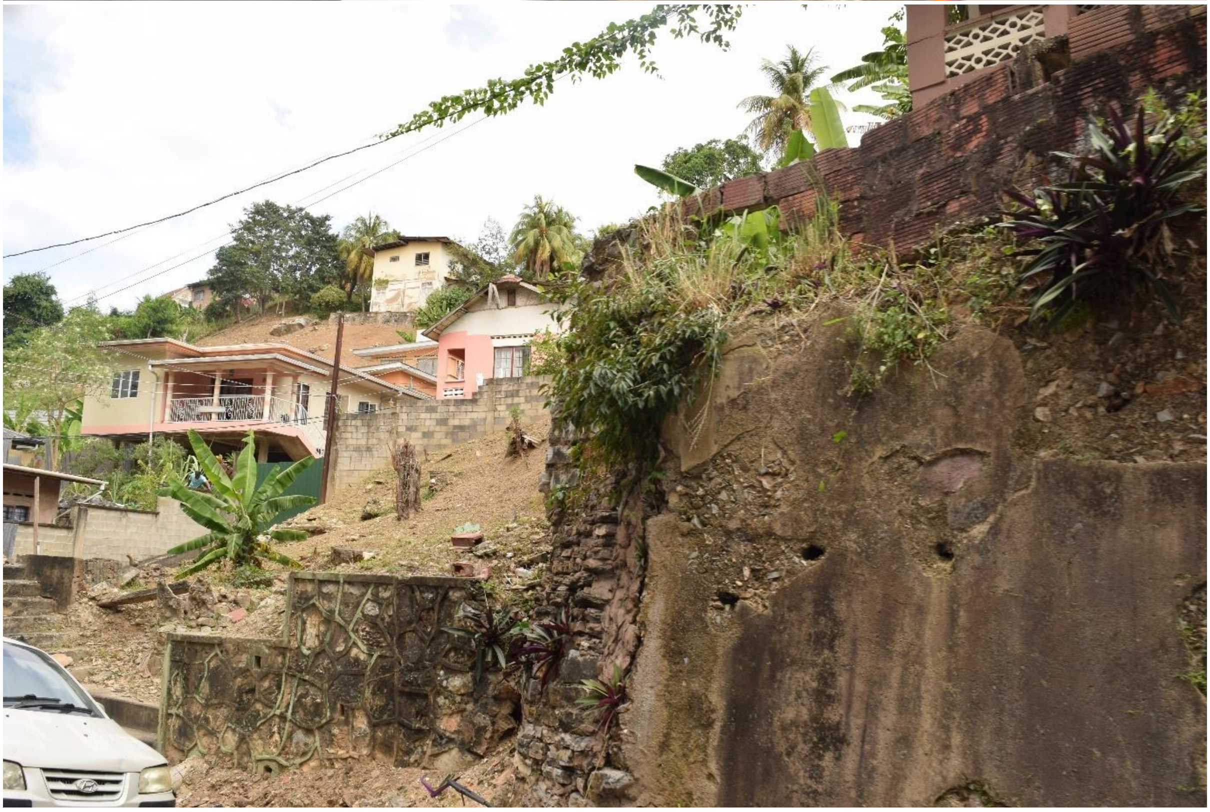

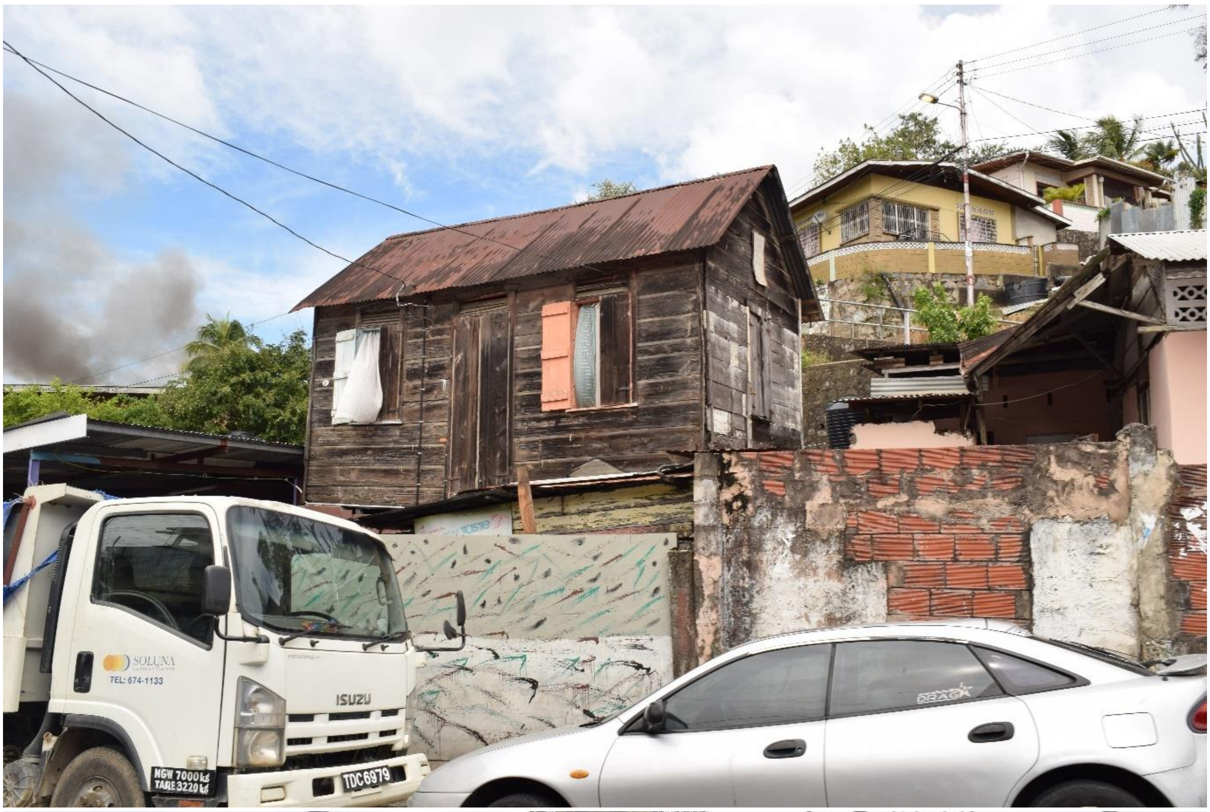
SOLUNA
TEL: 674-1133
ISUZU
TDC 6979

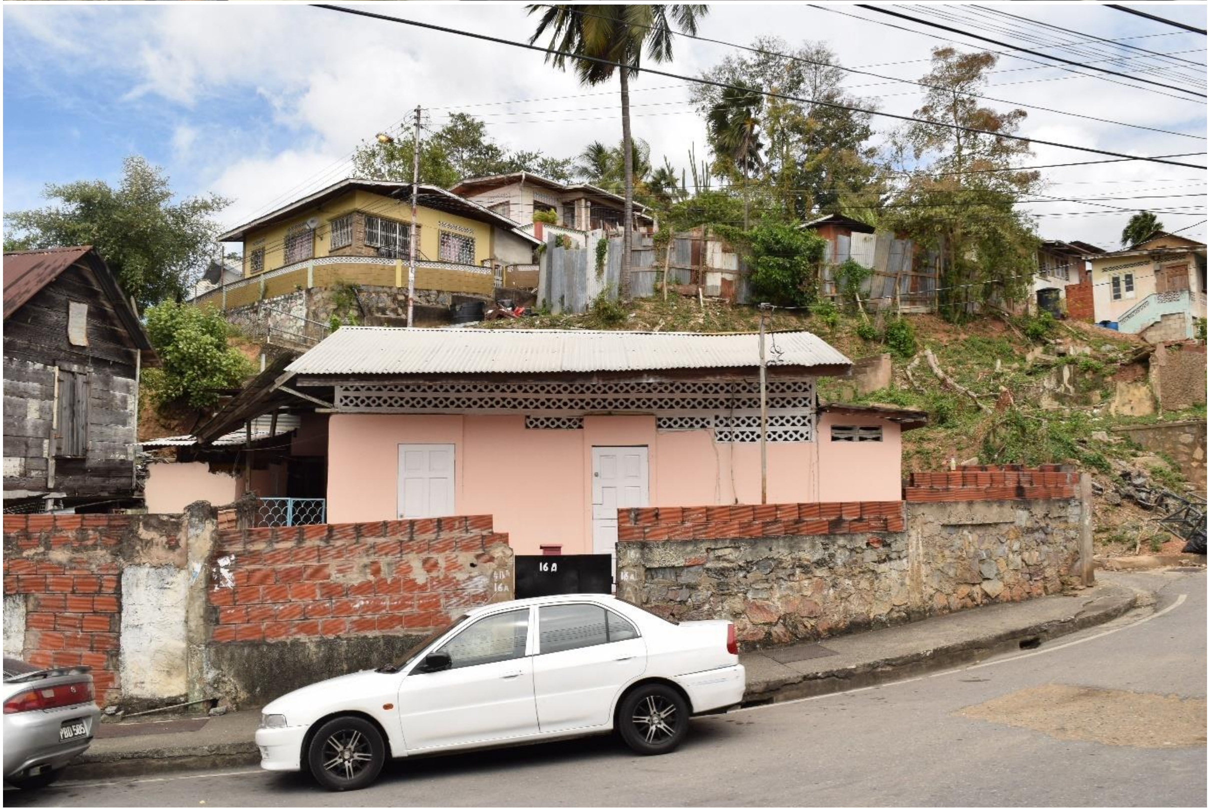
16 A

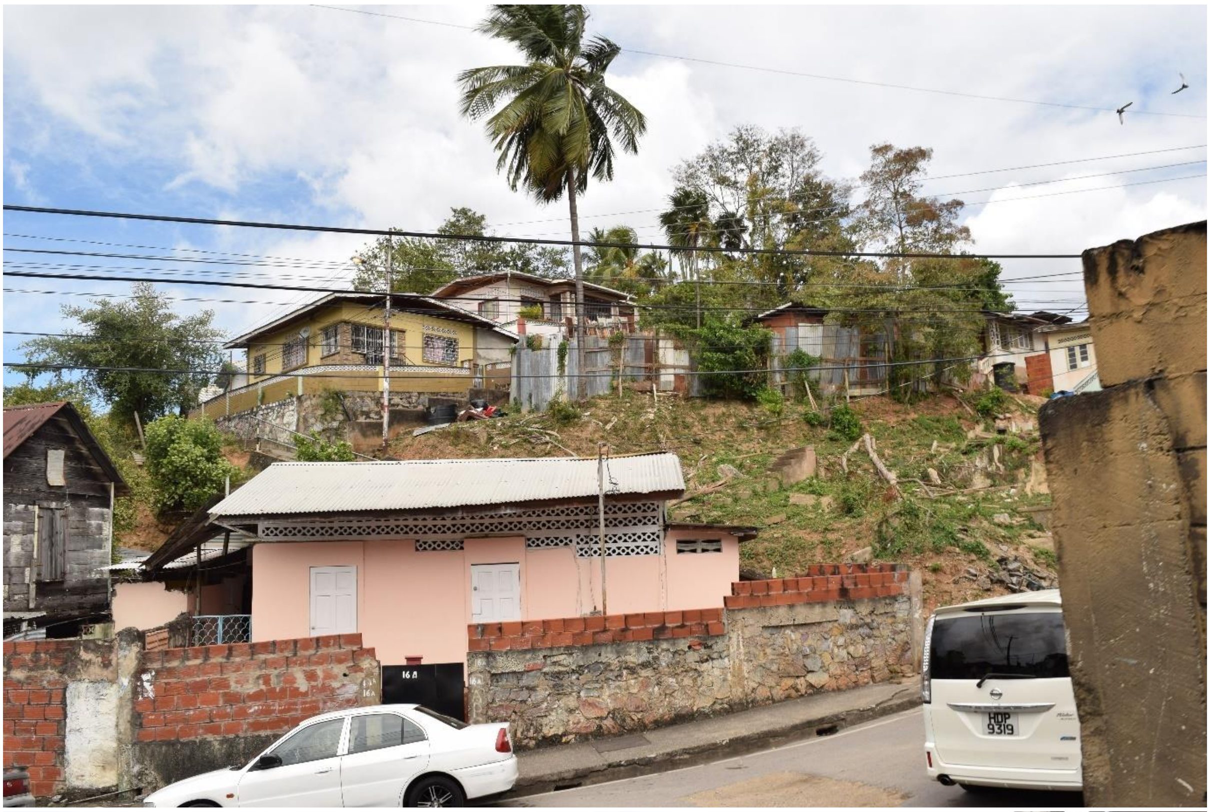
HDP
9319

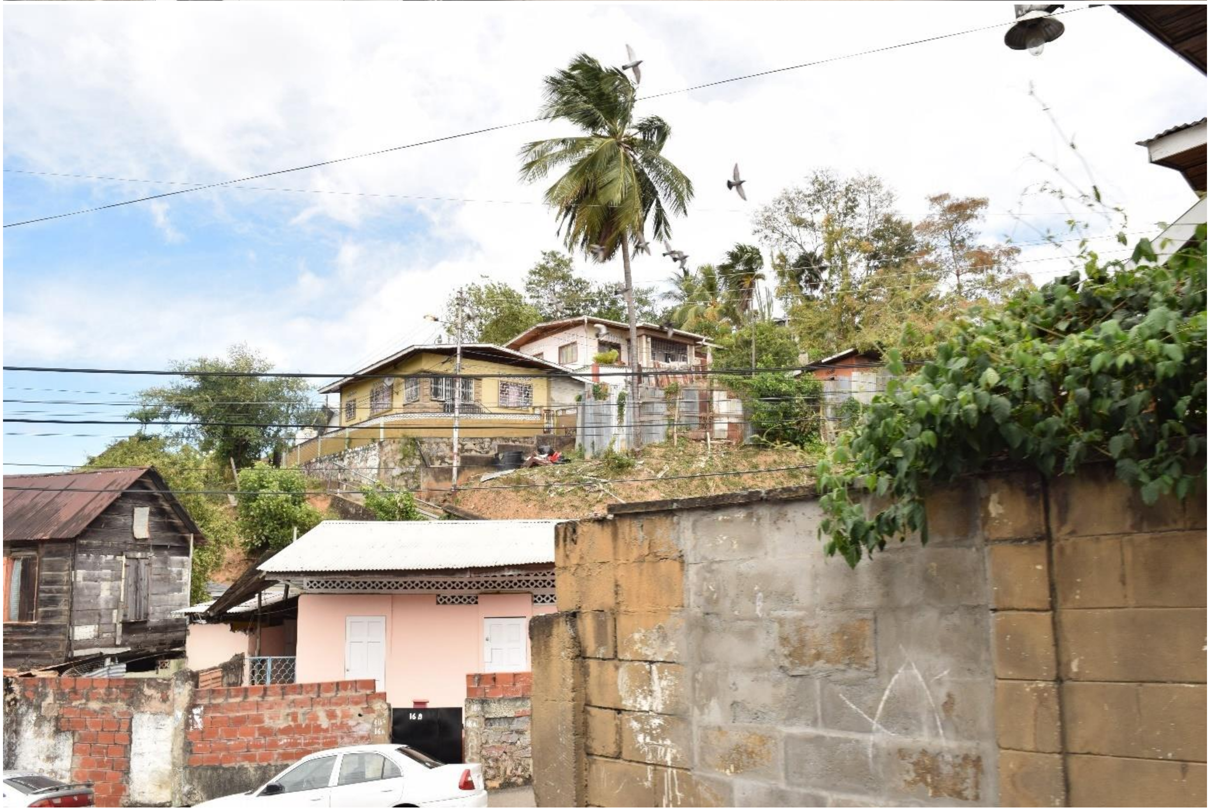

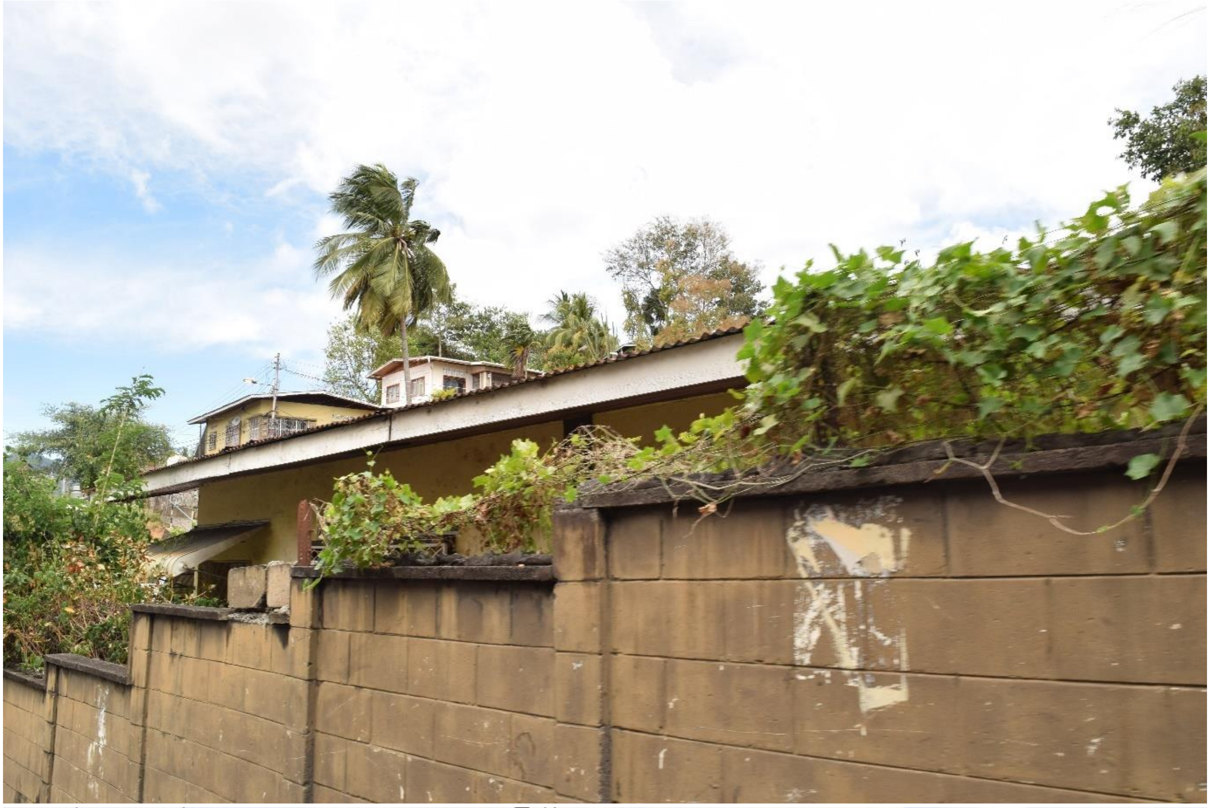

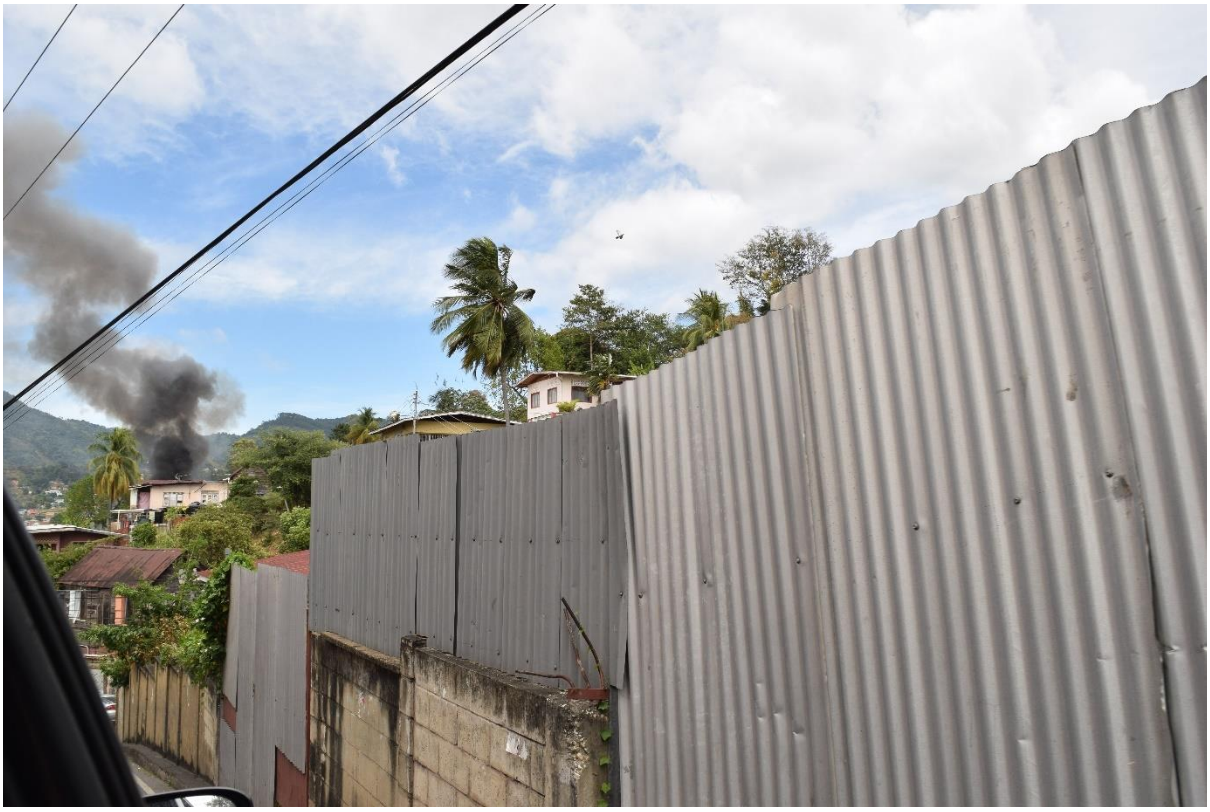

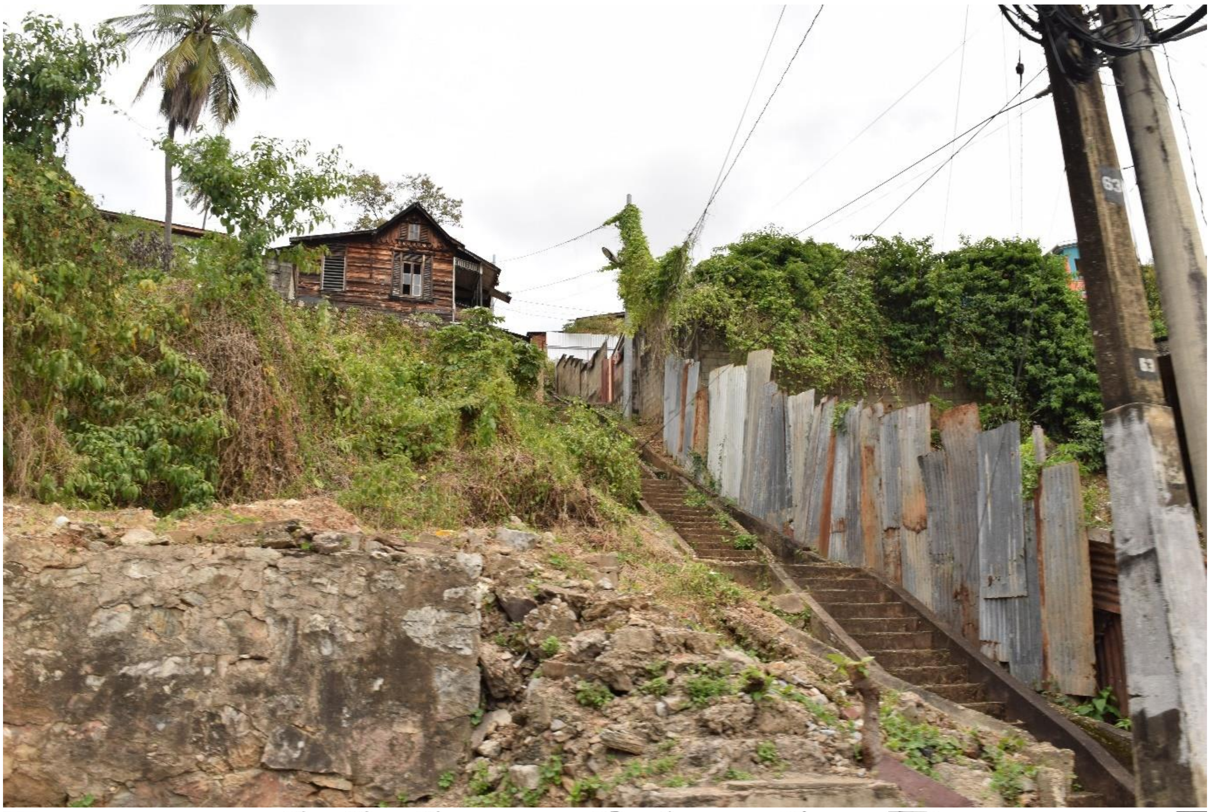
63

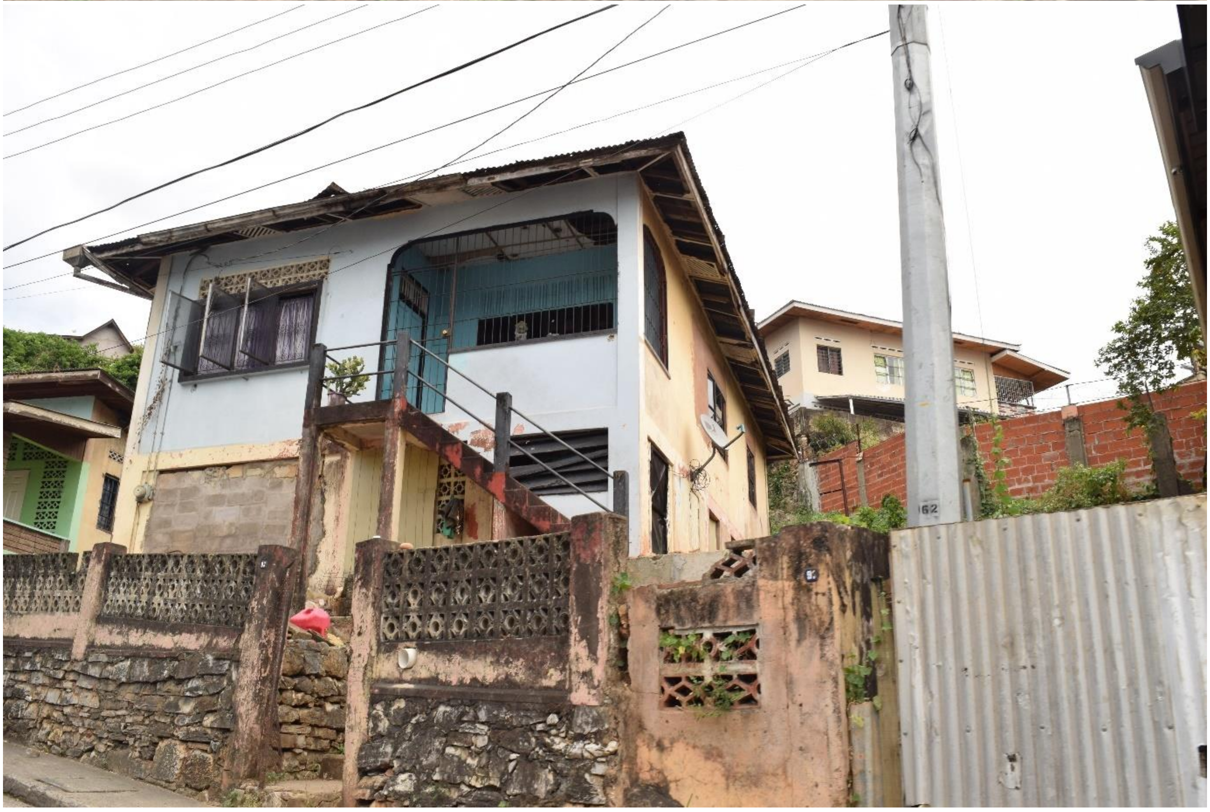
62

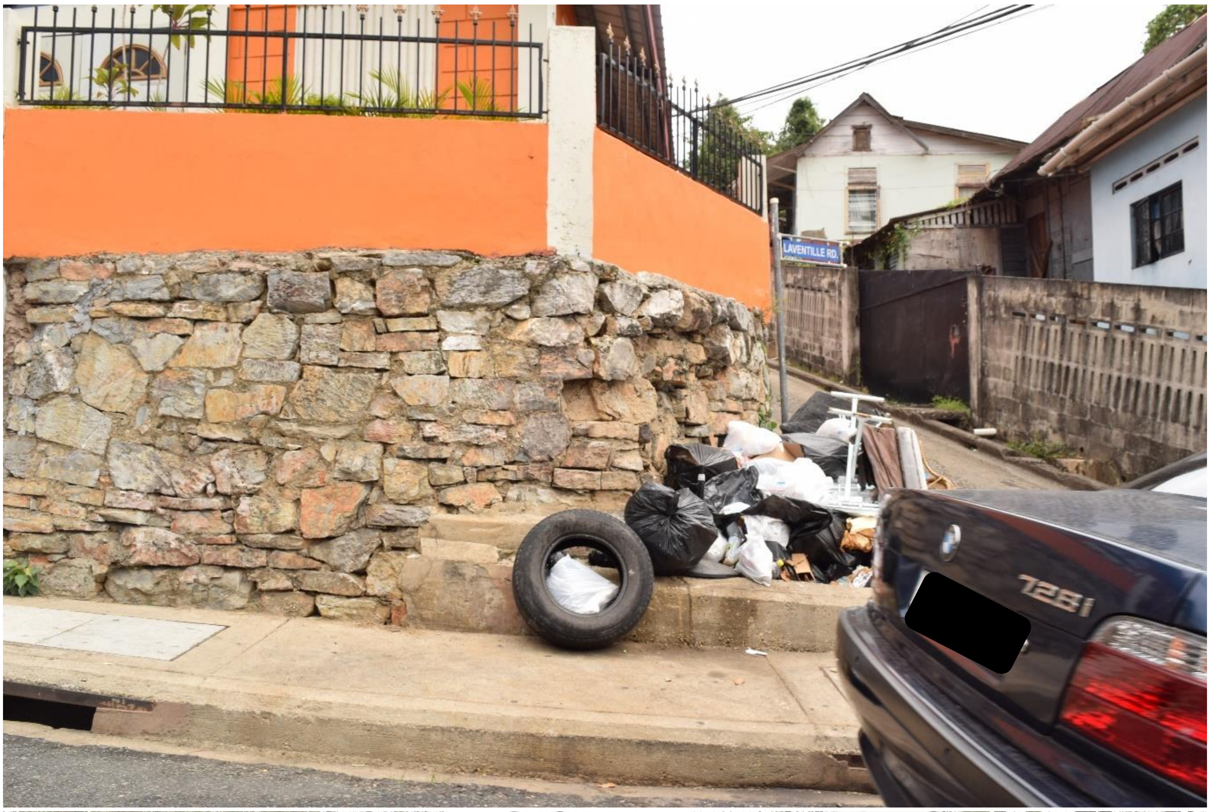
LAVENTILLE RD.
728i

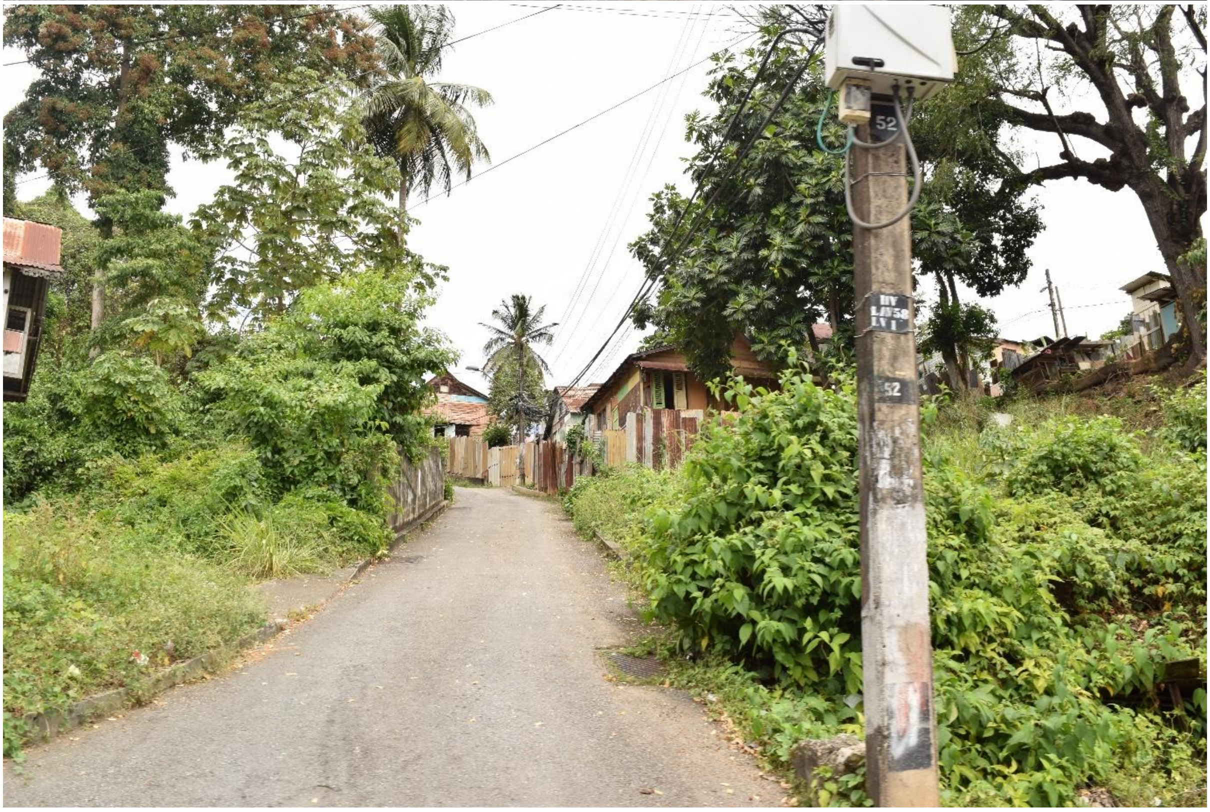
52
52

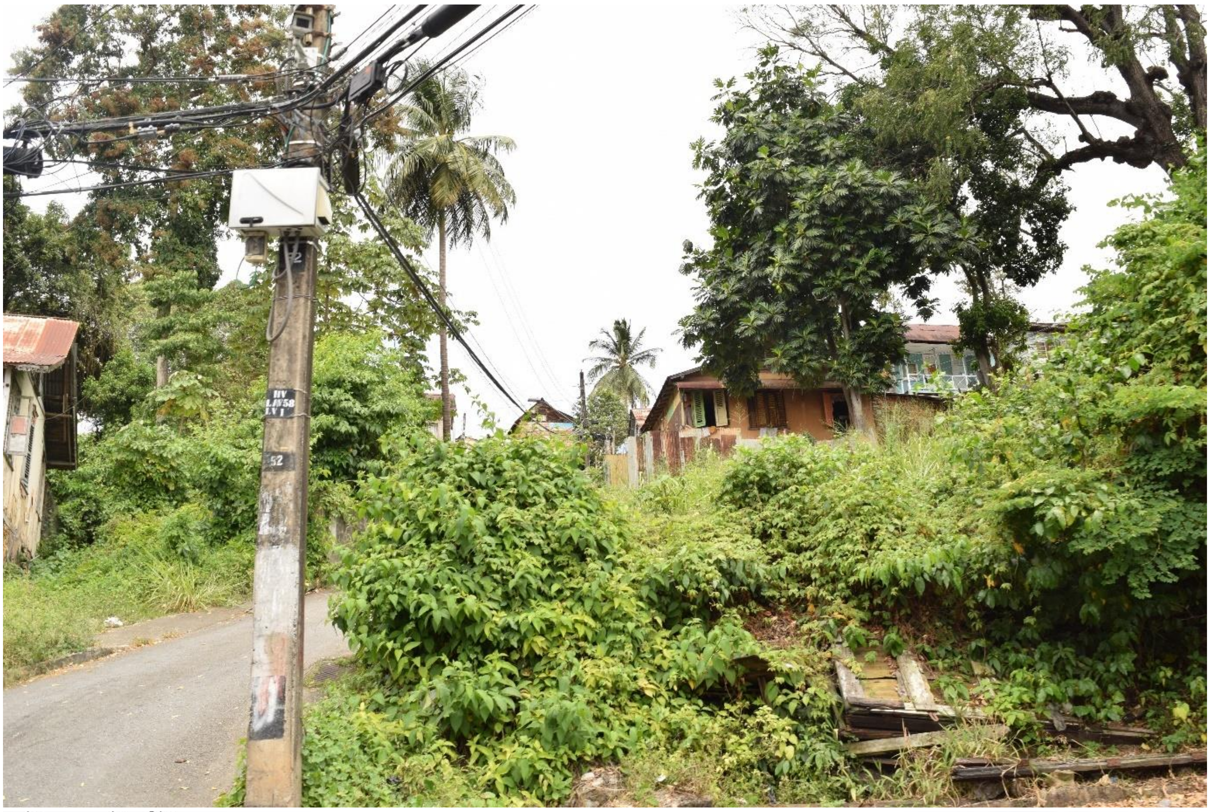

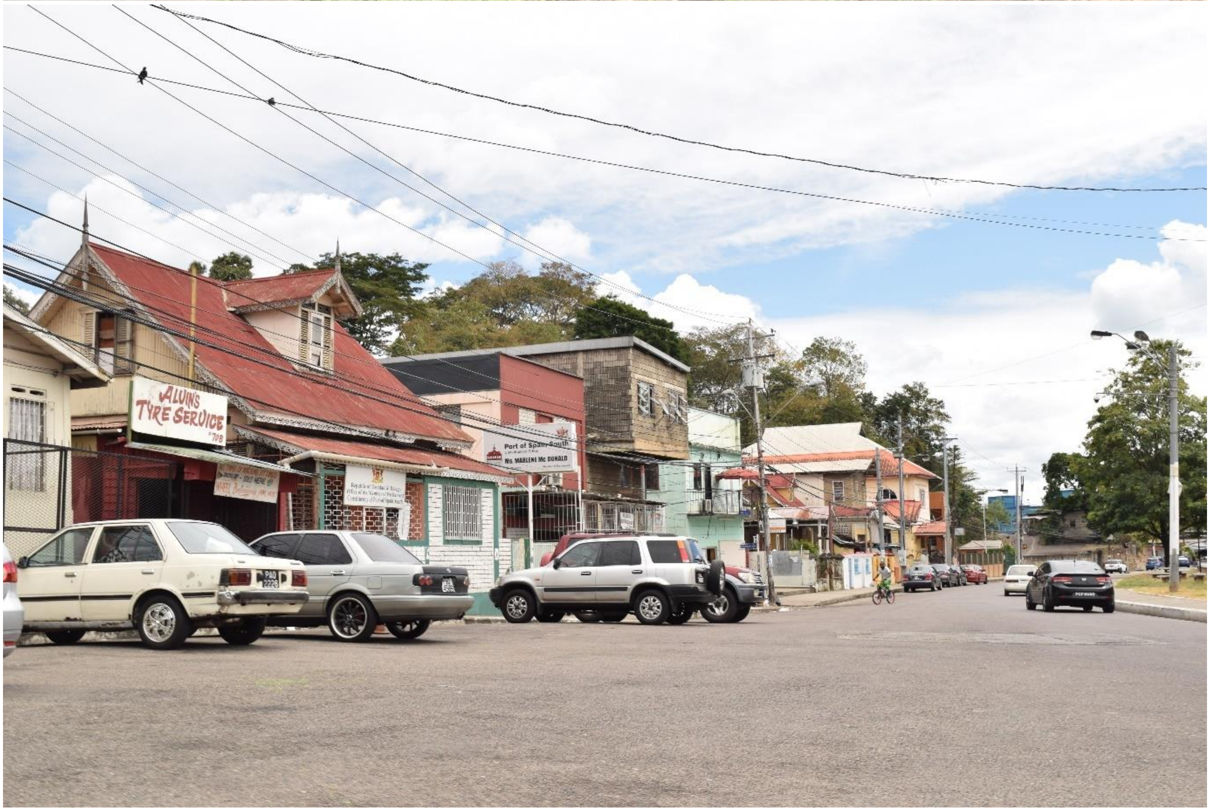
ALVIN'S
TYRE SERVICE
#70B
Ms. MARLENE Mc DONALD

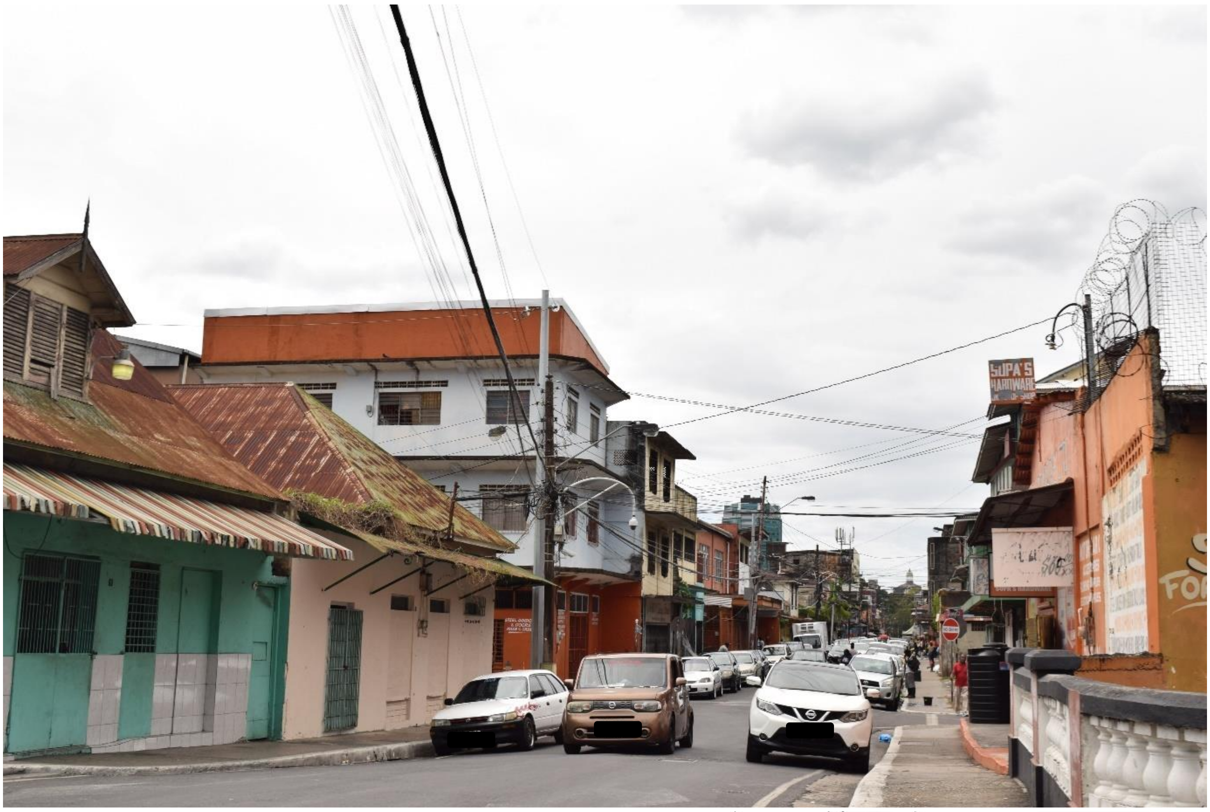
SUPA'S
HARDWARE

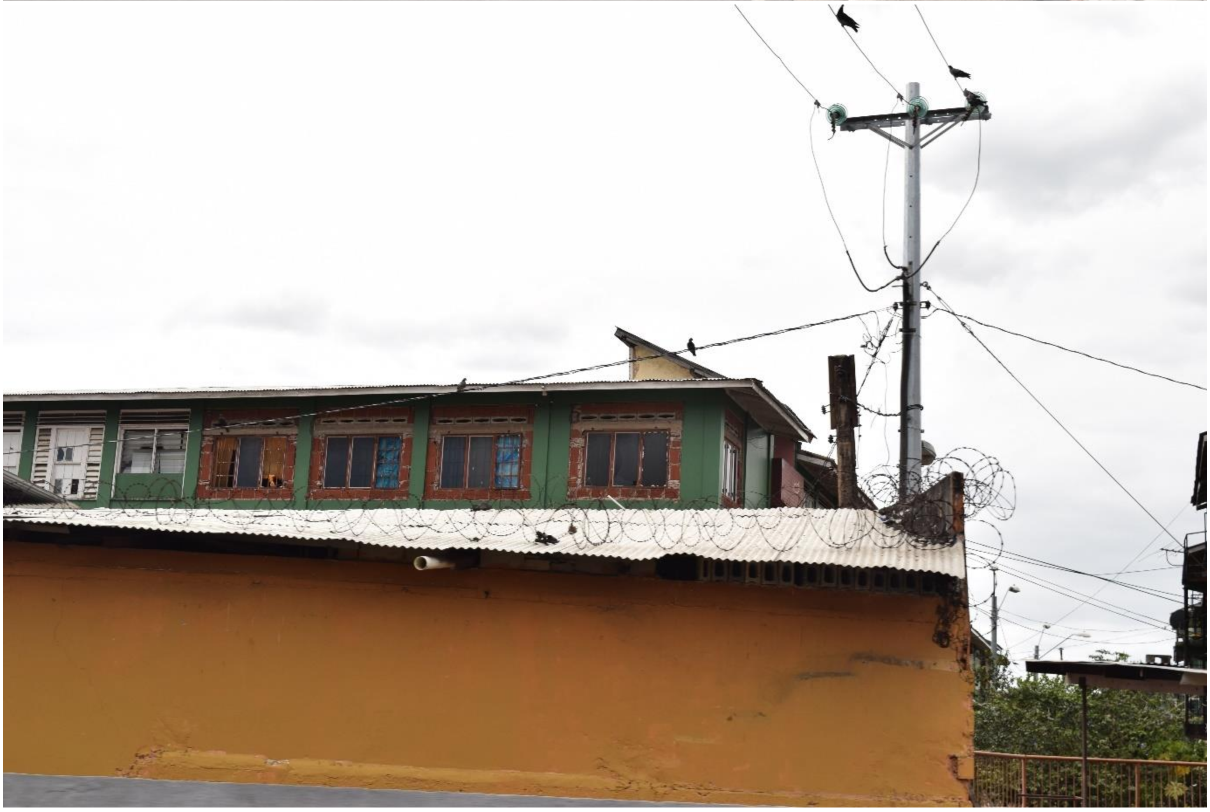

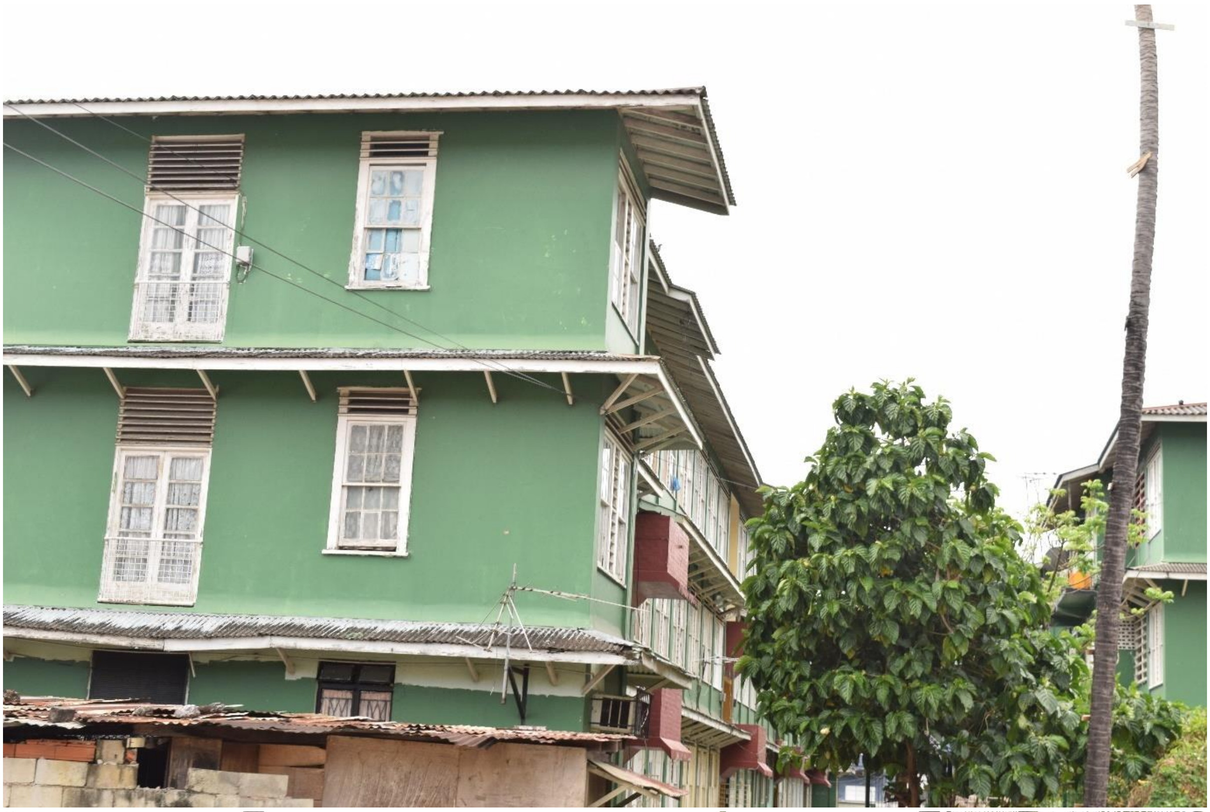

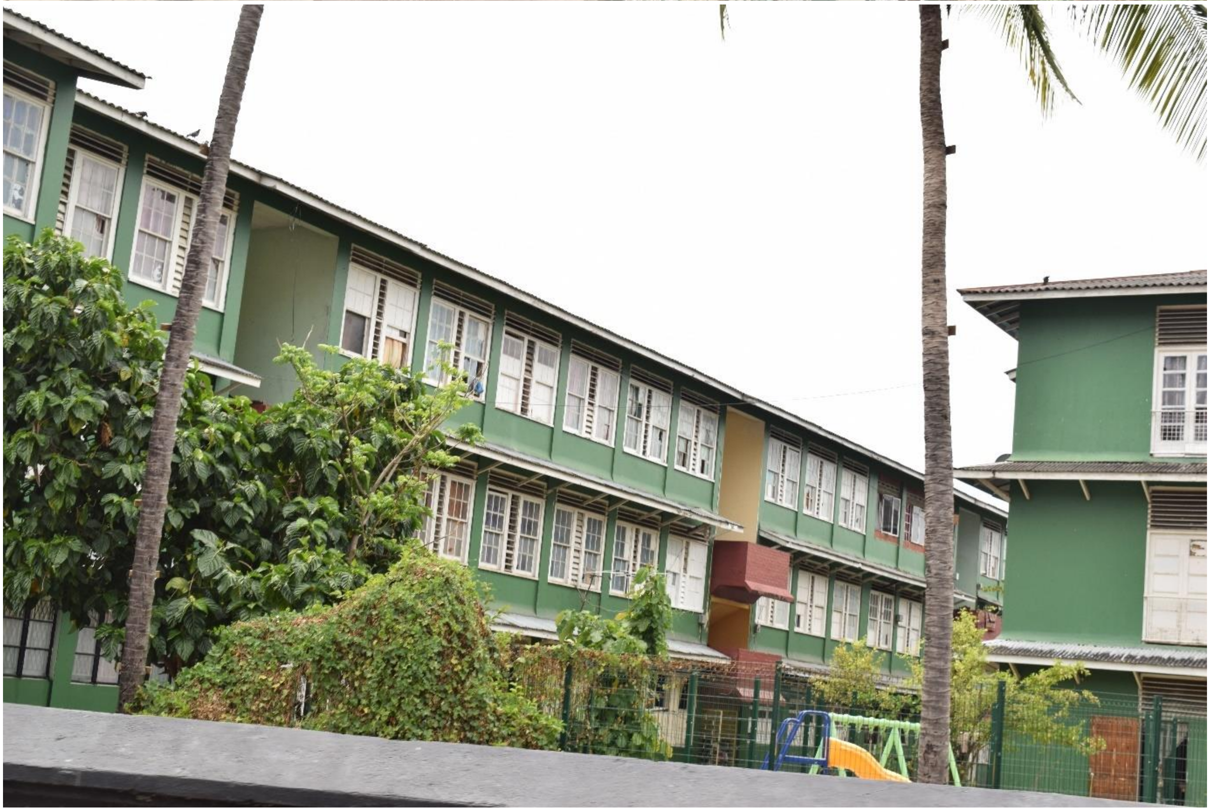

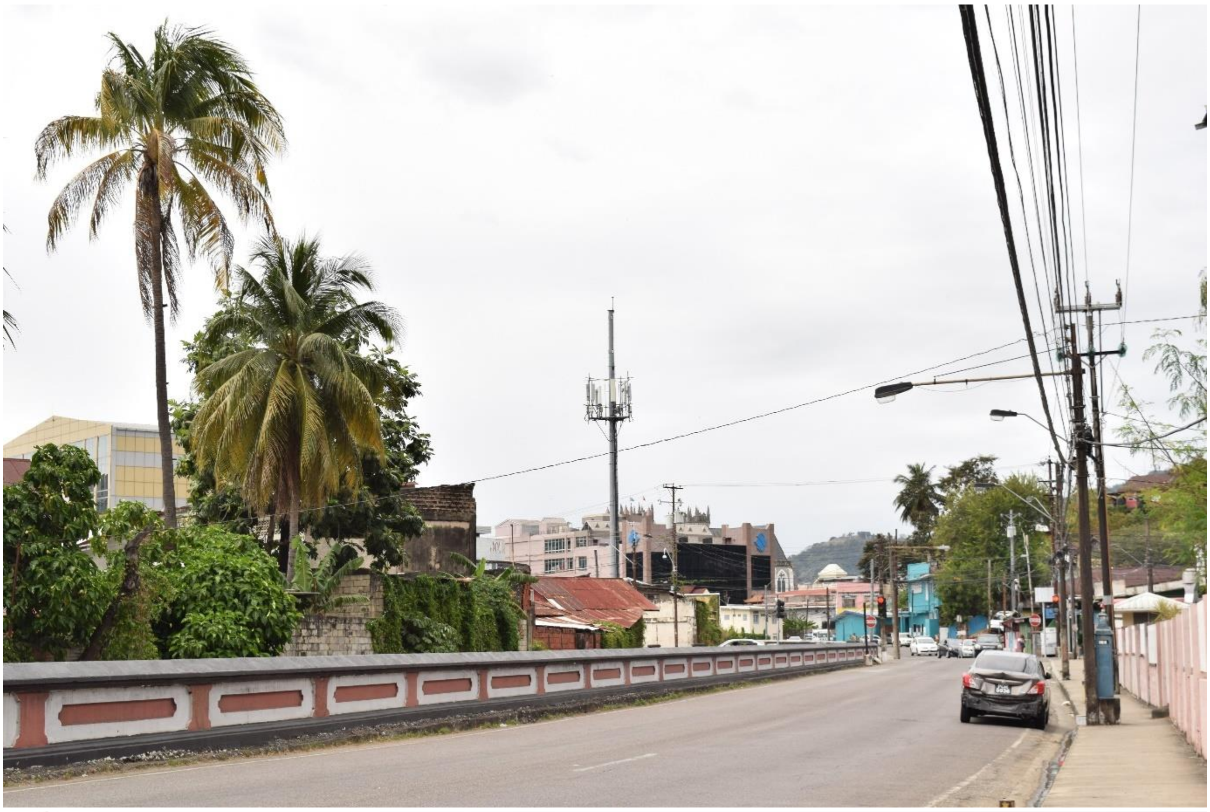

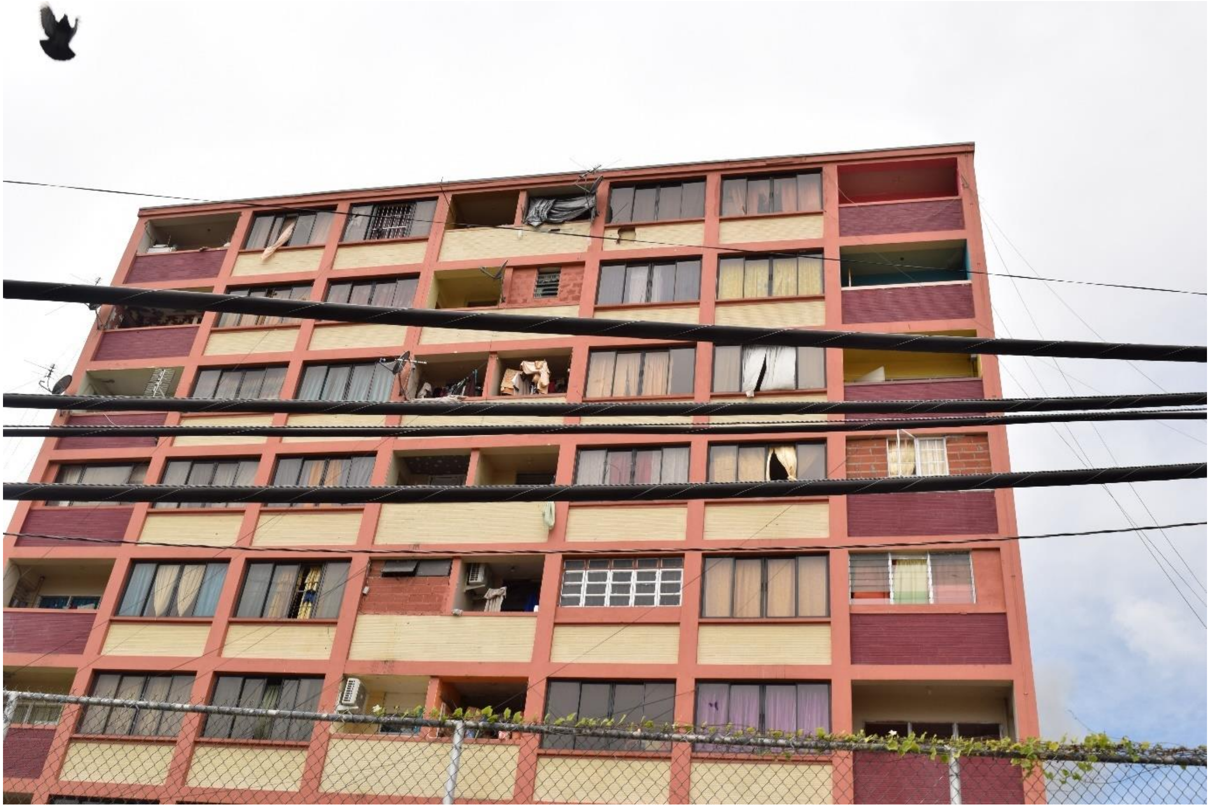

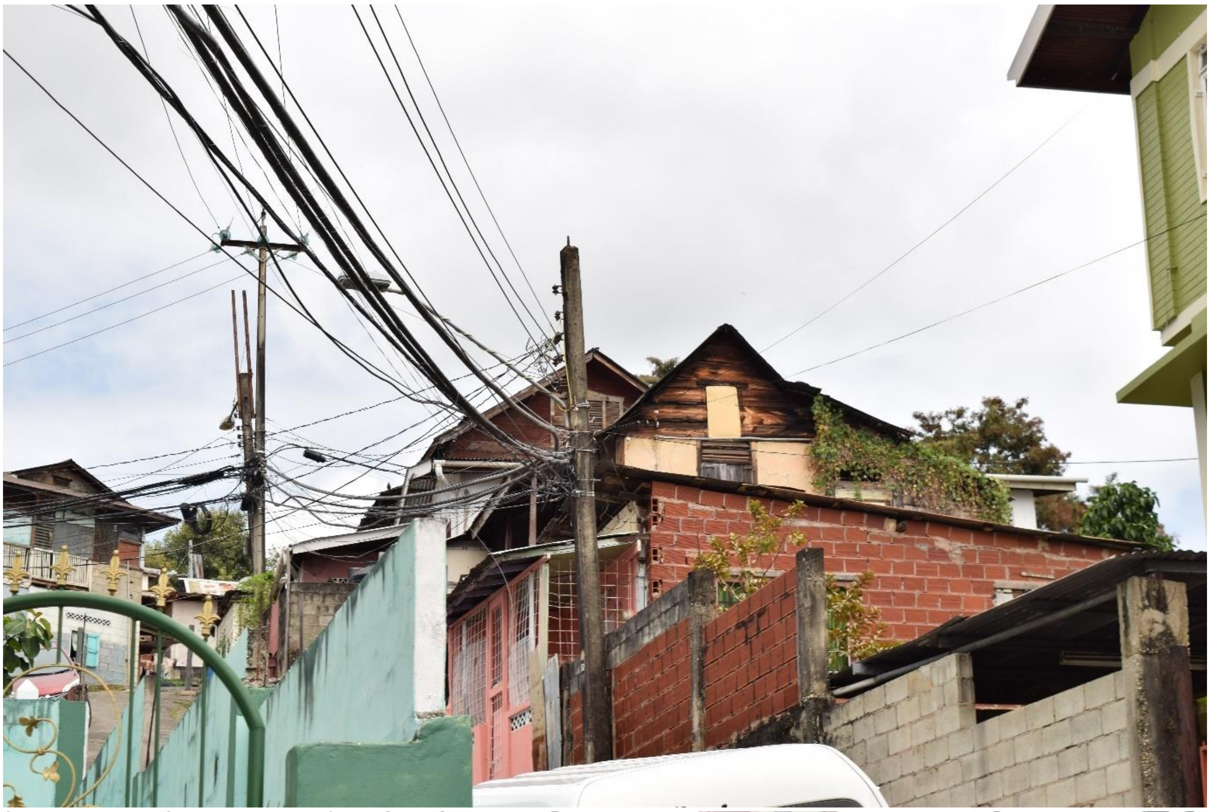

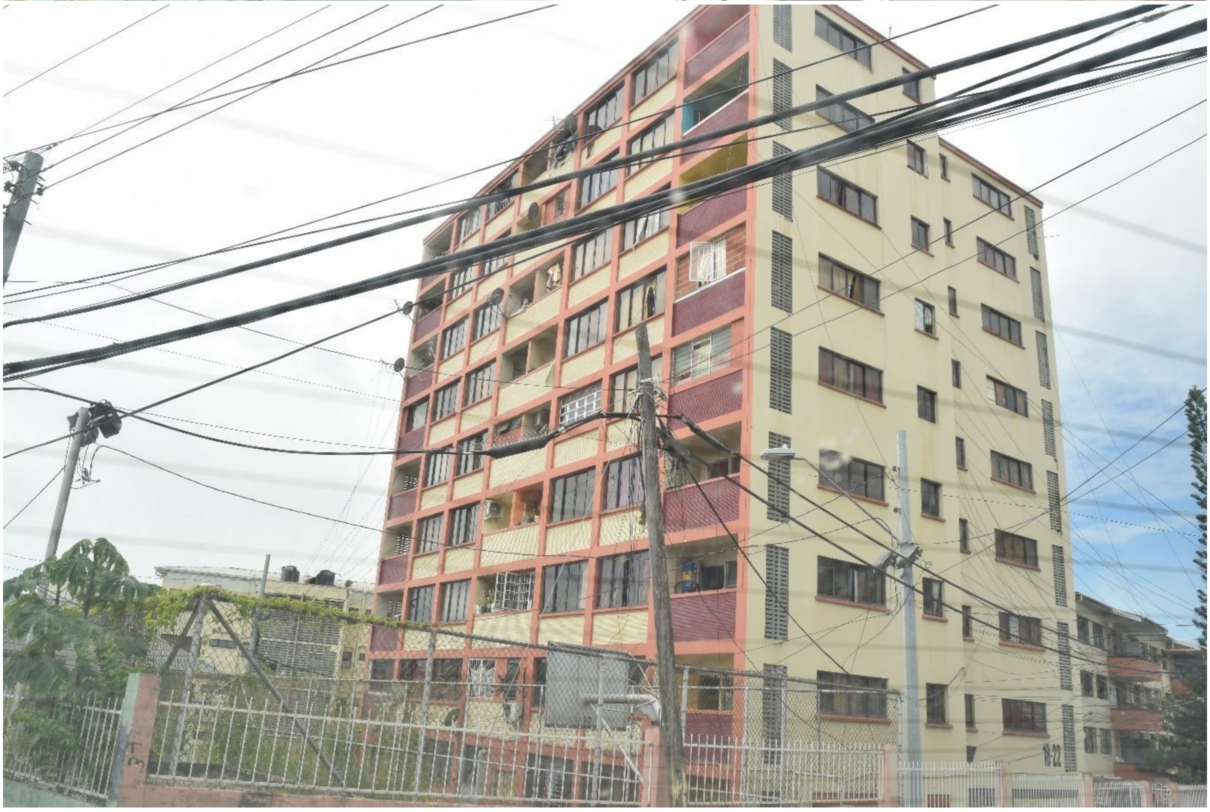

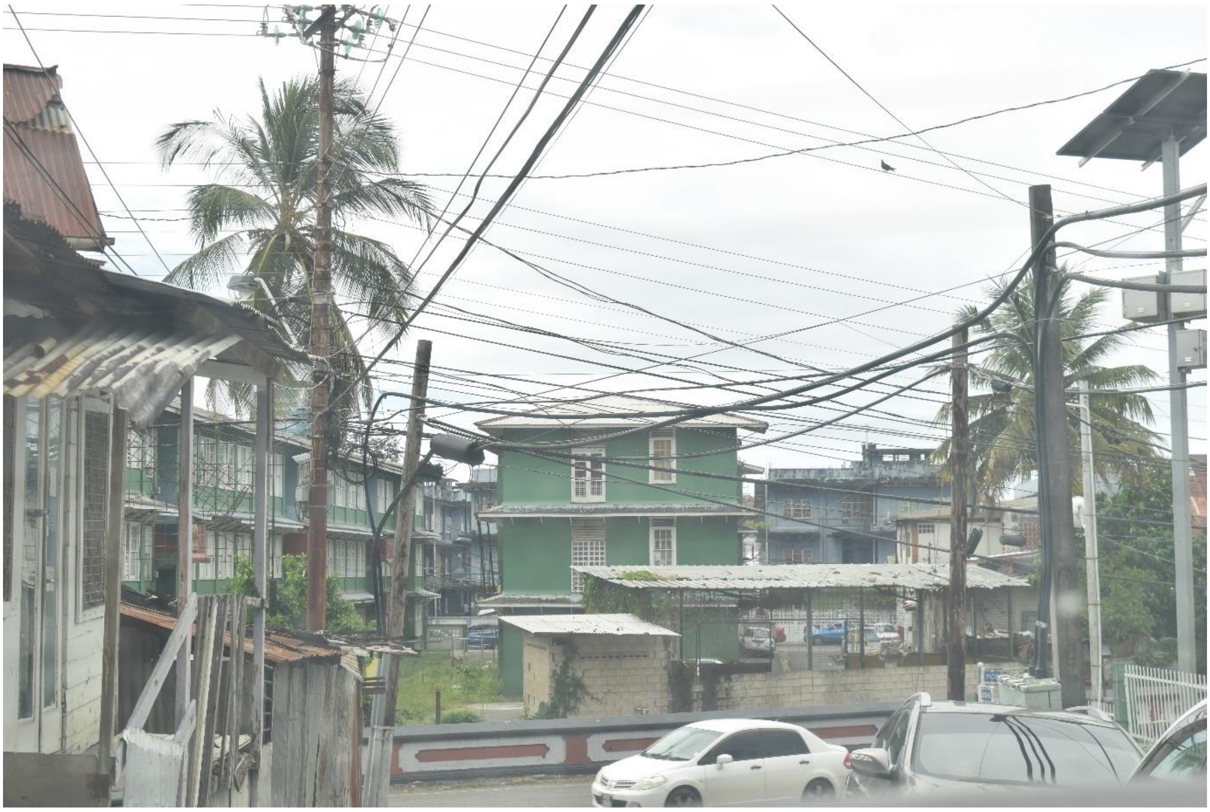

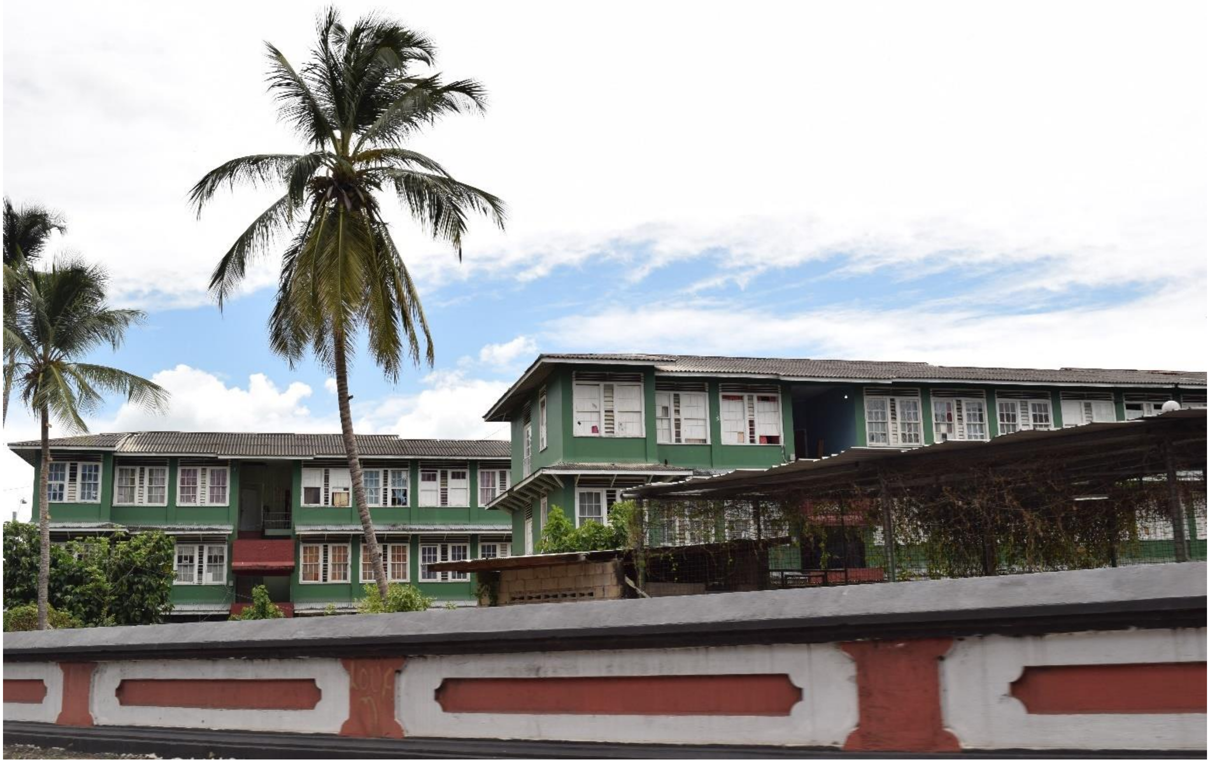

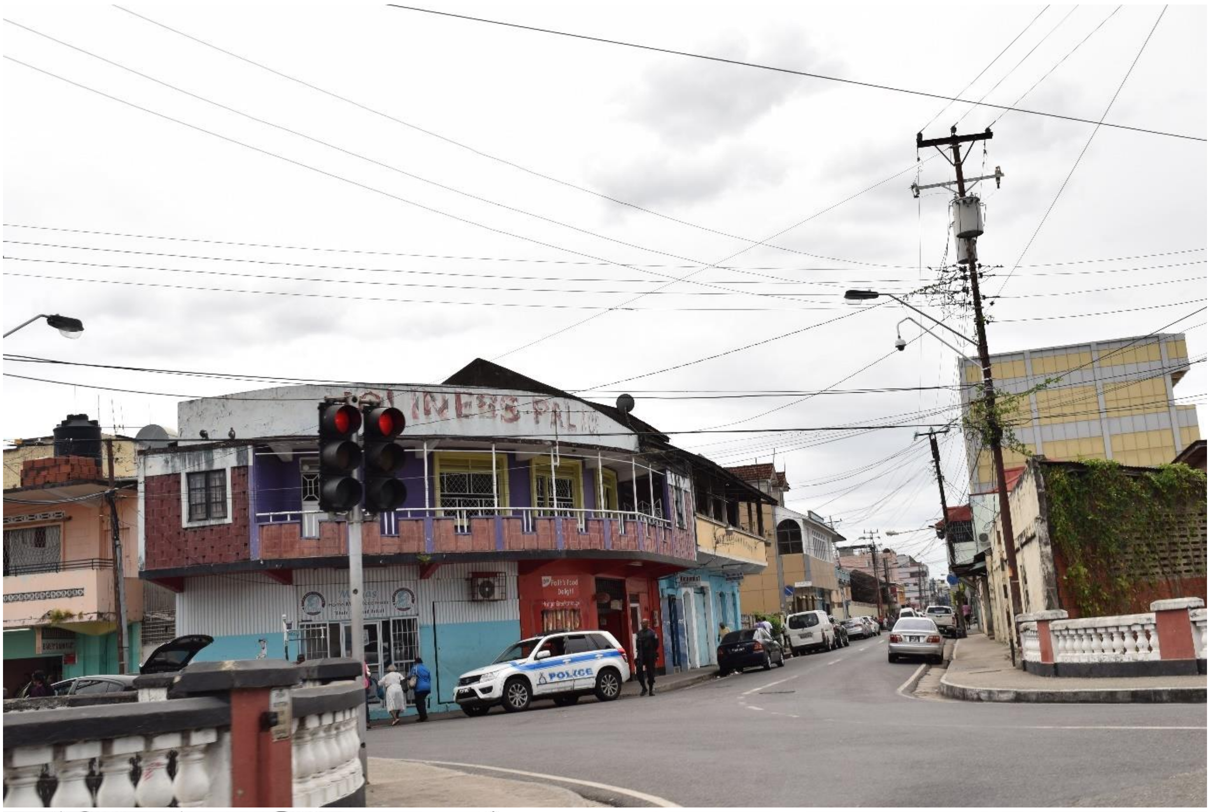
POLICE

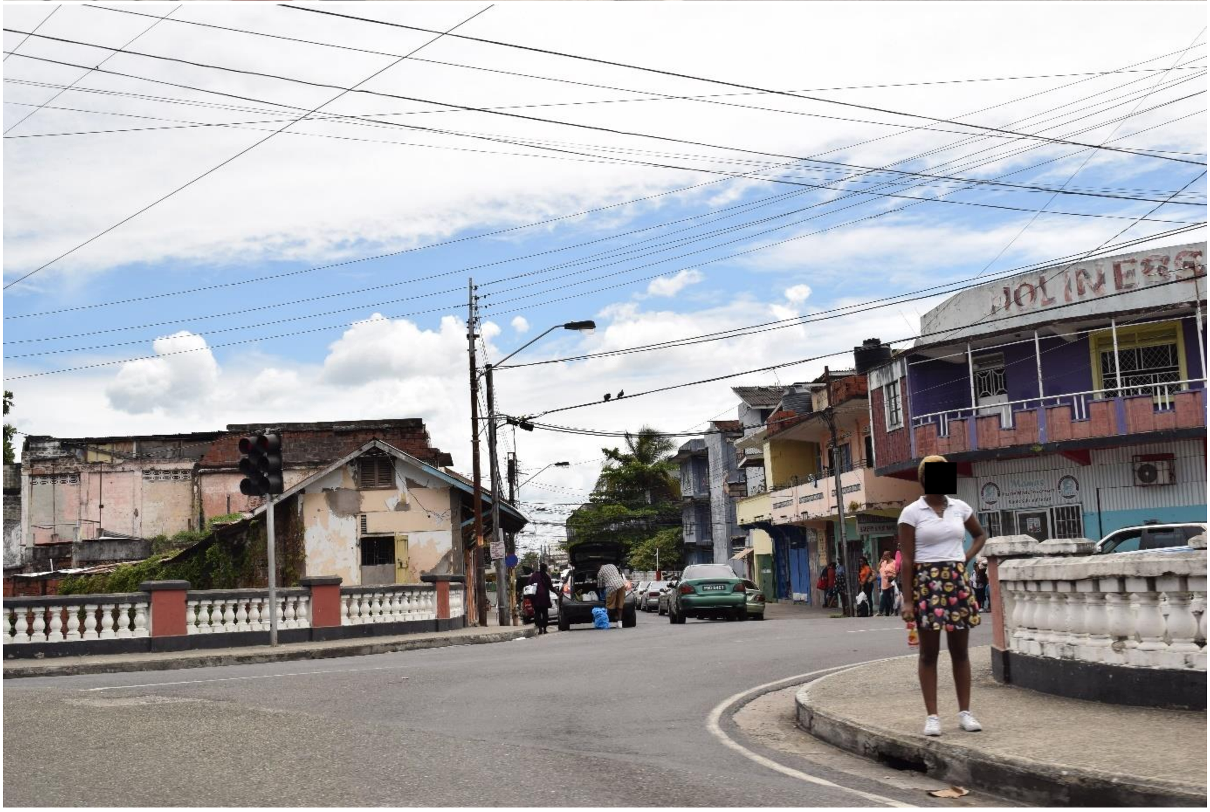

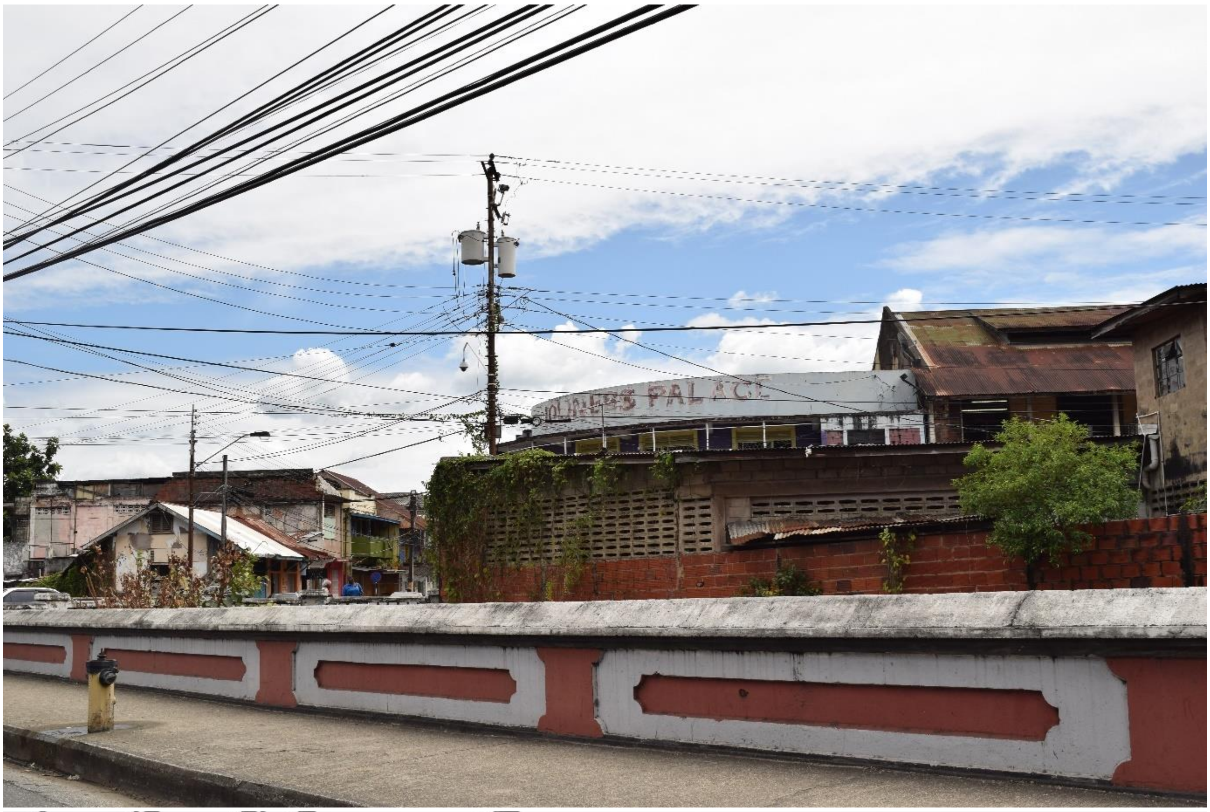
PALACE

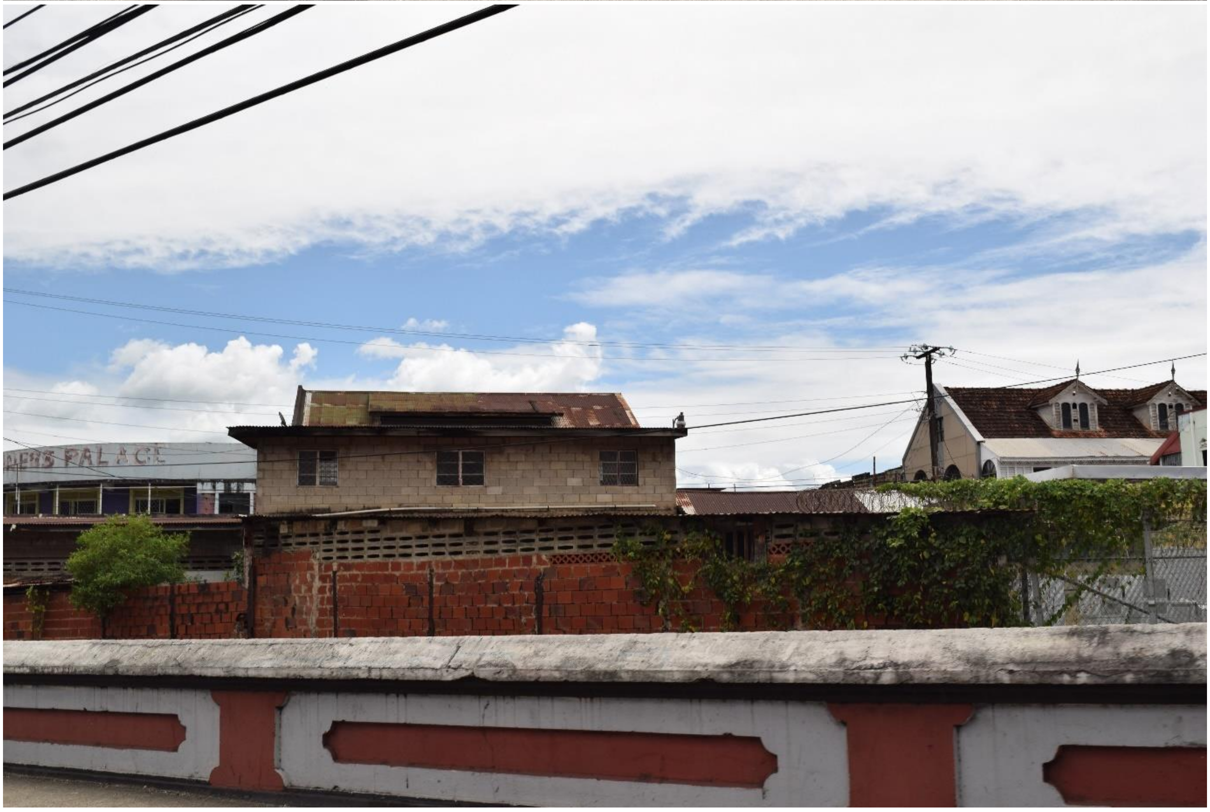

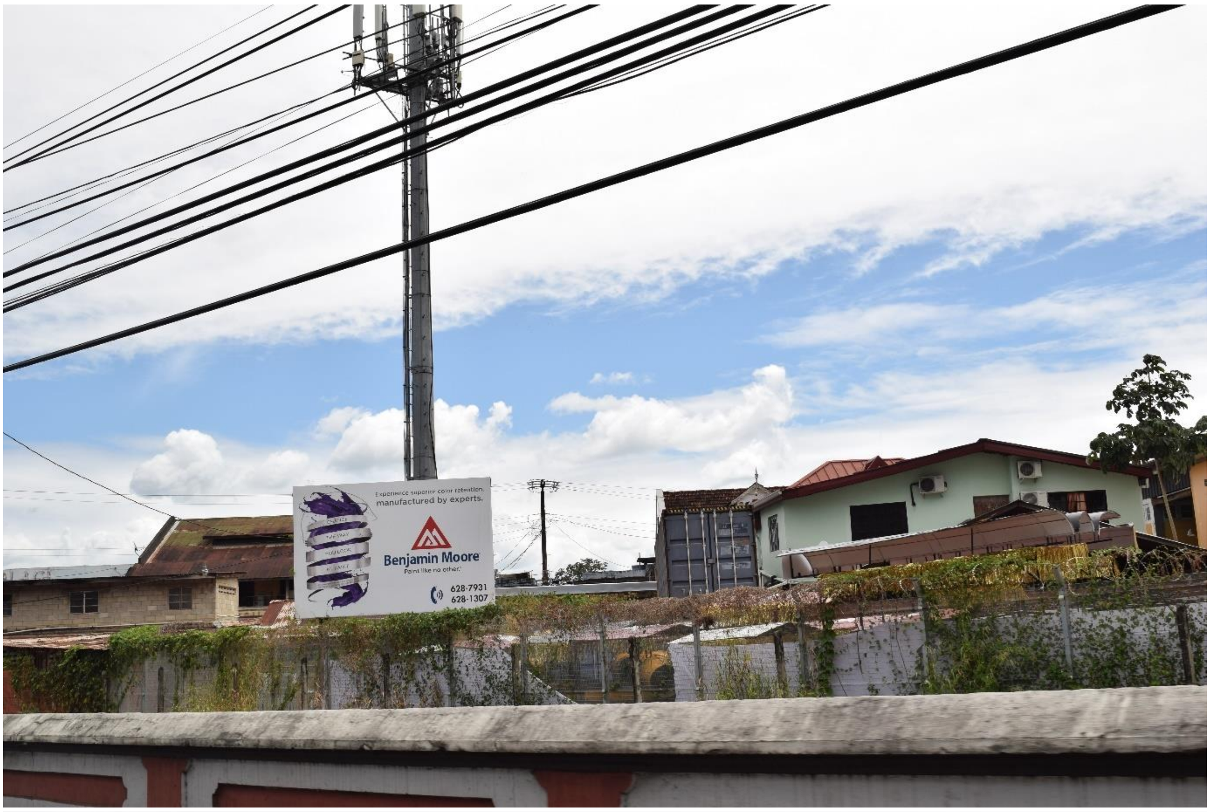
manufactured by experts.
Benjamin Moore
628-7931
628-1307

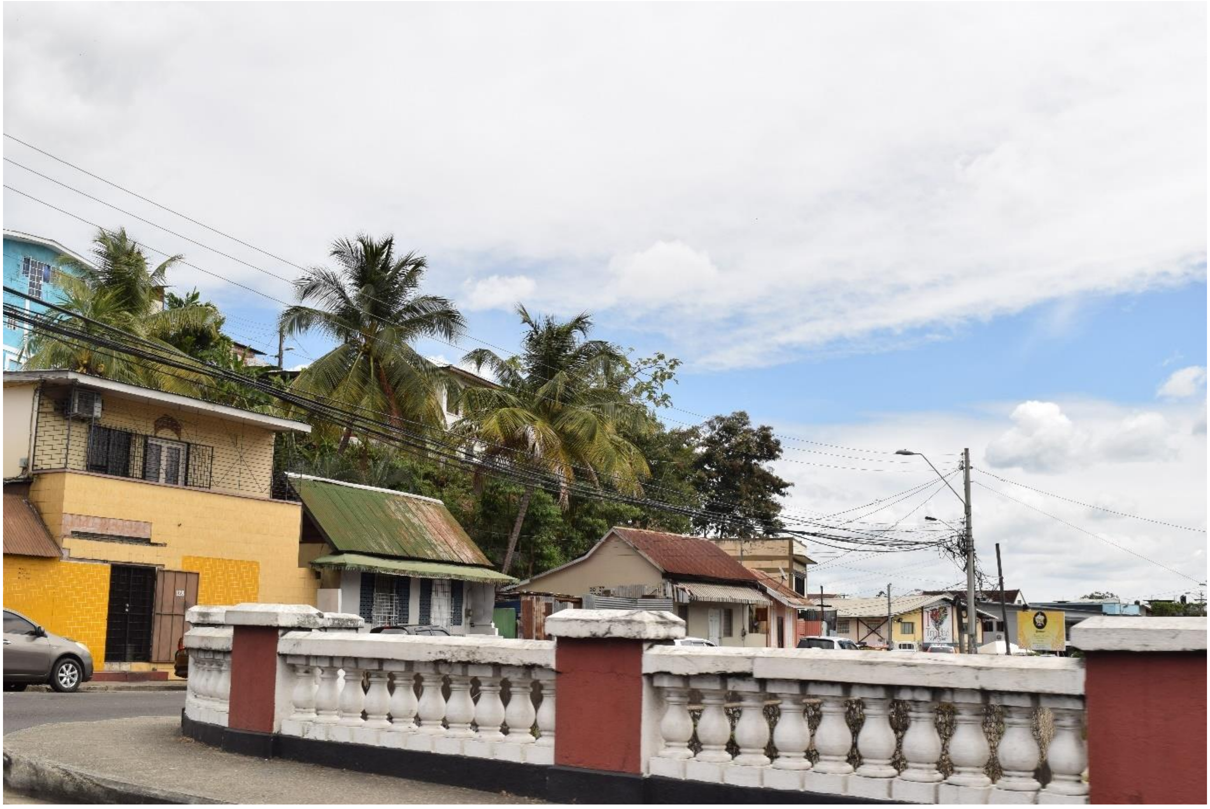

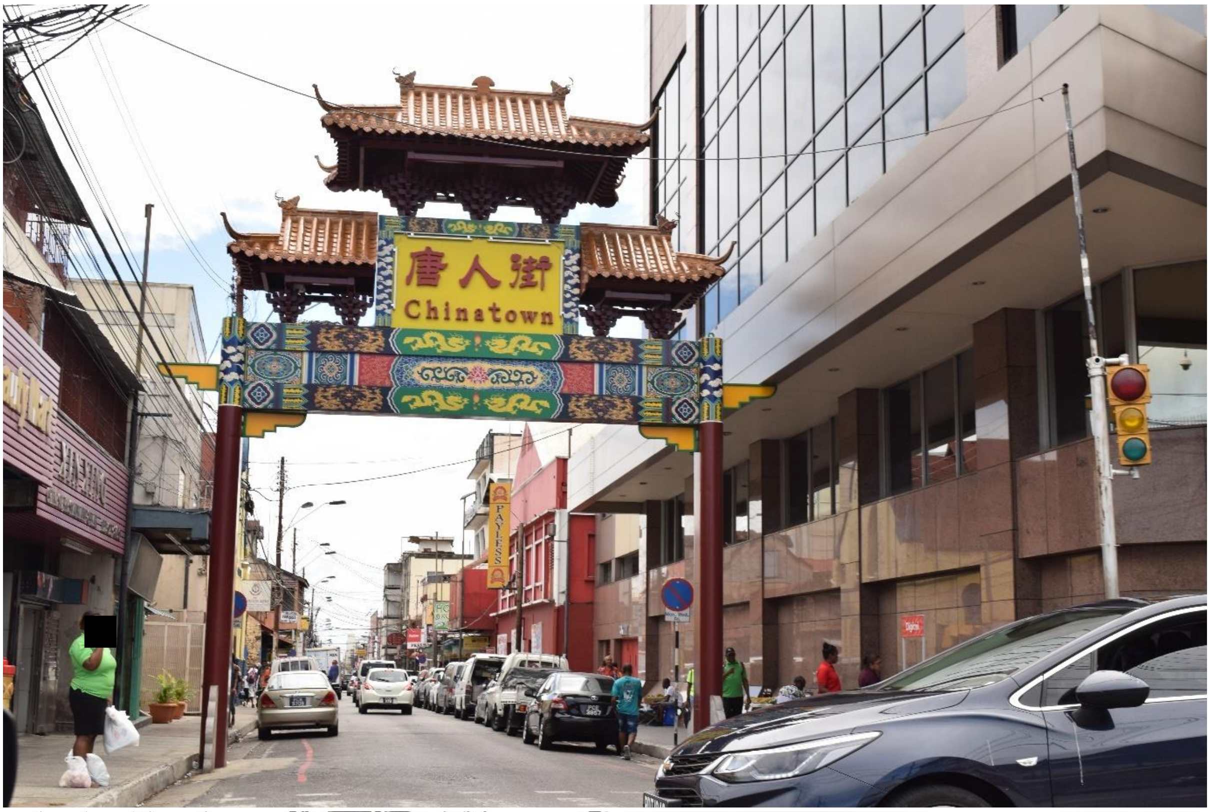
唐人街
Chinatown
PAYLESS

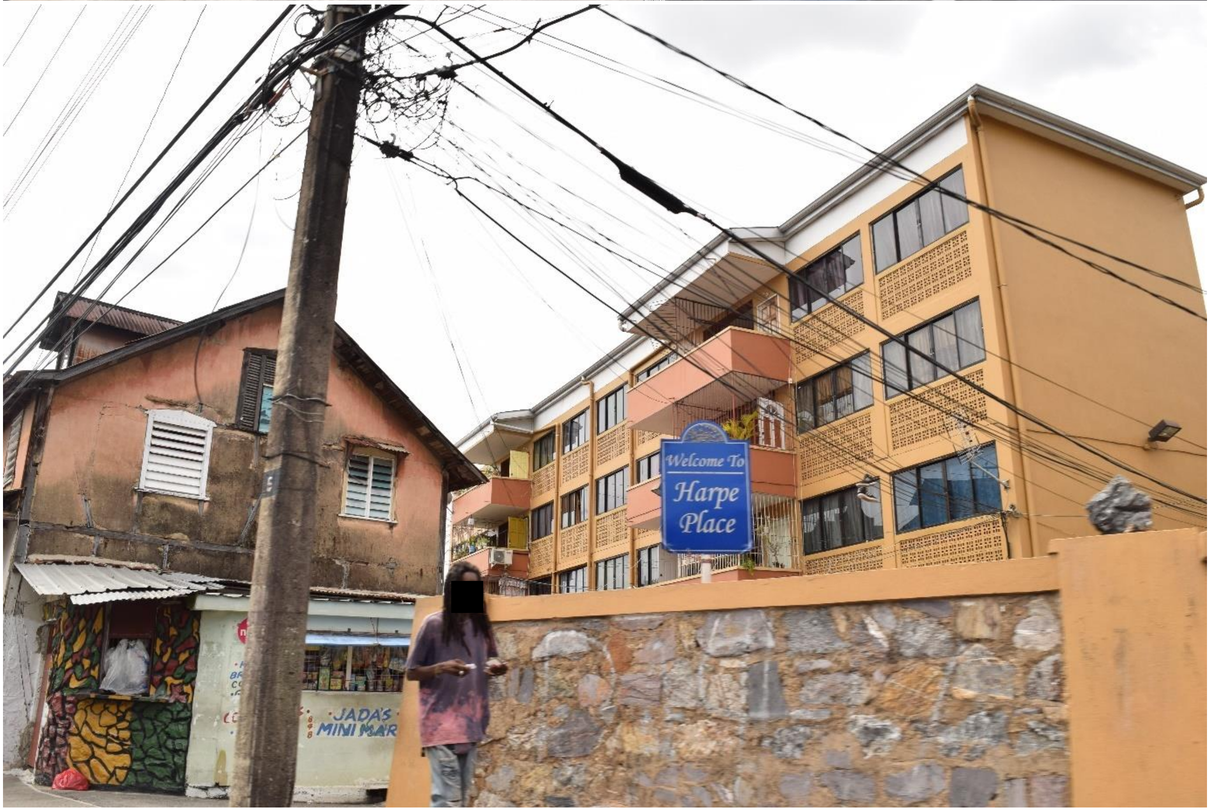
Welcome To
Harpe
Place
JADA'S
MINI MAR

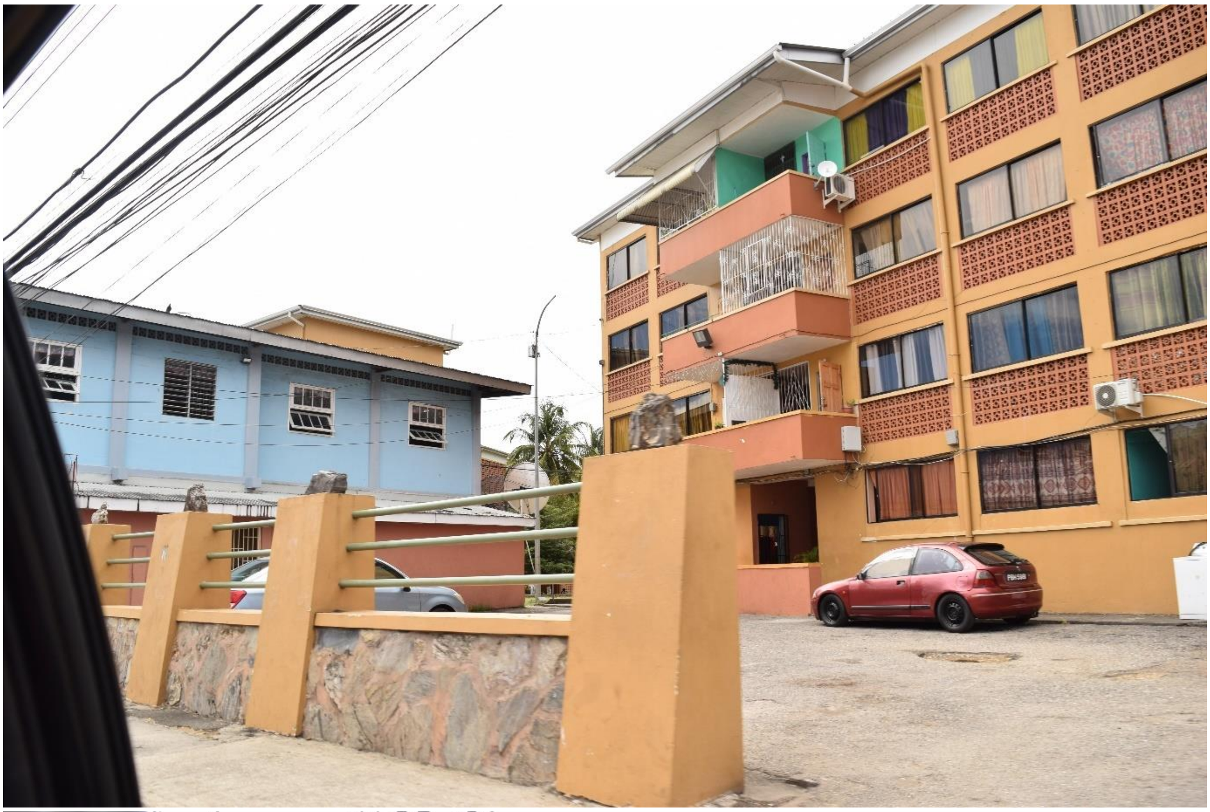

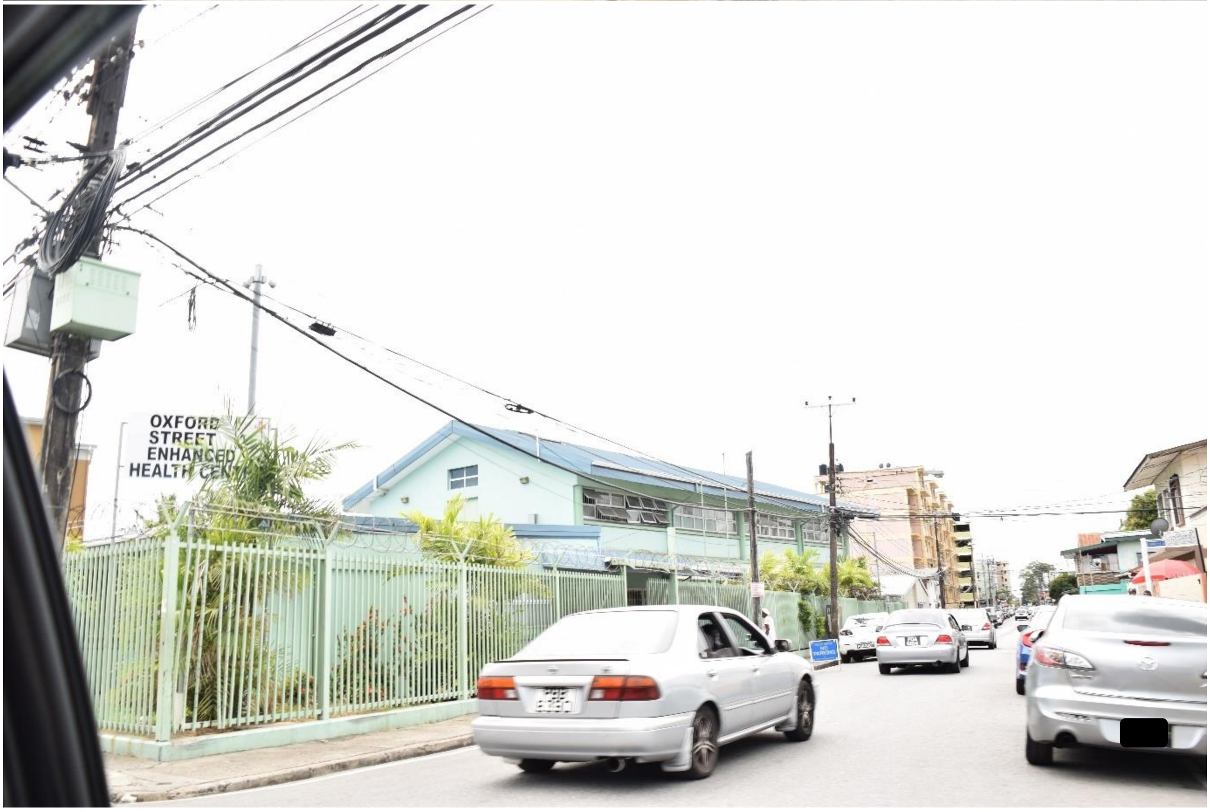
OXFORD
STREET
ENHANCED

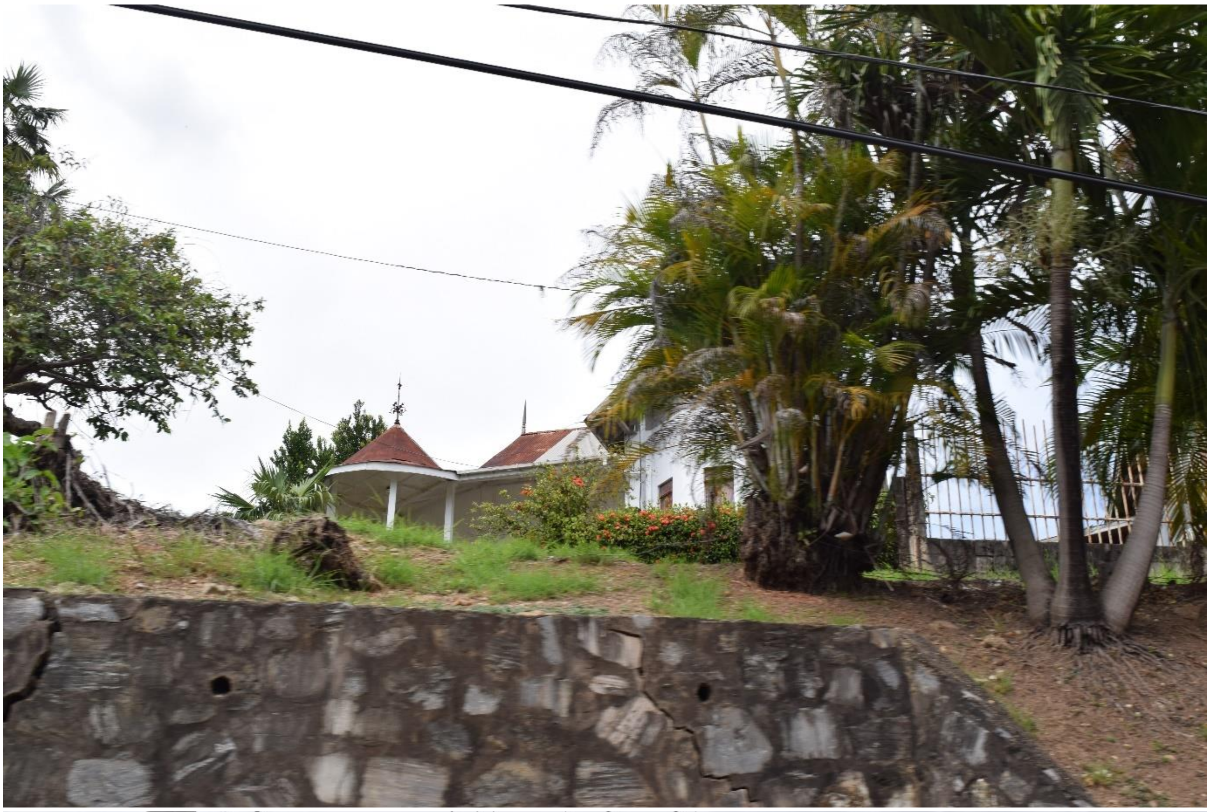

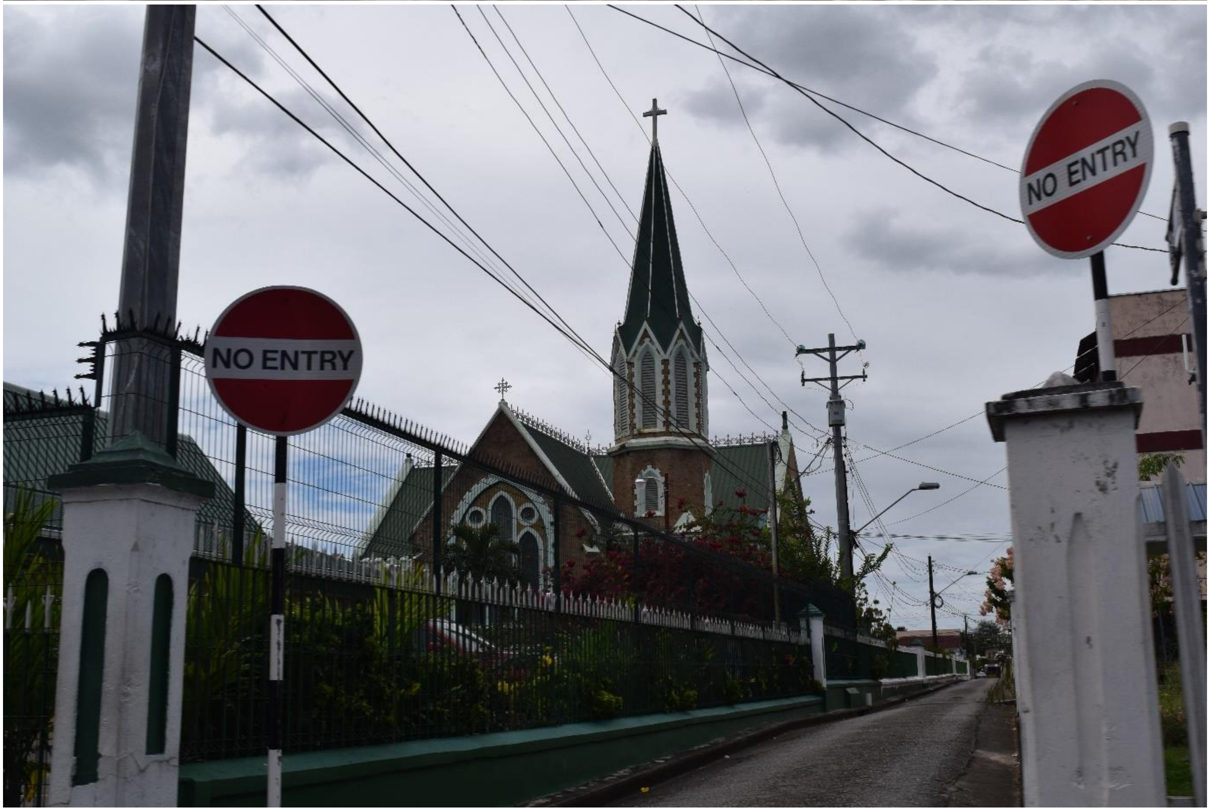
NO ENTRY
NO ENTRY

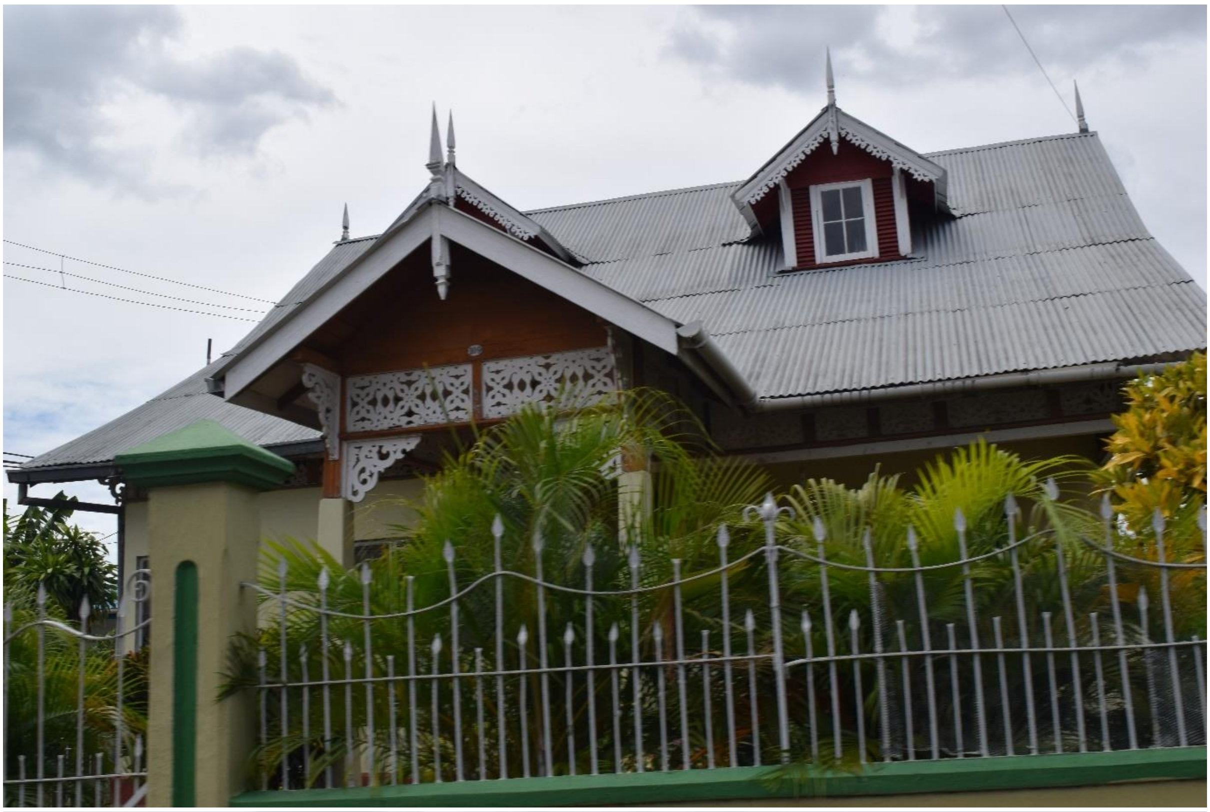

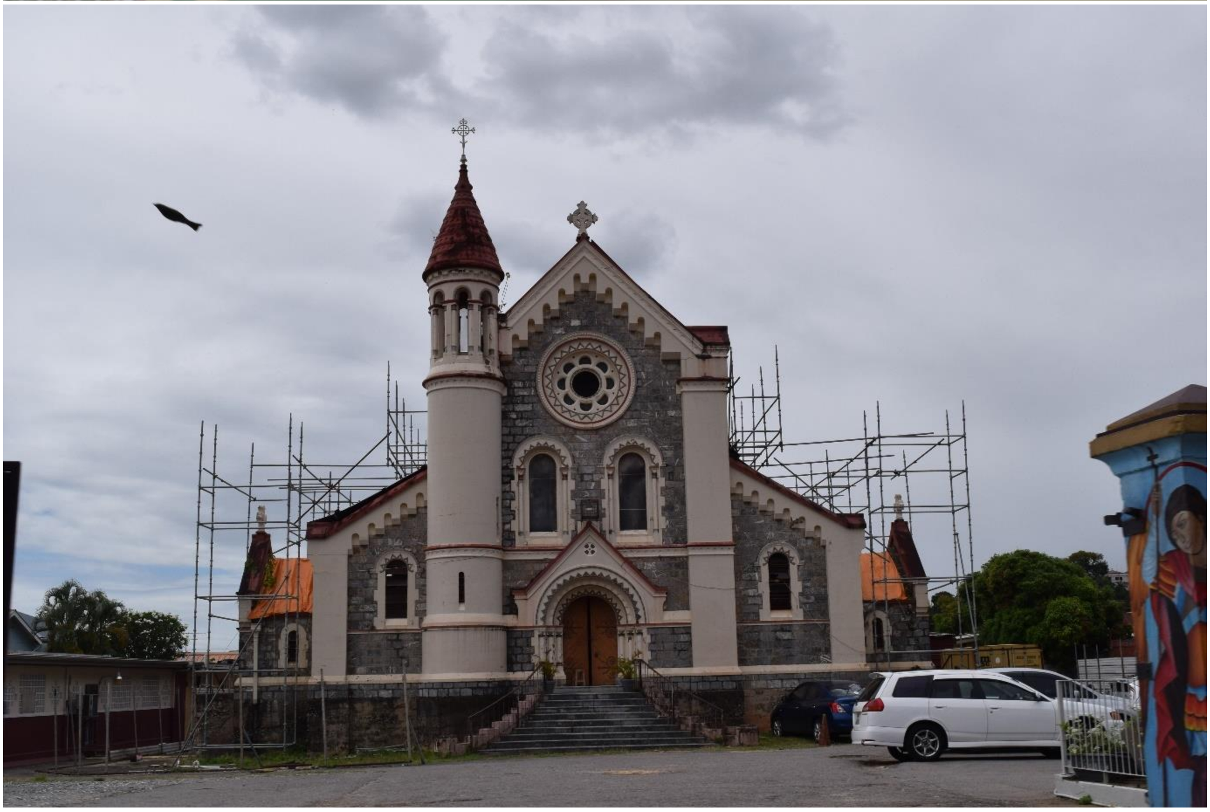

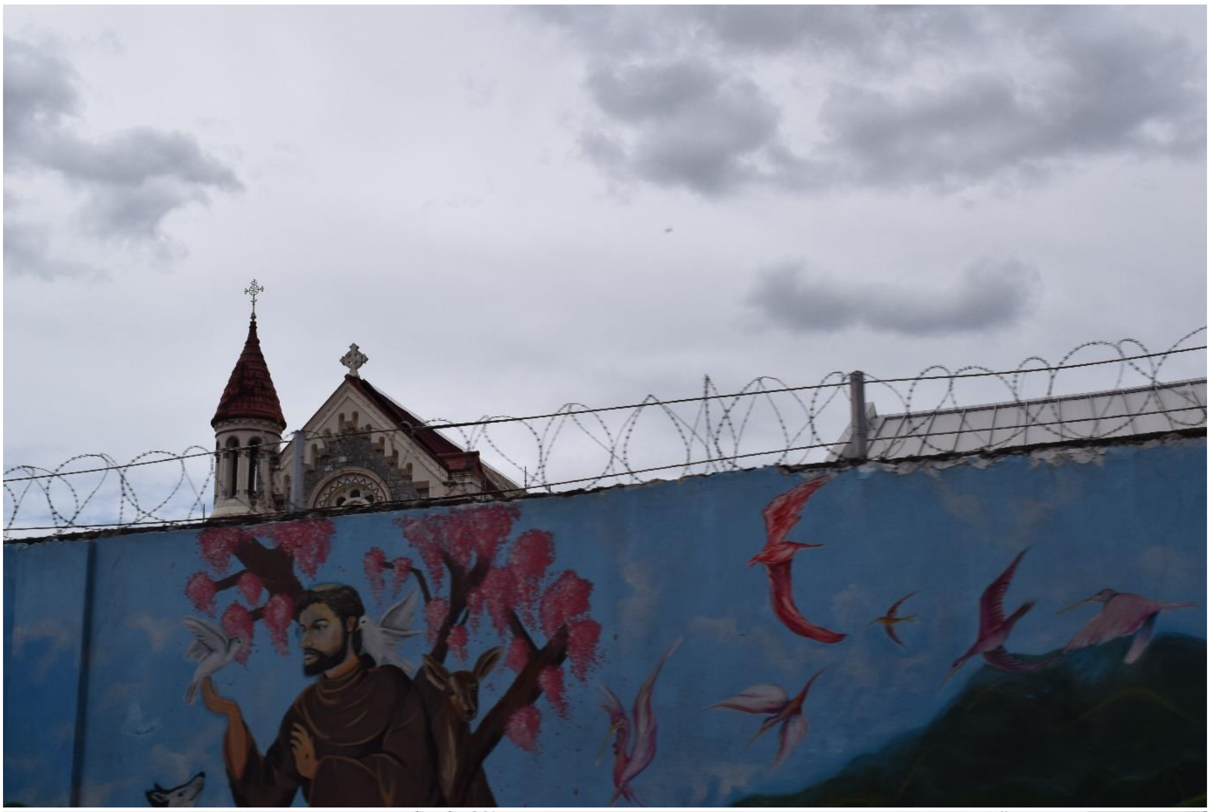

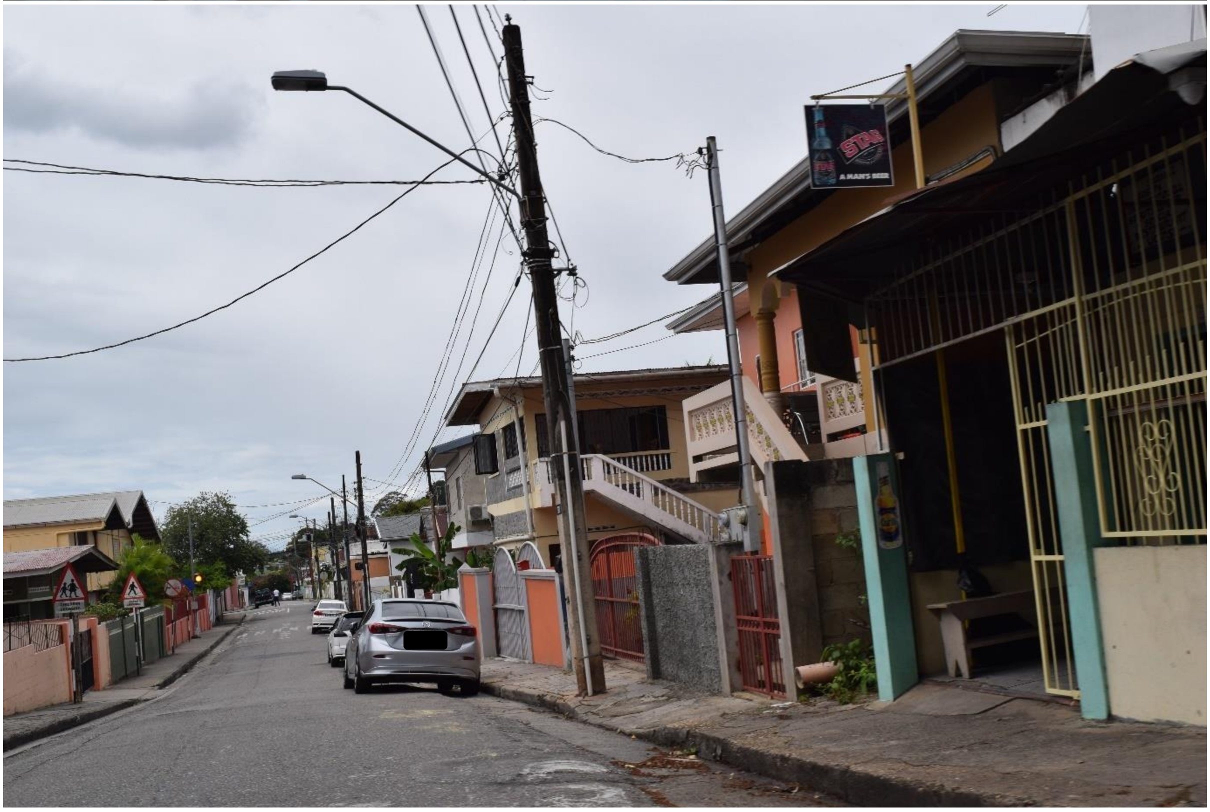
A MAN'S BEER

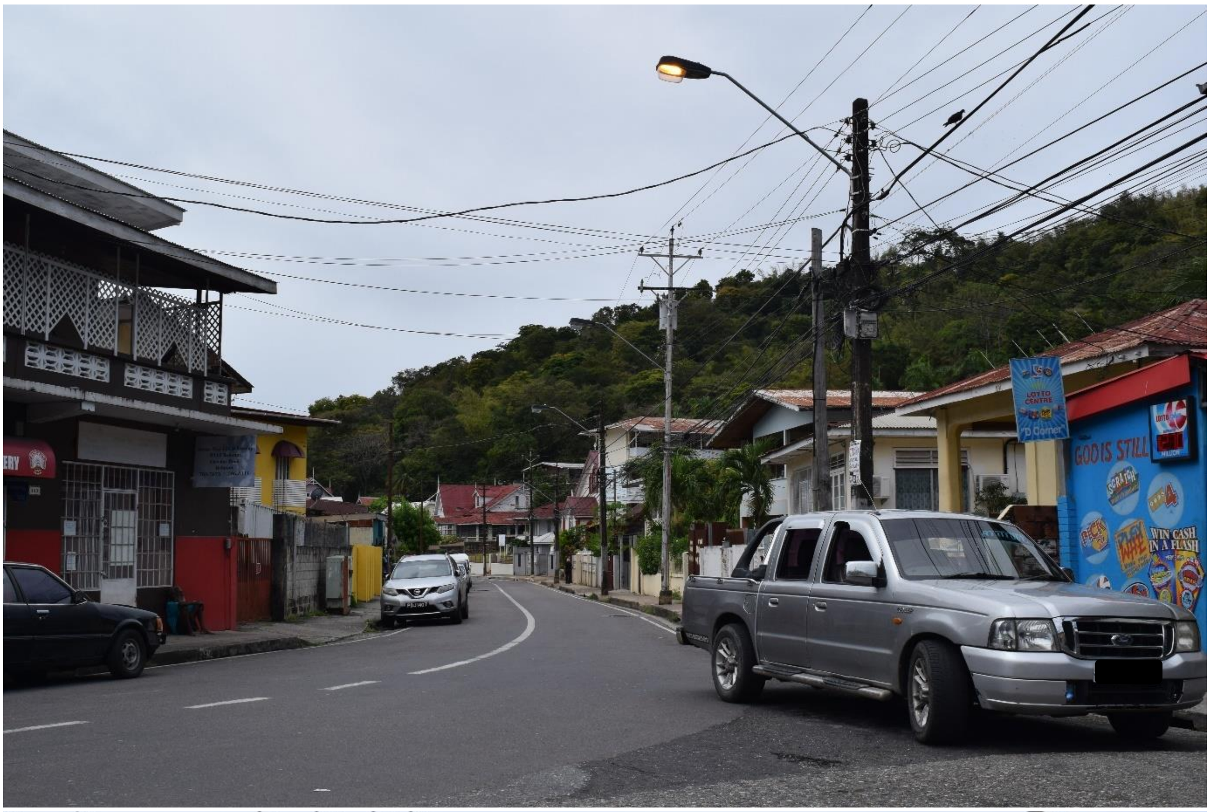
LOTTO CENTRE
GODS STILL
WIN CASH IN A FLASH

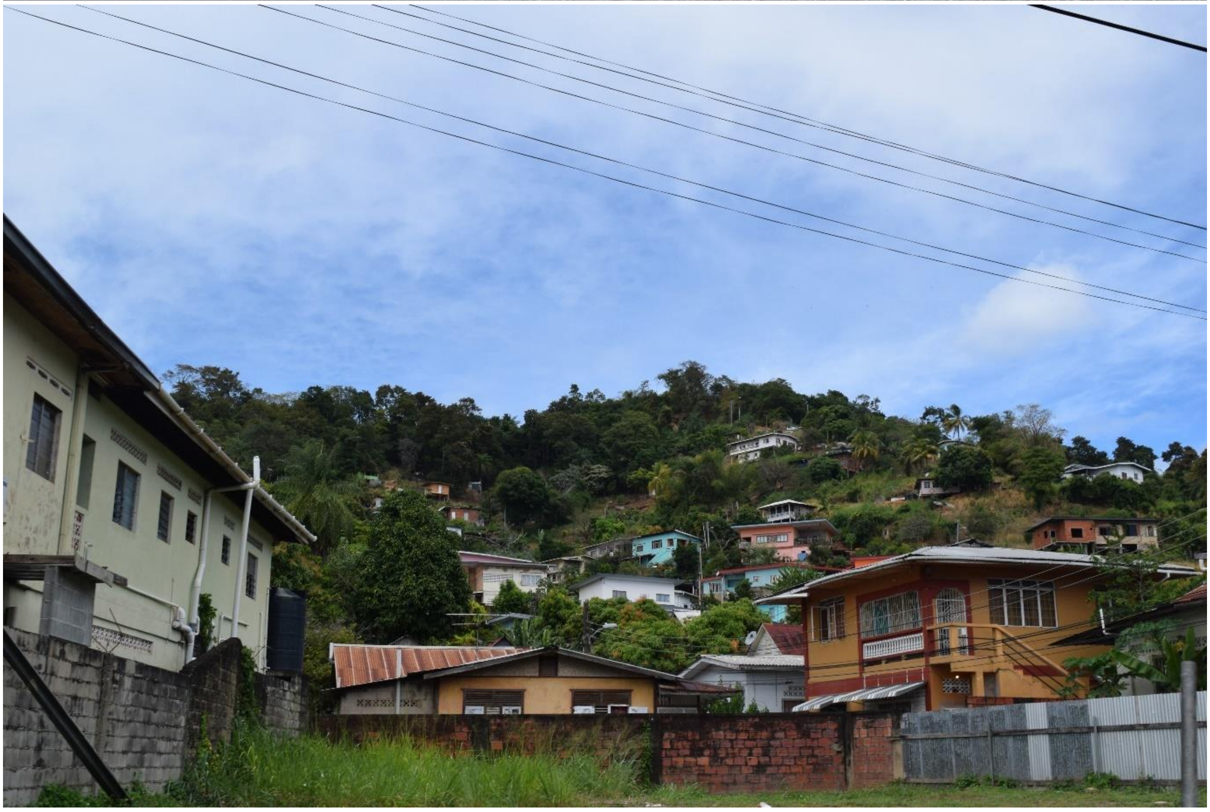

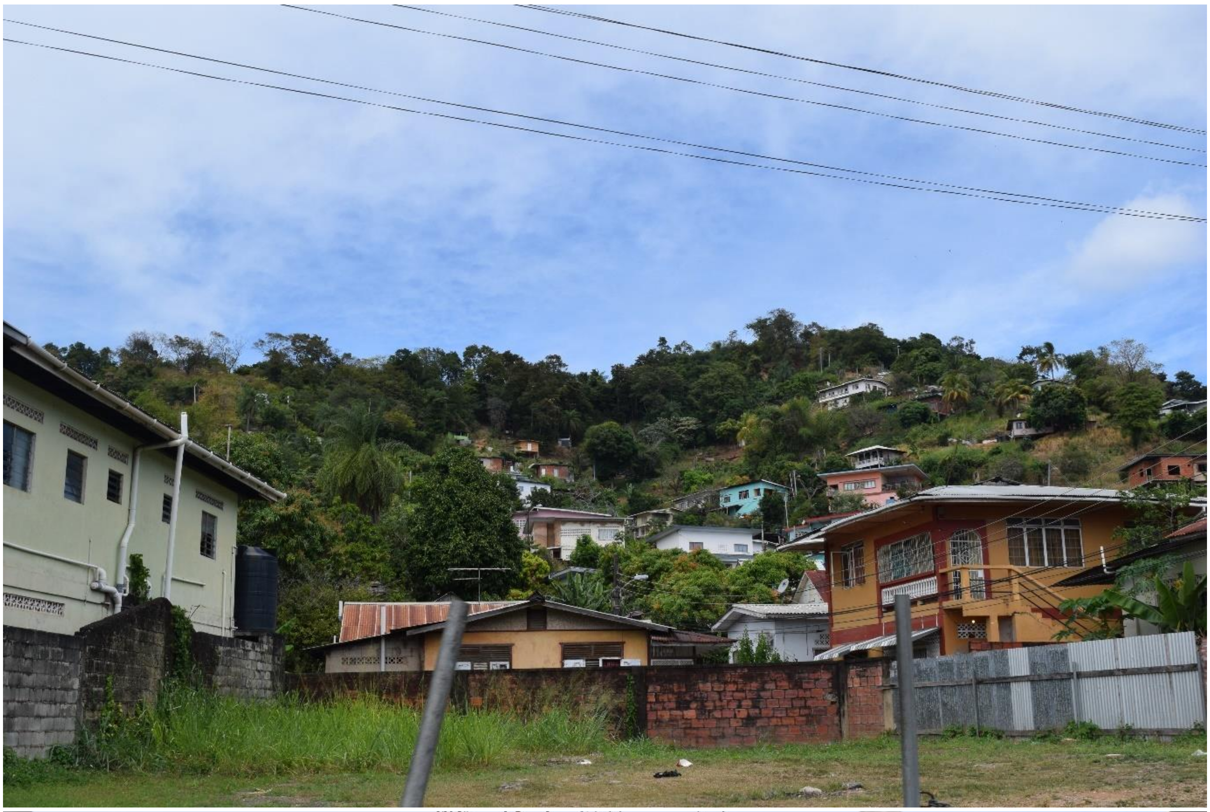

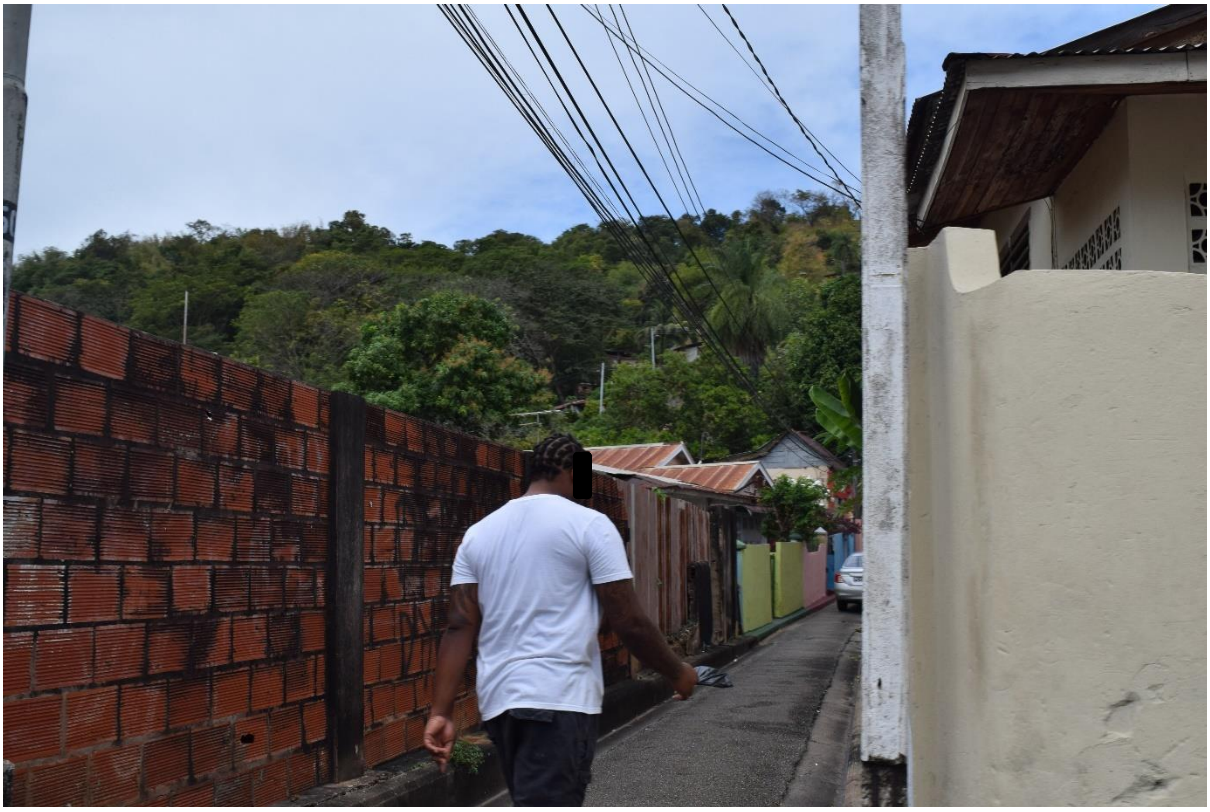

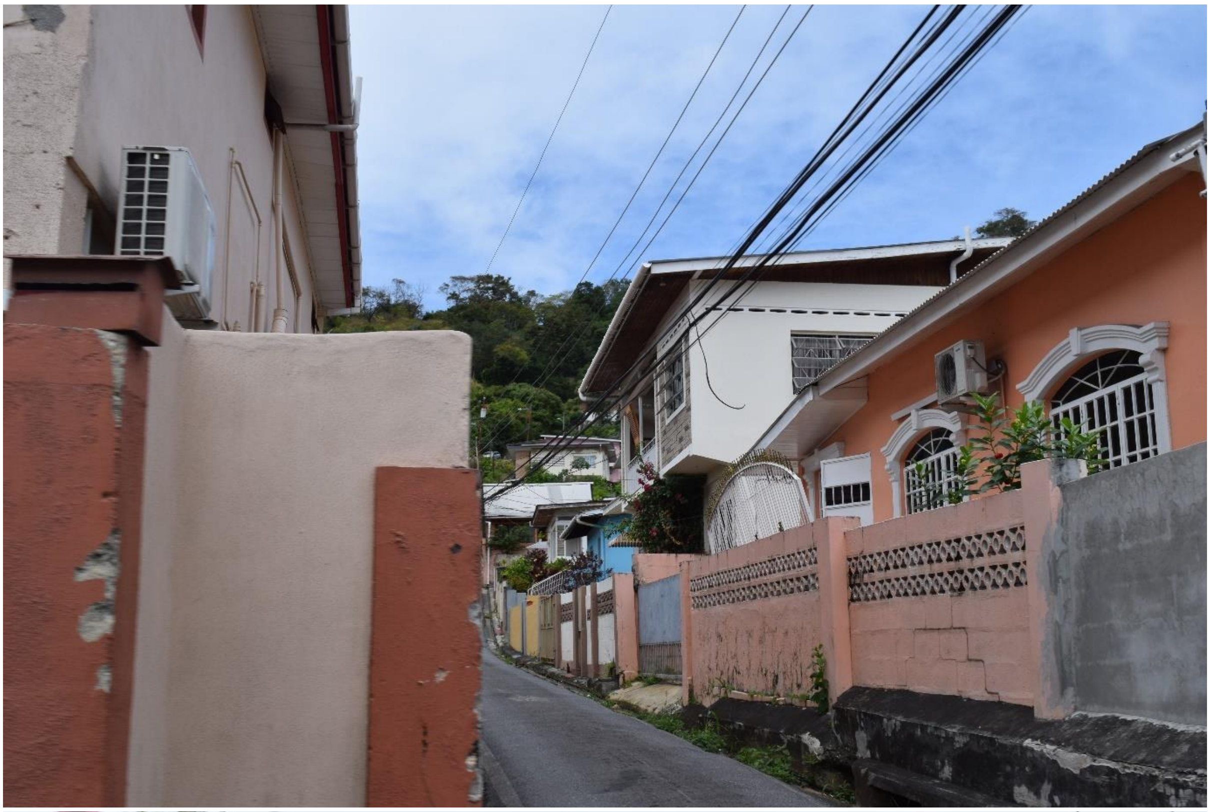

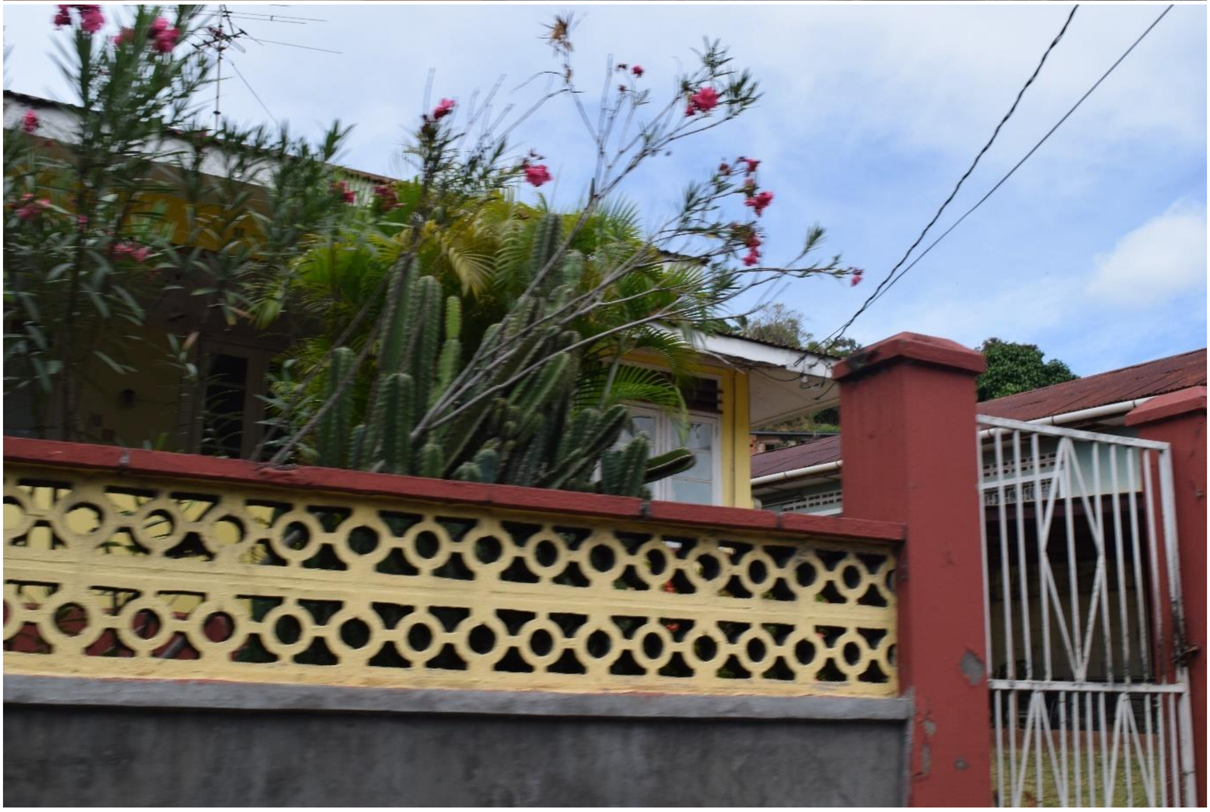

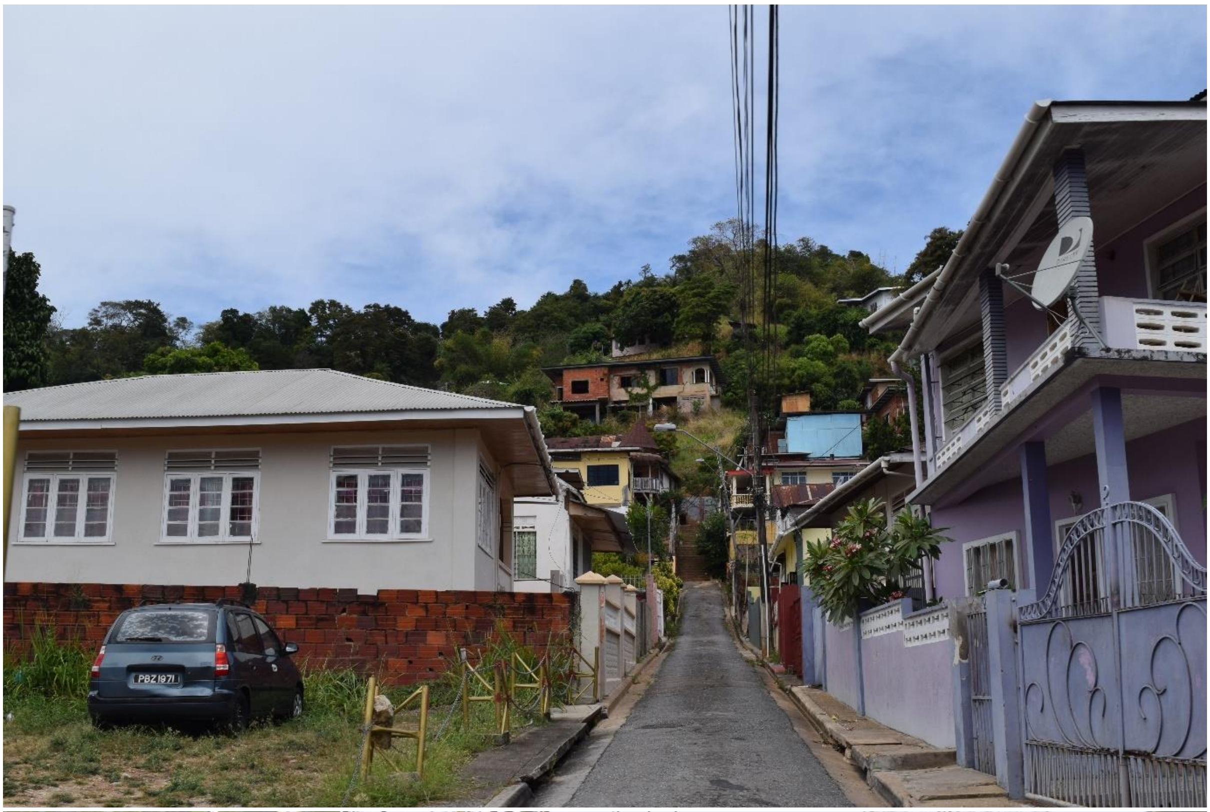
PBZ 1971

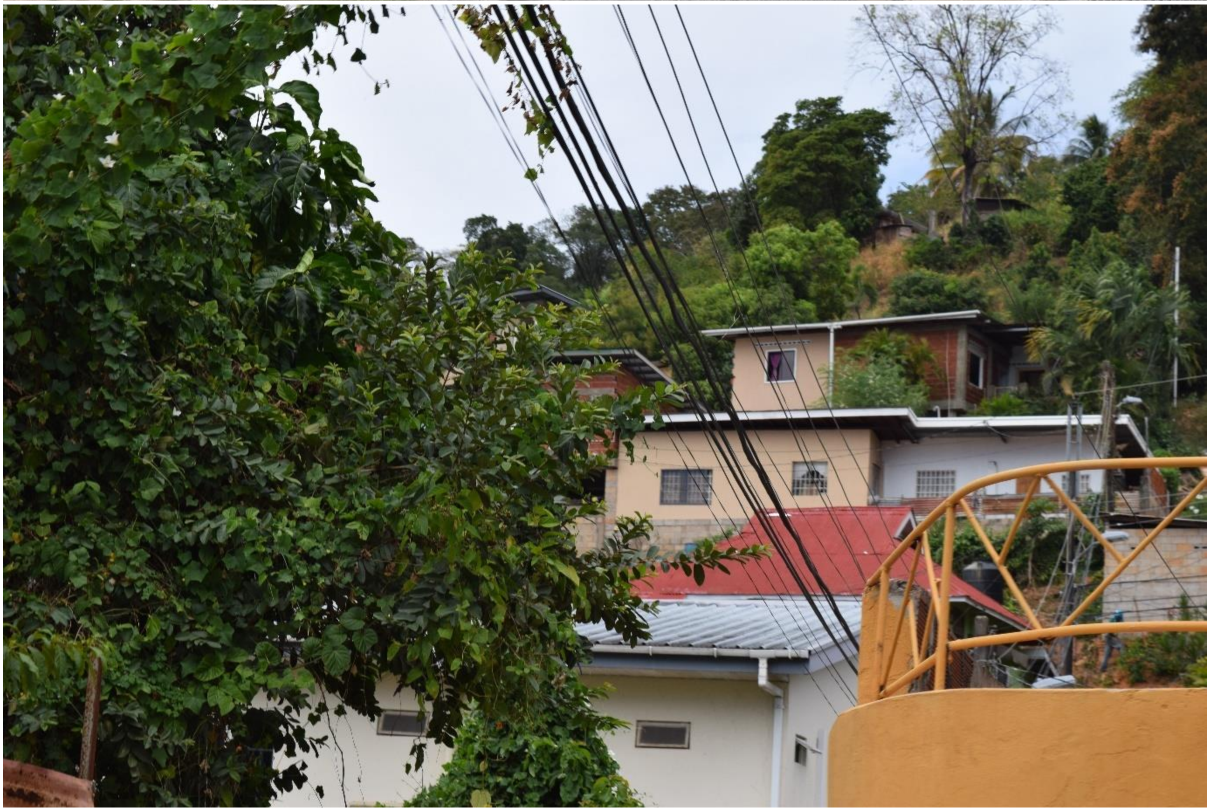

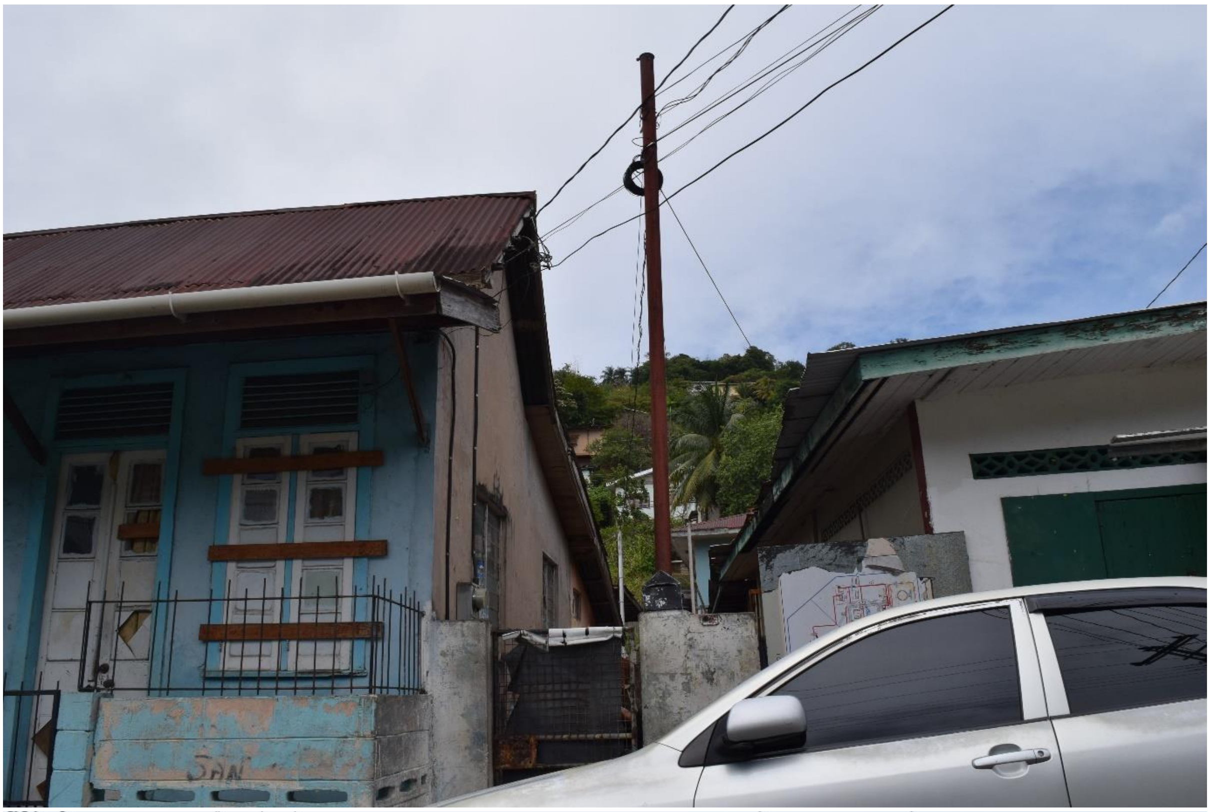
SAN

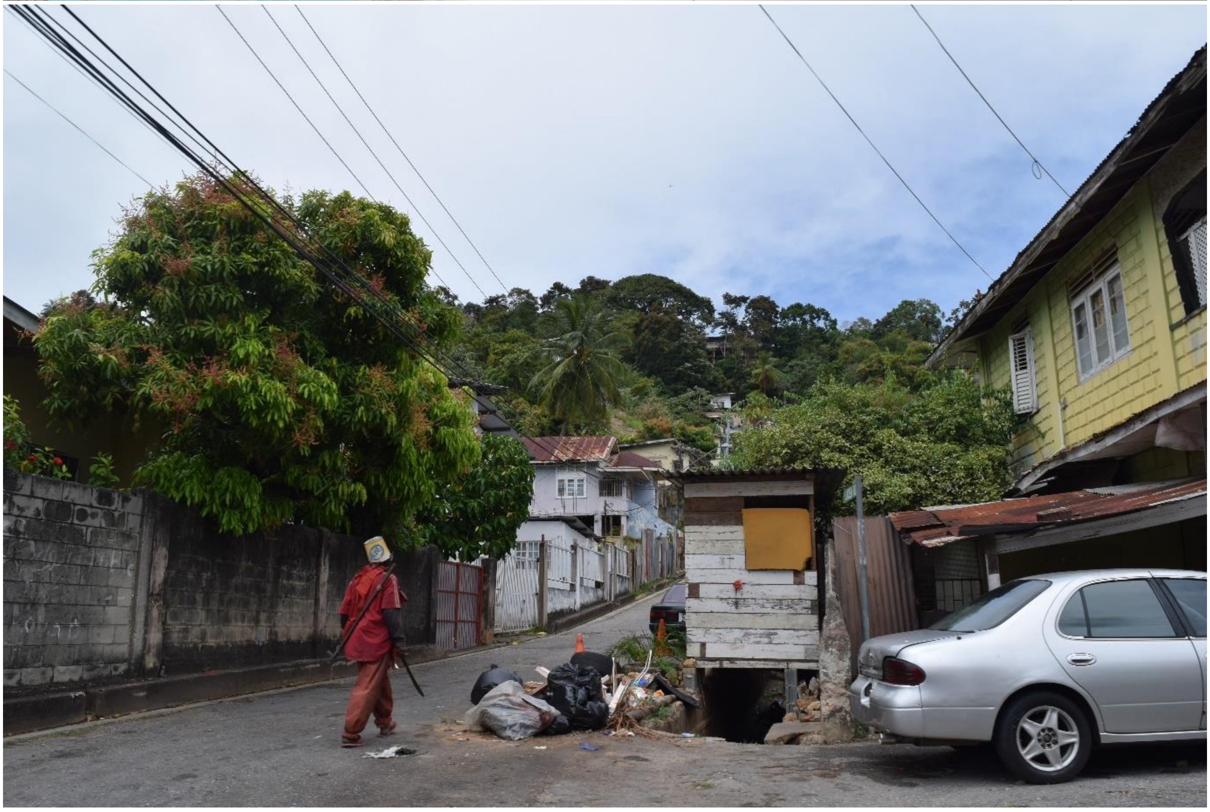

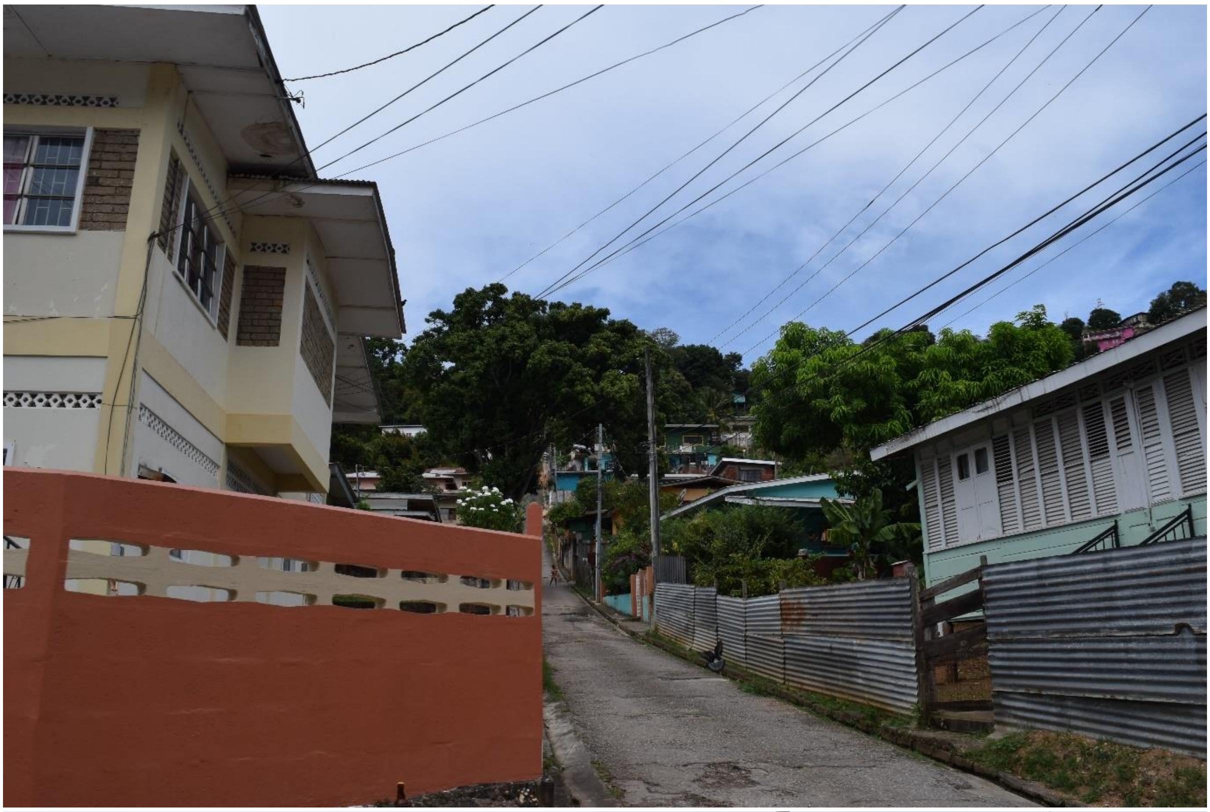

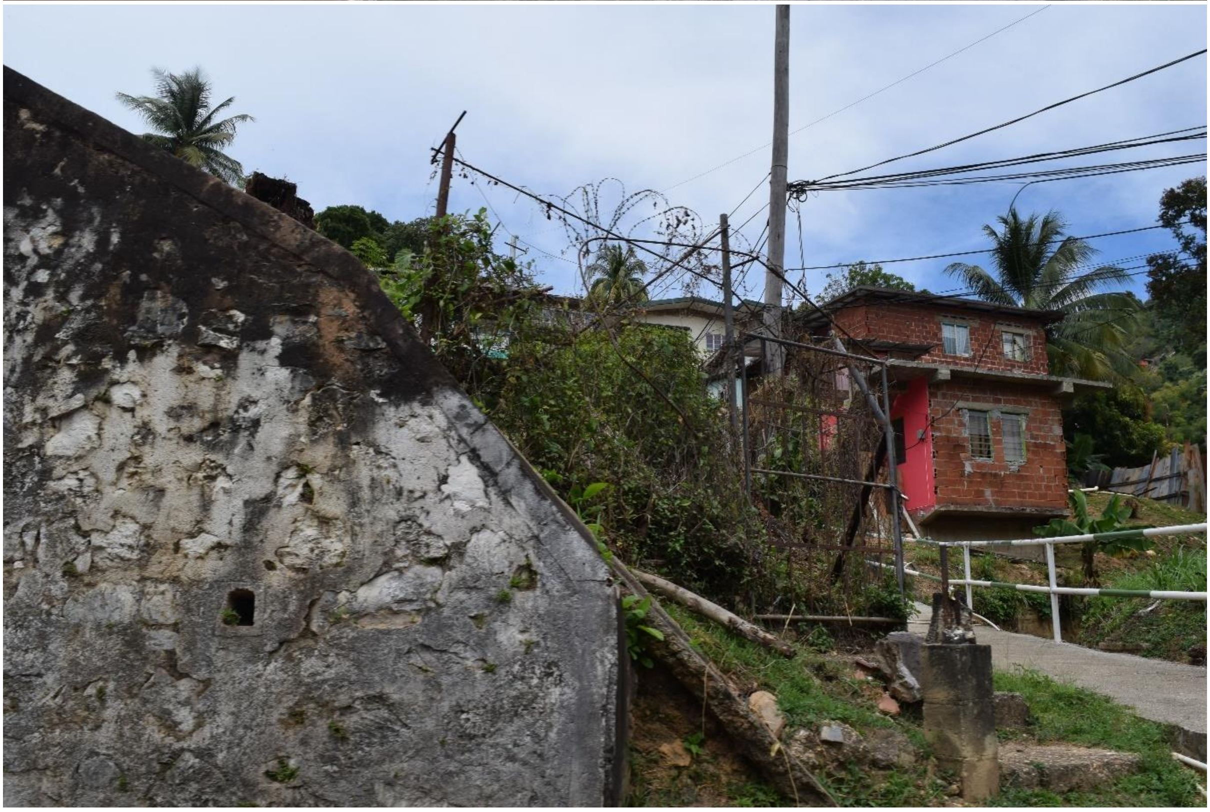

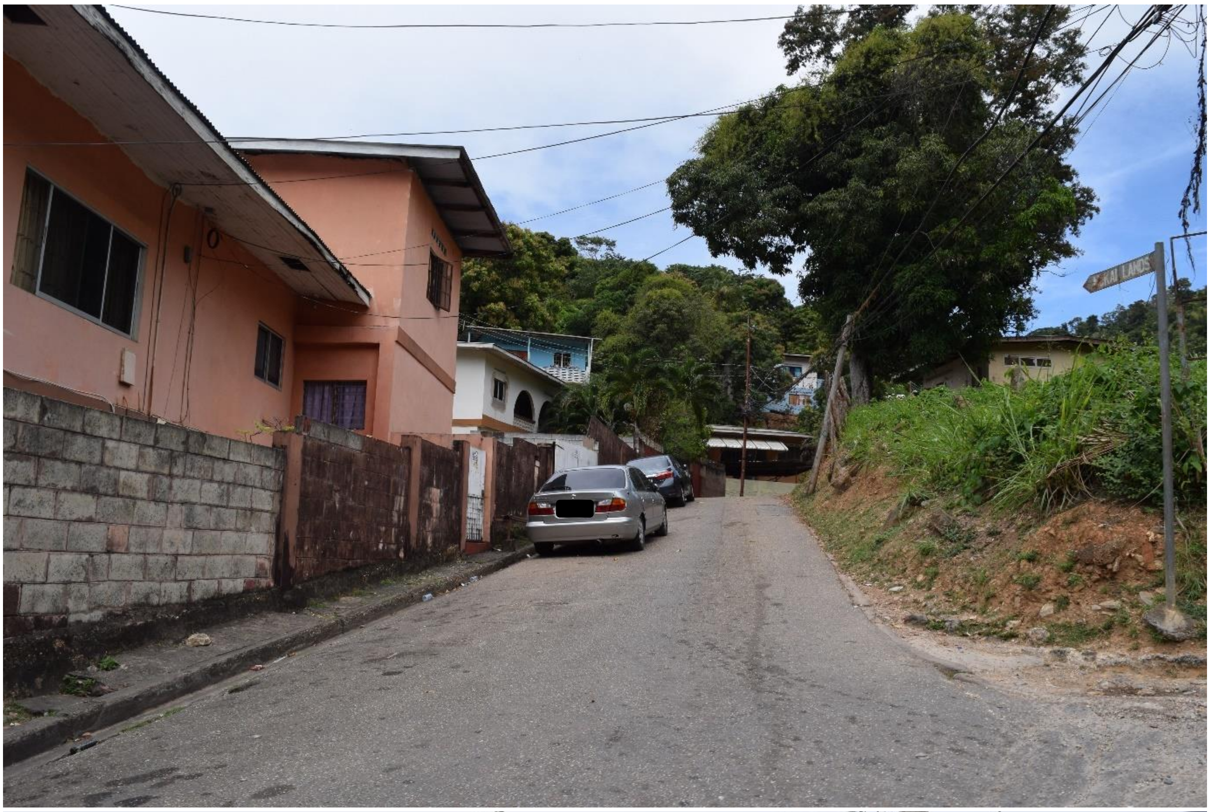

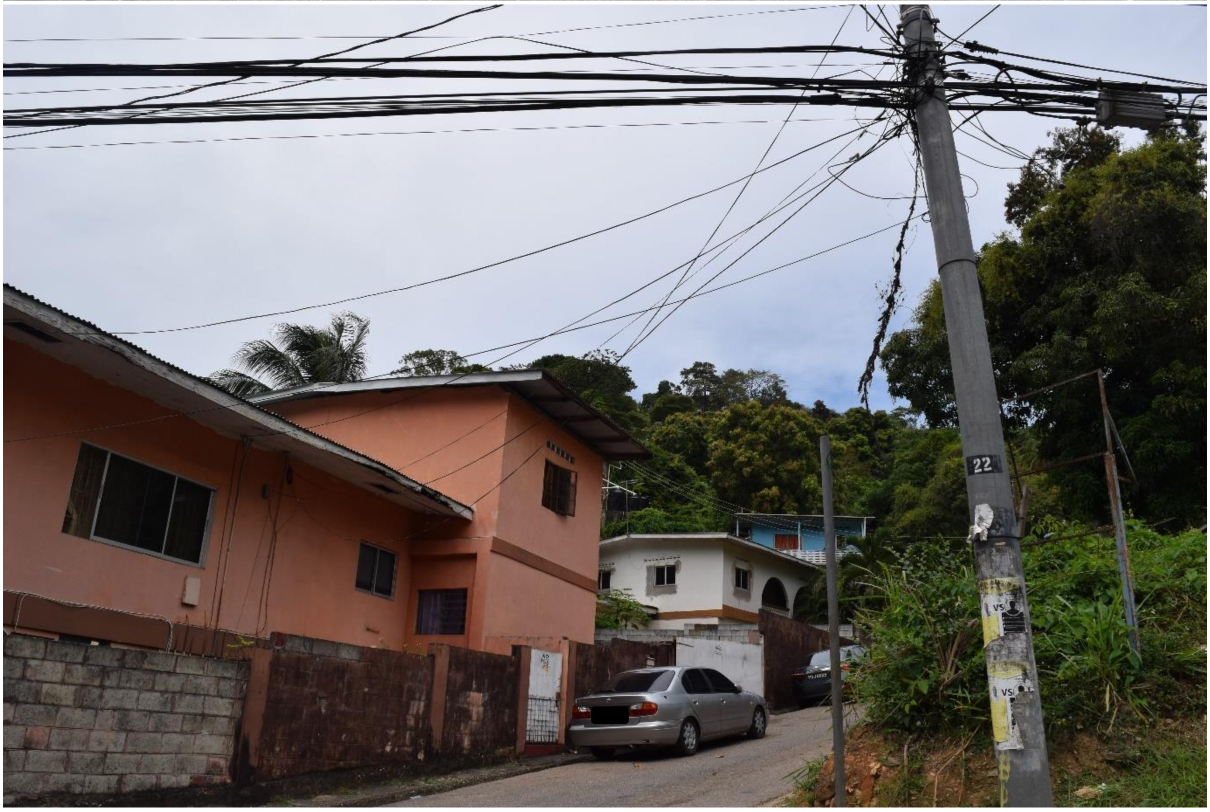
22

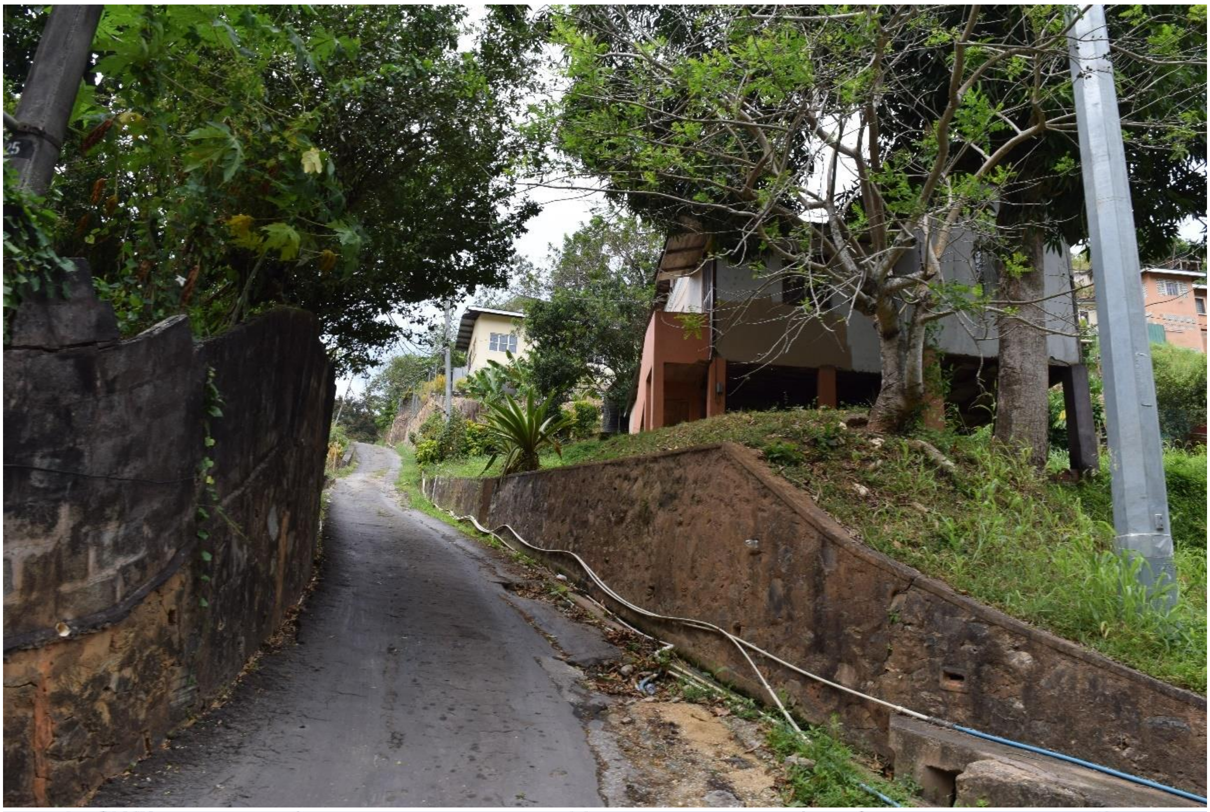

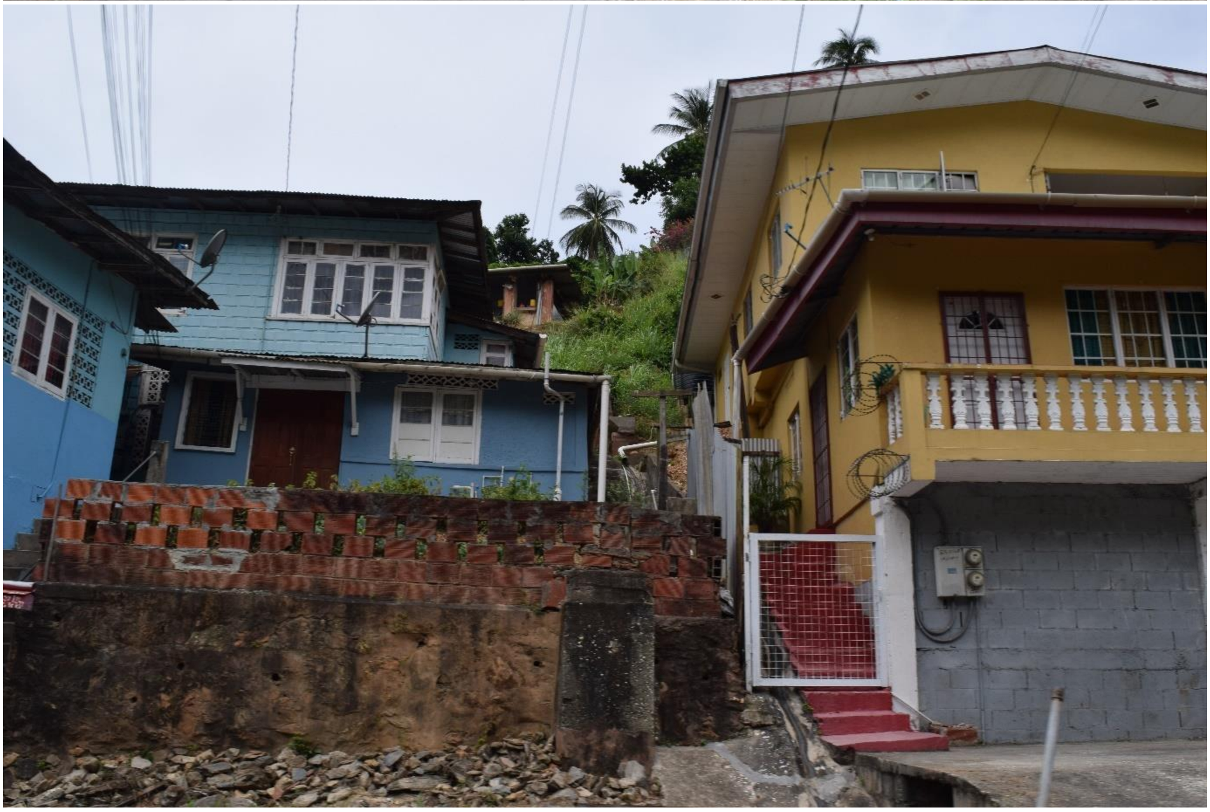

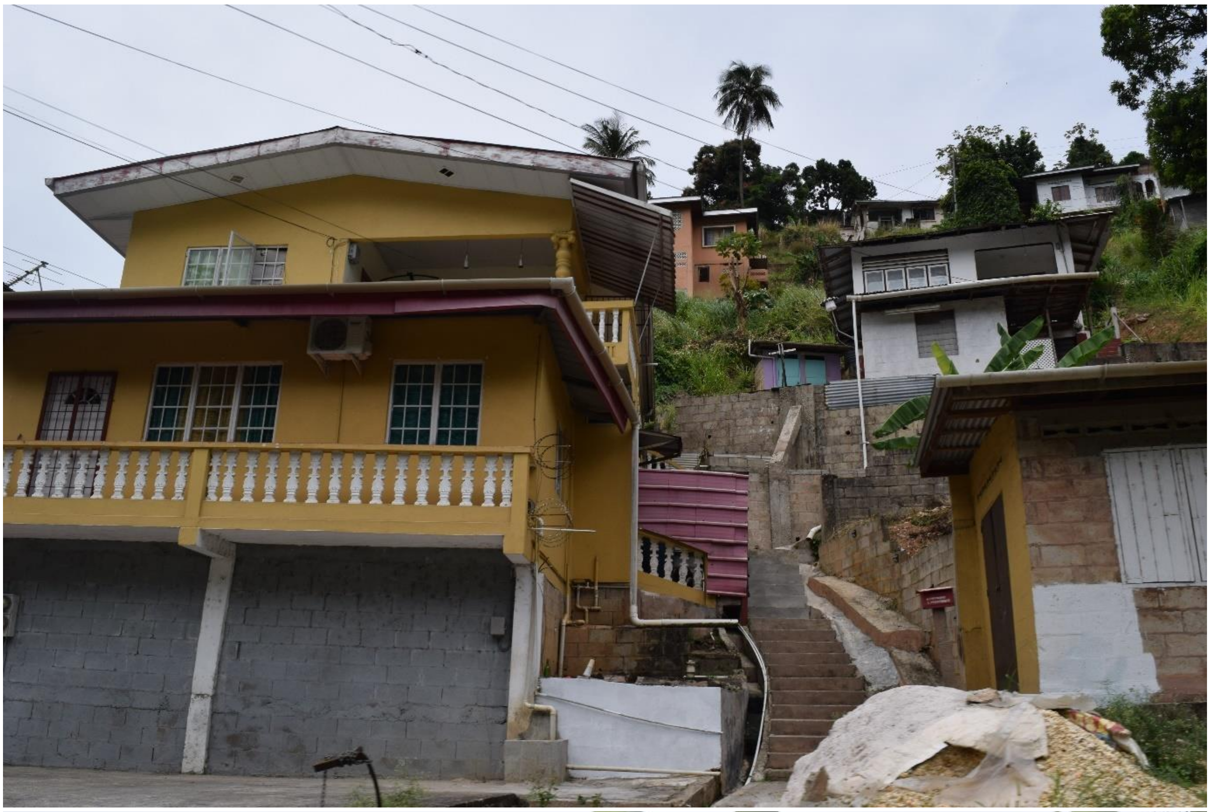

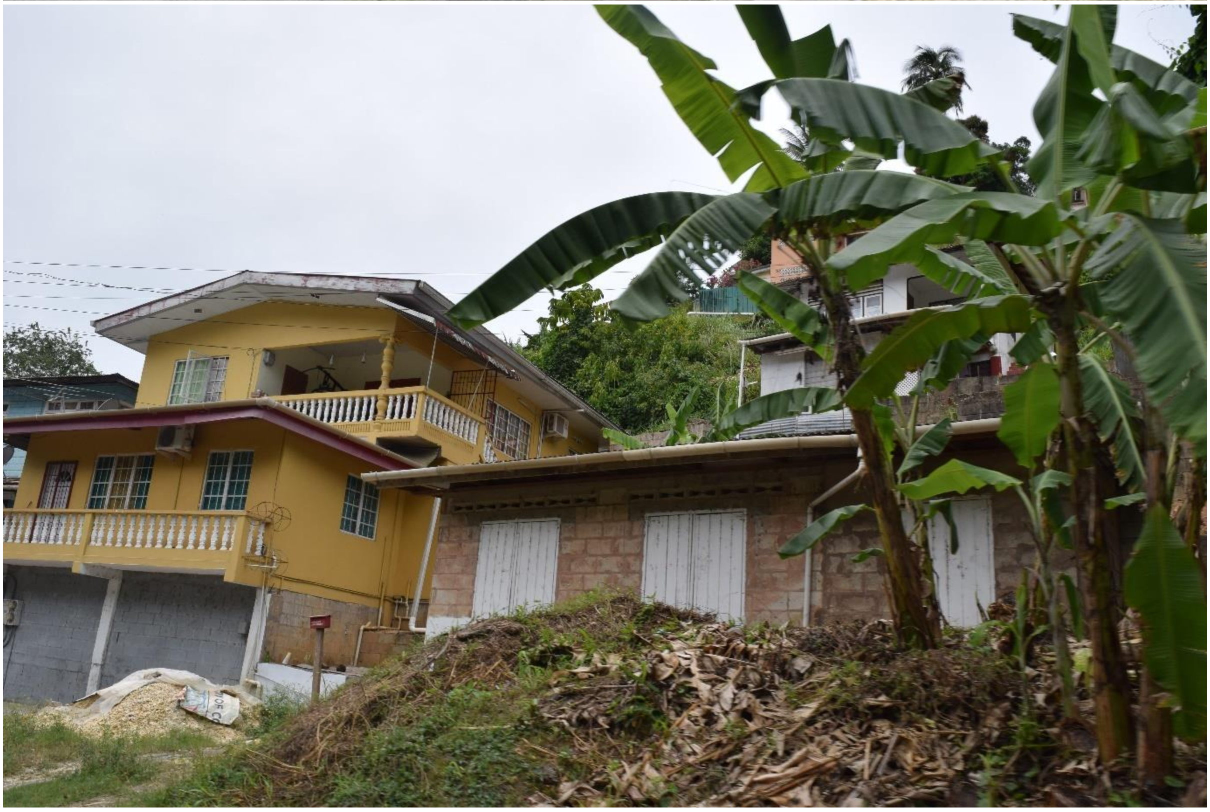

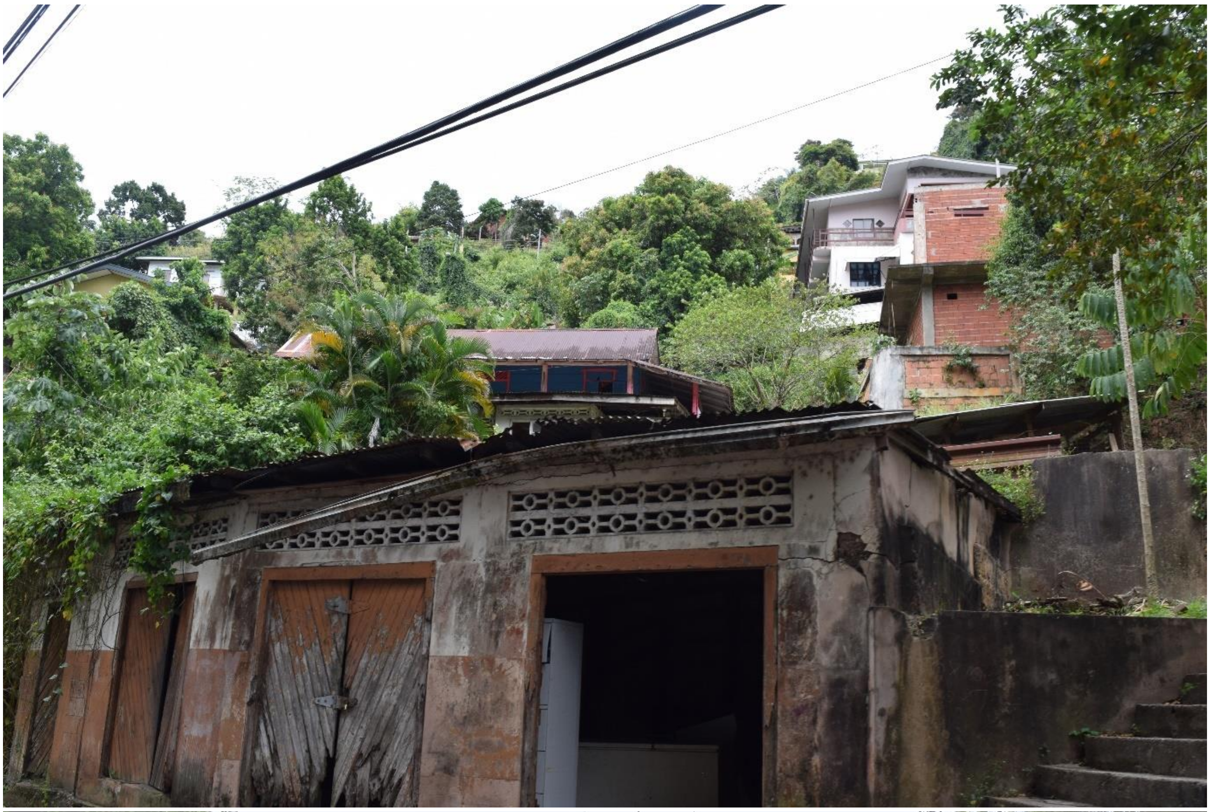

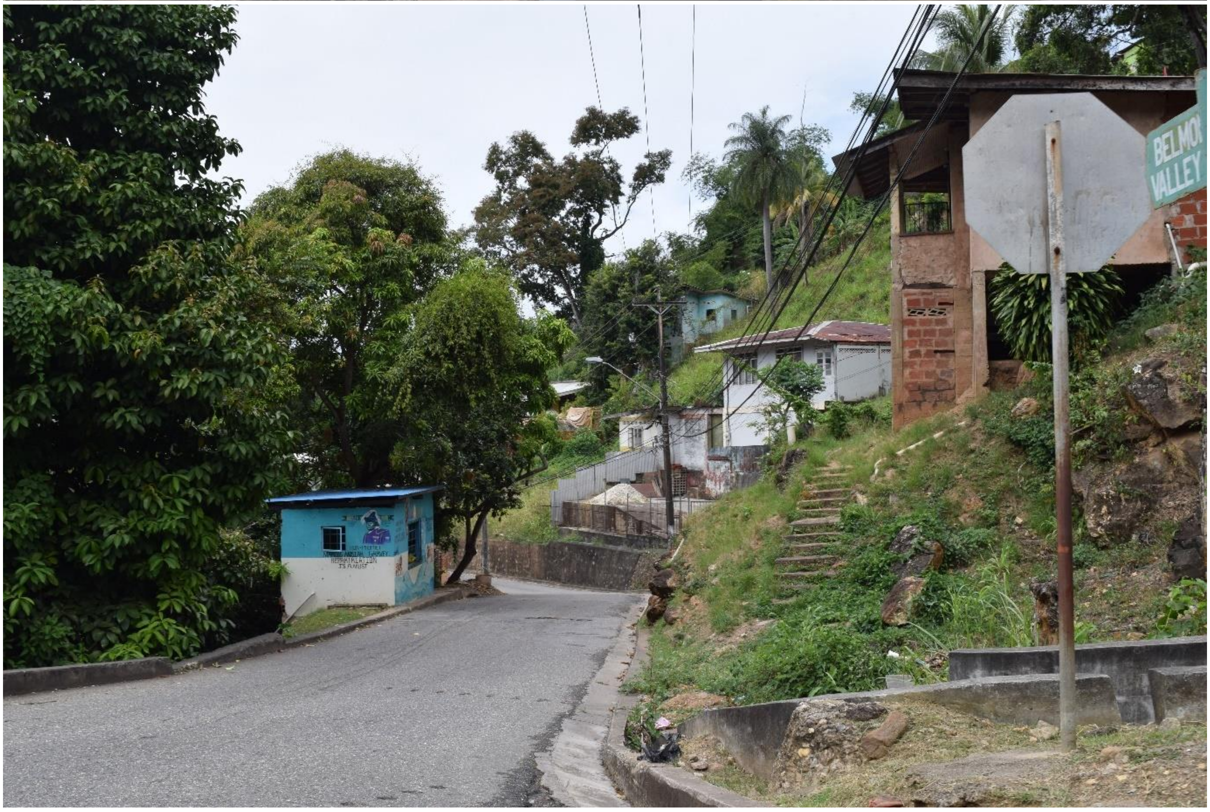
VALLEY

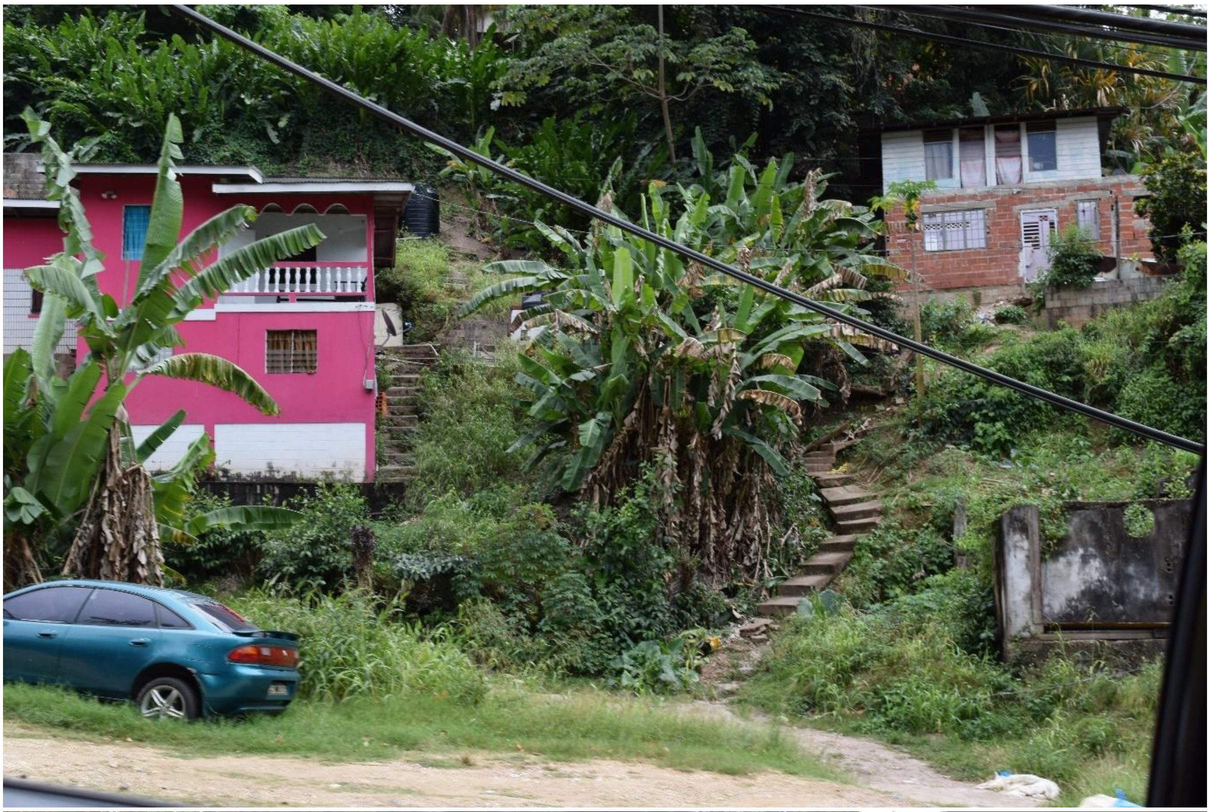

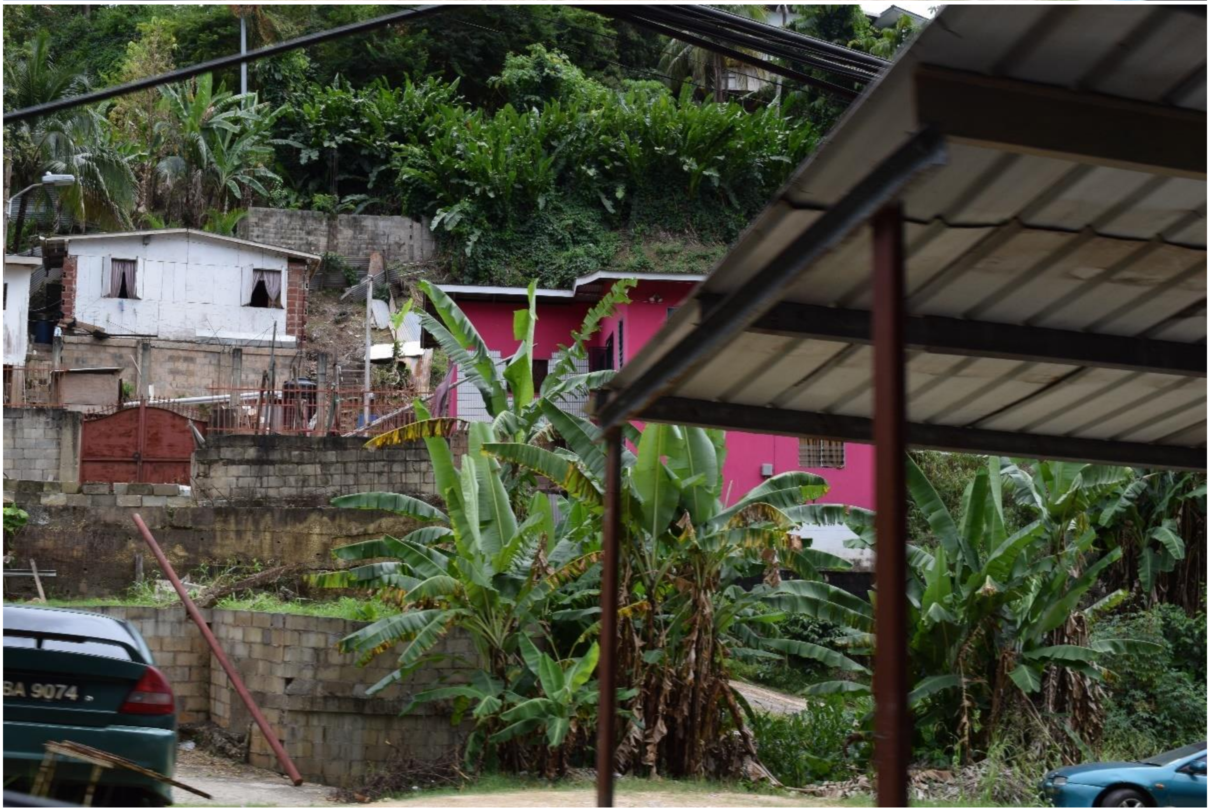
BA 9074

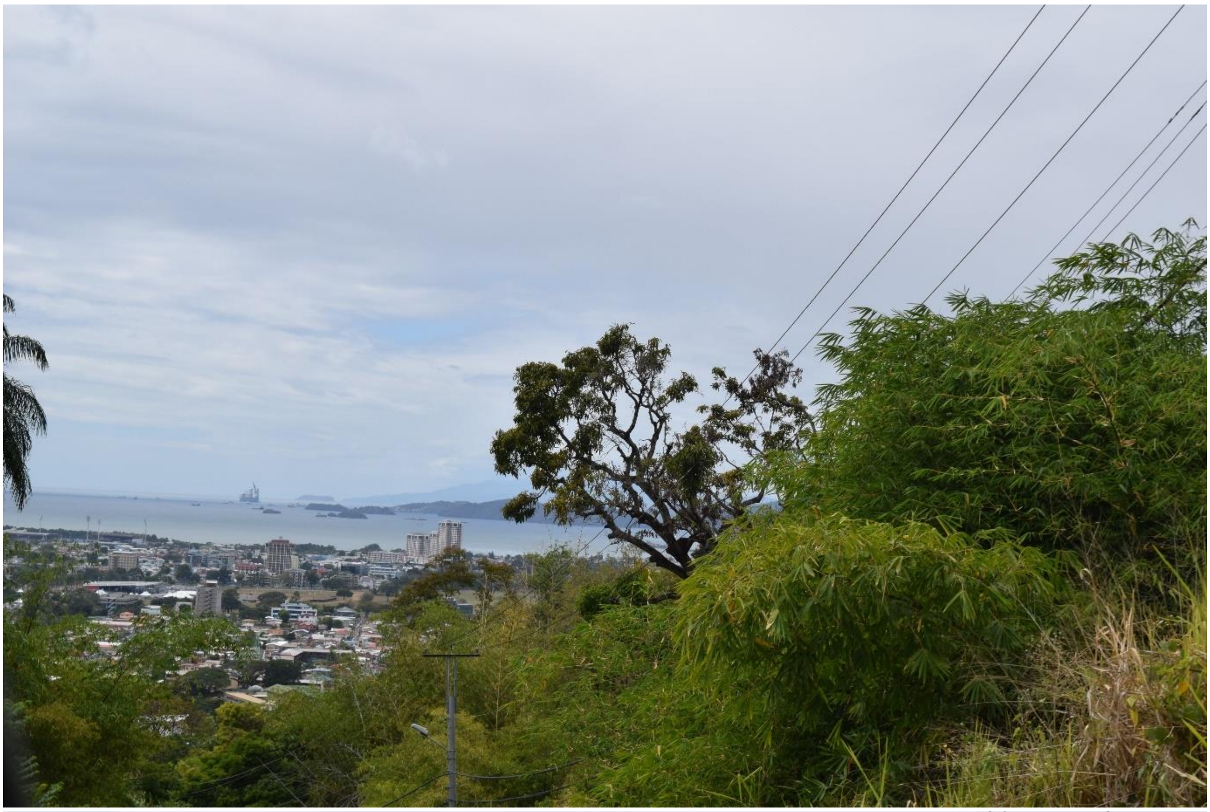

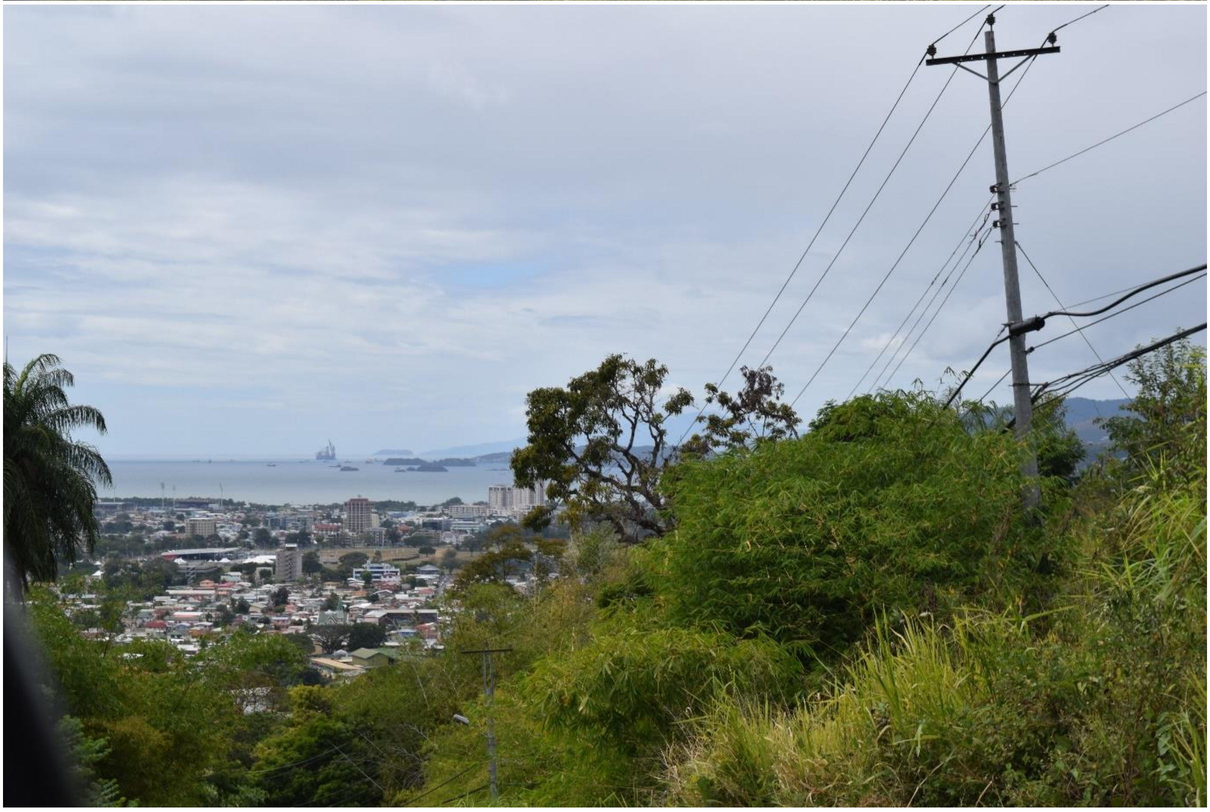

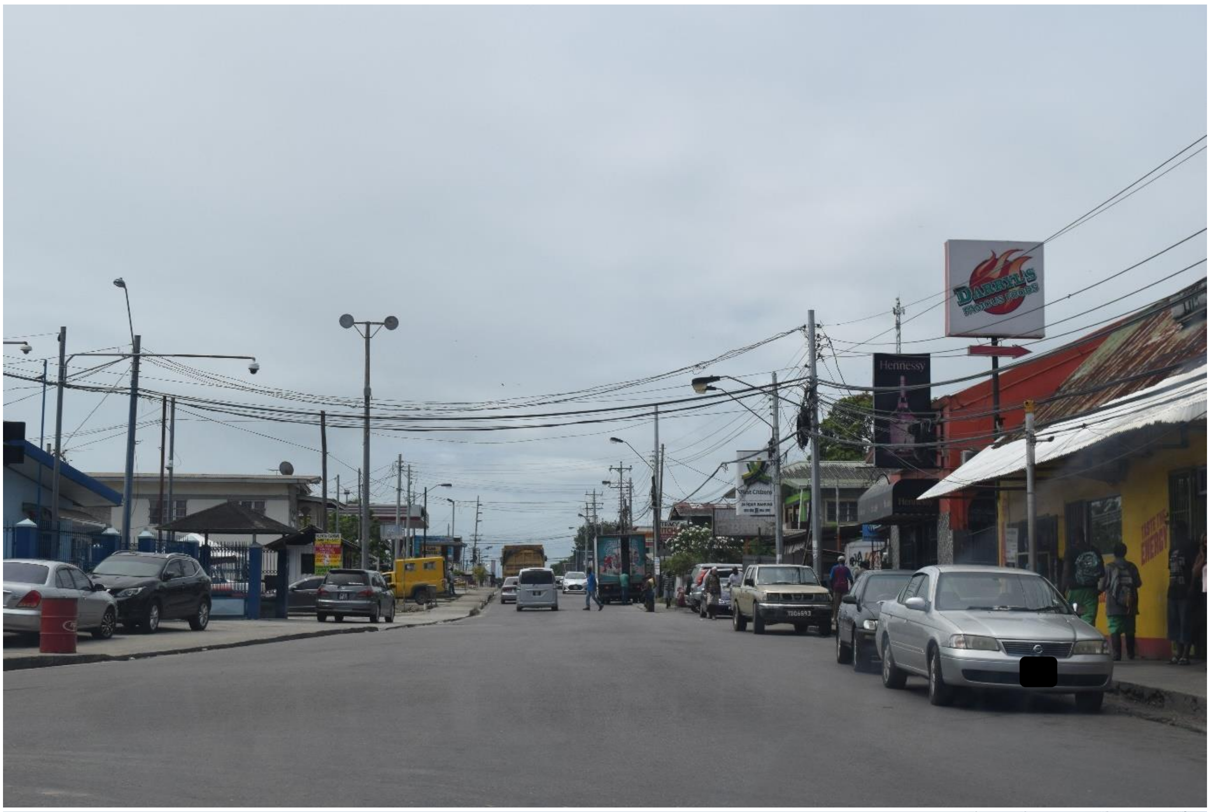
Hennessy

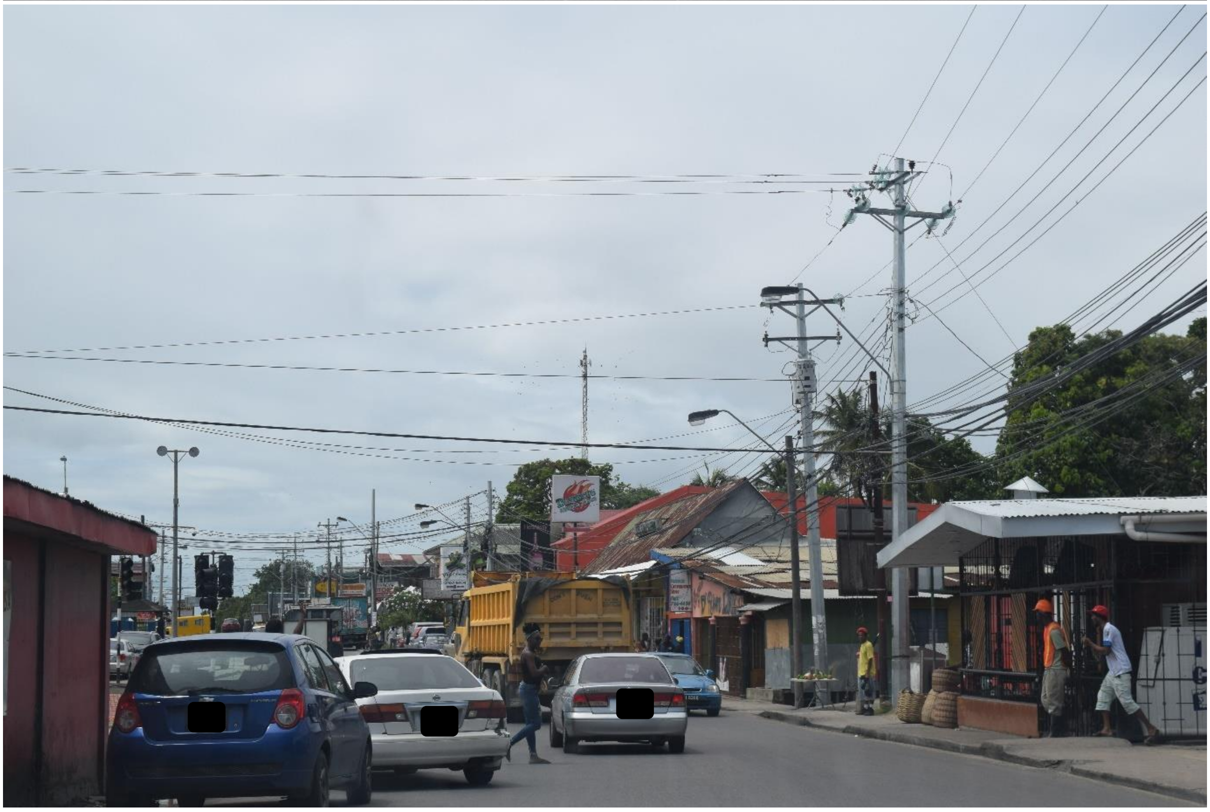

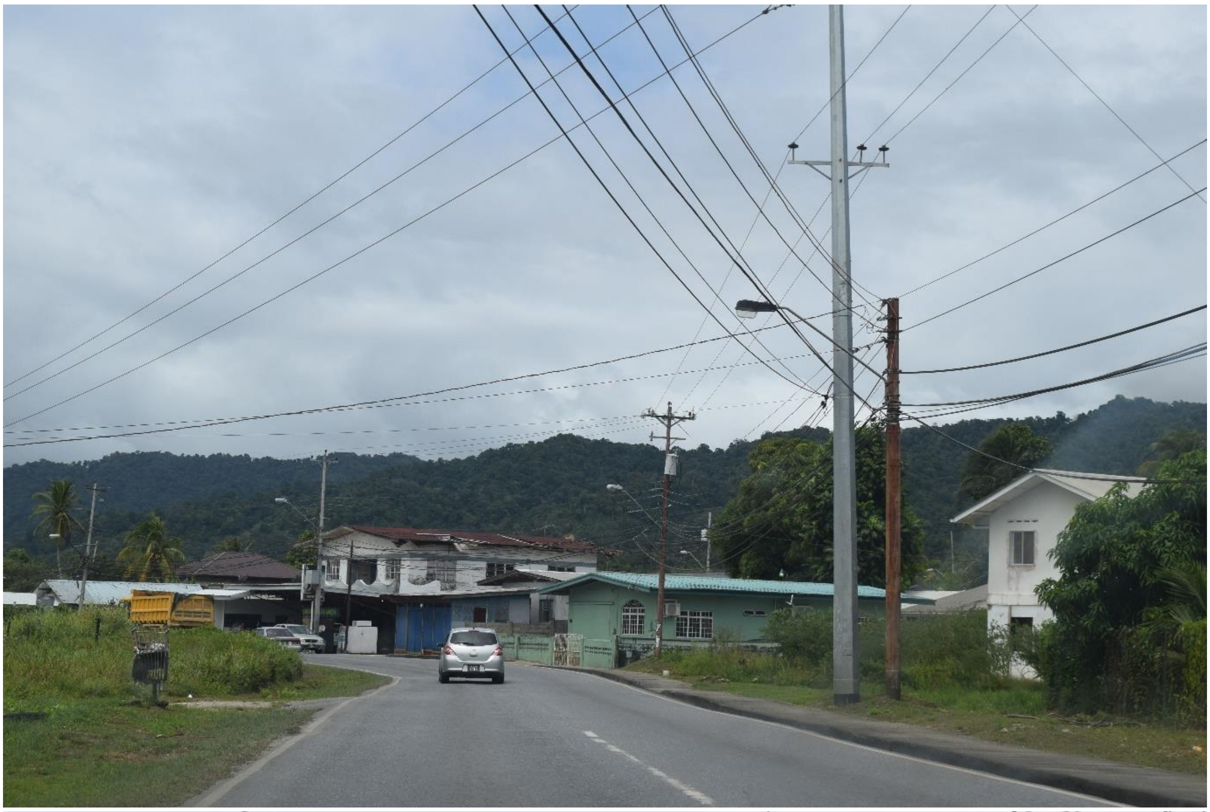

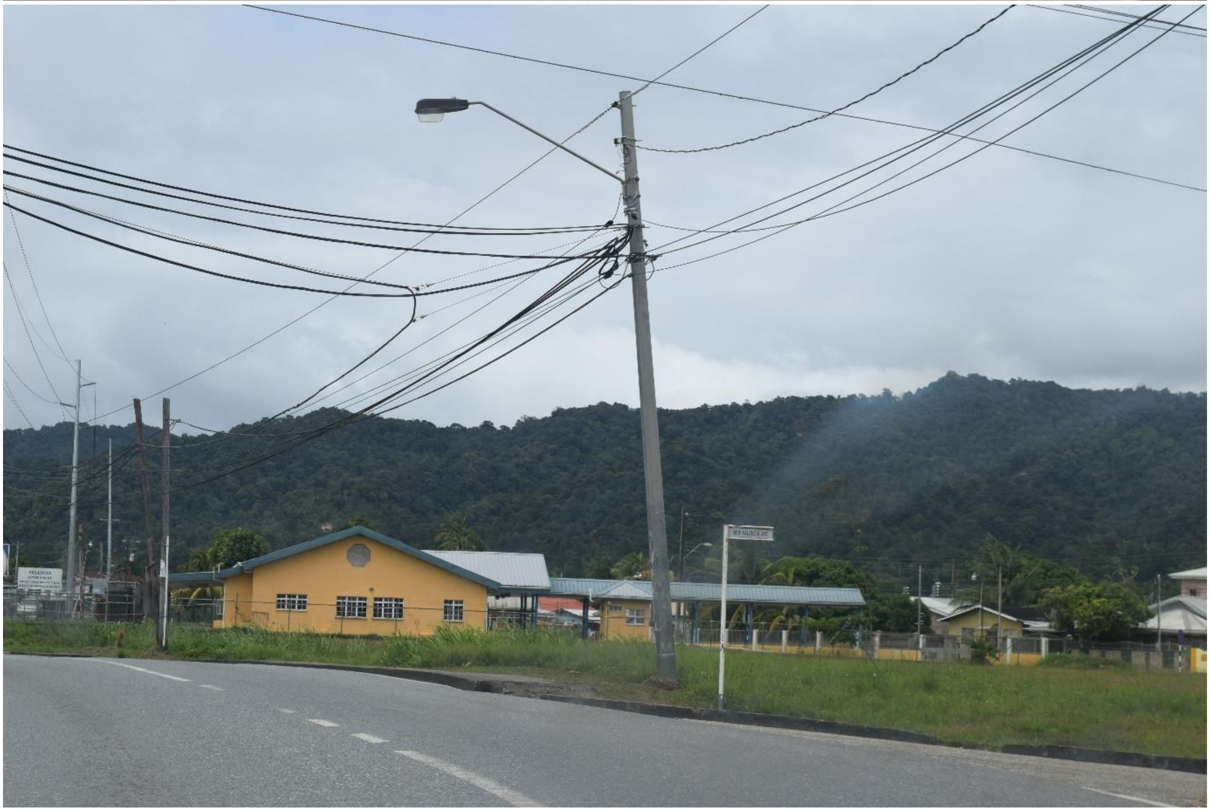

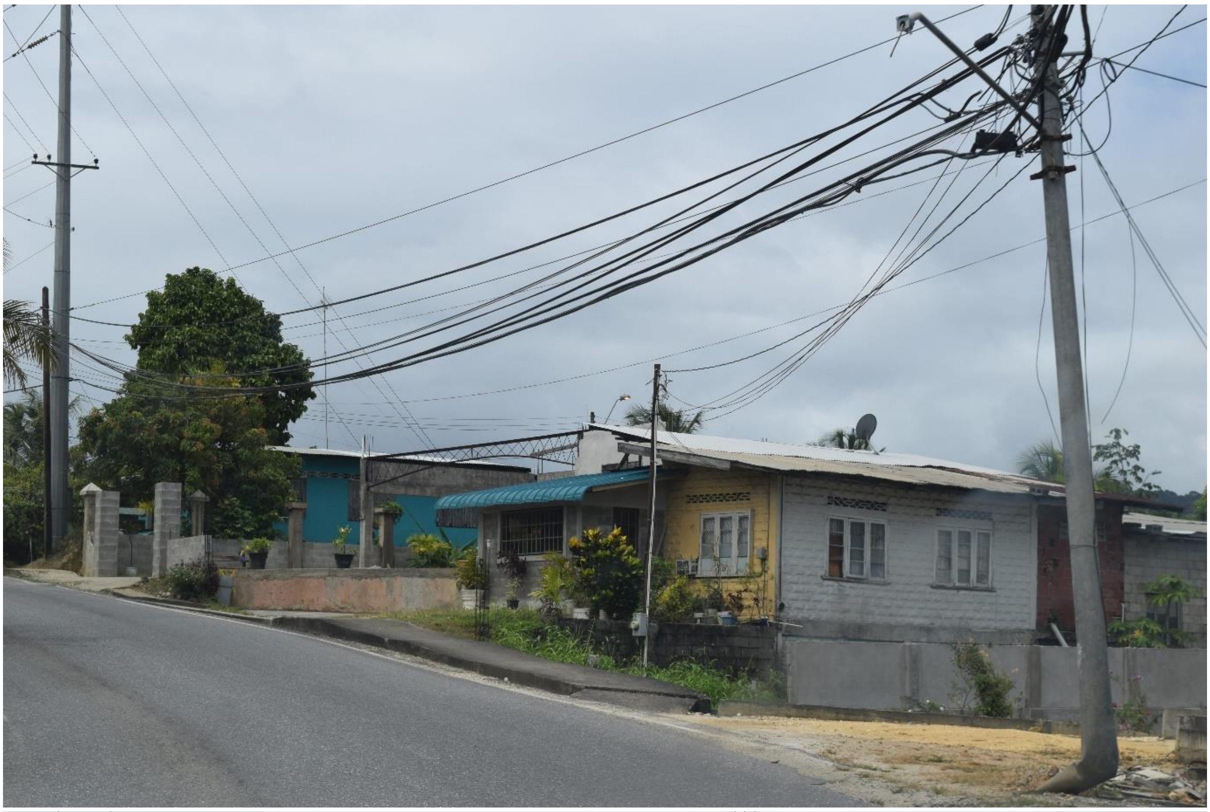

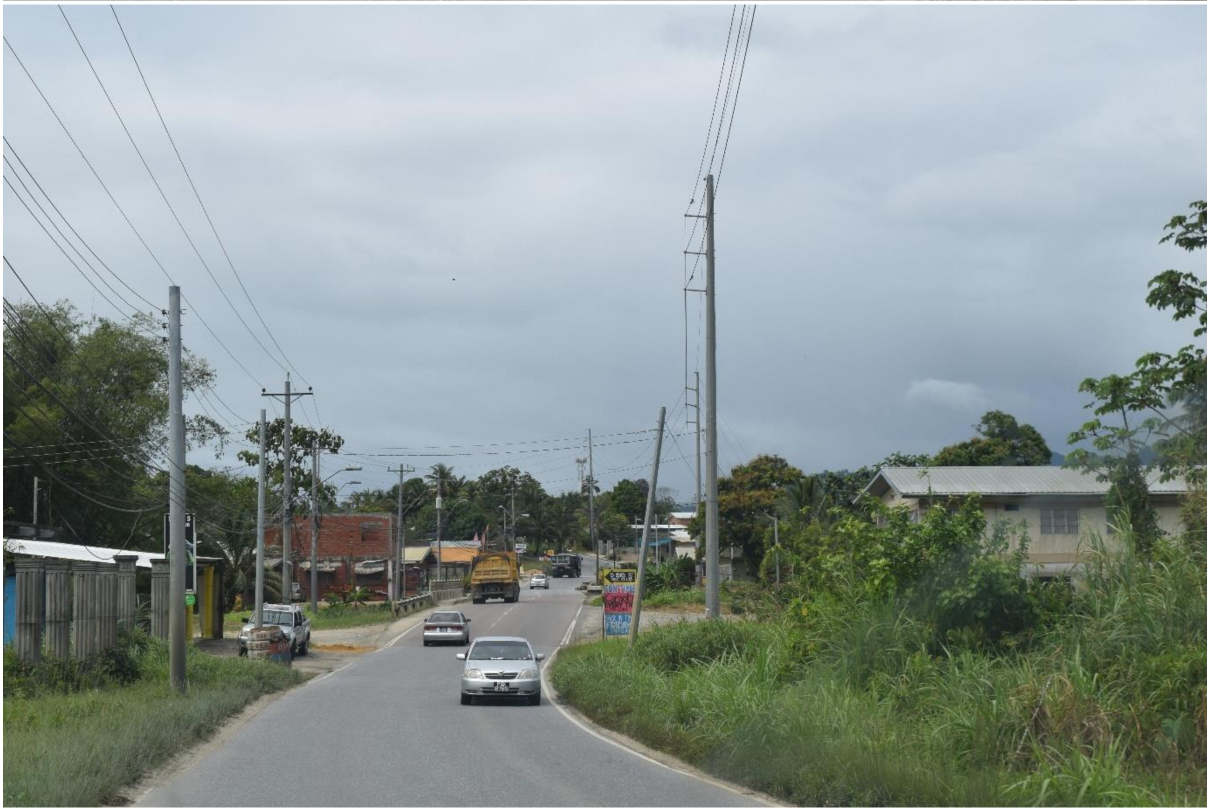

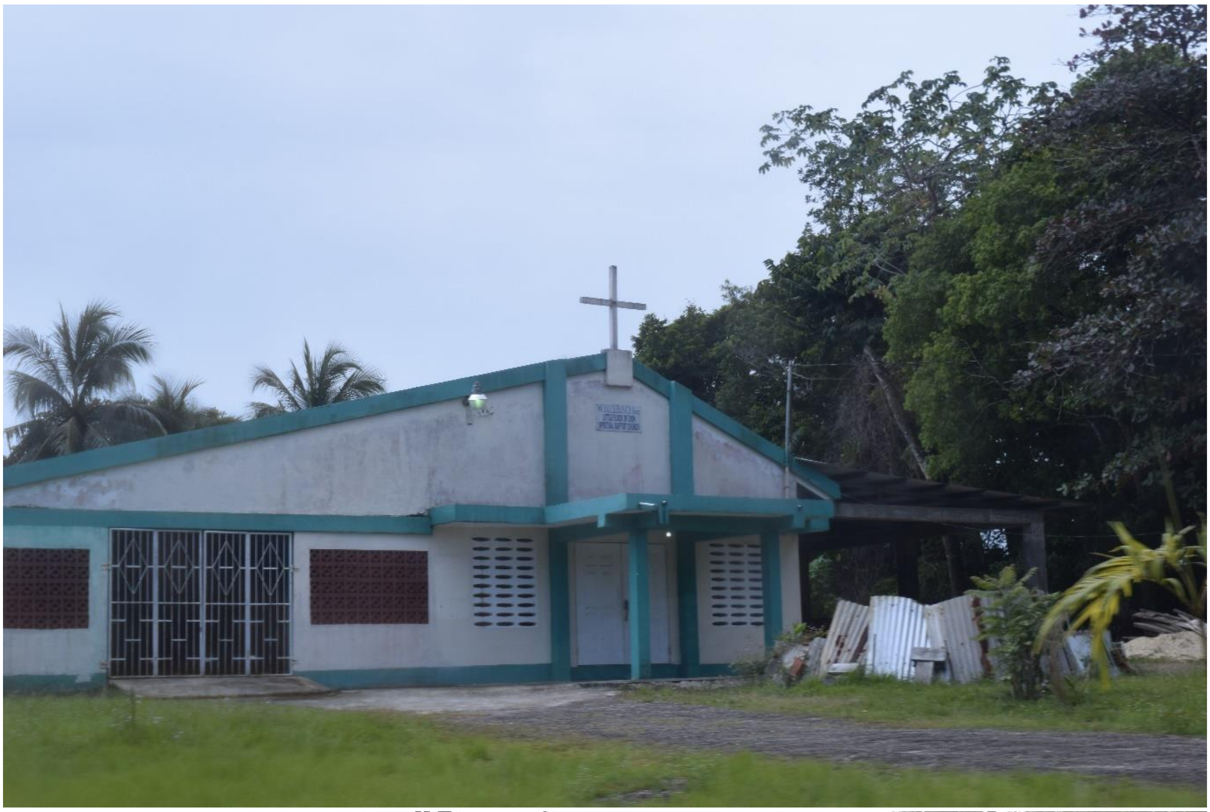

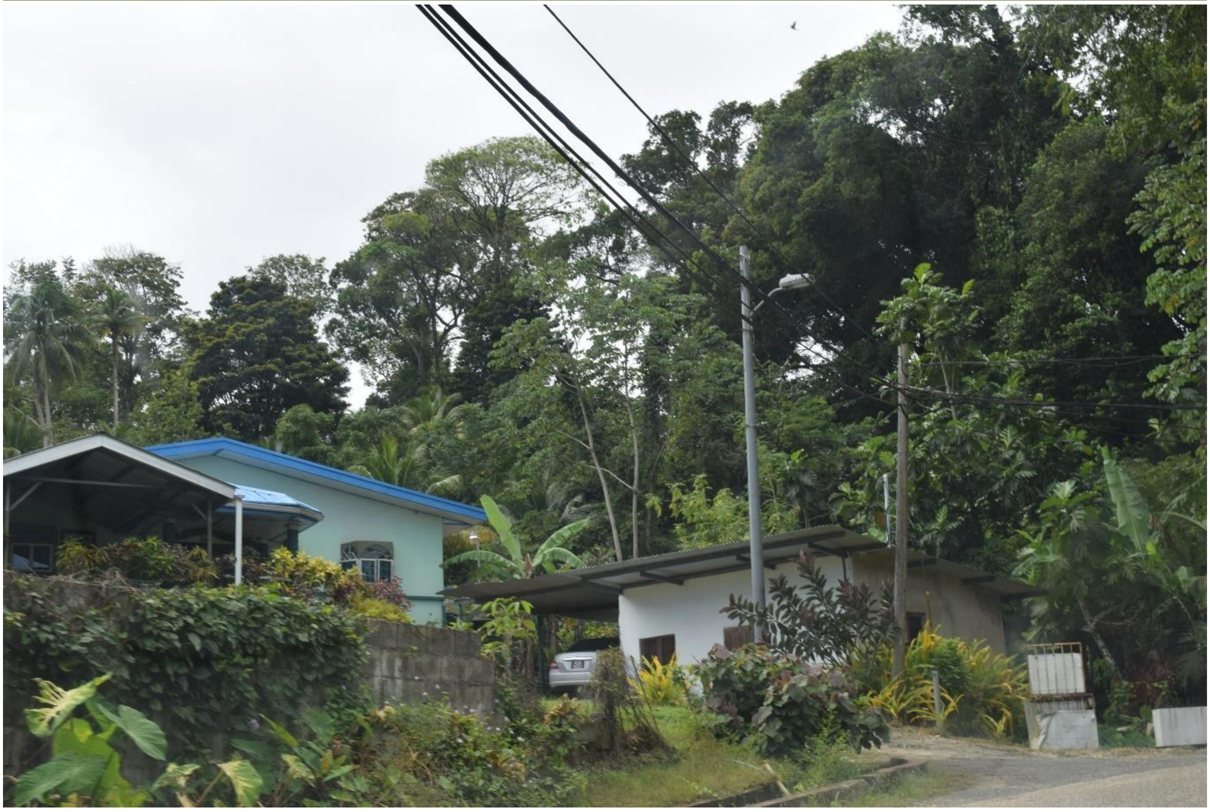

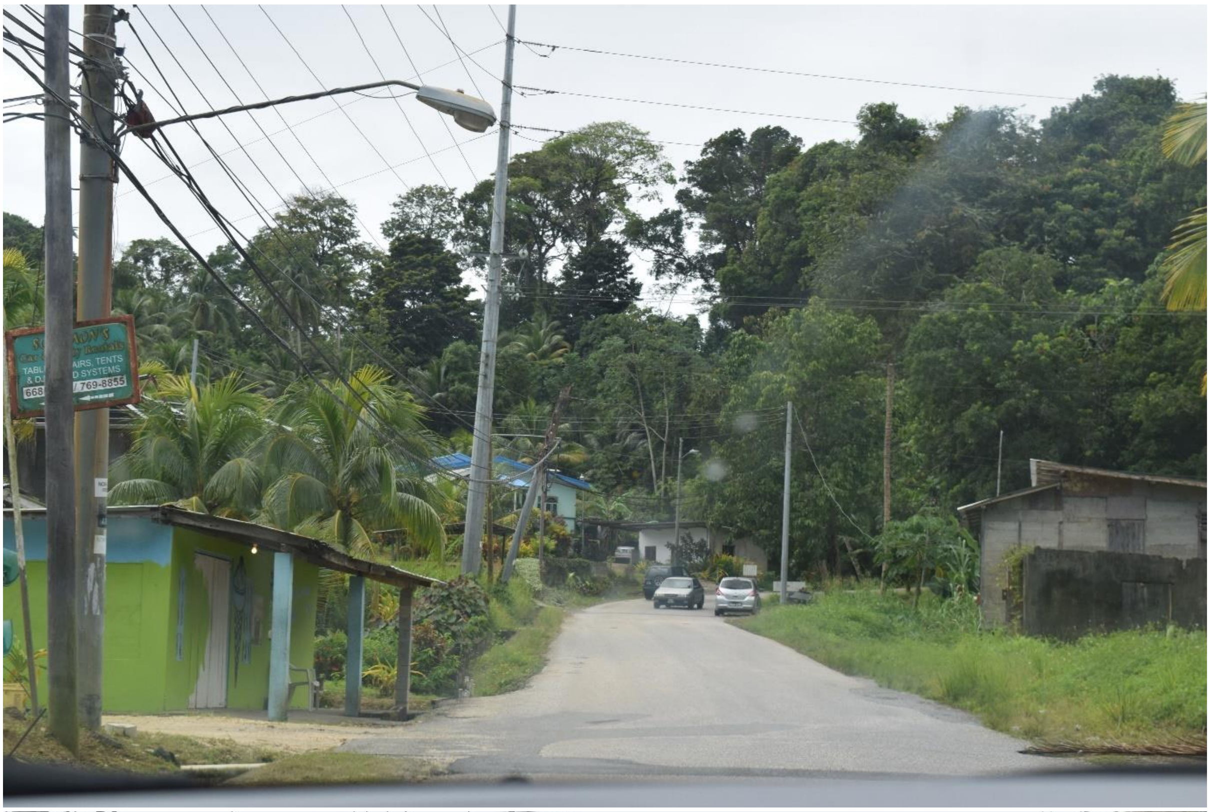
TABL AIRS, TENTS
D SYSTEMS
668 769-8855

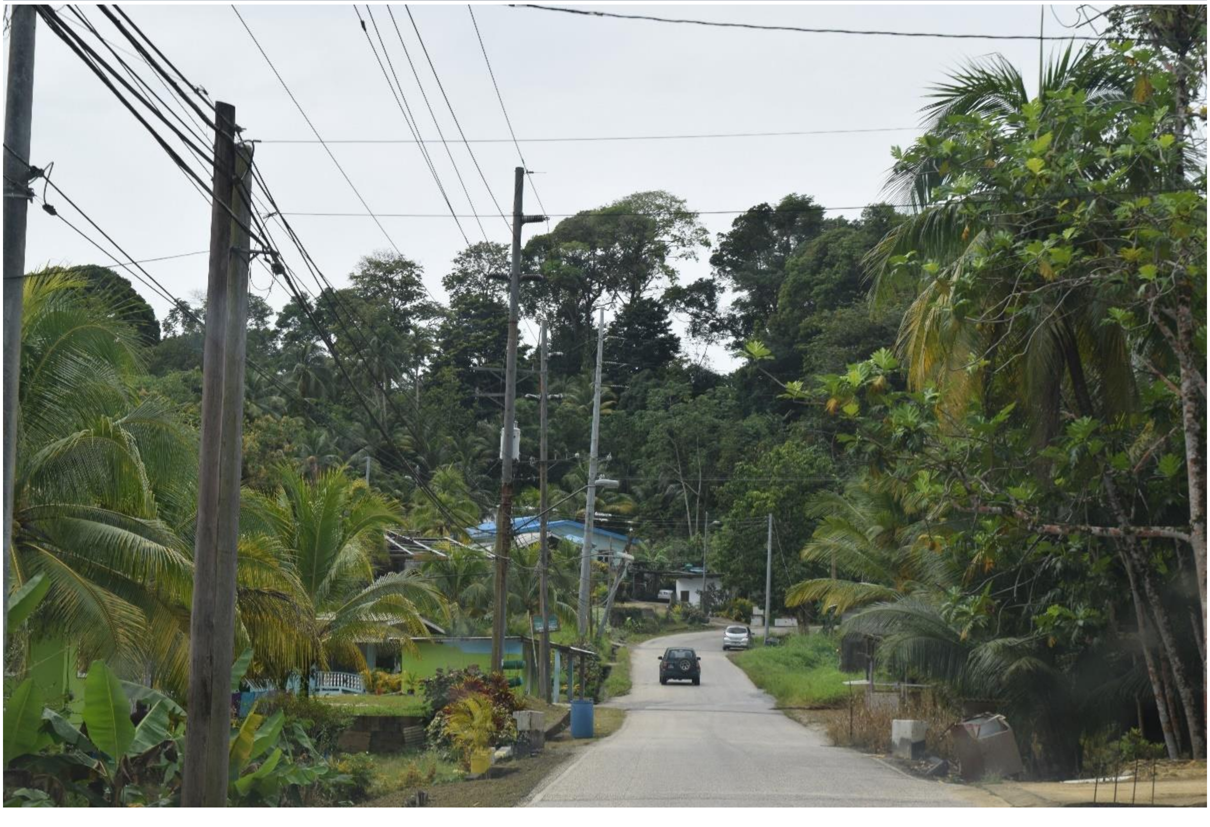

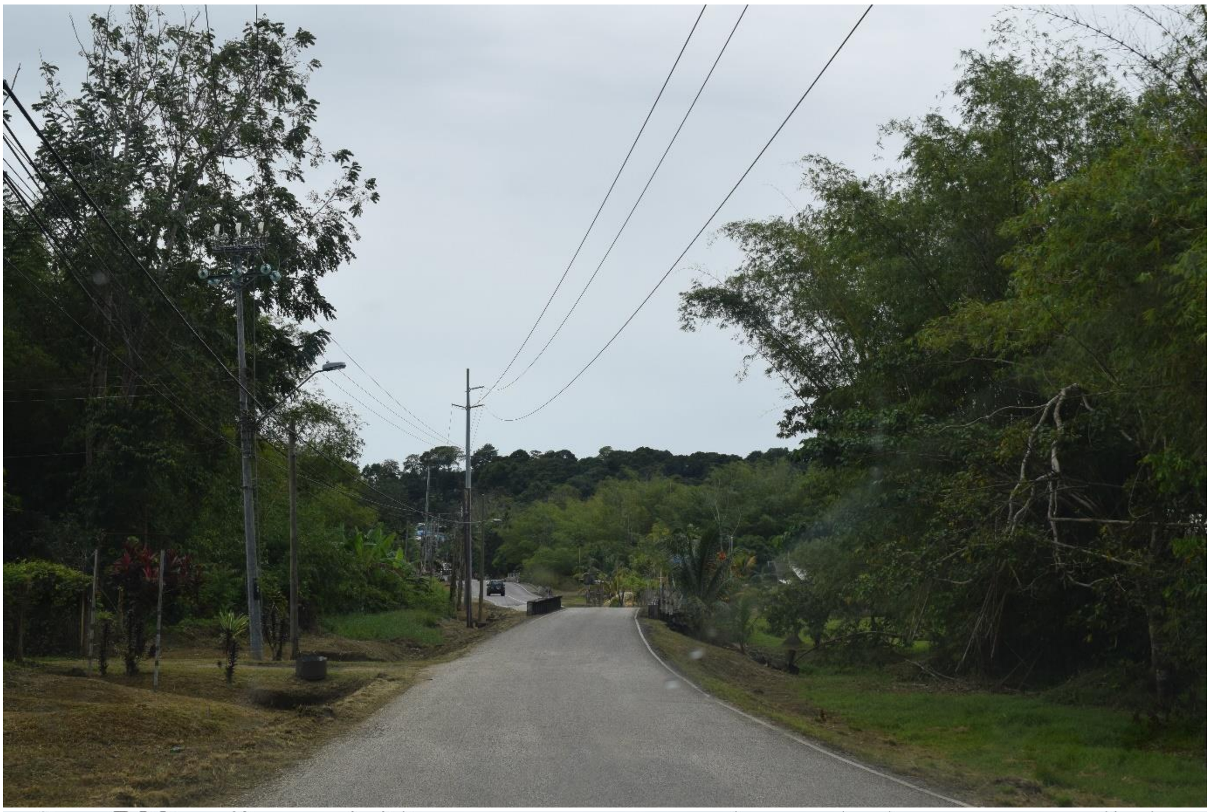

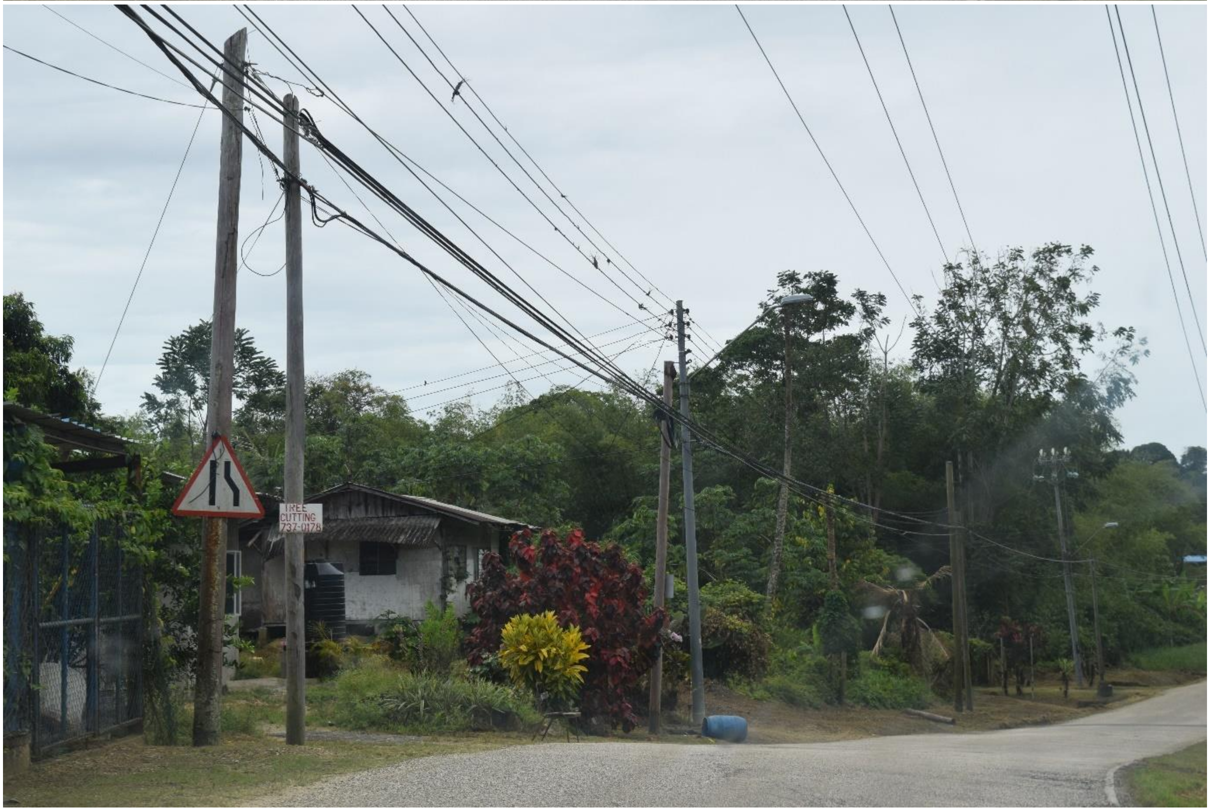
TREE
CUTTING
737-0178

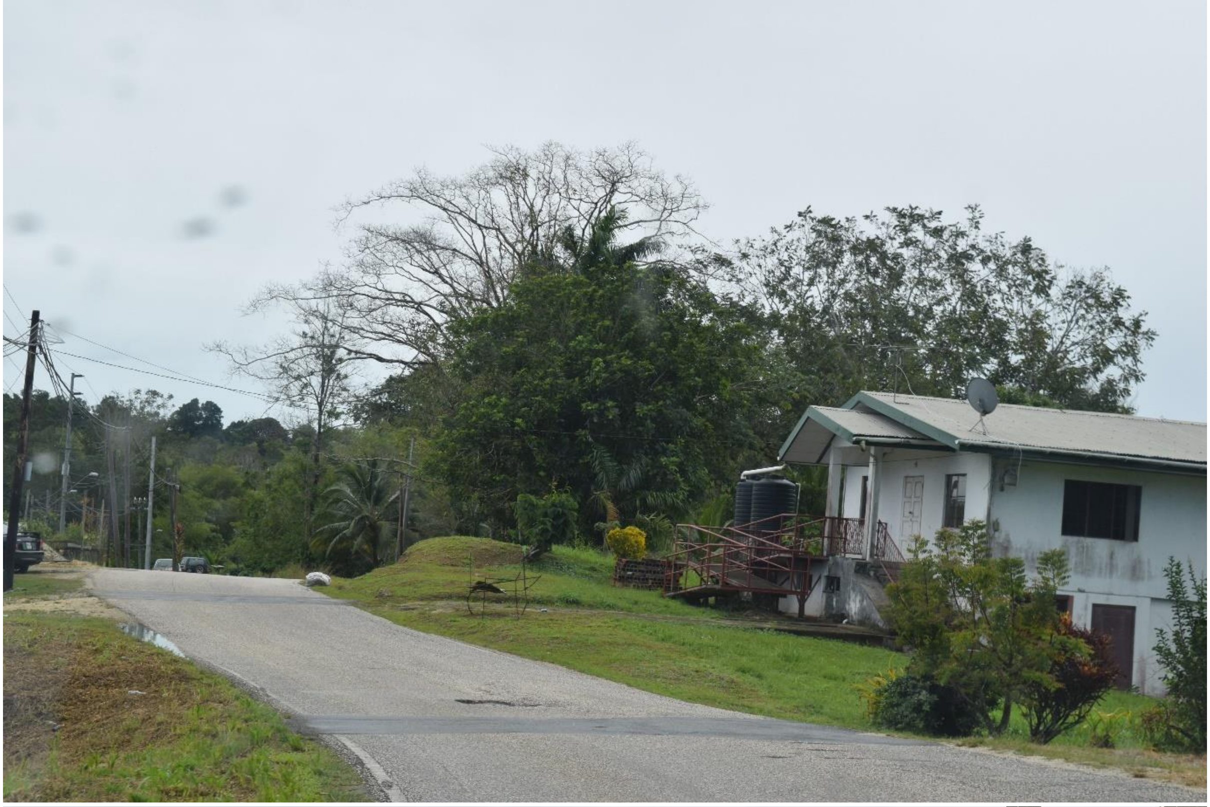

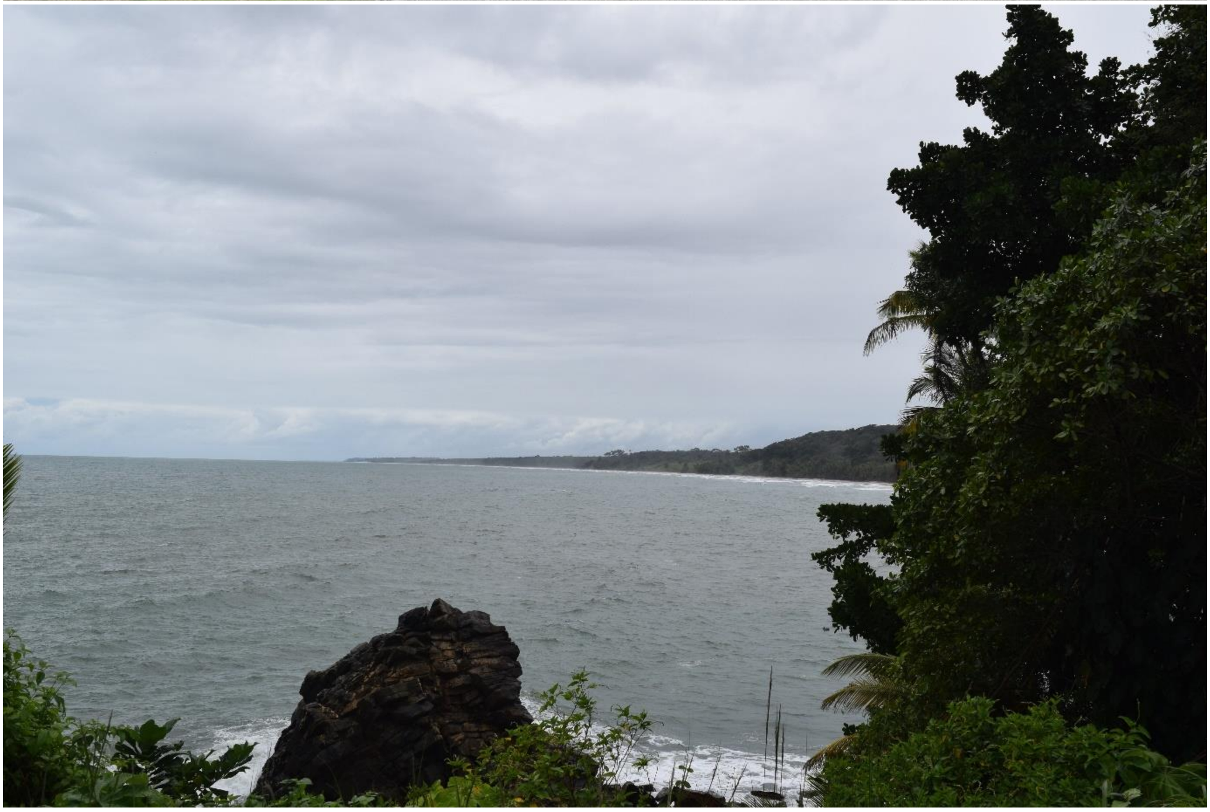

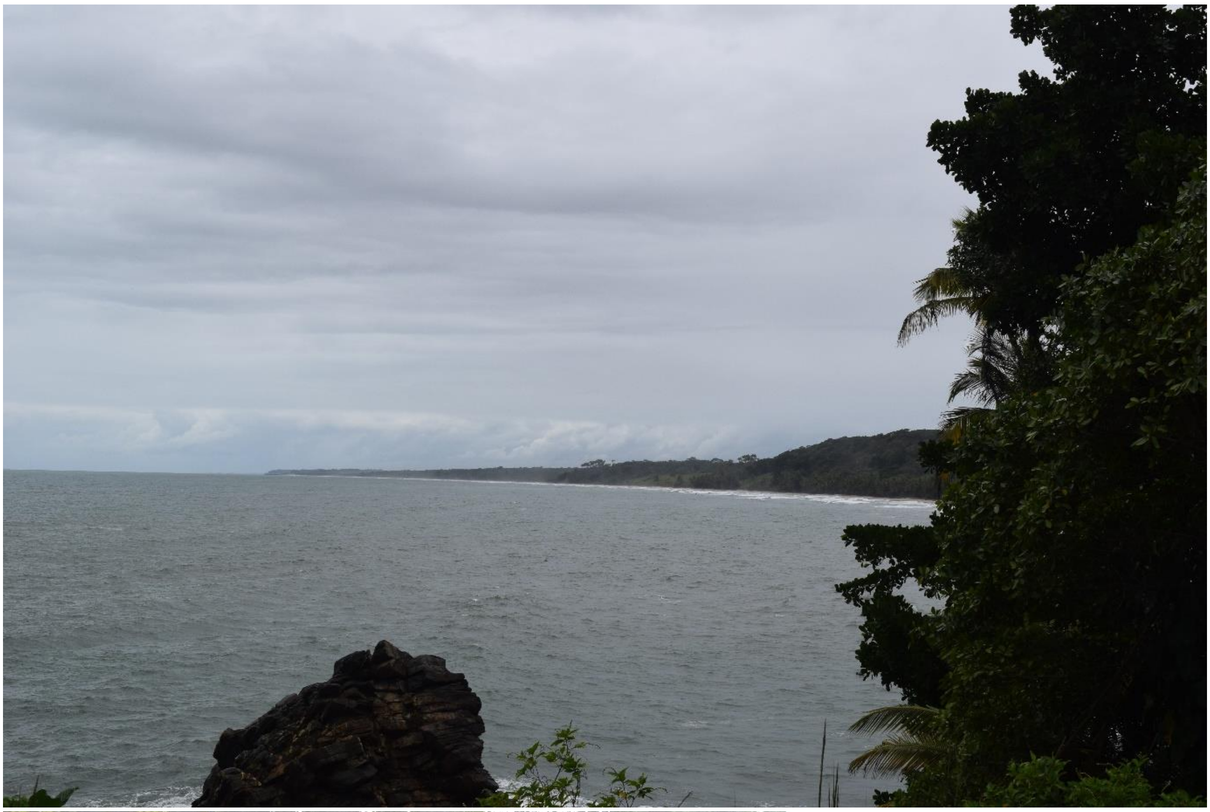

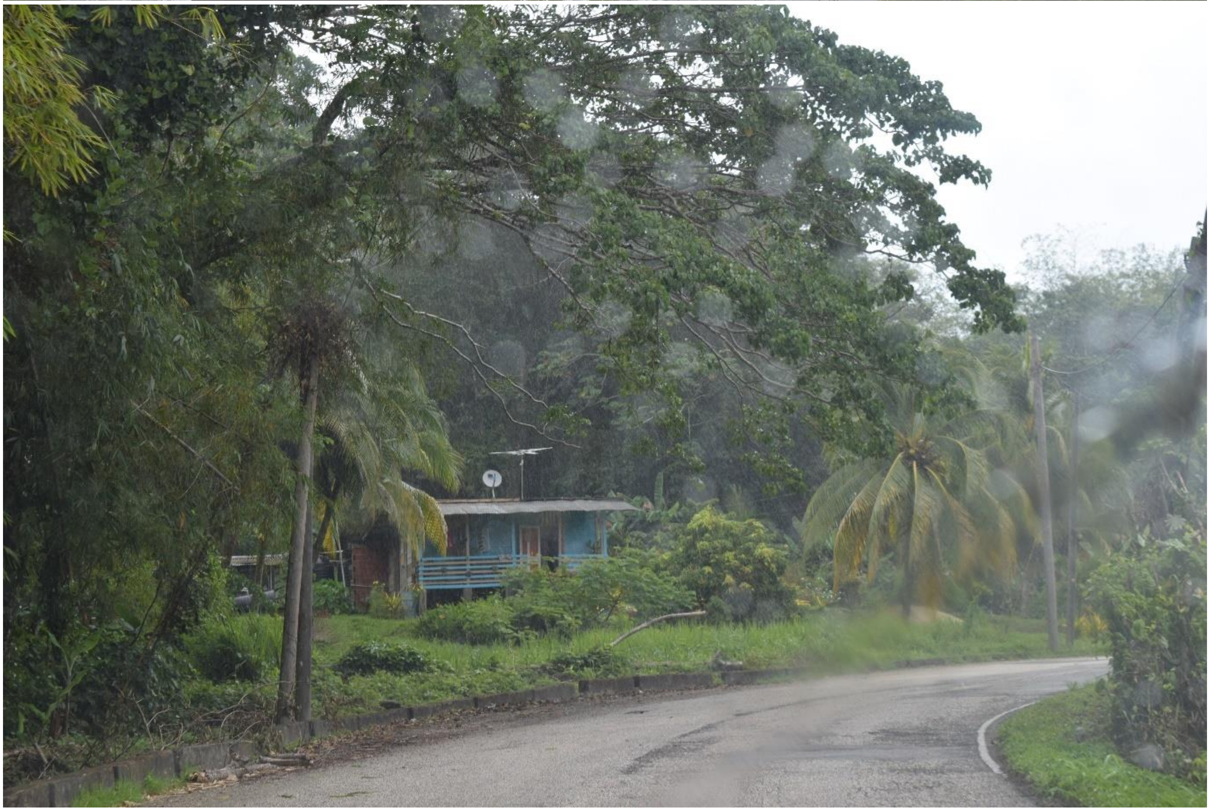

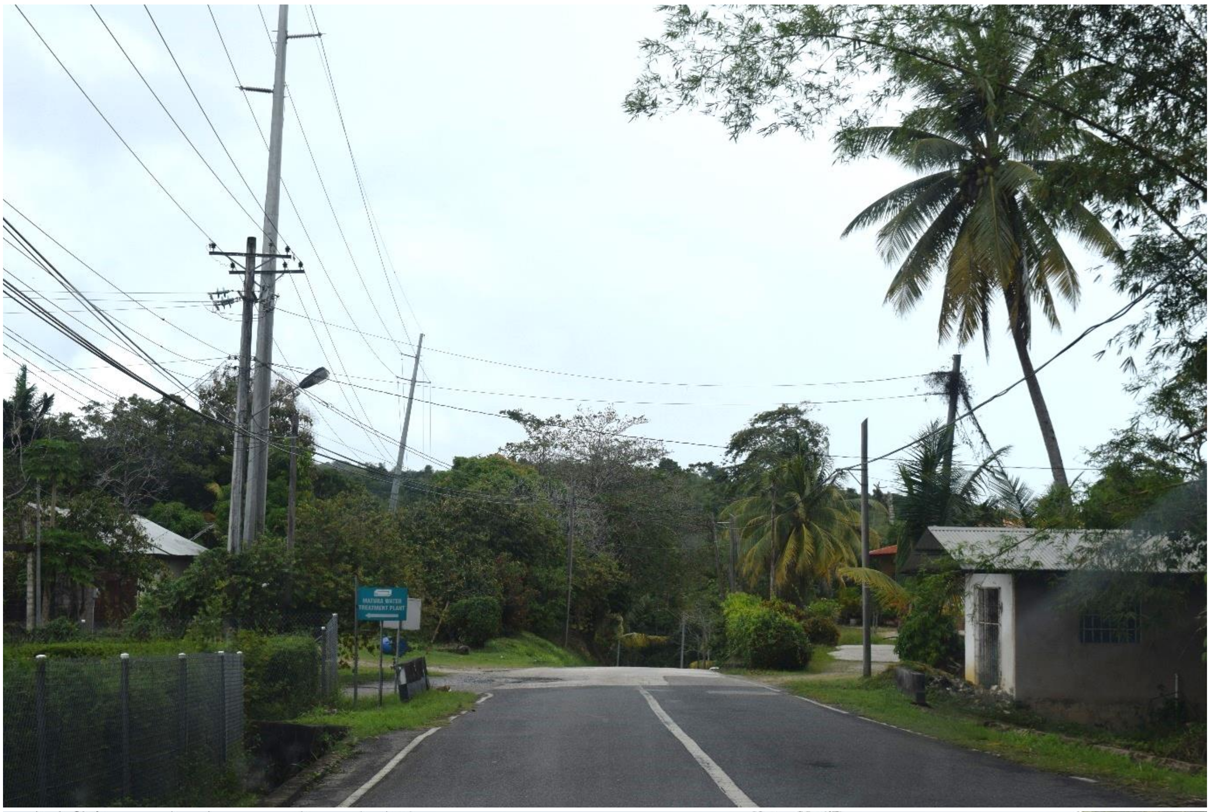
MATURA WATER
TREATMENT PLANT

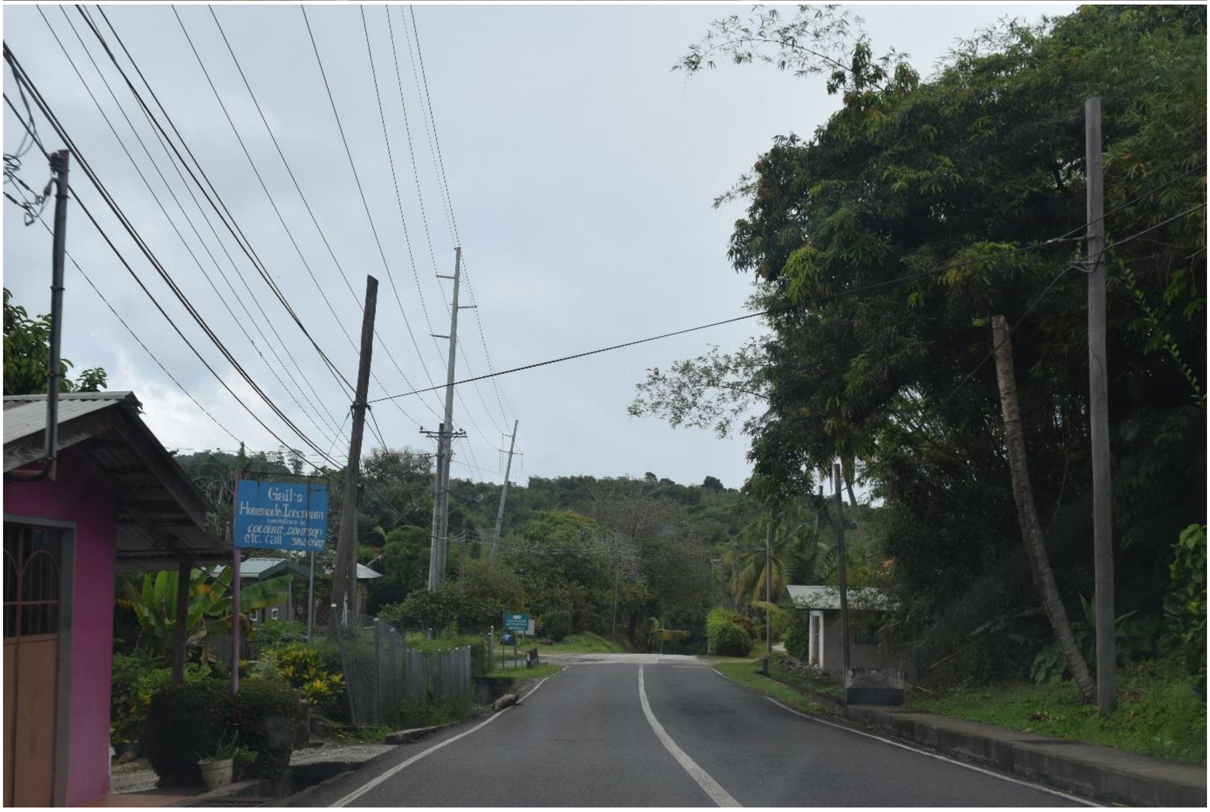
Gail's
Homemade Icecream
coconut, soursop
etc. call

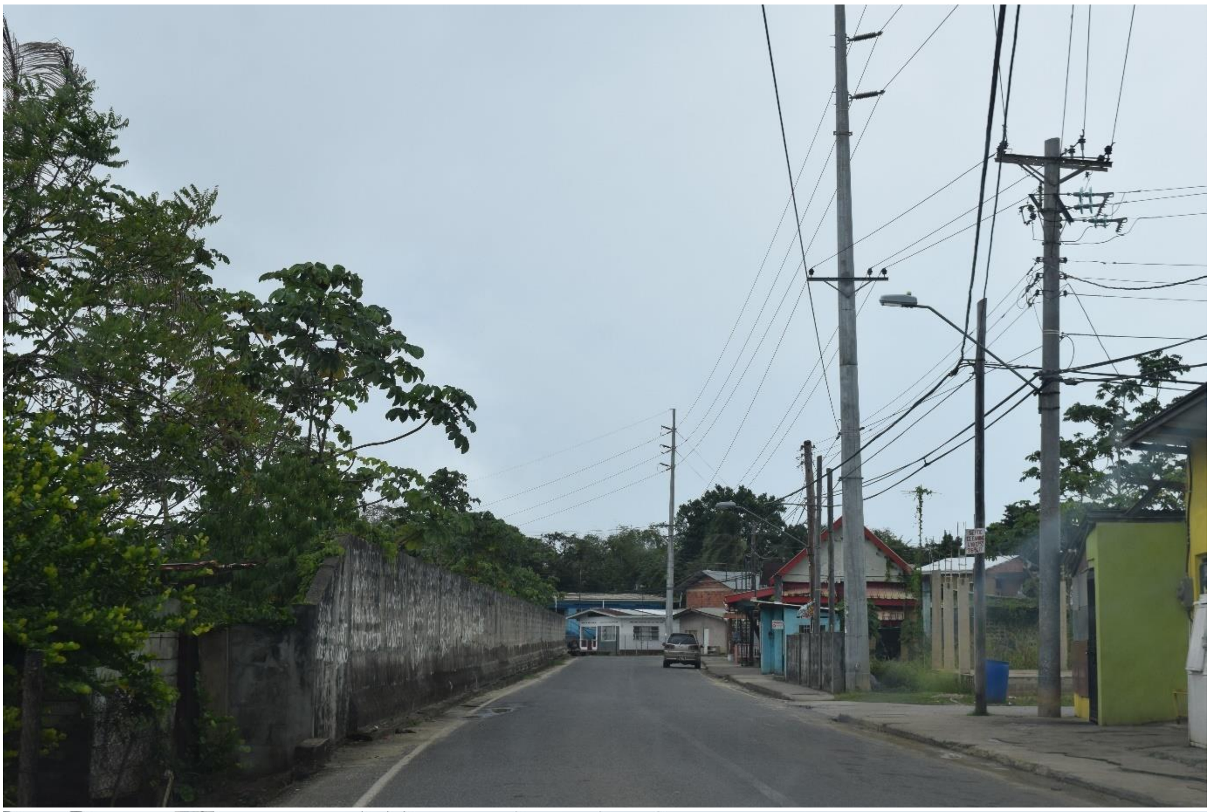

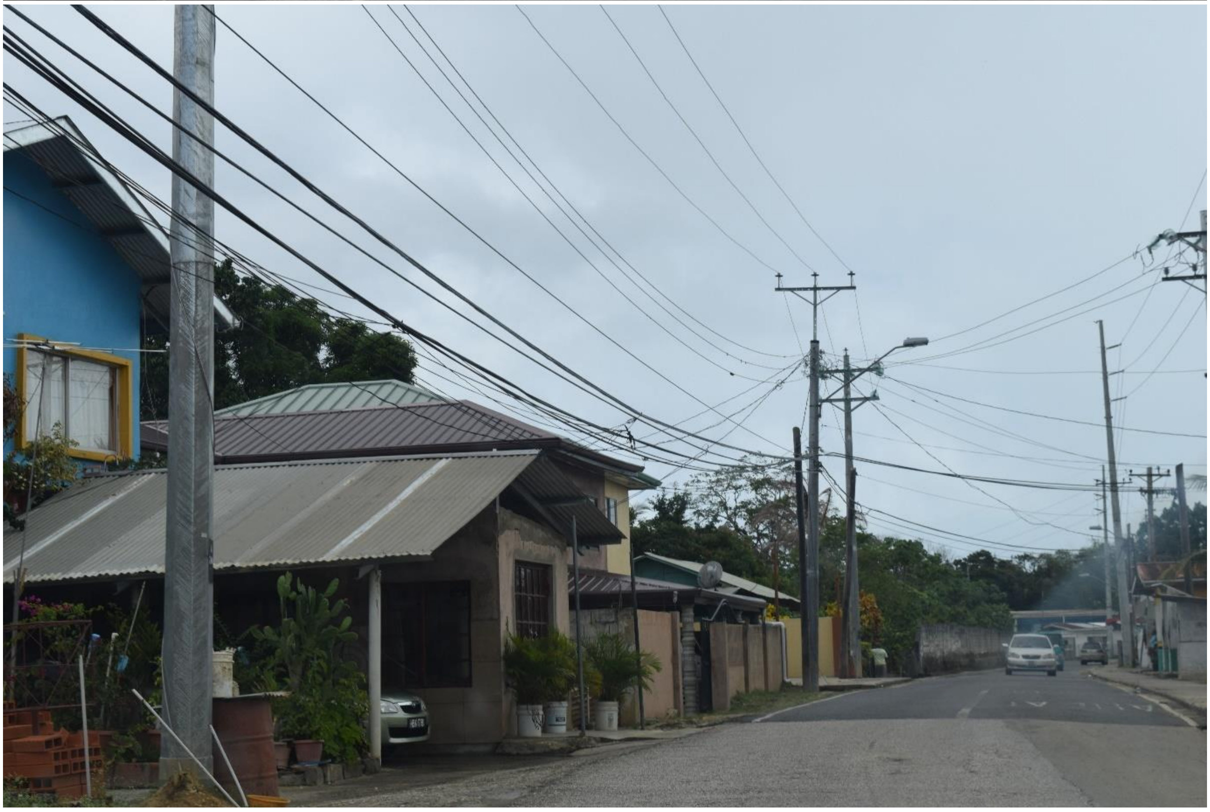

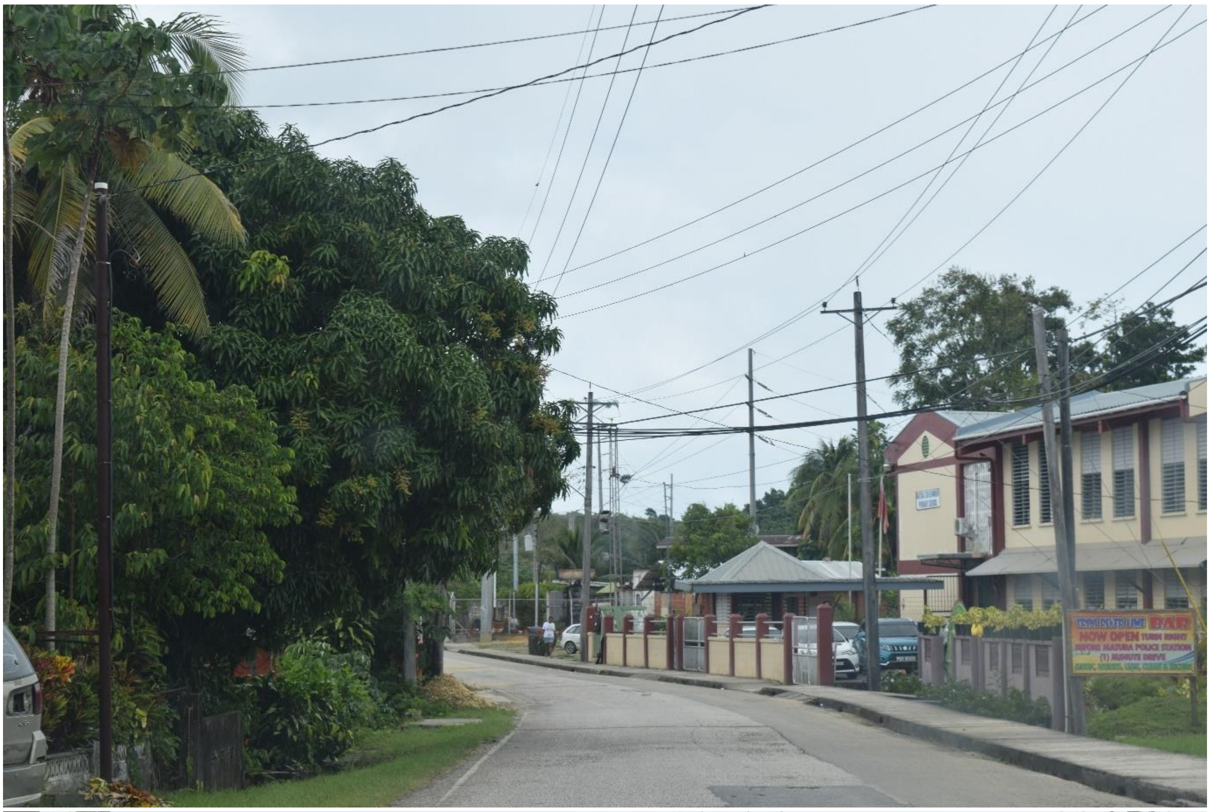
BAR
NOW OPEN
(1) MINUTE DRIVE

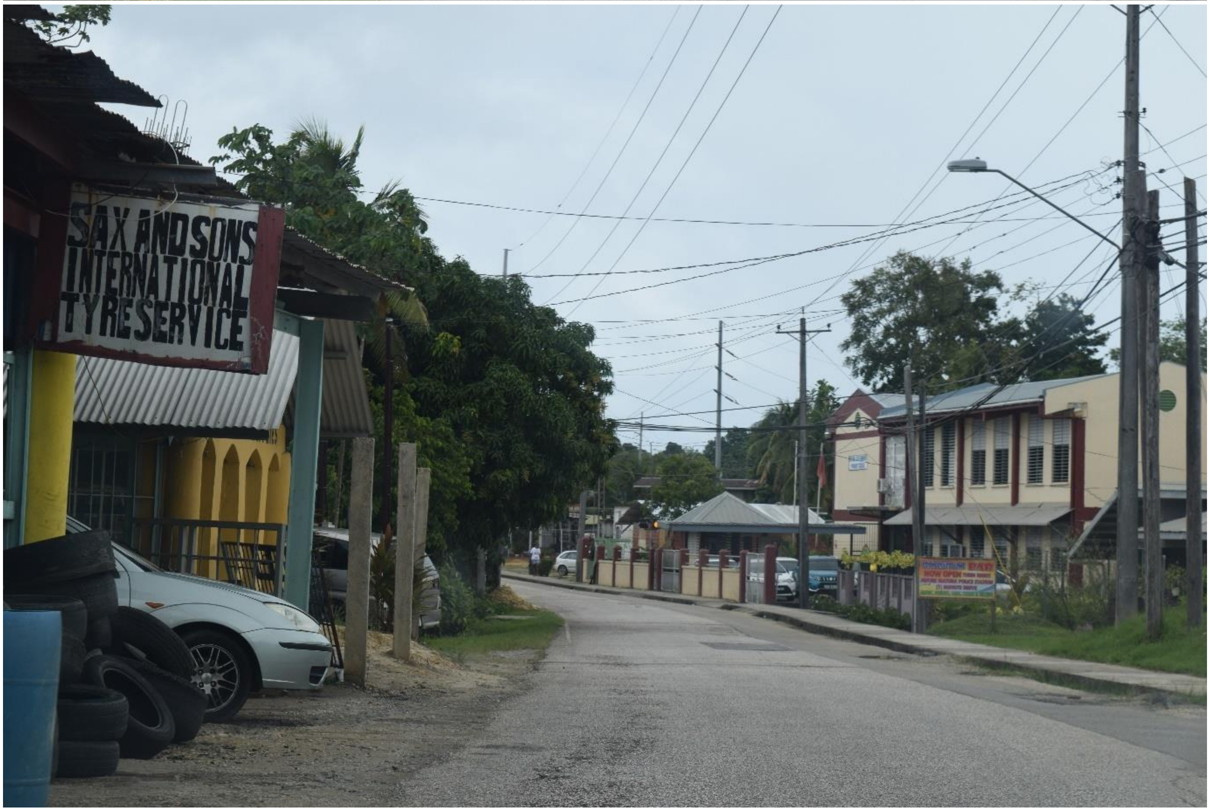
SAX AND SONS
INTERNATIONAL
TYRESERVICE

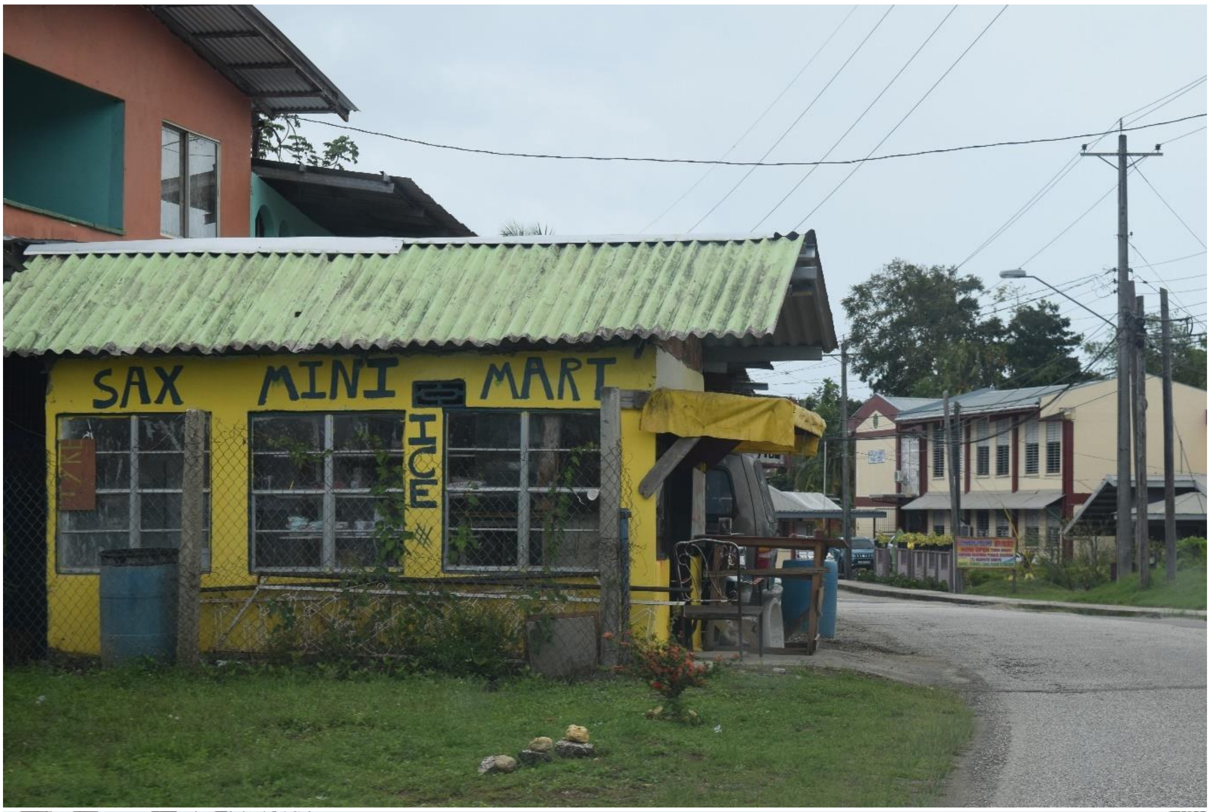
SAX MINI MART
ICE

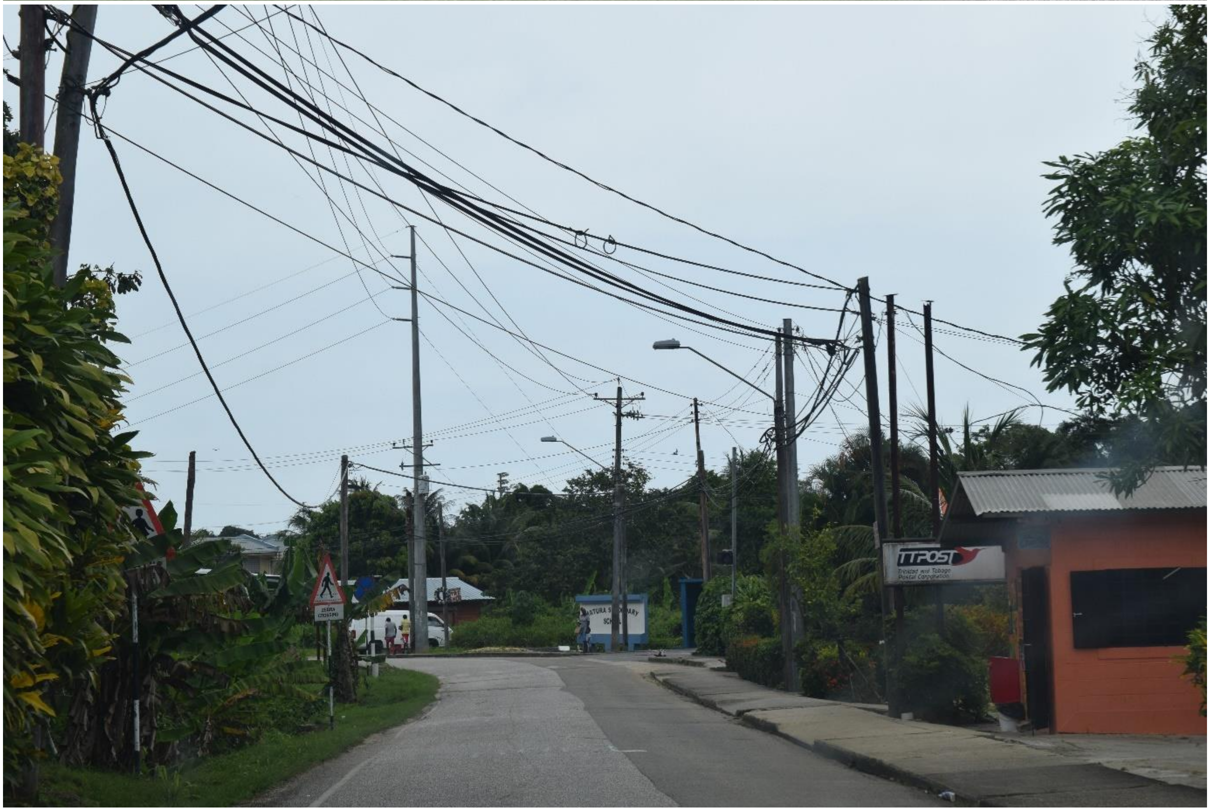
TTPOST

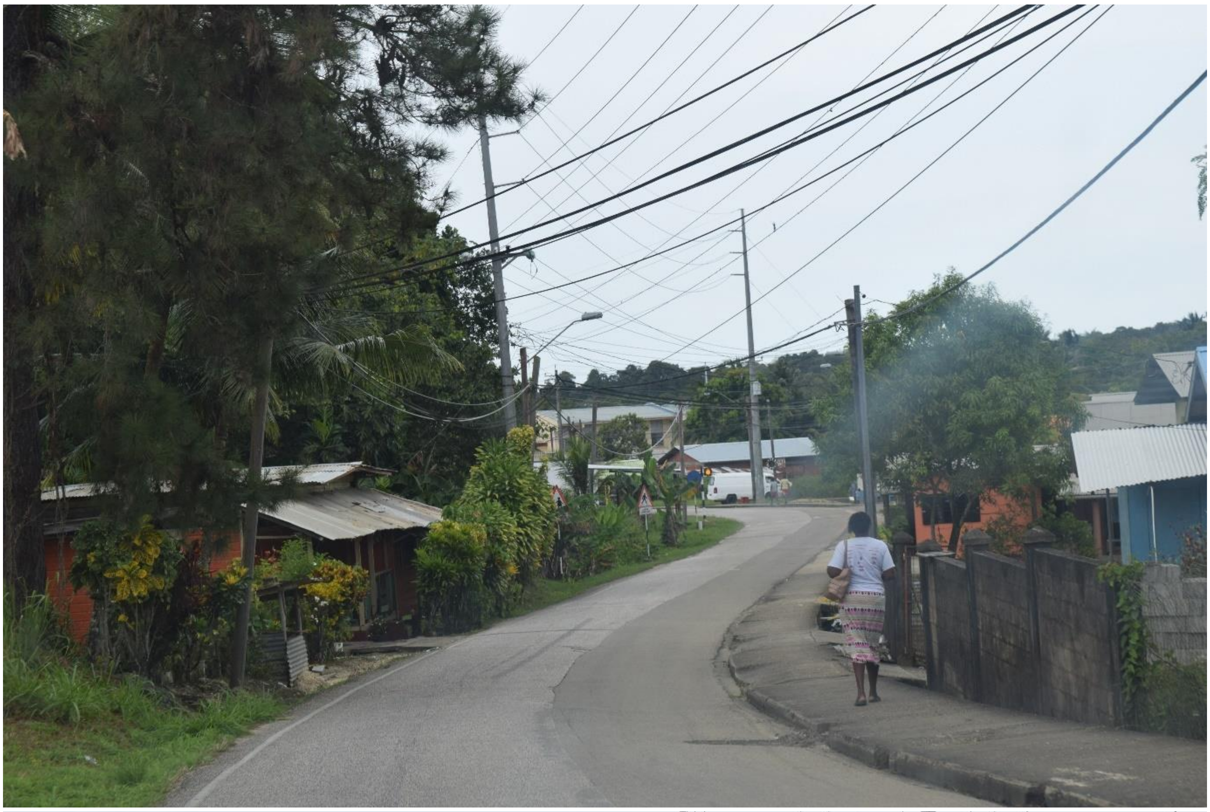

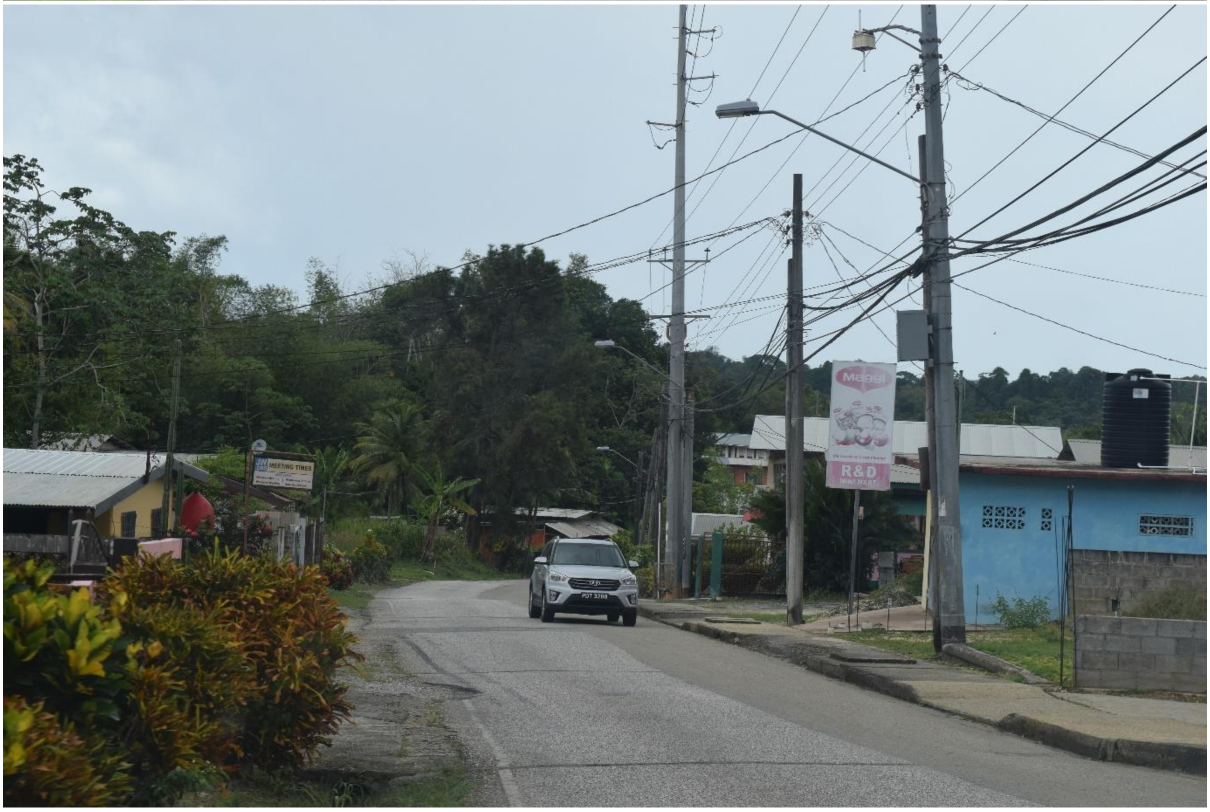
MEETING TIMES
Maggi
R&D

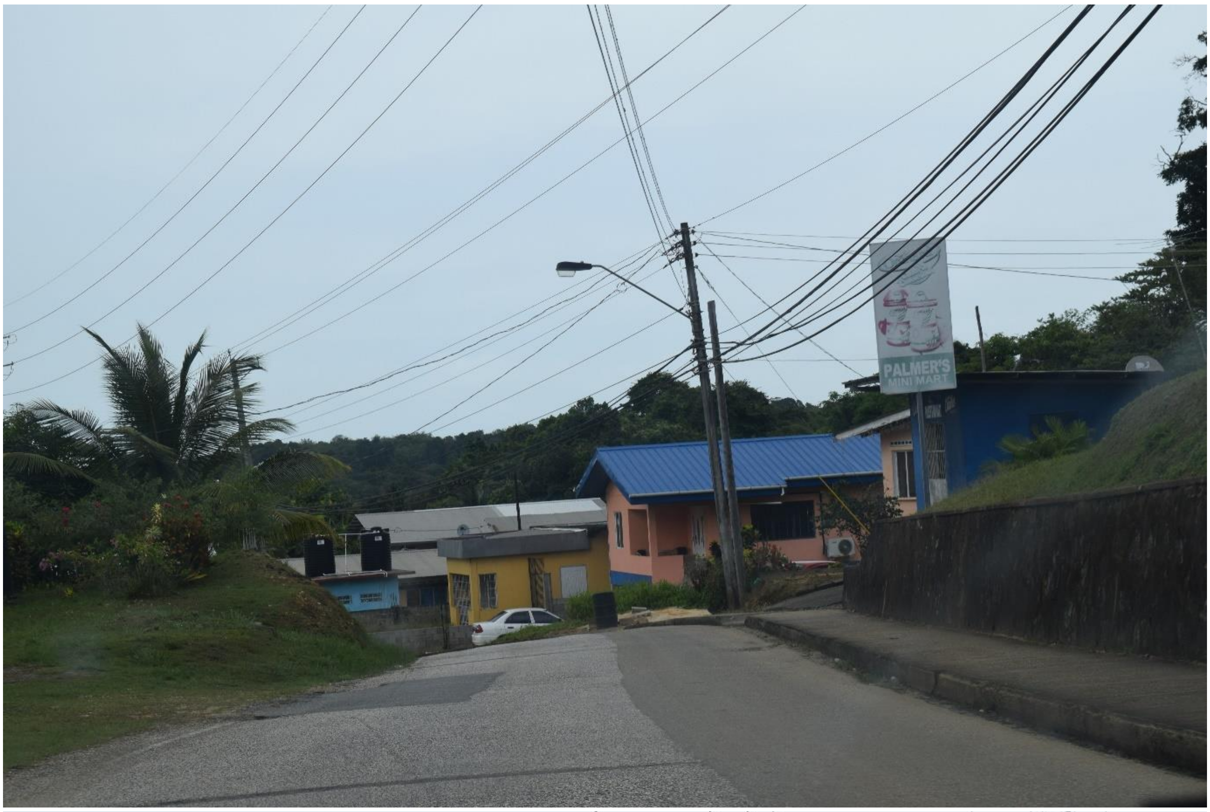
PALMER'S
MINI MART

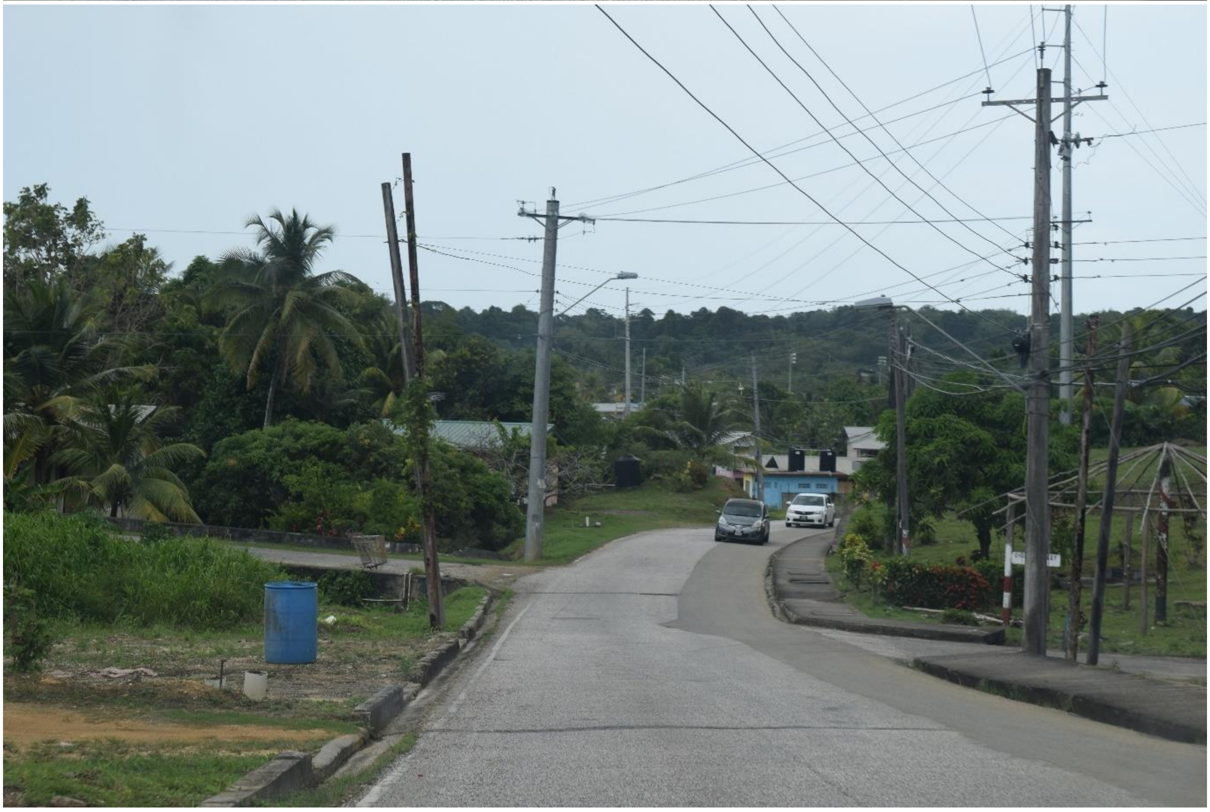

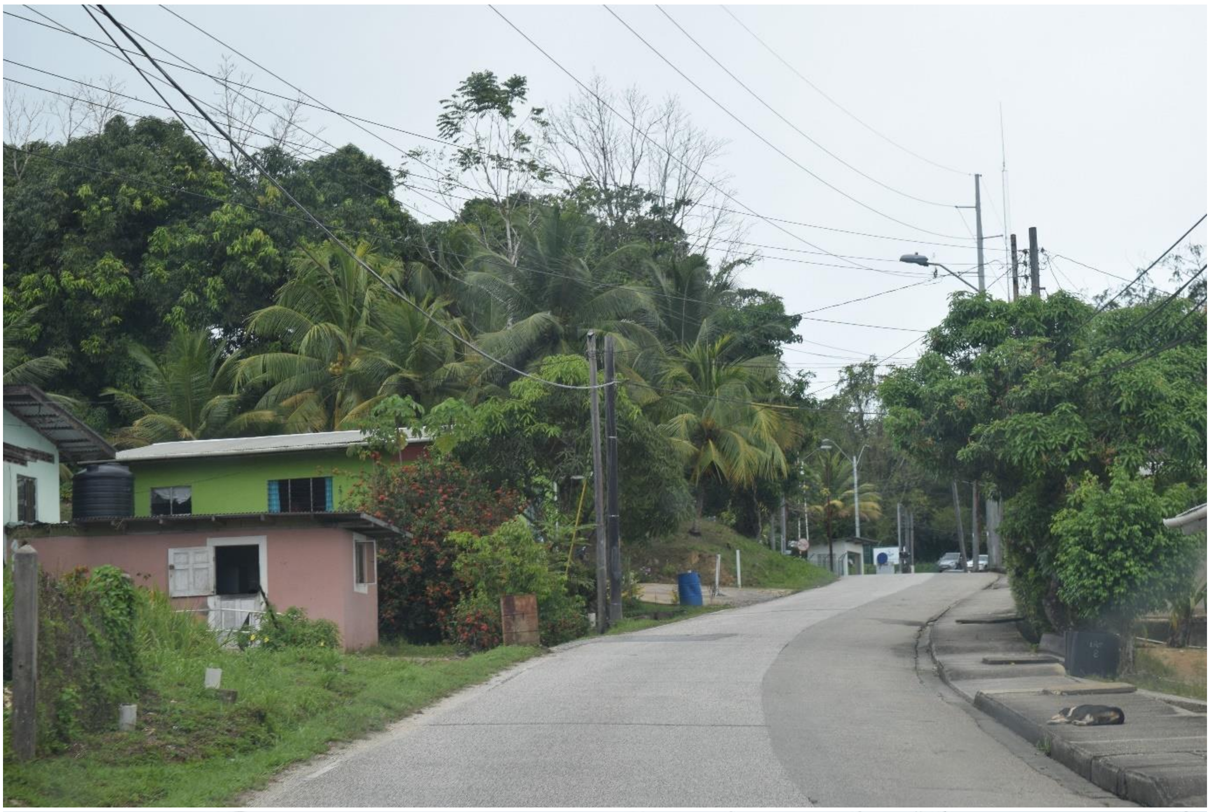

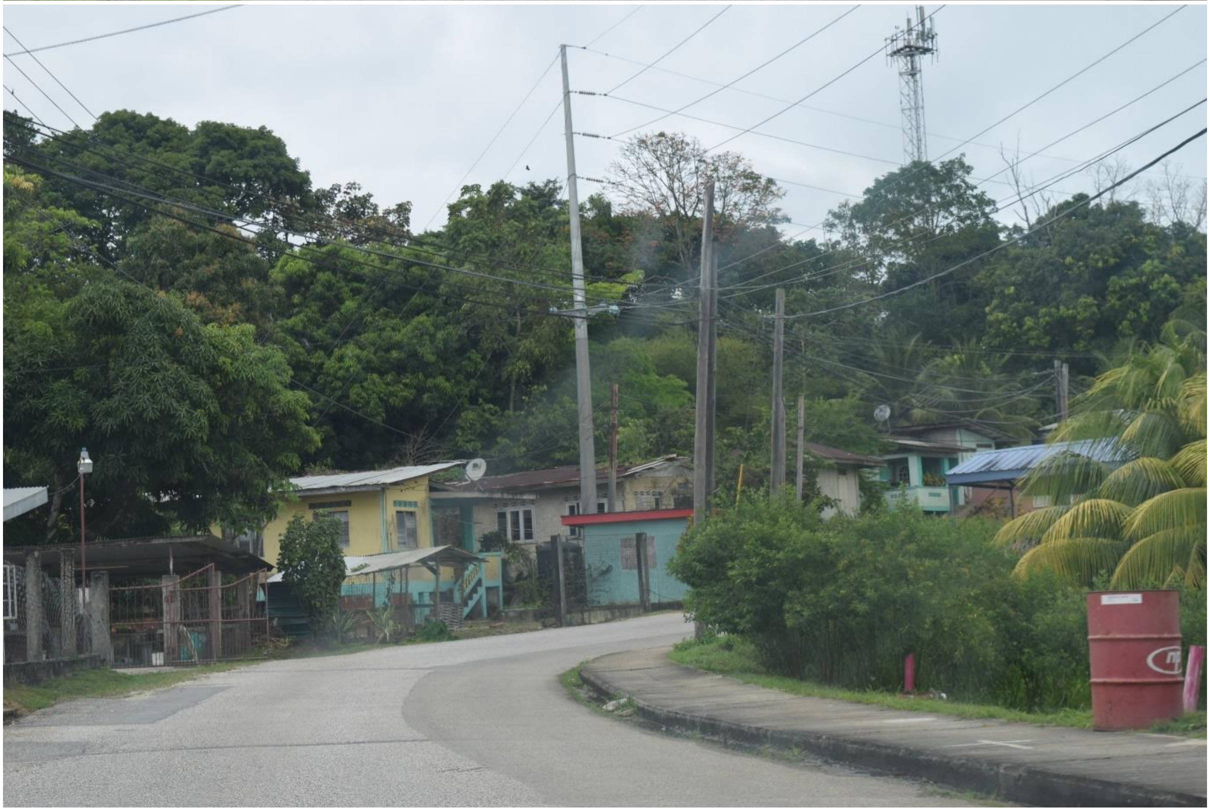

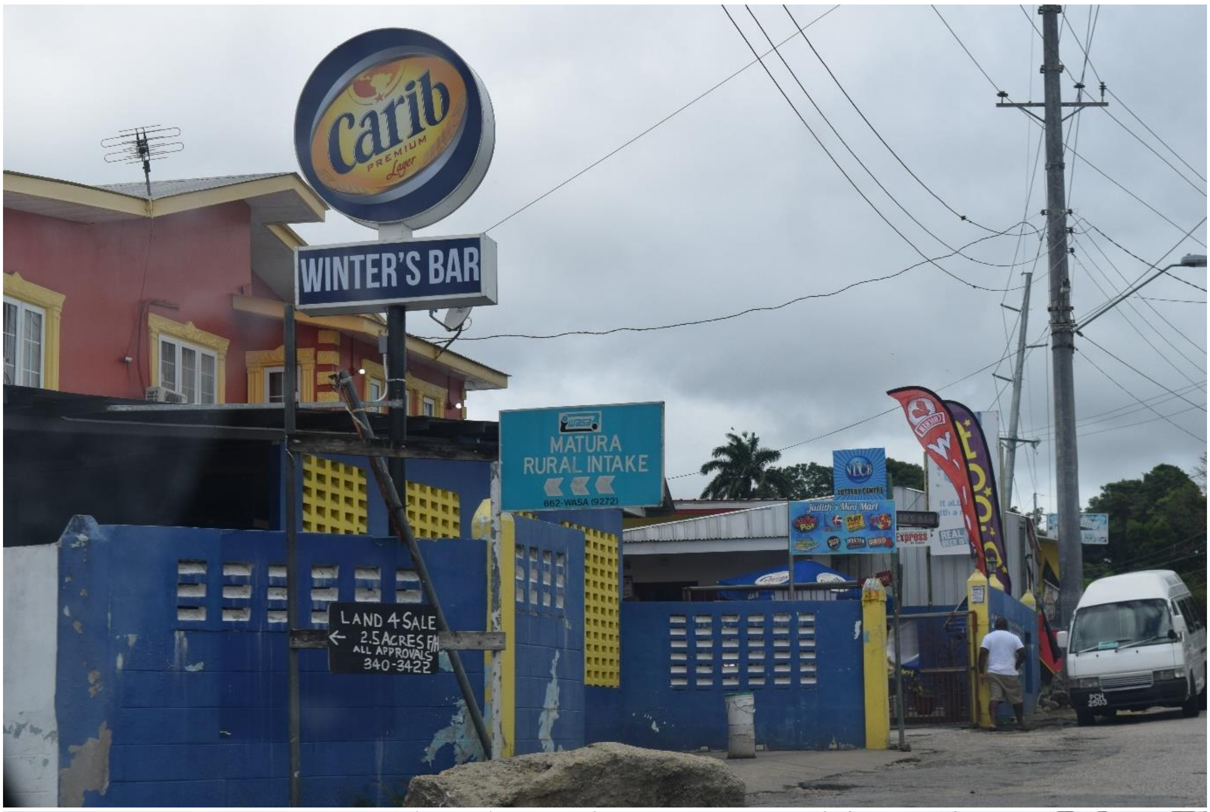
Carib
PREMIUM
Lager
WINTER'S BAR
MATURA
RURAL INTAKE
662-WASA (9272)
LAND 4 SALE
2.5ACRES
ALL APPROVALS
340-3422

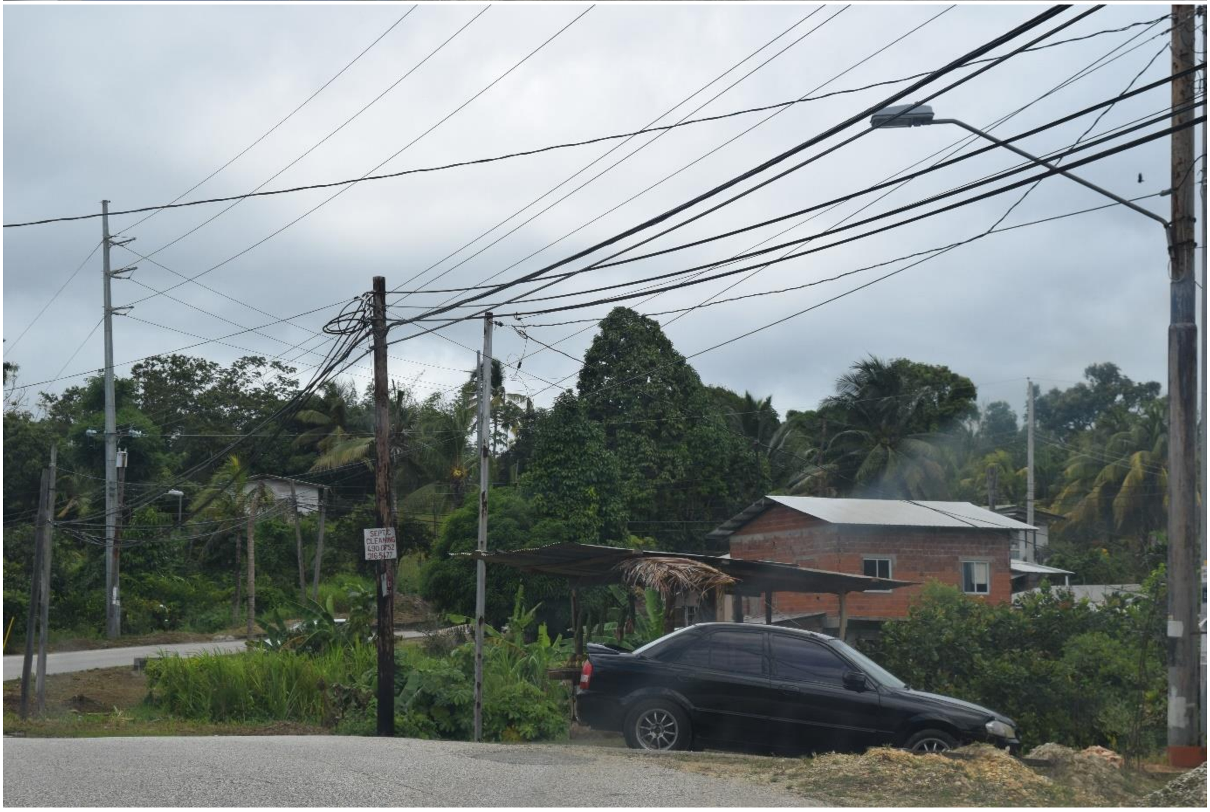

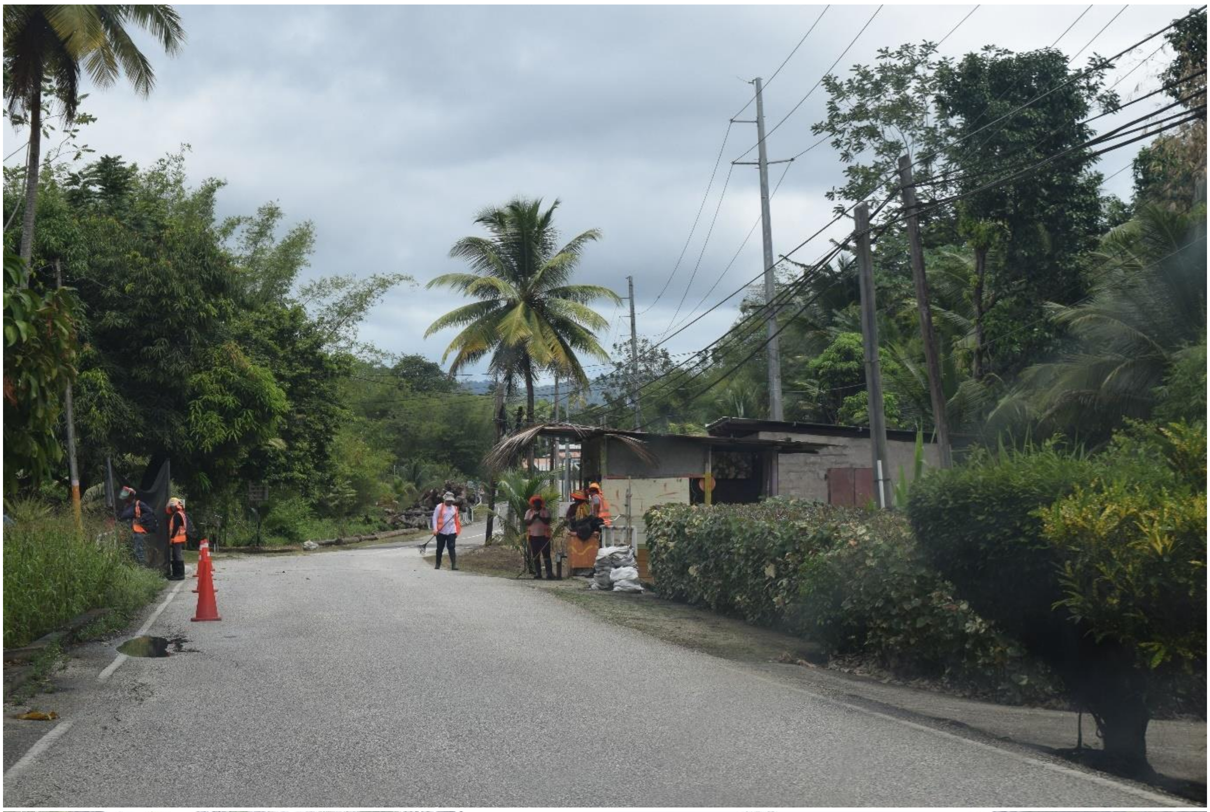

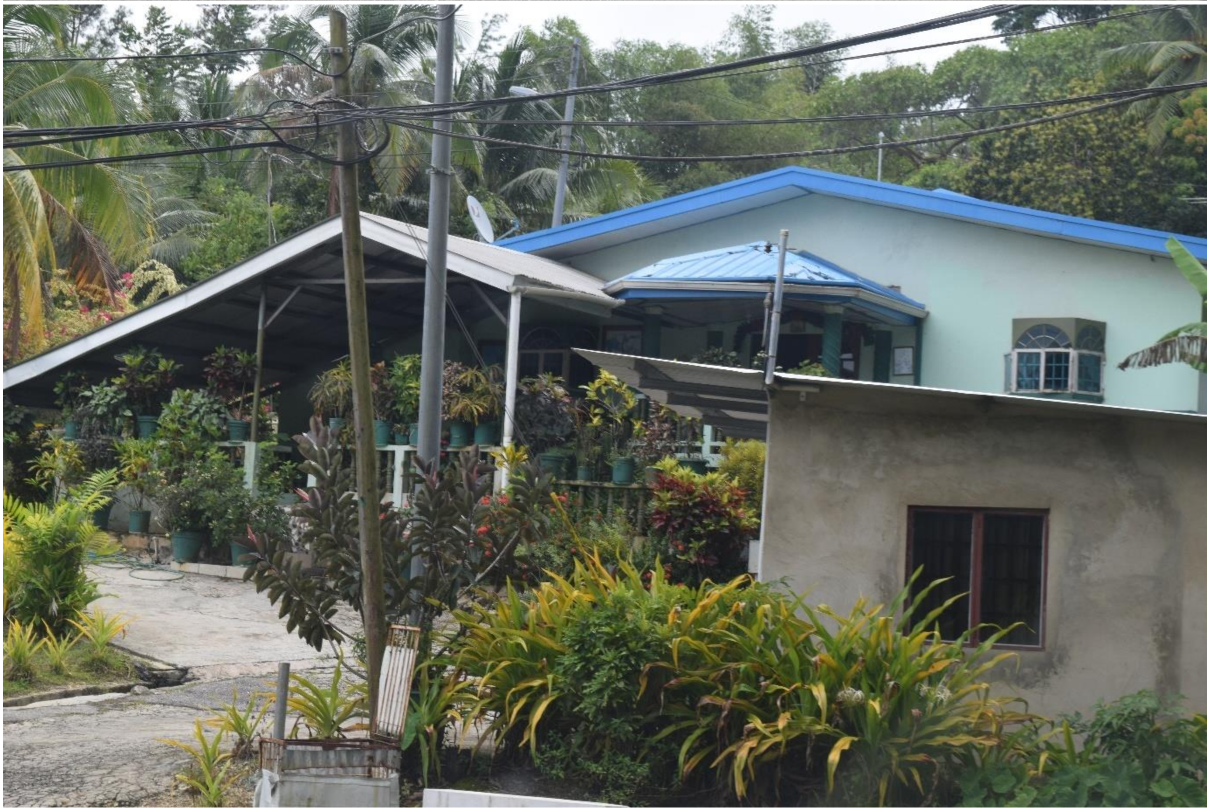

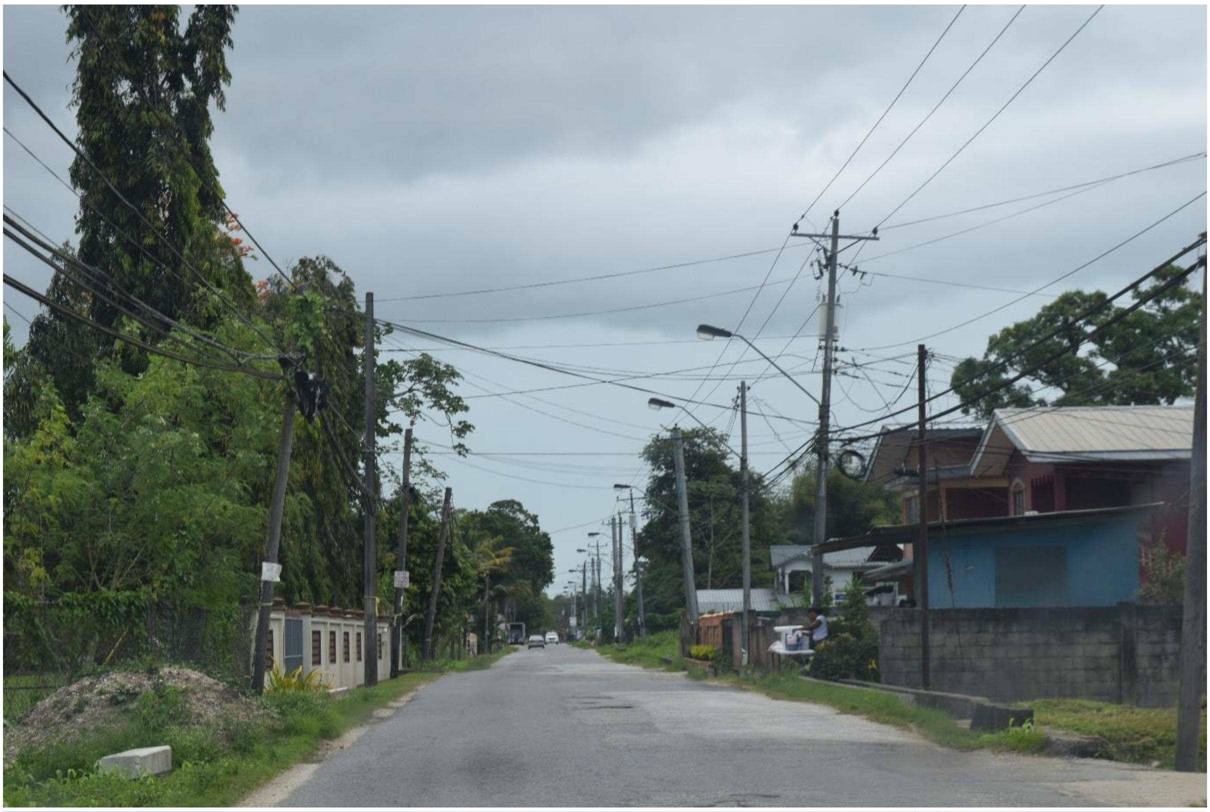

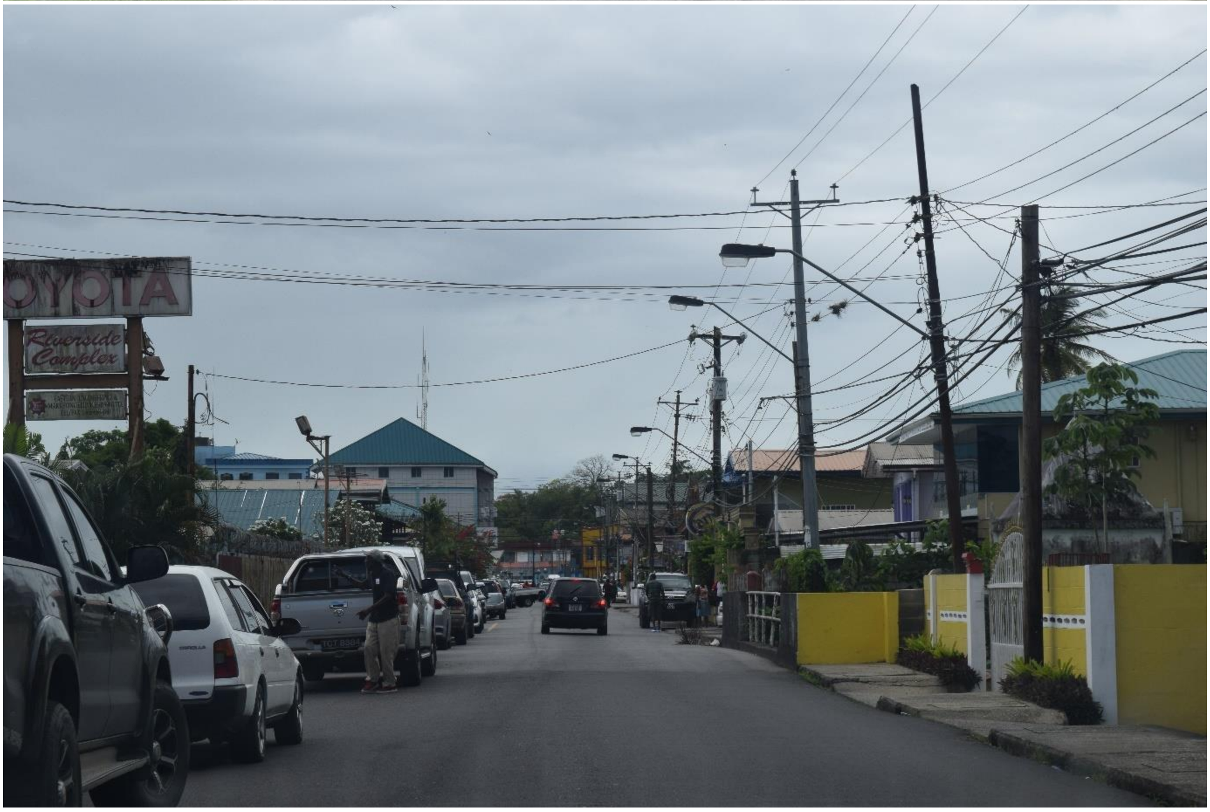
OYOTA
Riverside Complex

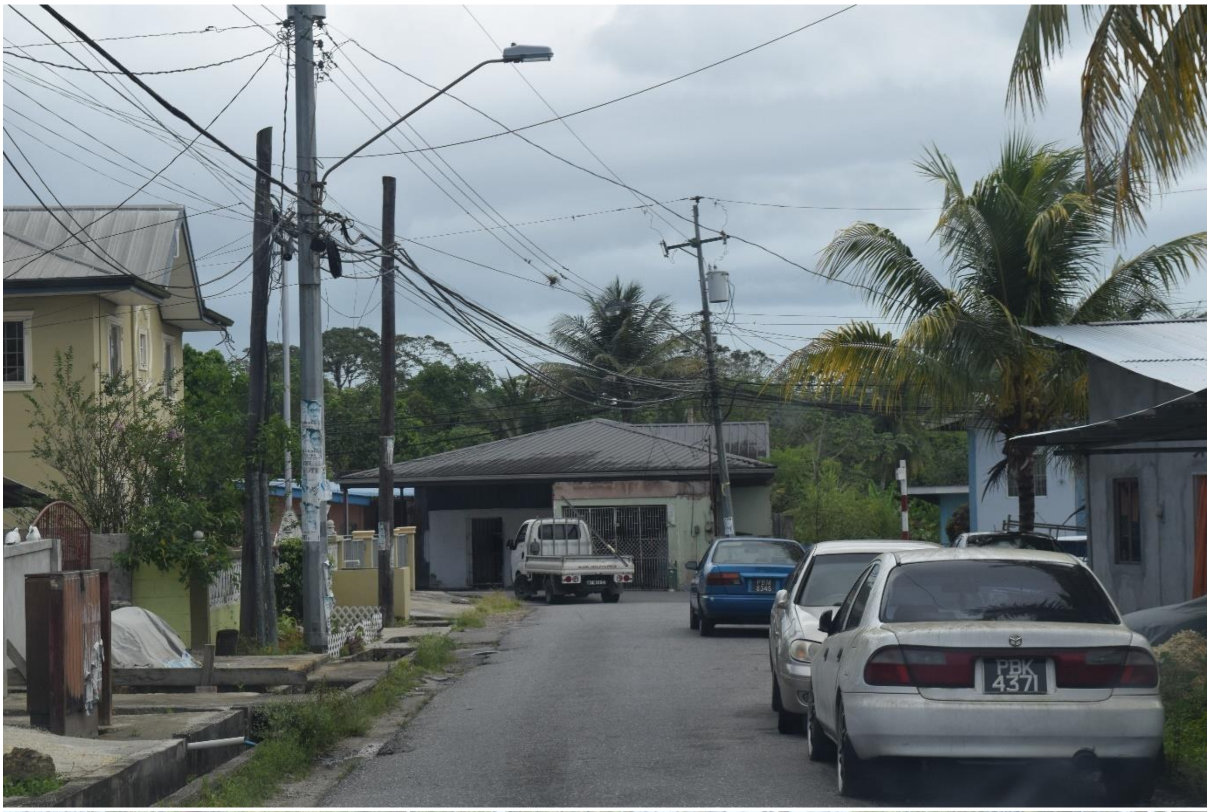
PBK
4371

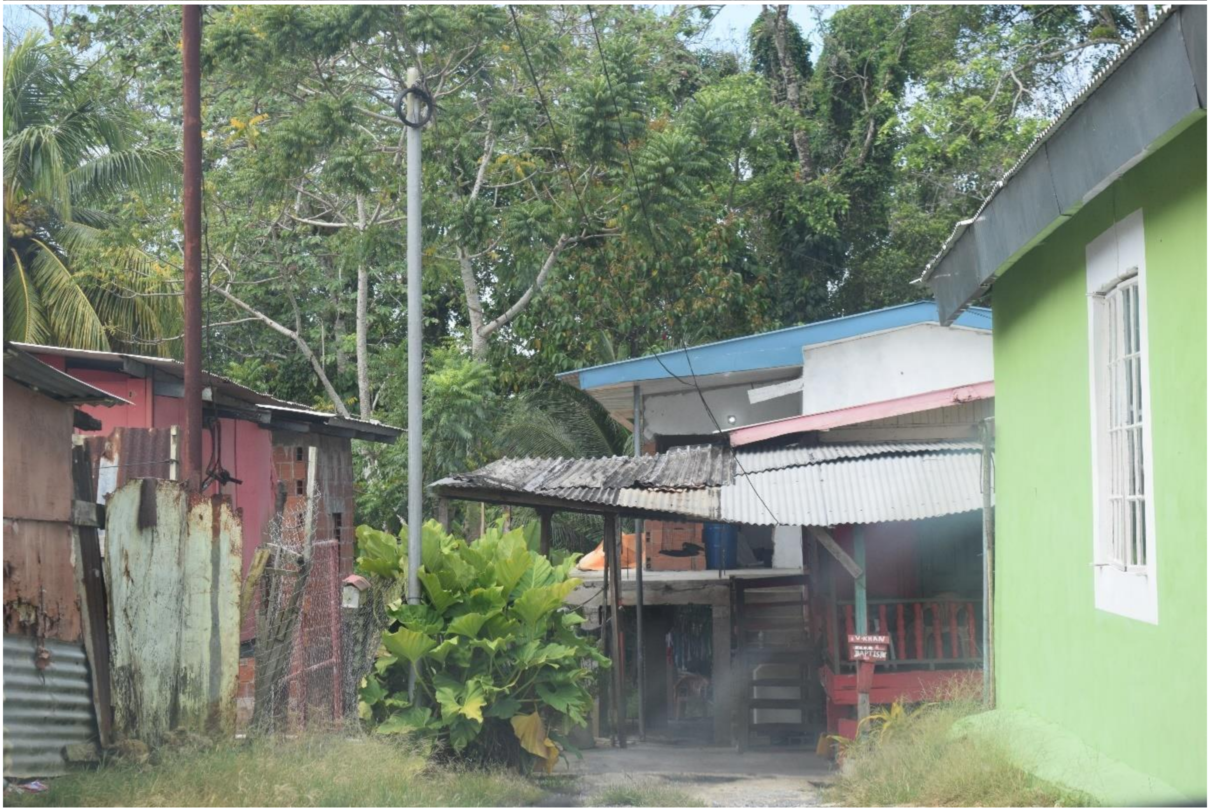

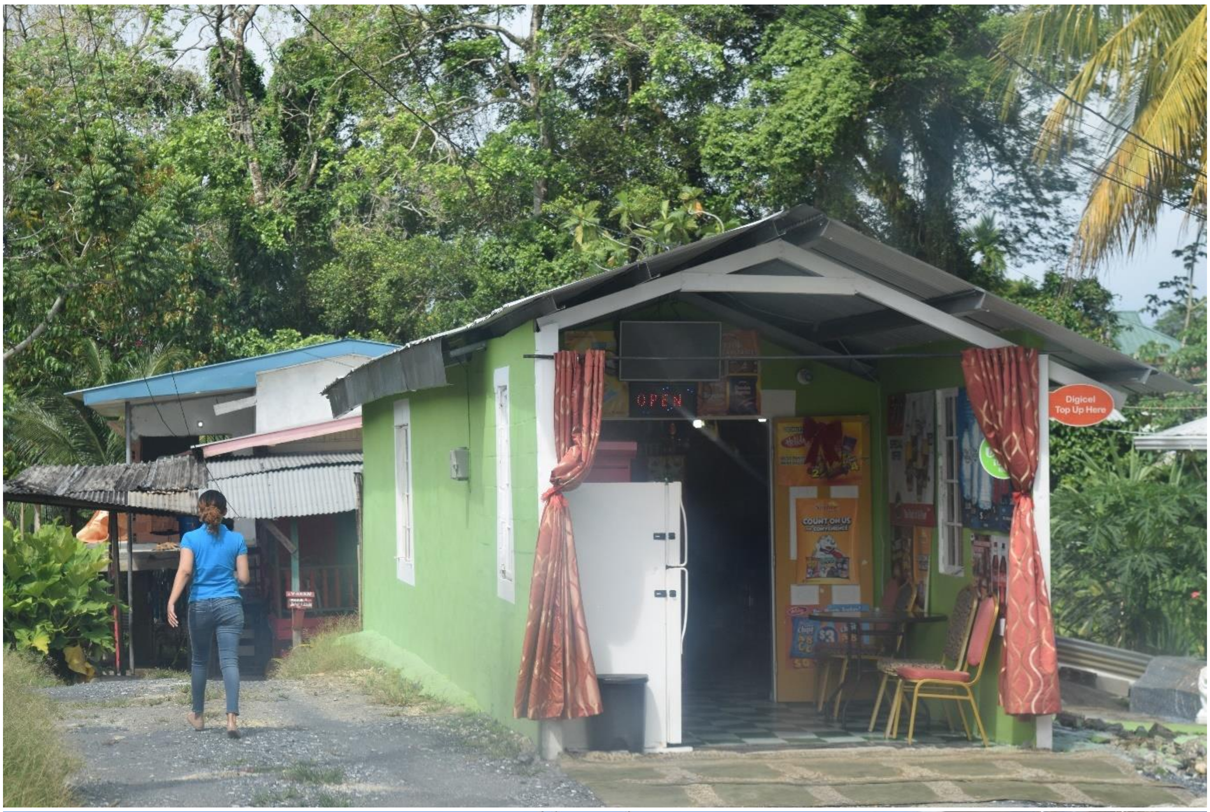
OPEN
Digicel
Top Up Here
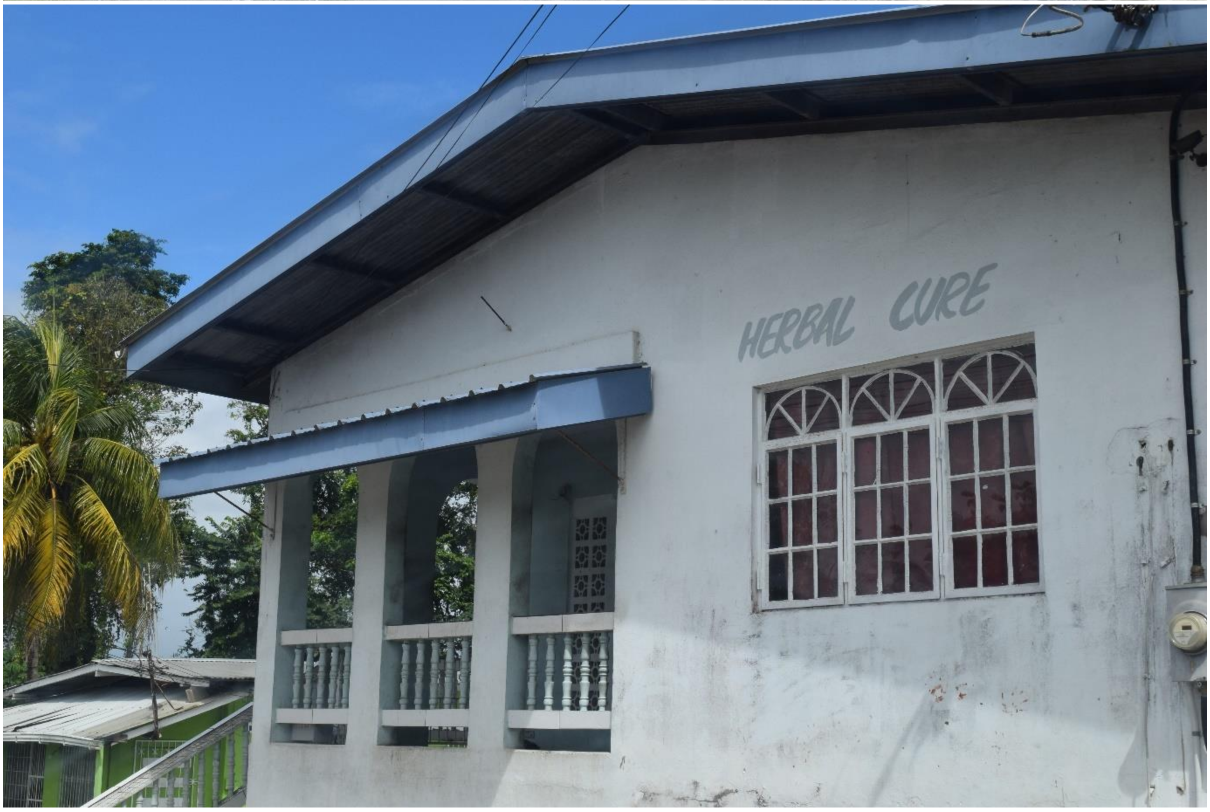
HERBAL CURE

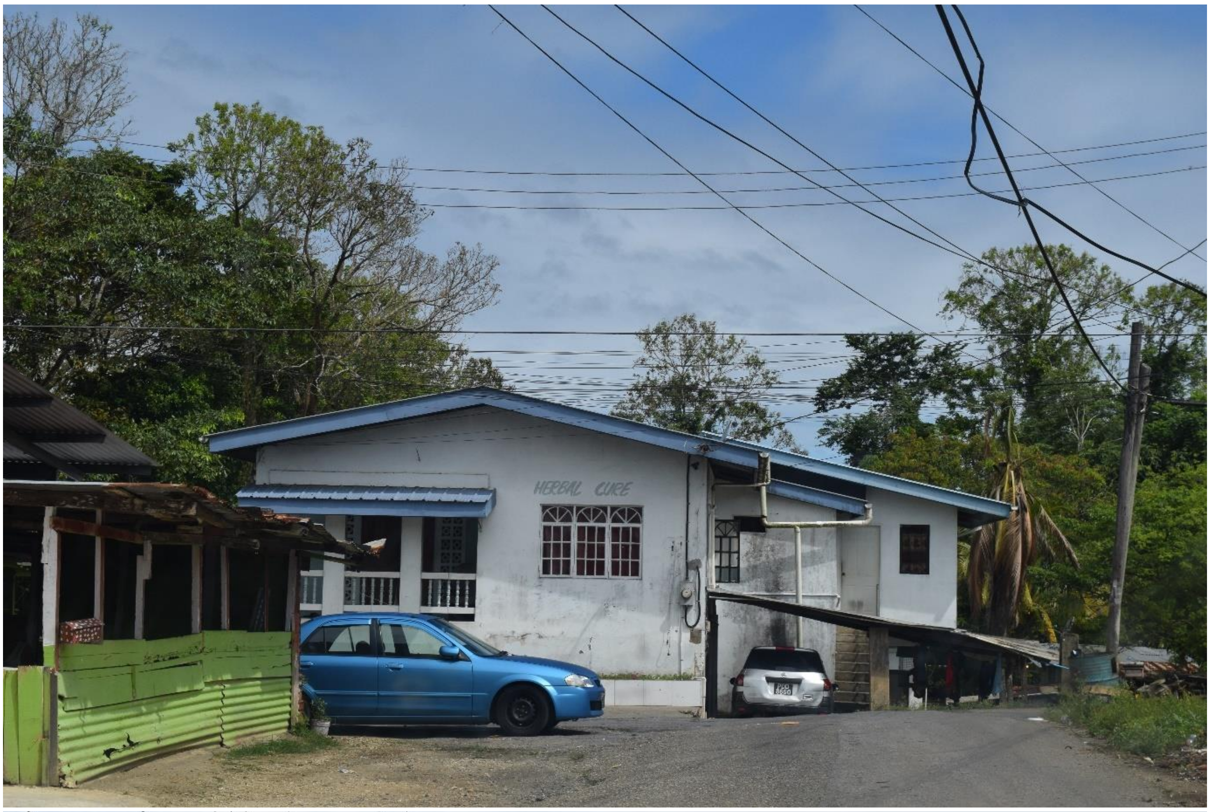
HERBAL CURE

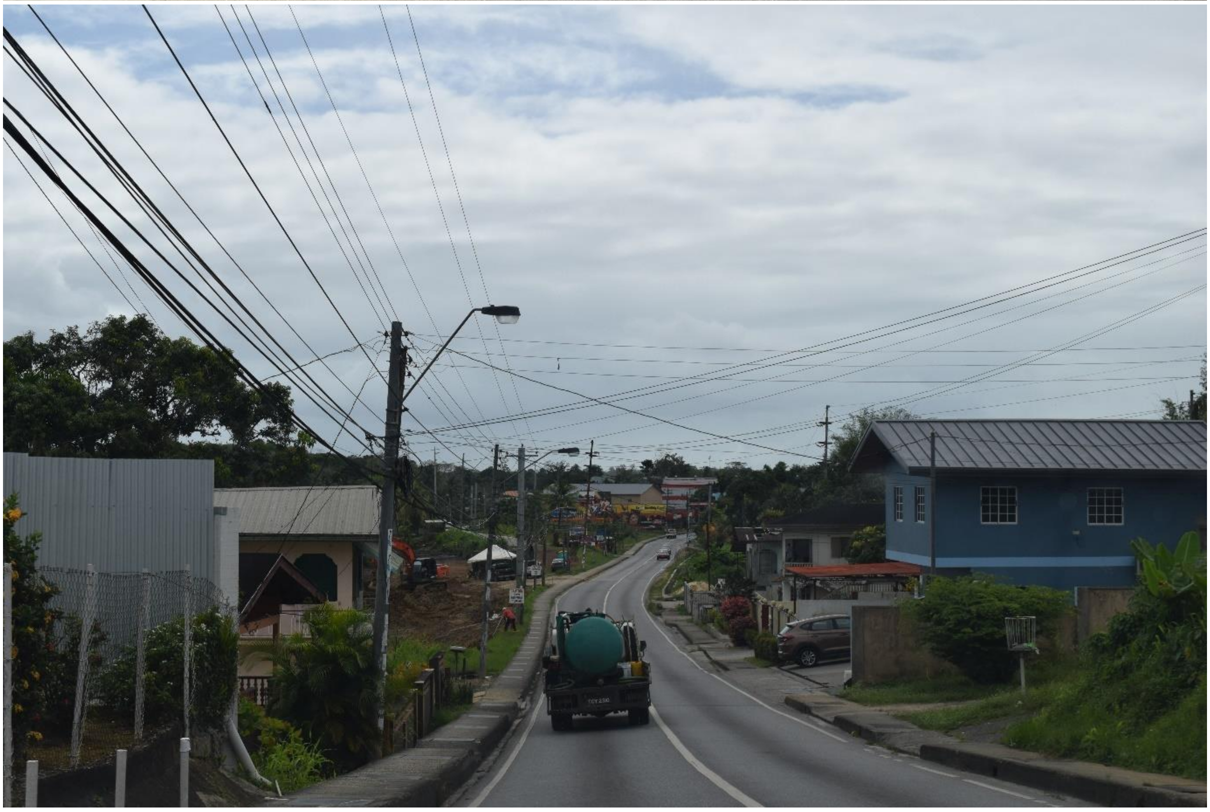

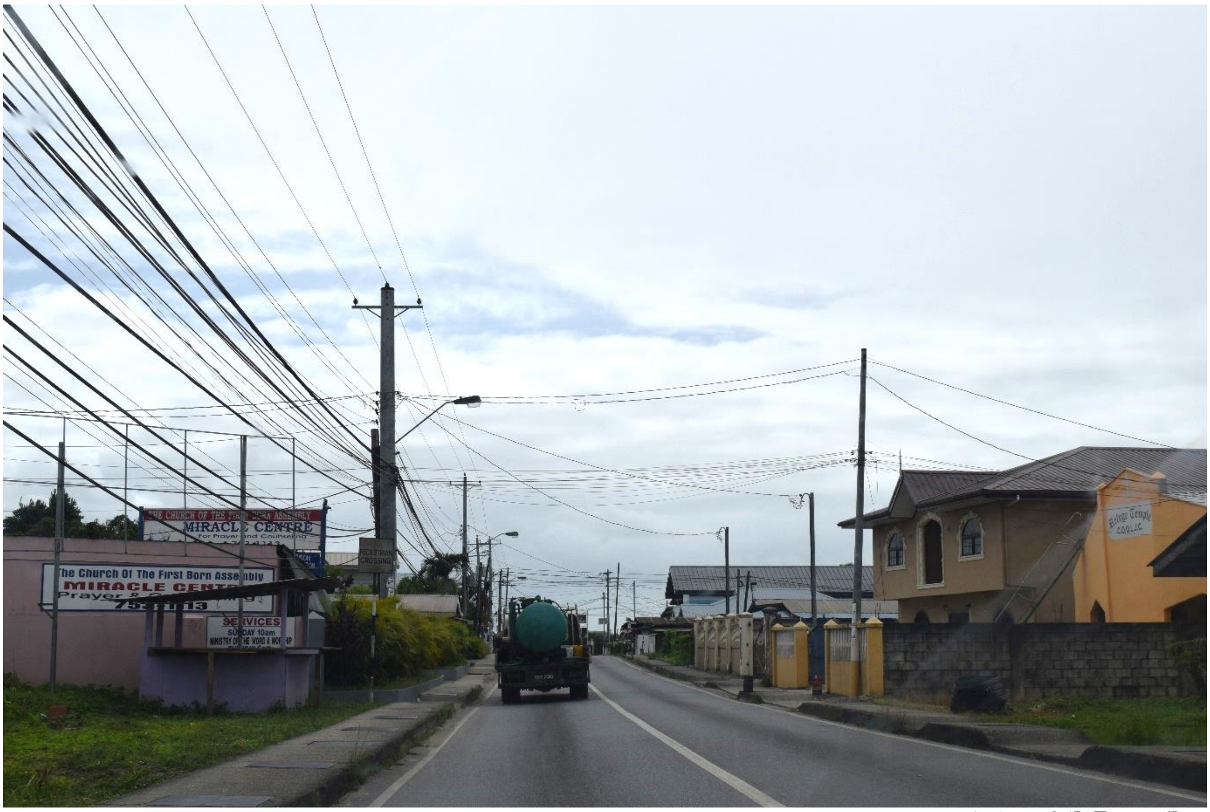
The Church Of The First Born Assembly
MIRACLE CENTRE
SERVICES
SUNDAY 10am

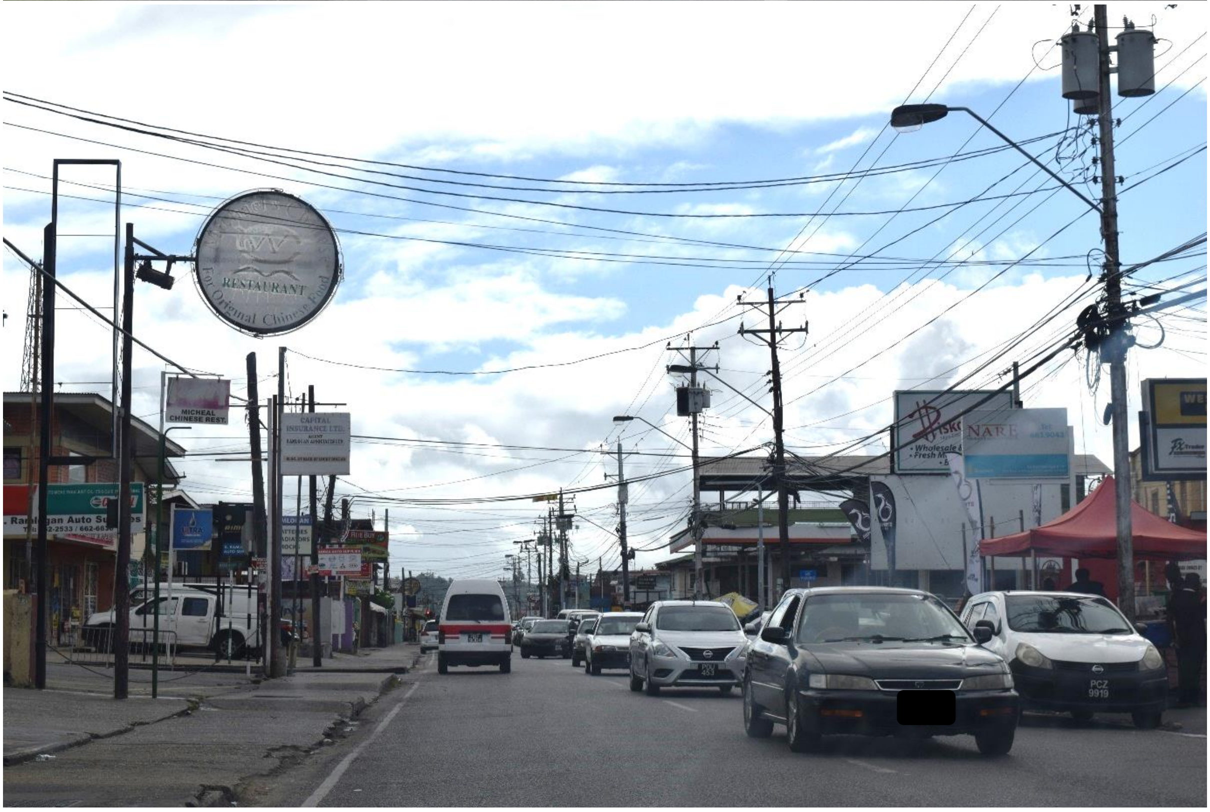
RESTAURANT
For Original Chinese Food
MICHEAL CHINESE REST.
CAPITAL INSURANCE LTD.

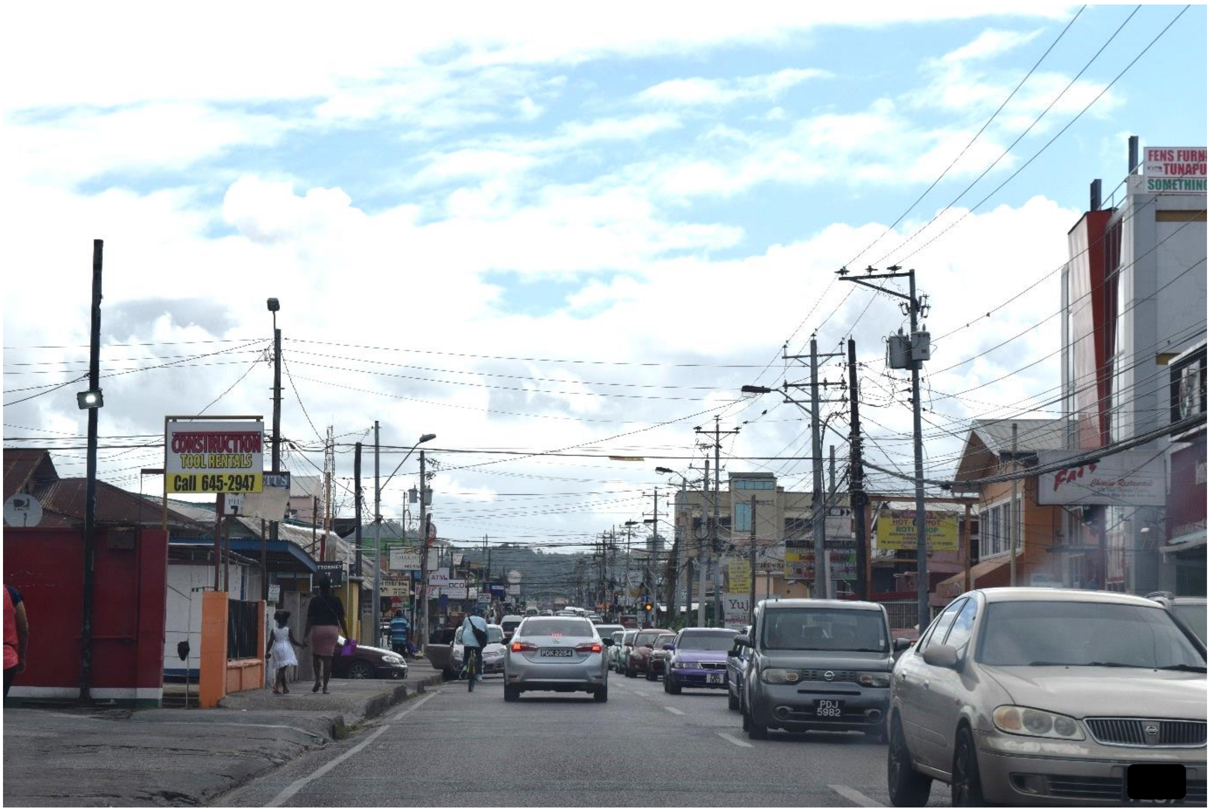
CONSTRUCTION
TOOL RENTALS
Call 645-2947
