# Supplementary material for: Neighbourhoods & recovery from psychosis in Trinidad: A qualitative study
Source: SSM Qual Res Health. 2024 Jun;5:100373. doi: 10.1016/j.ssmqr.2023.100373 (PMC11190840; doi:10.1016/j.ssmqr.2023.100373)
Supplement: Multimedia component 3 [file mmc3.docx]

**Report on catchment area in Trinidad and Tobago**

The catchment area consists of Diego Martin, Port of Spain, San Juan/Laventille, Tunapuna/Piarco, Arima, Chaguanas, and Sangre Grande.


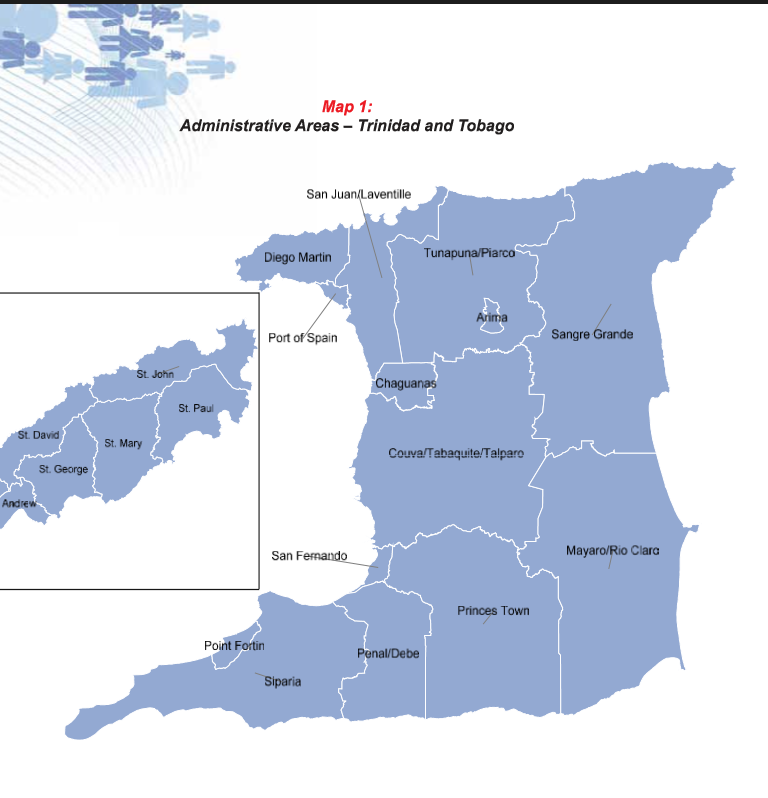


|  | Trinidad and Tobago | Catchment area |
| --- | --- | --- |
| Land mass | 5127 sq Km | Approx. 1000 sq Km |
| Population | 1,328,019 in 2011 (1,213,733 in 1990) | 624,993. 488,813 aged 16 and over |
| Population density | 259 per sq Km in 2011 (236 in 1990) | Approx. 624 per sq Km. Varies by municipality. |
| Sex ratio | 100.7 in 2011 (99.8 in 1990) | Equivalent |
| Age | Proportion of aged persons 9% in 2011 (6.2% in 1990) Median age 32.6 in 2011 (24.1 in 1990) | Equivalent |
| Ethnic composition | East Indians 35.4%, Africans 34.2%, Mixed 22.8% | 43% African, 26% Indian, 30% Mixed |
| Religious composition | Roman Catholic 25% , Hinduism 20%, Pentecostal 14% | 34% Roman Catholic, 13% Hindu, 13% Pentecostal |
| Education | Educational attainment – 29.8% of population attained primary education, 43.5% secondary, 6.2% tertiary. 38.7% of population aged 15 and over had no qualifications. | Equivalent |
| Fertility | Fertility rate 2.5 in 2011 (4.1 in 1990) | Equivalent |
| Household size | Average household size 3.3 in 2011 (4.1 in 1990) | Varies by municipality |

Data is taken from 2011 Census

**Metro Port of Spain**

- Metro POS encompasses San Juan/Laventille, POS and Diego Martin.
- Metro POS has a population of 312,045 and encompasses 8% of land area and 27% of the country’s population.
- Metro POS population distributed as such: 14.2% in SJ/L, 8.7% in DM and 4.7% in POS

**San Juan/Laventille**

Characterized by low household income, high unemployment, high crime, overcrowding, informal hillside settlements and squatting and an inadequate provision of amenities

- Land area of 220.4 km/238.6
- Population 156,966 persons/ 157,295
- 5 major sub-areas – Laventille/East POS, East West Corridor, Greater POS, Northern Range Interior, North Coast
- Heavily urbanised with exception of Caroni Swamp, North Coast and Northern Range
- There are several informal settlements on hillsides
- Population growth rate between 1990-2000 is10.8% compared to national of 4%. Is 0 between 2000-2011 compared to national of 0.5%.
- Adolescent fertility rate is among the country’s highest.
- Second highest rate of female headed households.
- Although it has a lower density (7 persons per hectare/659 per sq km) than POS(36.5) and Diego Martin(8.3) this is partially due to 76% of SJ/L being protected as Forest Reserve.
- Land use - 9% agricultural, 12% residential, 1% institutional, 1% industrial, 1% commercial, 0 recreational, 76% forest reserve
- High unemployment rate of 11% compared to nation of 10.2%, POS 5.6%, DM 2.6%. Unemployment is much higher for women (16.2% compared to national 12.7%). Ranks highest for gender inequality in the nation.
- Higher proportion of lower income families. Household income per capita is $6574.
- Percentage of population with chronic illness is 23.2%
- Significant gap in the provision of amenities and facilities such as schools, recreational grounds and health services.
- Educational attainment – primary and secondary 94.4%, secondary and higher is 65.7%
- Multidimensional Poverty Index MPI identifies deprivation at the individual level in health (child mortality and nutrition), education (years of schooling and school attendance) and standard of living (asset ownership, dirt floor, electricity, water, sanitation and cooking fuel) MPI score of 0.019 indicated high intensity of poverty and ranks area as 3^nd^ in country.
- Sex and age distribution is equivalent to national averages.
- Ethnicity – 55% African, 19% Indian, 25% mixed, 1% Caucasian/Syrian/Chinese
- Religion 31% Roman Catholic, 11% Spiritual Baptist, 13%Pentecostal. Close to national average.
- Marital status 57% never married compared to national average of 49%.
- Highest overcrowded households in catchment area (% >3 persons per bedroom). This is 8.9% compared to DM 7.3% and POS 7.72%

Areas within SJ/L

Laventille and Morvant

- Underprivileged communities, squatter settlements, tenement yards and public-housing apartments
- High crime rate
- Informal unplanned development

Barataria to Malick

- Largely residential area

San Juan to Petit Bourg

- Major commercial centre
- Residential in Lower Santa Cruz and Petit Bourg

Mount Hope/Champ Fleurs

- Largely residential with industrial activity along Eastern Main Road

Mt. Lambert

- Largely residential

Aranguez

- Agricultural area, one of the only area of open spaces

Bamboo No1

- Small farming community

Valsayn

- Middle and upper income neighbourhoods

North Coast –

- Unauthorized and unplanned development creating a dispersed settlement pattern

Blanchisseuse, Maracas and La Filette

- Bisected by boundary with Tunapuna/Piarco

**Port of Spain**

Characterized by high population density, very diverse (range of commercial and residential, among residential there is a wide range of high and low-income households), becoming increasingly commercial and high traffic congestion.

- Area of 12.3km accounting for 0.3% of land mass and 4% of national population with 49,031 residents in 2000.
- Central Business District as well as adjacent residential and mixed use districts. Very diverse.
- Highest density 3,996 people per sq Km.
- Population growth between 1990 and 2000 is 3.44% compared to national of 4.01%.
- The central areas with declining population is due to conversion of housing to commercial use and communities on periphery have experienced increase in population.
- Land use – 42% commercial, 6% recreational, 52% residential
- In 2003, POS unemployment rate was 5.6% compared to national of 16.5%, DM 2.6% and S/L 11%.
- Employment is characterized by private sector employment.
- Household income of $6215 per capita.
- Educational attainment – primary and secondary 99.4%, secondary and higher is 78.1%
- Percentage of population with chronic illness is 20.6%
- High traffic congestion.
- Disproportionate concentration of schools (excess).
- Inadequate open spaces and contains a large lower income informal spontaneous housing sector with public housing apartments.
- Sex and age distribution shows higher proportion of older persons. 20% of population 60+ compared to national average of 13%.
- Religion – 45% RC, 14% Anglican, 9% Spiritual Baptist and 9% Pentecostal.
- Ethnicity – 54% African, 10% Indian, 32% mixed, 2% Caucasian/Syrian/Chinese
- Marital status 58% never married compared to national average of 49%

Areas within POS

Newtown

- Was residential now commercial

Woodbrook

- Was residential now commercial

St. Clair

- Upper income residential

Belmont

- Street pattern of narrow winding lanes. Lower middle to middle class residential

St James

- Was POS Indian population. Mucurapo mostly residential.

Gonzales

- Poor housing, inadequate roads, lack of open space

East Dry River

- High density residential with mix of commercial and residential. Low income, social marginalization. Significant number of multi storey public housing. Ten of the eleven informal settlements located on State lands with POS are located here. The other is in St James.

**Diego Martin**

Characterized by higher household income, higher standard of living, residential - suburban, high population growth rate and an increase in hillside residential squatting, high educational attainment rate

- Area of 125 km with population of 105,720 in 2000.
- Density of 846 persons per sq km.
- Primarily suburban.
- In 2000, 89.2% suburban, 1.2% industrial, 7.2% rural, 2.4% natural, 24.8% informal hillside development
- Has experienced highest growth rate along with Arima between 1990 and 2000 (14.5%) compared to national of 4% and POS 3.4% and S/L 10.8%.
- Land use – agriculture 5.1%, commercial 1.8%, forest reserve 71.4%, industrial 0.5%, institutional 0.9%, recreational 3%, residential 17.3%
- Sex and age similar to national average with exception of older persons. % 60+ is 17% compared to national average of 13%.
- Ethnicity is 45% African, 10% Indian, 37% mixed, 7% Caucasian/Syrian/Chinese
- Religion is 50% RC, 11%Pentecostal, 10% Anglican
- Educational attainment – primary and secondary 100%, secondary and higher is 72.3%
- Approx 25% live in informal settlements typically on hillsides. These settlements are Dundonald Hill - DM, Sea View Hill - Carenage West, Factory Road Waterfall & Upper Mercer Road both off DM Main road, Upper Carenage Scorpion Village, Jean Avenue- DM, Bagatelle Central including Savannah Trace Nos 1 and 2, L’Anse Mitan- Carenage, Big Yard – Carenage, Bagatelle South – DM, Upper Bagatelle and Patna – DM, River Estate – DM, Simeon Rd – Petit Valley
- Inability to control squatting for lower income groups. Increasing spontaneous hillside settlements moving higher up.
- Unemployment rate of 2.6% lower than national rate of 10.2%. Unemployment among women is 13% higher than national of 12.7%.
- Percentage with chronic illness is 19.7%.
- Household income is $7176 per capita. Higher standard of living.
- The settlements closer to POS and the more urbanized communities along DM main road have experienced a decline in population and suburban and peripheral communities have had population growth.

Areas within DM

Diego Martin

- Stretched out valley. Most intensively built up.

Goodwood Park

- Upper class residential neighbourhood

Cocorite

- Coastal fishing village. Waterhole is a squatting village on slopes of hill.

Westmooring

- High standard residential area.

Glencoe/Bayshore

- Mix of upscale and informal settlements along hillside

Carenage

- Primarily hillside residential. Few facilities.

Chaguaramas

- Extensive rural landscape.

North Coast

Maraval

**Tunapuna/Piarco**

Characterized by high population density, more Indian population than Metro POS, high incidence of chronic disease, university town

- Area of 527.2 km with population of 275000 in 2009
- In 2000 population of 203975 with 55206 households.
- Density of 422 person per sqkm higher than national average of 259. 16.2% of national population reside in 9.9% of total land area.
- Population is concentrated in a band along East West Corridor. Areas of high density are Curepe, Tunapuna Proper, Dinsley, Bon Air, Maloney, La Horquetta and Santa Rosa Heights.
- Employment is mostly in Wholesale and Retail Trade, Manufacturing and Construction in that order.
- University town has developed
- With regard to poverty, only Brasso Seco is among the ten poorest communities in the country.
- Much of the housing has expanded without expansion of physical and social infrastructure
- Residential squatting in some areas such as Bangladesh in St Joseph and Jacob Hill in Wallerfield
- Young population 53.2% under 35 years.
- Educational attainment – primary and secondary 79.1%, secondary and higher is 67.4%
- Percentage of population with chronic illness is 22.8%
- Household income per capita is $6214.
- Age and sex distribution approximate the national average
- Ethnic makeup is 37% African, 32% Indian, 30% mixed.
- Religion – 31% RC, 17% 17% Hindu, 13% Pentecostal, 7% Anglican
- Marital status approximates national average.
- Highest percentage of adult persons with no children 42%. This may be due to university population.
- Relatively high incidence of chronic disease.

Areas within T/P

Caura/Paradise/Tacarigua, Curepe/Pasea, D’Abadie/Carapo, Auzonwille/Tunapuna, Blanchisseuse/Santa Rosa, St. Augustine South/Piarco/St. Helena, Macoya/Trincity, La Florissante/Cleaver, Wallerfield/La Horquetta, Valsayn/St.Joseph, Maracas/Santa Margarita, Bon Air/Arouca/Cane Farm, Kelly Village/Warrenville, Mausica/Maloney, Five Rivers/Lopinot

**Arima**

Characterized by high population density, high incidence of chronic disease, highest percentage of mixed ethnic population, ranked high in Multidimensional Poverty Index after SJ/L in the catchment area

- 1990 was 28612 to 32278 in 2000 growth of 12.8% - 8400 households. 33606 in 2011.
- Second most dense in 2011 with 2801 persons per sqkm.
- Current population is 41000 in 10800 households. A large percentage of active population work outside borough.
- Scored highest in gender inequality. Low female labour force participation.
- High incidence of chronic disease. % of population with chronic illness is 24.8%
- Businesses primarily in retail and distribution sector (54%) and O’Meara industrial estate
- 44% of household heads employed in private enterprises. Majority of household heads employed in service and sales positions.
- Household income is 5,994
- Educational attainment – primary and secondary 71.1%, secondary and higher is 73.6%
- Squatting and informal settlements in Calvary Hill, Jones Town, Malabar and Printeryville
- Sex and age distribution approximate national averages.
- Ethnicity – 34% African, 17% Indian, 49% mixed. Has the largest mixed population.
- Religion - 47% RC, 16% Pentecostal, 5% Anglican

Areas within Arima

Arima, Calvary, O'Meara, Santa Rosa Heights, Tunapuna

**Chaguanas**

Characterized by high population density, high educational attainment, good access to utilities, increasingly commercial, highest population growth in nation, highest % Indian population highest % Hindu population in catchment area

- Population of 71462 in land area of 59km. in 2011 population was 83516 with density of 1416 per sq km.
- 98.9% have primary and secondary educational attainment, 62.9% have secondary and higher educational attainment
- Household income is $5452
- Adolescent fertility rate is 51.9
- Older, educated population. Highest population growth of 2% per yr compared to national average of 0.5%.
- 31% female headed households.
- Good access to utilities
- Land use – predominantly rural, gradually changing with an increase in housing projects. Two main commercial districts in Chaguanas and Enterprise. Developed from an agro-based economy to a trading center.
- Main urban areas are aesthetically unappealing and chaotic.
- Educational attainment – primary and secondary 98.9%, secondary and higher is 62.9%
- Female unemployment is 16.2% higher than national of 12.7%. high gender inequality
- Percentage with chronic illness is 21.1%
- Ethnicity - 26% African, 56% Indian and 17% mixed.
- Religion – 33% Hindu, 16% RC, 13% Pentecostal, 9% Islam
- Highest percentage of married persons – 46% compared to national of 41%

Areas within Chaguanas

Downtown Chaguanas, Montrose, Edinburgh Gardens, Lange Park, Felicity, Orchard Gardens, Endeavour, Enterprise, Longdenville, Cunupia, Carlsen Field

**Sangre Grande**

Largely rural, with the exception of Sangre Grande city. 101 kilometres of Trinidad’s 420 kilometre coastline is within Sangre Grande Municipality. Much of the Municipality falls

within the Northern Basin, from the foothills of the Northern Range to those of the Central Range, consisting of the floodplains and alluvial flats of Oropouche River system and the Northern Terraces and Las Lomas Peneplain. The remaining sections of the Municipality form part of the Central Range with its highest peaks reaching 335 metres. Sangre Grande’s

north Coast consists of a series of cliffs and headlands interspersed with small bays. There are nine wetlands in the municipality, including the North Oropouche and a section of the Nariva Swamp.

- Population of 73,647 in land area of 898 sq km (5.7% of the national population in 18.1% of the total land area, second least densely populated municipality in the country)
- 39% have only completed primary school, while 44% have completed both primary and secondary school (1.7% lower than the national average)
- Ranked among the lowest areas of the country using a national measure of human development (based on the UN’s human development index)
- The region has the highest Multidimensional Poverty Index in the country and the highest rate of adolescent births (75.2 per 1000 women aged 15-19)
- Sangre Grande had the third lowest household income per capita per annum.
- Sangre Grande was among the regions that scored the highest in the gender inequality index, and a slightly lower than national average female labour force participation
- rate (50.1%)
- Access to essential facilities is good, however; only 6% lack access to electricity or clean running water
- The population is relatively young, with more than half of the population (57%) under 35 years, and 25% aged 0-15.
- Between 2000 and 2011, the population grew by 15.4%, making it the country’s fastest-growing region.
- Most of the land is either forest cover or agricultural
- Percentage with chronic illness is 18%
- Ethnicity - 31% African, 32% Indian and 37% mixed.
- Religion – 28% Roman Catholic, 17% Hindu, 14% Pentecostal, 9% Seventh Day Adventist, 7% Baptist, 4% Islam, 4% Anglican
- Economy is based on agriculture and fisheries, although environmental damage by the offshore oil and gas industries have threatened fishing livelihoods
- Second lowest level of reported serious crime per 100,000 persons in the country

Areas within Sangre Grande

Town of Sangre Grande; Greater Sangre Grande; Matura to Matelot (North-East corner of the island, most villages on the coast); Valencia; Greater Tamana; Manzanilla; Fishing Pond/North Manzanilla
